# Supplementary material for: Targeted degradation of USP7 in solid cancer cells reveals distinct effects of deubiquitinase degraders and inhibitors
Source: Nat Commun. 2026 May 13;17:4331. doi: 10.1038/s41467-026-72295-x (PMC13172357; doi:10.1038/s41467-026-72295-x)
Supplement: Supplementary file 1 — Supplementary Information [file 41467_2026_72295_MOESM1_ESM.pdf]

Supplementary Information

Targeted degradation of USP7 in solid cancer cells reveals distinct effects of deubiquitinase degraders and inhibitors

Nikolas Klink, Sebastian Urban, Johanna A. Seier *et al.*

Table of Contents

1. Supplementary Figures 1-7 .... page 2

2. Supplementary Tables .... page 9

3. Chemical synthesis procedures .... page 12

   3.1 Synthesis of USP7 inhibitor .... page 13

   3.2 Synthesis of functionalized USP7 inhibitors .... page 15

   3.3 Synthesis of further functionalized USP7 inhibitors .... page 19

   3.4 Synthesis of acid-functionalized VHL ligands .... page 23

   3.5 Synthesis of an amine-functionalized VHL ligand .... page 28

   3.6 Synthesis of PROTACs from acid-functionalized USP7 ligands .... page 29

   3.7 Synthesis of VHL-alkylcarboxy-functionalized PROTACs .... page 34

   3.8 Synthesis of VHL-cyclohexyl-functionalized PROTACs .... page 37

   3.9 Synthesis of VHL-cubane-functionalized PROTACs .... page 40

   3.10 Synthesis of enhanced PROTACs NK250 and NK266 .... page 43

   3.11 Synthesis of a VHL inhibitor .... page 45

4. NMR spectra of compounds .... page 46

5. Supplementary References .... page 87

1. Supplementary Figures 1-7

a

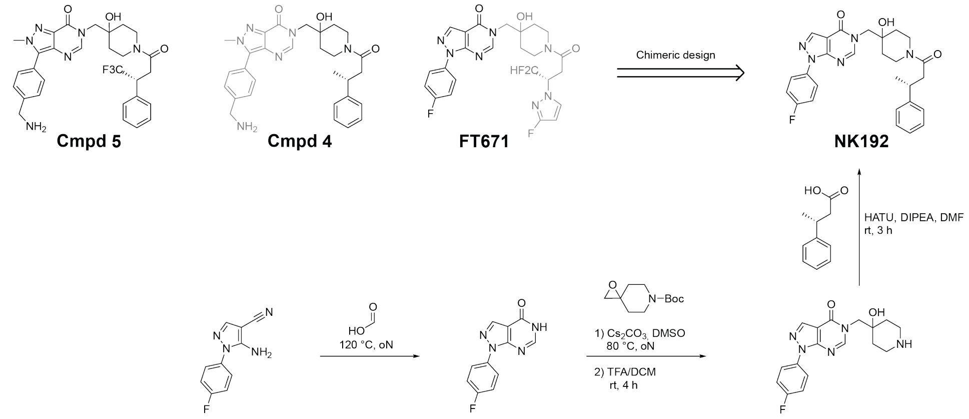

b

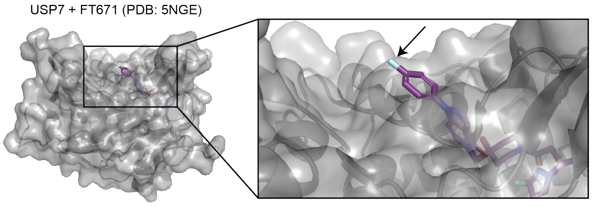

Supplementary Figure 1. Design of chimeric USP7 inhibitor NK192 and of derivatives.

a. Chemical structures of previously described USP7 inhibitors Compound 5[1], Compound 4[1] and FT671[2] which were used for the design of chimeric USP7 inhibitor NK192. Its chemical synthesis is shown. Detailed synthesis procedures are shown in the chemistry methods section. **b.** Crystal structure of USP7 in complex with FT671 inhibitor (PDB: 5NGE).[2] A close-up view of the binding site is shown with the solvent-exposed 4-fluorophenyl group (which was use as exit vector for further functionalization) highlighted with an arrow.

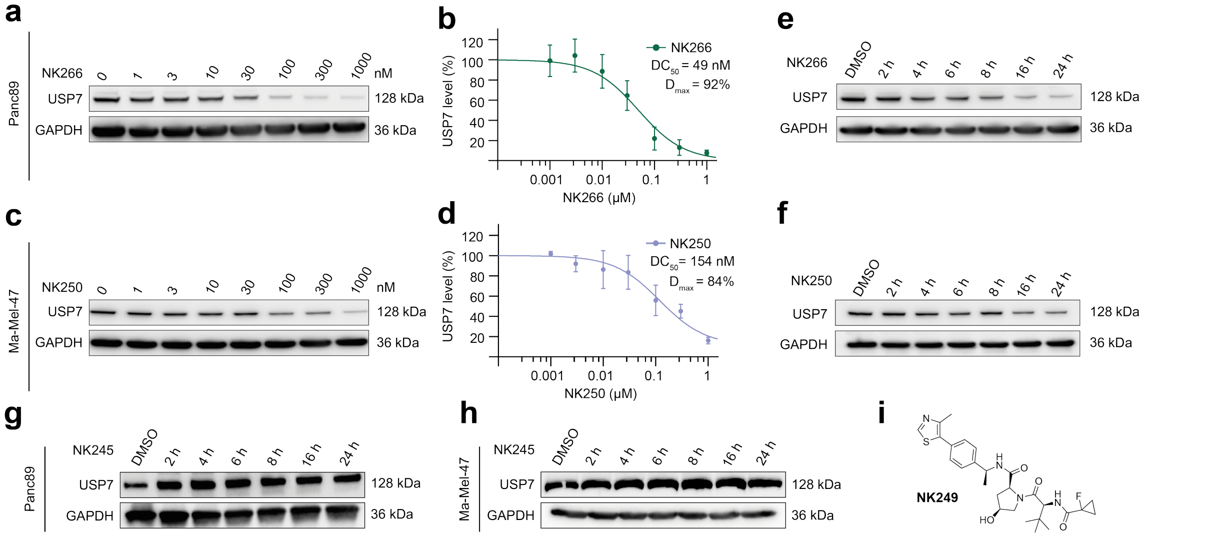

Supplementary Figure 2. E3 ligase engagement is required for USP7 degradation.

a. Assessment of degradation efficiency of PROTAC NK266 in Panc89 cells, treated with indicated compound concentrations for 24 h. **b**. Quantification of degradation efficiency of NK266 in Panc89 cells. Data are shown as mean ± s.d. (n=3 independent experiments). **c**. Assessment of degradation efficiency of PROTAC NK250 in Ma-Mel-47 cells, treated with indicated compound concentrations for 24 h. **d**. Quantification of degradation efficiency of NK250 in Ma-Mel-47 cells. Data are shown as mean ± s.d. (n=3 independent experiments). **e.-f**. Degradation kinetics. Western blot analysis of Panc89 cells treated with NK266 (1 µM) (e) or of Ma-Mel-47 cells treated with NK250 (1 µM) (f) for indicated timepoints. **g.-h.** USP7 degradation assays with negative control compound NK245. Western blot analysis of Panc89 (g) and Ma-Mel-47 (h) cells treated with NK245 (1 µM) for indicated times. See Fig. 3a for the structure and rational of NK245. **i**. Chemical structure of VHL-ligand NK249. Uncropped blots are provided as Source Data.

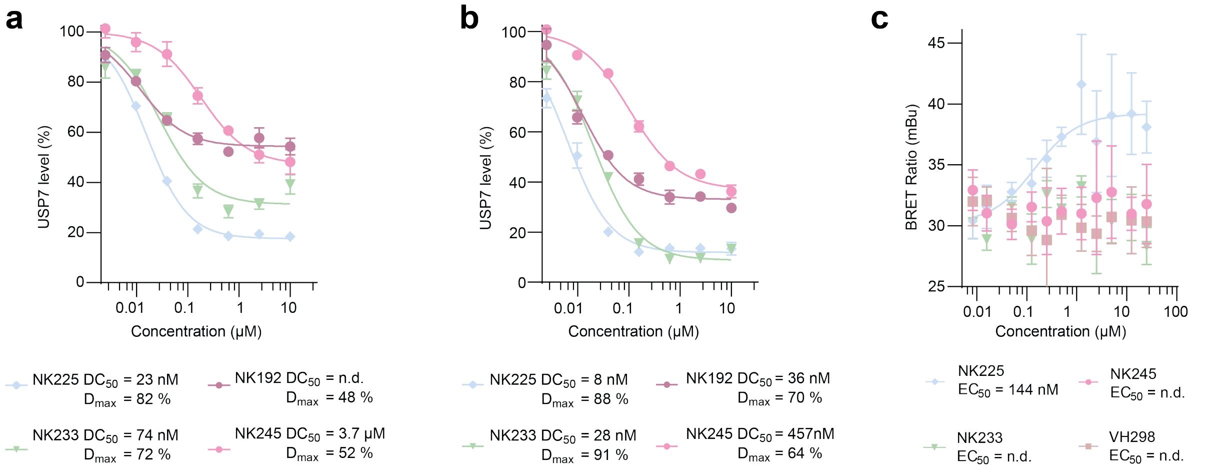

Supplementary Figure 3. Quantitative assessments of USP7 degradation efficiency and of ternary complex formation of first generation PROTAC molecules and control compounds.

a.-b. Assessment of USP7 degradation using HiBiT endpoint degradation assays in MV4-11 cells expressing HiBiT-USP7. Cells were treated for either 6 h (a) or 24 h (b) with NK192, NK225, NK233 or NK245 at varying concentrations. Data are shown as mean ± s.d. (n=3). Half-maximal degradation concentrations (DC_{50}) and maximal percentage of protein degradation values (D_{max}) derived from these data are given below. **c.** Cellular ternary complex formation assay using PROTACs NK225 and NK233, as well as negative control PROTAC NK245 and VHL ligand VH298. The determined half-maximal ternary complex formation concentration (EC_{50}) for NK225 is given below. Data are shown as mean ± s.d. (n=4). mBu, milli BRET units.

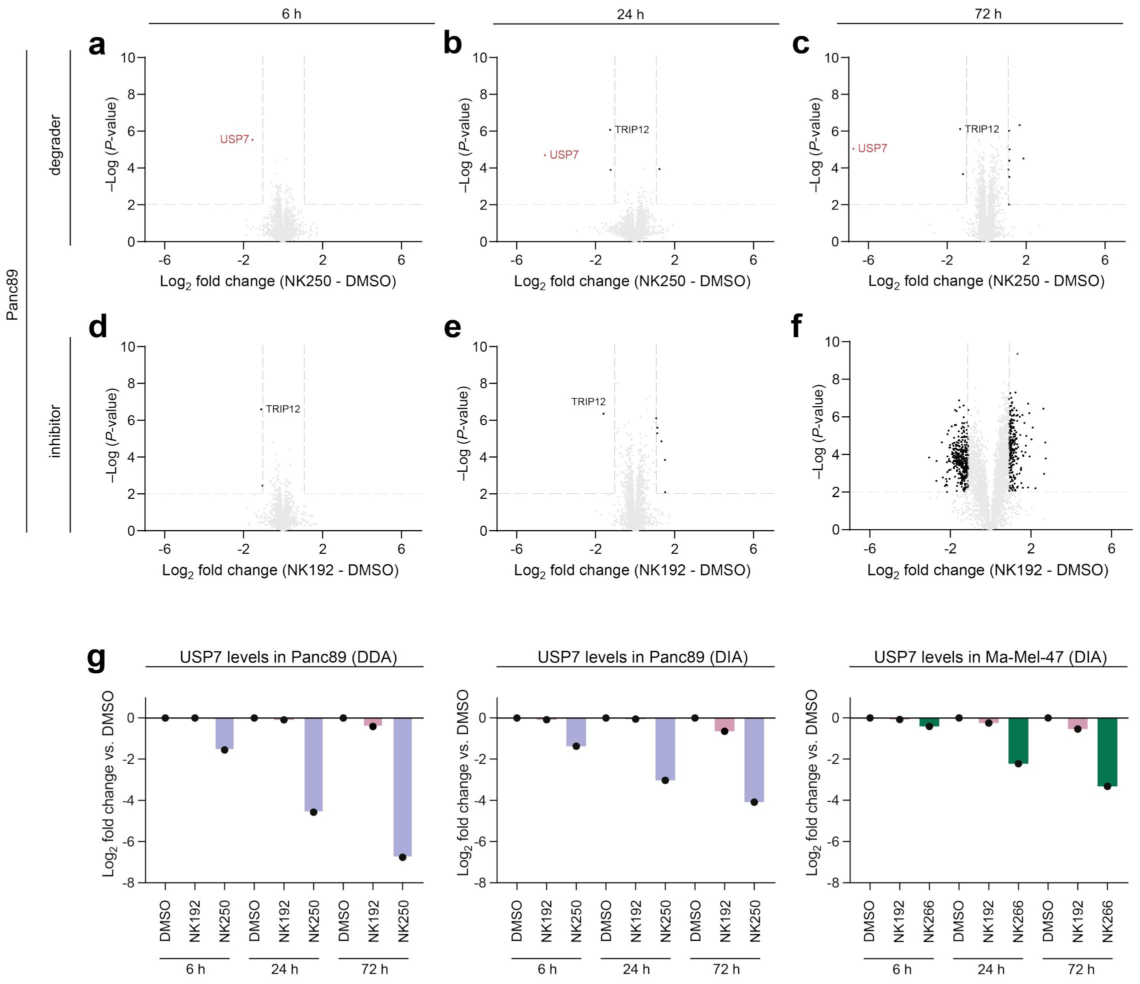

Supplementary Figure 4. Proteomic analysis of USP7 modulation in Panc89 cells reveals exceptional USP7 degradation specificity of NK250.

a.-f. Proteomic analysis of Panc89 cells treated with PROTAC NK250 (a-c) or inhibitor NK192 (d-f) for indicated time points (6, 24 and 72 h). Volcano blots reporting quantitative analysis of proteins identified by mass spectrometry through data-dependent acquisition (DDA). Significantly regulated proteins are colored in black, USP7 is highlighted in red. See Fig. 5a-f for an analysis of the same samples through data-independent acquisition (DIA) mass spectrometry. *P*-values were determined from two-sided *t*-tests as implemented in Perseus. **g.** Log_2 fold changes of USP7 protein levels compared to DMSO across all measured timepoints and for both DDA and DIA approaches. Shown is the average of the quadruplicates of each condition.

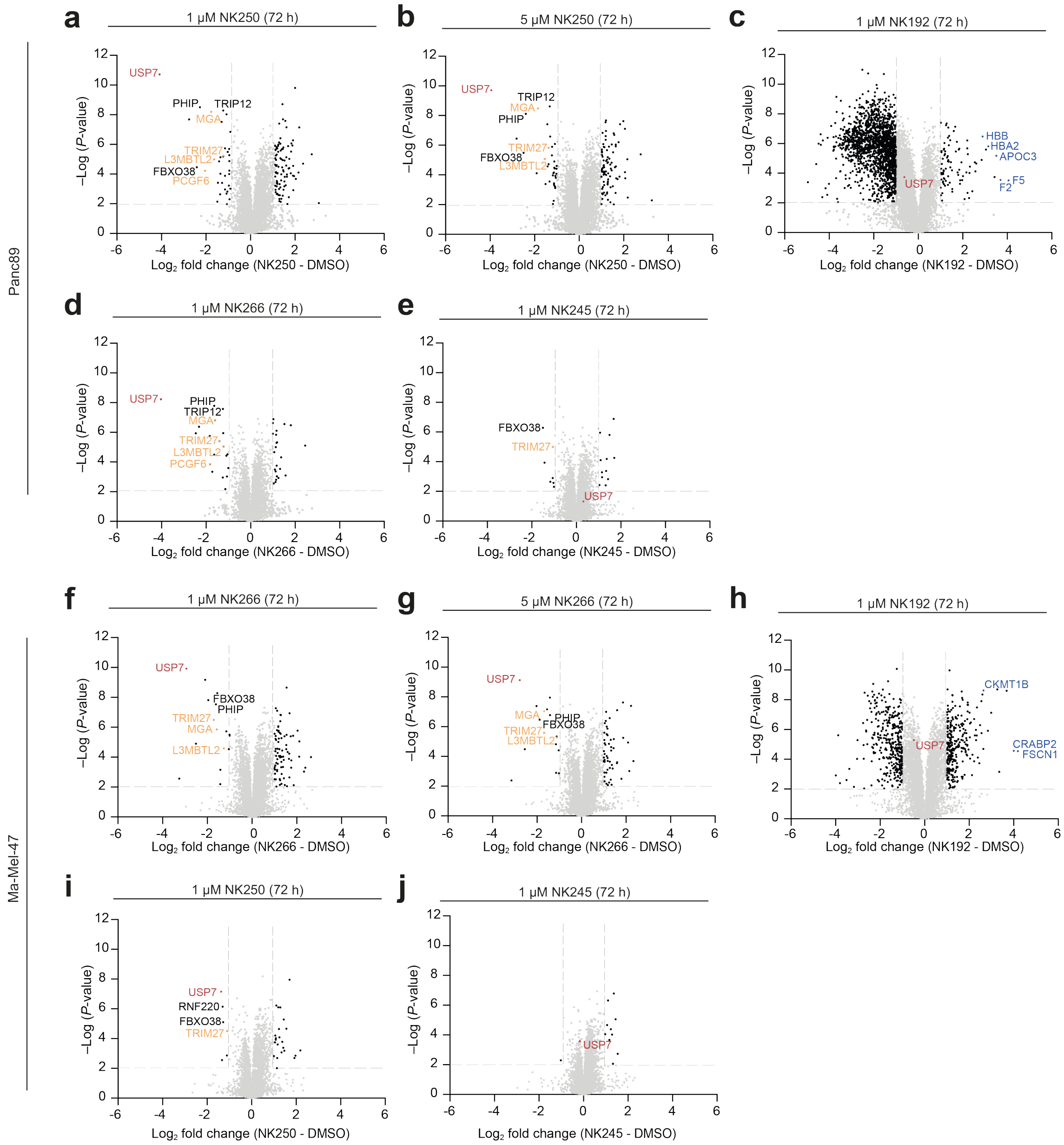

Supplementary Figure 5. Dose-matched proteomic analysis confirms different effects elicited by USP7 inhibitor and degraders.

a.-j. Volcano blots reporting quantitative analysis of proteins identified by mass spectrometry through data-independent acquisition (DIA). Panc89 cells were treated with PROTAC NK250 (a-b), inhibitor NK192 (c), PROTAC NK266 (d) or negative control NK245 (e) for 72 h at indicated concentrations. Ma-Mel-47 cells were treated with PROTAC NK266 (f-g), inhibitor NK192 (h), PROTAC NK250 (i) and negative control NK245 (j) for 72 h at indicated concentrations. Members of the non-canonical Polycomb repressive complex 1.6 are highlighted in orange, chromatin-bound E3 ligases in black, USP7 in red. Selected upregulated proteins upon inhibitor treatment consistent with Fig. 5 are highlighted in blue. See Fig. 5 (panels c, f, i, l) for the corresponding dose-matched analyses. *P*-values were determined from two-sided *t*-tests as implemented in Perseus.

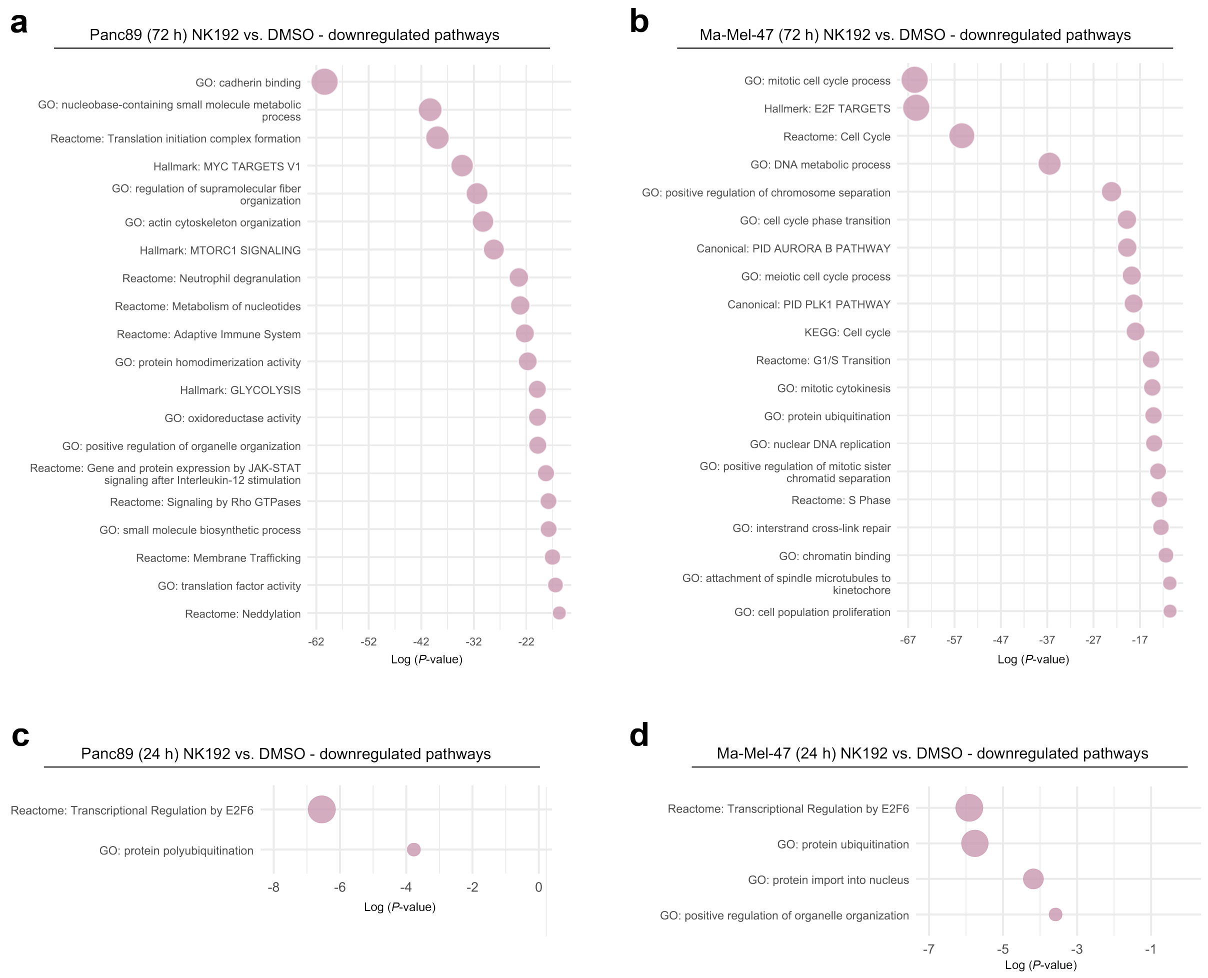

Supplementary Figure 6. Metascape pathway analysis of significantly regulated proteins identified by mass spectrometry analysis in PDAC and melanoma cell lines following inhibitor treatment.

a-d. Pathway enrichment analysis of significantly downregulated proteins in Panc89 cells treated for 72 h (a) or 24 h (c) with NK192 (a) and Ma-Mel-47 cells treated for 72 h (b) or 24 h (d) with NK192.

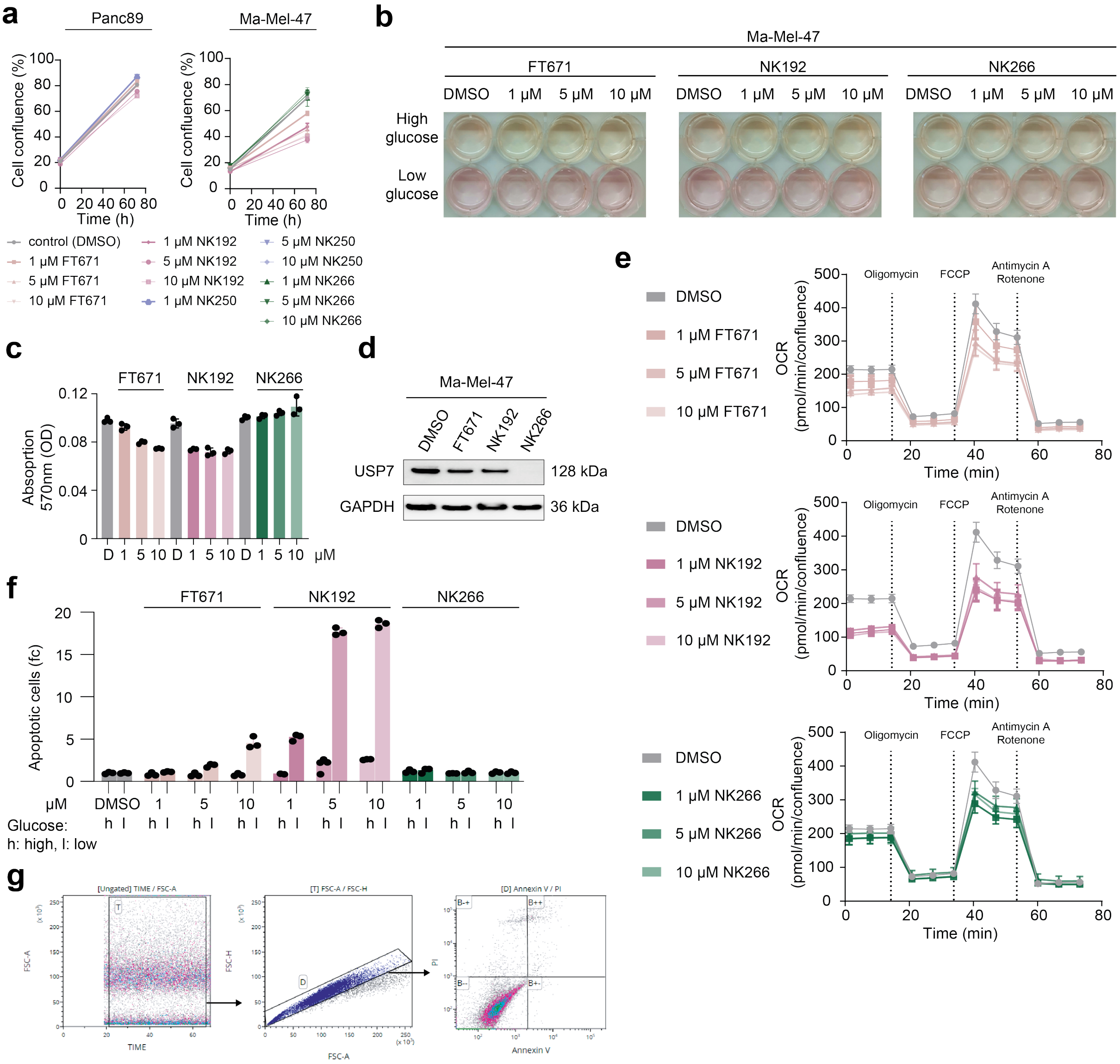

Supplementary Figure 7. Disparate effects of inhibitor vs. PROTAC in Ma-Mel-47.

a. Confluence of Panc89 (left) and Ma-Mel-47 (right) cells treated with FT671, NK192 or NK250 or NK266 at 1, 5 or 10 µM versus DMSO after 72 h. Data are shown as mean ± s.d. (n=3 independent experiments). **b.** Images of Ma-Mel-47 cells in culture medium containing 5 mM (low) or 11 mM (high) glucose, treated with indicated concentrations of FT671, NK192 and NK266 after 72 h. **c**. Photometric analysis of Ma-Mel-47 cells treated with indicated concentrations of FT671, NK192 or NK266 in culture medium containing 11 mM (high) glucose for 72h. Data are shown as mean ± s.d. (n=3 independent experiments). **d.** Western blot analysis of Ma-Mel-47 cells treated with 1 µM of indicated compounds for 72 h. **e.** Seahorse mito stress test. Ma-Mel-47 cells treated with indicated concentrations of FT671, NK192 or NK266 for 24 h, followed by OCR measurements. Three independent experiments were performed, in which at least triplicates were used for data analysis; one representative is shown as mean ± s.e.m. (n=3-5 independent experiments). **f.** Apoptosis assay. Ma-Mel-47cells treated with compounds for 72 h in culture medium containing 5 mM (low) or 11 mM (high) glucose were analyzed by FACS for Annexin-V staining. Data were analyzed as in Fig. 7e. Data are shown as mean ± s.d. (n=3 independent experiments). **g.** FACS gating strategy. Events were first gated on time versus FSC-A to exclude acquisition artefacts, followed by singlet discrimination using FSC-A versus FSC-H and assigned by subsequent quadrant gating. Uncropped blots are provided as Source Data.

2. Supplementary Tables

Supplementary Table 1. Mass spectrometry settings for DDA analysis (ACE_0869 and ACE_0882-DDA).

general	*MS1*	*MS2*	*Comments; special settings*
Tune v4.1.4244<br>Xcalibur v4.7.69.37<br>SII: 1.7.0.468<br>Gradient: 105 min	Analyzer: FT<br>Res.: 120000<br>SR: 375 - 1500<br>AGC: Standard<br>AGC abs.: 400000<br>AcT: 50 ms<br>RF: 30<br>SF: --<br>DDM: CT/3sec	Analyzer: IT<br>Res./ScR: -/rapid<br>SR: Auto<br>AGC: 300%<br>AGC abs.: 30000<br>AcT: 70 ms<br>CS: +2 to +7<br>IsM: Q<br>IsW: 1.6<br>Frag.: sHCD<br>NCE: 20, 30, 45	classic orbitrap experiment: MS1 in Orbitrap at high resolution and data dependent MS2 also in Orbitrap high resolution. Dynamic exclusion enabled (exclude after n times=1; Exclusion duration (s)= 30; mass tolerance= ± 10 ppm)<br><br>Intensity Threshold: 5000<br>Ion transfer Tube Temp: 230 °C<br>Ion Source Voltage: 2500 V

Note: **FT**= Fourier Transform (Orbitrap); **IT**= Iontrap; **Q**= Quadrupol; **Res.**= max. Resolution at 200 m/z (Lumos) or 400 m/z (Elite) [FWHM (full width at half maximum)]; **ScR**= scan rate for measurements in the IT; **SR**= scan range [m/z]; **AGC**= automatic gain control, max number of acquired ions per measurement; **AcT**= max. Ion acquisition time [ms]; **CS**= charge states used for fragmentation; **IsM**= Isolation mode (Q or IT), MS2 isolation and further is only done in IT; **IsW**= Isolation window [m/z], value followed by scan mode the isolation is based on (MS1, MS2 …) **Frag.**= Fragmentation method; **HCD**= Higher-energy collisional dissociation; **CID**= Collision-induced dissociation; **ETD**= Electron-transfer dissociation**; EThcD=** Electron-Transfer/Higher-Energy Collision Dissociation; **sHCD**= stepped HCD**; NCE**= normalized collision energy; **cycles**: number of MSn recorded or max cycle time; RF= RF Lens [%]; **SF**= Source Fragmentation [V]; **DDM**: Data dependent Mode (cycle time in seconds, CT/[s] or number of scans, NS); **NS**= Number of data dependent scans; **NSE**: Number of Scan Events; **WO**: Window overlap; **PMR**: Precursor Mass Range

Supplementary Table 2. Mass spectrometry settings for DIA analysis (ACE_0882-DIA and ACE_0883-DIA).

General	MS1	MS2	MS1	MS2	Comments; special settings
	Experiment 1	Experiment 2 DIA 400-1000 with 8 mz window	Experiment 3	Experiment 4 DIA with windows staggered	
Tune v4.1.4244<br>Xcalibur v4.7.69.37<br>SII: 1.7.0.468<br>Gradient: 105 min	Analyzer: FT<br>Res.: 60000<br>SR: 390 - 1010<br>AGC: Standard<br>AGC abs.: 400000<br>AcT: auto<br>RF: 30<br>SF: --<br>DDM: -/-	PMR: 400-1000<br>Analyzer: FT<br>Res./ScR: 15000/-<br>SR: 145-1450<br>AGC: 800%<br>AGC abs.: 400000<br>AcT: auto<br>CS: ---<br>IsM: Q<br>IsW: 8<br>WO: 0<br>NSE: 75<br>Frag.: HCD<br>NCE: 33	Analyzer: FT<br>Res.: 60000<br>SR: 390 - 1010<br>AGC: Standard<br>AGC abs.: 400000<br>AcT: auto<br>RF: 30<br>SF: --<br>DDM: -/-	PMR: 396-1004<br>Analyzer: FT<br>Res./ScR: 30000/-<br>SR: 145-1150<br>AGC: 800%<br>AGC abs.: 400000<br>AcT: 60 ms<br>CS: ---<br>IsM: Q<br>IsW: 4<br>WO: 0<br>NSE: 152<br>Frag.: HCD<br>NCE: 33	DIA orbitrap experiment. MS1 followed by fast DIA-MS2 followed by MS1 and slower DIA-MS2 with staggered windows.<br><br>Intensity Threshold: 5000<br>Ion transfer Tube Temp: 275 °C<br>Ion Source Voltage: 2300 V<br>Lock mass: 445.12002<br>Default charge State: +3

Note: See the footnote of Supplementary Table 1 for a list of abbreviations.

Supplementary Table 3. Mass spectrometry settings for DIA analysis (ACE_1029-DIA and ACE_1030-DIA).

General	MS1	MS2	MS1	MS2	Comments; special settings
	Experiment 1	Experiment 2 DIA 400-1000 with 8 mz window	Experiment 3	Experiment 4 DIA with windows staggered	
Tune v4.1.4244<br>Xcalibur v4.7.69.37<br>SII: 1.7.0.468<br>Gradient: 140 min	Analyzer: FT<br>Res.: 60000<br>SR: 390 - 1010<br>AGC: Standard<br>AGC abs.: 400000<br>AcT: auto<br>RF: 30<br>SF: --<br>DDM: -/-	PMR: 400-1000<br>Analyzer: FT<br>Res./ScR: 15000/-<br>SR: 145-1450<br>AGC: 800%<br>AGC abs.: 400000<br>AcT: auto<br>CS: ---<br>IsM: Q<br>IsW: 8<br>WO: 0<br>NSE: 75<br>Frag.: HCD<br>NCE: 33	Analyzer: FT<br>Res.: 60000<br>SR: 390 - 1010<br>AGC: Standard<br>AGC abs.: 400000<br>AcT: auto<br>RF: 30<br>SF: --<br>DDM: -/-	PMR: 396-1004<br>Analyzer: FT<br>Res./ScR: 30000/-<br>SR: 145-1450<br>AGC: 800%<br>AGC abs.: 400000<br>AcT: 60 ms<br>CS: ---<br>IsM: Q<br>IsW: 4<br>WO: 0<br>NSE: 152<br>Frag.: HCD<br>NCE: 33	DIA orbitrap experiment. MS1 followed by fast DIA-MS2 followed by MS1 and slower DIA-MS2 with staggered windows.<br><br>Intensity Threshold: 5000<br>Ion transfer Tube Temp: 275 °C<br>Ion Source Voltage: 2300 V<br>Lock mass: 445.12002<br>Default charge State: +3

Note: See the footnote of Supplementary Table 1 for a list of abbreviations.

3. Chemical synthesis procedures

The chemicals and solvents used for this work were purchased from companies such as Activate Scientific, BLDpharm, Honeywell, Fisher Scientific, Merck, Roth, Sigma-Aldrich, TCI or VWR and were used without further purification.

Chemicals and solvents used for the synthesis are abbreviated as follows. EA: ethyl acetate, DCM: dichloromethane, MeOH: methanol, PE: petroleum ether, DMF: dimethylformamide, DMSO: dimethyl sulfoxide THF: tetrahydrofuran, TFA: trifluoroacetic acid, ACN: acetonitrile, H_2O: water, EtOH: ethanol.

Silica gel aluminum plates (silica gel 60 F254, Merck) were used for thin-layer chromatography. The detection was carried out using UV light at 254 nm or potassium permanganate as staining reagent.

A Pure C-850 FlashPrep system or a Pure-C-810 Flash system (Büchi) were used for automated column chromatographic purification. For preparative reverse phase column chromatographic purification, a VP125/21 Nucleodur C18 Gravity column (5 µm, Macherey Nagel) was used; for low pressure reverse phase column chromatographic purification various Ecoflex C18 columns (Büchi) were used. Compound purification by preparative HPLC was achieved with the following gradients: 0% ACN in H_2O + 0.1% TFA over 6 min, 0-10% ACN in H_2O + 0.1% TFA over 3 min, 10-55% ACN in H_2O + 0.1% TFA over 30 min, 100% ACN + 0.1% TFA over 7 min) or: 0% ACN in H_2O + 0.1% TFA over 6 min, 15-70% ACN in H_2O + 0.1% TFA over 40 min, 100% ACN + 0.1% TFA over 7 min).

A 1260 series HPLC system (Agilent Technologies) with a ZORBAX Eclipse XDB column (C18 80 Å; 4.6 x 150 mm; 5 µm) was used for low resolution LC-MS analysis.

For high resolution mass spectrometry (HRMS) a 1260 Infinity II system (Agilent Technologies) using a G7129A autosampler, a G7116A column oven, a G7117C photodiode array detector and a G7111B quaternary pump system was used. The measurement was performed on a compact QTOF (Bruker Daltonics). The ionization mode was ESI (Electrospray ionization) with a source voltage of 4.5 kV.

The following devices from Bruker were used to record NMR spectra: AV 500 Avance III HD (500 MHz for ^{1}H and 125 MHz for ^{13}C-NMR), AV 600 Avance III HD (600 MHz for ^{1}H and 151 MHz for ^{13}C NMR) and AV 700 Avance III HD (700 MHz for ^{1}H and 176 MHz for ^{13}C NMR).

The chemical shifts of all spectra are specified in ppm and the coupling constants *J* are given in Hertz (Hz). Peaks of deuterated solvent were used as internal standards (DMSO-d_6: δ = 2.50 ppm / 39.52 ppm; $CDCl_3$: δ = 7.26 ppm / 77.16 ppm). Spectra were analyzed with MestreNova.

The multiplicities of the signals in the ^{1}H spectra are abbreviated as follows: s (singlet), d (doublet), dd (doublet of doublets), t (triplet), td (triplet of doublets), q (quartet), m (multiplet) and b (broad).

Compounds including the (*R*)-3-phenylbutanoic acid moiety (such as NK192) showed splitting of some of the ^{13}C signals, as described before for molecules including this moiety[1,3] due to slowing exchanging rotamers (conformers).

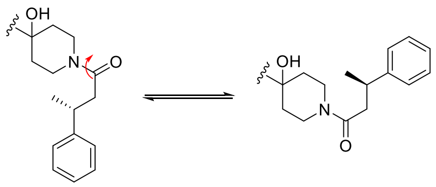

In line with previously published annotations, signals showing this splitting are annotated as “d” as apparent doublet[1].

3.1 Synthesis of a USP7 inhibitor

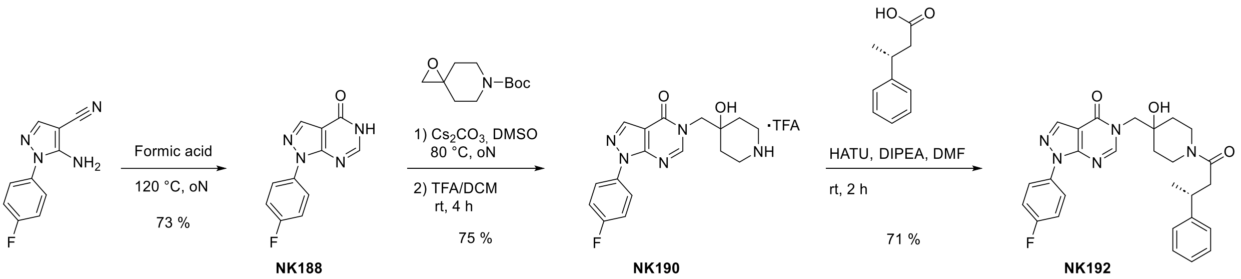

Supplementary Figure 8. Synthesis of NK192.

Synthesis of 1-(4-fluorophenyl)-1,5-dihydro-4*H*-pyrazolo[3,4-*d*]pyrimidin-4-one (NK188)

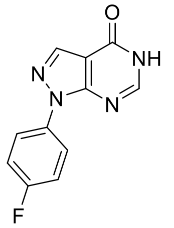

5-Amino-1-(4-fluorophenyl)-1*H*-pyrazole-4-carbonitrile (5.00 g, 24.7 mmol) was dissolved in 150 mL of formic acid. The mixture was heated to 120°C and stirred overnight. After completion, the mixture was cooled to rt for 15 min and then poured into 250 mL of ice-cold water. The mixture was filtered, and the precipitate was washed with ice-cold water (3 x 100 mL). The resulting solids were collected and dried under reduced pressure which yielded 1-(4-fluorophenyl)-1,5-dihydro-4*H*-pyrazolo[3,4-*d*]pyrimidin-4-one (NK188) (4.14 g, 18.0 mmol, 73% yield) as an off-white powder.

HRMS (m/z) $[M+H]^+$ calculated for $C_{11}H_8FN_4O^+$: 231.0667, found: 231.0675.

^1H NMR (500 MHz, DMSO-d_6) δ (ppm): 12.46 (s, 1H), 8.33 (s, 1H), 8.20 (s, 1H), 8.06 (dd, *J* = 8.9, 4.9 Hz, 2H), 7.42 (t, *J* = 8.8 Hz, 2H).

^{13}C NMR (126 MHz, DMSO-d_6) δ (ppm): 160.61 (d, *J* = 244.3 Hz), 157.17, 151.79, 148.93, 136.04, 134.62 (d, *J* = 2.9 Hz), 123.87 (d, *J* = 8.6 Hz), 116.07 (d, *J* = 22.8 Hz), 107.52.

Synthesis of 1-(4-fluorophenyl)-5-((4-hydroxypiperidin-4-yl)methyl)-1,5-dihydro-4*H*-pyrazolo[3,4-*d*]pyrimidin-4-one TFA salt (NK190)

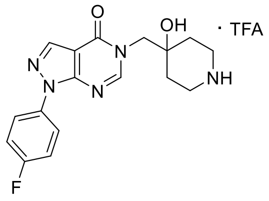

Step1:

NK188 (3.0 g, 13.0 mmol, 1.0 eq), *tert*-butyl 1-oxa-6-azaspiro[2.5]octane-6-carboxylate (4.17 g, 19.6 mmol, 1.5 eq), and cesium carbonate (12.74 g, 39.1 mmol, 3 eq) were added to a dry round bottom flask to which was added dry DMSO (30 mL). The reaction was heated to 80°C and stirred overnight. After completion, the mixture was diluted with water (100 mL) and then extracted with DCM (3x 100 mL). The combined organic layers were dried over $MgSO_4$ and concentrated under reduced pressure. The resulting oil was purified by reverse phase flash column chromatography (ACN/H_2O + 0.1 % TFA) yielding *tert*-butyl 4-((1-(4-fluorophenyl)-4-oxo-1,4-dihydro-5*H*-pyrazolo[3,4-*d*]pyrimidin-5-yl)methyl)-4-hydroxypiperidine-1-carboxylate (NK189) (4.31 g, 9.73 mmol, 75 % yield) as an off-white powder.

HRMS (m/z) $[M+H]^+$ calculated for $C_{22}H_{26}FN_5NaO_4^+$: 466.1861, found: 466.1850.

^1H NMR (500 MHz, DMSO-d_6) δ (ppm): 8.37 (s, 1H), 8.34 (s, 1H), 8.09 – 8.05 (m, 2H), 7.45 – 7.40 (m, 2H), 4.90 (s, 1H), 4.03 (s, 2H), 3.66 (d, *J* = 13.1 Hz, 2H), 3.04 (s, 2H), 1.48 (ddd, *J* = 13.2, 11.2, 4.7 Hz, 2H), 1.39 (s, 9H), 1.38 – 1.33 (m, 2H).

^{13}C NMR (126 MHz, DMSO-d_6) δ (ppm): 160.61 (d, *J* = 244.3 Hz), 156.86, 153.82, 152.39, 151.08, 136.33, 134.53 (d, *J* = 2.8 Hz), 123.71 (d, *J* = 8.6 Hz), 116.14 (d, *J* = 23.0 Hz), 106.64, 78.52, 68.99, 53.05, 34.21, 28.08.

Step 2:

NK189 (4.31 g, 9.84 mmol) was dissolved in TFA/DCM (50:50, 30 mL) and stirred at rt for 3 h. After completion, the solvents were evaporated under reduced pressure. The resulting off-white solids of the product 1-(4-fluorophenyl)-5-((4-hydroxypiperidin-4-yl)methyl)-1,5-dihydro-4*H*-pyrazolo[3,4-*d*]pyrimidin-4-one TFA salt (NK190) (4.5 g, 9.84 mmol, quant. yield) were used for the next step without further purification.

HRMS (m/z) $[M+H]^+$ calculated for $C_{17}H_{19}FN_5O_2^+$: 344.1517, found: 344.1510.

Synthesis of (*R*)-1-(4-fluorophenyl)-5-((4-hydroxy-1-(3-phenylbutanoyl)piperidin-4-yl)methyl)-1,5-dihydro-4*H*-pyrazolo[3,4-*d*]pyrimidin-4-one (NK192)

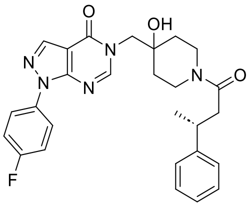

(*R*)-3-Phenylbutanoic acid (10.4 mg, 0.06 mmol, 1.2 eq), DIPEA (34.0 mg, 0.26 mmol, 5 eq) and HATU (26.03 mg, 0.07 mmol, 1.3 eq) were dissolved in DMF (1 mL) and stirred at rt for 10 minutes. To this was added NK190 (20.0 mg, 0.05 mmol, 1 eq) and the reaction was stirred for 2 h until completion. The solvent was evaporated under reduced pressure and the resulting crude product was directly purified by preparative reverse phase column chromatography. The product (*R*)-1-(4-fluorophenyl)-5-((4-hydroxy-1-(3-phenylbutanoyl)piperidin-4-yl)methyl)-1,5-dihydro-4*H*-pyrazolo[3,4-*d*]pyrimidin-4-one (NK192) (18.4 mg, 0.04 mmol, 71 % yield) was obtained as a white powder.

HRMS (m/z) $[M+Na]^+$ calculated for $C_{27}H_{28}FN_5NaO_3^+$: 512.2068, found: 512.2076.

^{1}H NMR (500 MHz, DMSO-d_6) δ (ppm): 8.37 (s, 1H), 8.33 (d, *J* = 11.5 Hz, 1H), 8.09 – 8.05 (m, 2H), 7.45 – 7.41 (m, 2H), 7.26 (dd, *J* = 8.1, 5.9 Hz, 4H), 7.16 (ddt, *J* = 6.6, 5.0, 2.3 Hz, 1H), 4.91 (s, 1H), 4.09 – 3.98 (m, 2H), 3.95 (d, *J* = 2.8 Hz, 1H), 3.65 (ddd, *J* = 14.6, 9.4, 4.7 Hz, 1H), 3.25 – 3.12 (m, 2H), 2.91 – 2.83 (m, 1H), 2.65 – 2.53 (m, 2H), 1.57 – 1.24 (m, 4H), 1.20 (d, *J* = 6.9 Hz, 3H).

^{13}C NMR (126 MHz, DMSO-d_6) δ (ppm): 169.13 (“d”, *J* = 2.2 Hz), 160.62 (d, *J* = 244.4 Hz), 156.82 (“d”, *J* = 6.8 Hz), 152.36 (“d”, *J* = 2.5 Hz), 151.08 (“d”, *J* = 1.5 Hz), 146.64 ("d", *J* = 13.5 Hz), 136.33, 134.53 (d, *J* = 3.0 Hz), 128.21 (“d”, *J* = 4.5 Hz), 126.91 (“d”, *J* = 3.7 Hz), 125.95 (“d”, *J* = 5.0 Hz), 123.72 (d, *J* = 8.5 Hz), 116.16 (d, *J* = 23.1 Hz), 106.66, 69.11 (“d”, *J* = 6.1 Hz), 53.06, 41.00 (“d”, *J* = 14.5 Hz), 40.24 (“d”, *J* = 3.2 Hz), 36.92, 36.12 (“d”, *J* = 26.3 Hz), 34.89 (“d”, *J* = 13.9 Hz), 34.17 (“d”, *J* = 16.7 Hz), 21.99 (“d”, *J* = 25.1 Hz).

3.2 Synthesis of functionalized USP7 inhibitors

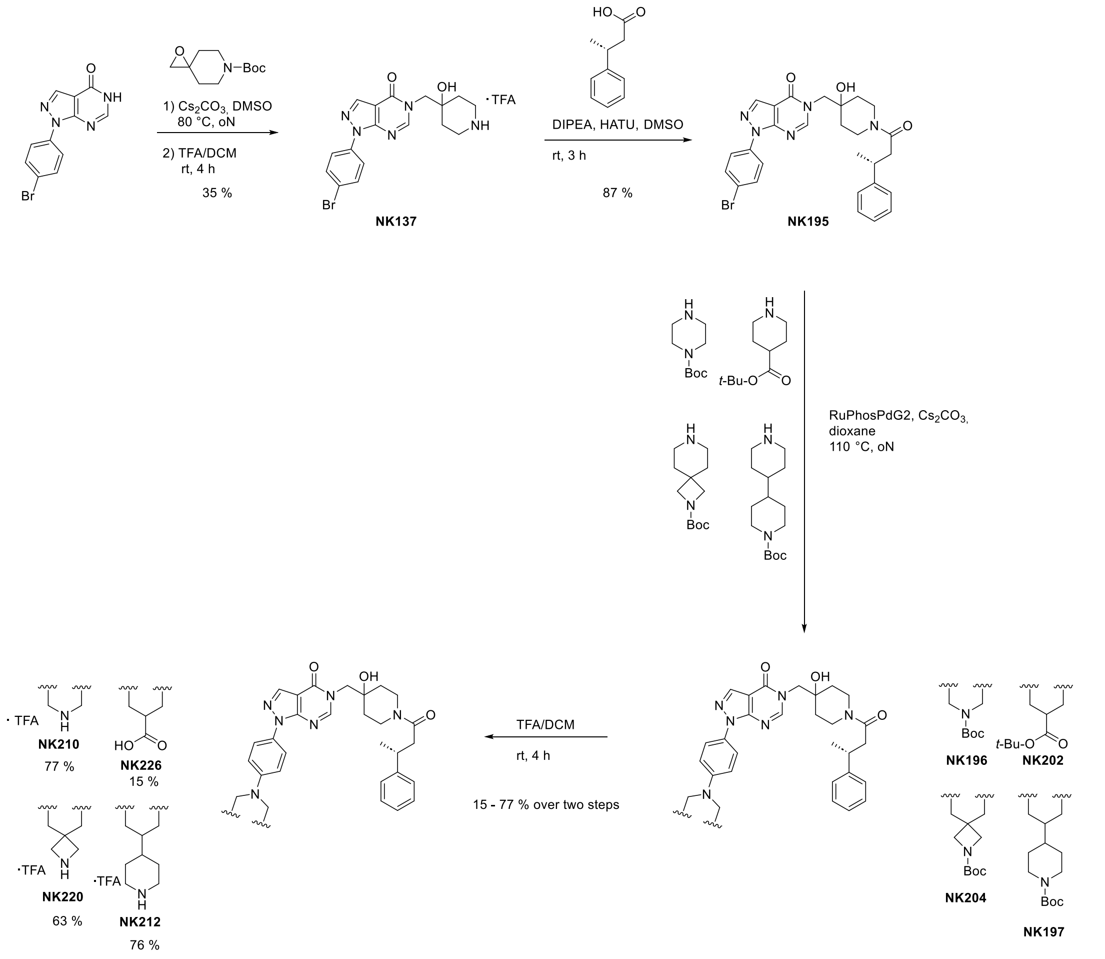

Supplementary Figure 9. Synthesis of USP7 inhibitors NK210, NK226, NK220 and NK212.

Synthesis of 1-(4-bromophenyl)-5-((4-hydroxypiperidin-4-yl)methyl)-1,5-dihydro-4*H*-pyrazolo[3,4-*d*]pyrimidin-4-one (NK137)

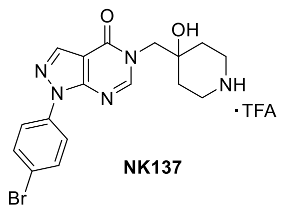

Step 1:

To a solution of 1-(4-bromophenyl)-1,5-dihydro-4H-pyrazolo[3,4-*d*]pyrimidin-4-one (3.00 g, 10.3 mmol, 1.0 eq) in dry DMF (25 mL) were added *tert*-butyl 1-oxa-6-azaspiro[2.5]octane-6-carboxylate (2.4 g, 11.4 mmol, 1.1 eq) and cesium carbonate (10.1 g, 31.0 mmol, 3.0 eq) at rt. The reaction was heated to 80 °C and stirred overnight. The mixture was then diluted with H_2O (20 mL) and extracted with EA (3x 50 mL). The combined organic layers were dried over $MgSO_4$, and the solvent was removed under reduced pressure. The resulting crude product was purified by flash column chromatography (PE/EA) which yielded the product *tert*-butyl 4-((1-(4-bromophenyl)-4-oxo-1,4-dihydro-5*H*-pyrazolo[3,4-*d*]pyrimidin-5-yl)methyl)-4-hydroxypiperidine-1-carboxylate (NK135) as an off-white solid (1.8 g, 3.66 mmol, 35 %).

HRMS (m/z) $[M+Na]^+$ calculated for $C_{22}H_{26}BrN_5NaO_4^+$: 526.1060, found: 526.1065.

^{1}H NMR (500 MHz, DMSO-*d*$_6$) δ (ppm): 8.39 (s, 1H), 8.36 (s, 1H), 8.07 (d, *J* = 8.9 Hz, 2H), 7.78 (d, *J* = 8.8 Hz, 2H), 4.90 (s, 1H), 4.03 (d, *J* = 2.0 Hz, 2H), 3.66 (d, *J* = 13.0 Hz, 2H), 3.05 (s, 2H), 1.48 (ddd, *J* = 13.4, 11.1, 4.6 Hz, 2H), 1.39 (s, 9H), 1.35 (s, 2H).

^{13}C NMR (126 MHz, DMSO-*d*$_6$) δ (ppm): 156.78, 153.81, 152.49, 151.29, 137.46, 136.66, 132.24, 123.08, 119.54, 106.96, 78.51, 68.98, 53.08, 34.20, 28.08.

Step 2:

NK135 (1.6 g, 3.2 mmol) was dissolved in TFA/DCM (20:80, 20 mL) and the reaction was stirred at rt for 4 h. After completion, the solvents were evaporated under reduced pressure which yielded 1-(4-bromophenyl)-5-((4-hydroxypiperidin-4-yl)methyl)-1,5-dihydro-4*H*-pyrazolo[3,4-*d*]pyrimidin-4-one (NK137) as an off-white solid (1.68 g, 3.2 mmol, quant. yield). The product was used for the next step without further purification.

HRMS (m/z) $[M+H]^+$ calculated for $C_{17}H_{19}BrN_5O_2^+$: 404.0717, found: 404.0709.

^{1}H NMR (500 MHz, DMSO-d_6) δ (ppm): 8.61 (s, 1H), 8.40 (d, *J* = 8.7 Hz, 2H), 8.32 (s, 1H), 8.06 (d, *J* = 8.4 Hz, 2H), 7.78 (d, *J* = 8.4 Hz, 2H), 5.30 (s, 1H), 4.09 (s, 2H), 3.21 – 3.13 (m, 2H), 3.02 (t, *J* = 11.5 Hz, 2H), 1.83 – 1.73 (m, 2H), 1.58 (d, *J* = 14.3 Hz, 2H).

^{13}C NMR (126 MHz, DMSO-d_6) δ (ppm): 156.81, 152.44, 151.28, 137.41, 136.67, 132.29, 123.11, 119.64, 106.94, 67.36, 52.80, 31.04.

Synthesis of (*R*)-1-(4-bromophenyl)-5-((4-hydroxy-1-(3-phenylbutanoyl)piperidin-4-yl)methyl)-1,5-dihydro-4*H*-pyrazolo[3,4-*d*]pyrimidin-4-one (NK195)

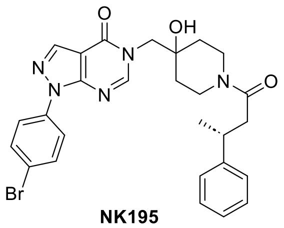

NK195

(*R*)-3-phenylbutanoic acid (587.9 mg, 3.58 mmol, 1.2 eq), DIPEA (1.54 g, 0.01 mol, 4.0 eq) and HATU (1.59 g, 4.18 mmol, 1.4 eq) in DMSO (10 mL) were added to a round bottom flask and were stirred at rt for 15 min. To this was added NK137 (1.31 g, 2.98 mmol, 1 eq). The reaction was stirred for 3 h at rt. The crude product was directly purified by reverse phase flash column chromatography (ACN/H_2O + 0.1 % TFA). The product (*R*)-1-(4-bromophenyl)-5-((4-hydroxy-1-(3-phenylbutanoyl)piperidin-4-yl)methyl)-1,5-dihydro-4*H*-pyrazolo[3,4-*d*]pyrimidin-4-one (NK195) was obtained as an off-white solid (1.43 g, 2.6 mmol, 87 % yield).

HRMS (m/z) $[M+Na]^+$ calculated for $C_{27}H_{28}BrN_5NaO_3^+$: 572.1268, found: 572.1266.

^{1}H NMR (600 MHz, DMSO-d_6) δ (ppm): 8.39 (s, 3H), 8.34 (d, *J* = 13.8 Hz, 3H), 8.06 (d, *J* = 8.9 Hz, 4H), 7.77 (d, *J* = 8.9 Hz, 4H), 7.26 – 7.24 (m, 10H), 7.15 (ddt, *J* = 8.4, 5.7, 2.0 Hz, 3H), 4.09 – 3.94 (m, 6H), 3.95 (d, *J* = 3.7 Hz, 3H), 3.69 – 3.62 (m, 22H), 3.25 – 3.14 (m, 5H), 2.92 – 2.83 (m, 3H), 2.66 – 2.52 (m, 4H), 1.57 – 1.30 (m, 10H), 1.20 (dd, *J* = 7.0, 1.3 Hz, 10H).

^{13}C NMR (151 MHz, DMSO-d_6) δ (ppm): 169.12 (“d”, *J* = 2.8 Hz), 156.75 (“d2, *J* = 8.1 Hz), 152.45 (“d”, *J* = 3.2 Hz), 151.92 – 150.78 (m), 146.64 (“d”, *J* = 16.5 Hz), 137.47, 136.66, 132.25, 128.21 (“d”, *J* = 5.5 Hz), 126.90 (“d”, *J* = 4.8 Hz), 125.95 (“d”, *J* = 6.2 Hz), 123.09, 119.56, 106.98, 69.11 (“d”, *J* = 7.2 Hz), 53.10 (“d”, *J* = 2.5 Hz), 41.00 (“d”, *J* = 17.5 Hz), 40.25 (“d”, *J* = 4.5 Hz), 36.92, 36.12 (“d”, *J* = 31.9 Hz), 34.88 (“d”, *J* = 16.5 Hz), 34.16 (“d”, *J* = 20.1 Hz), 21.99 (“d”, *J* = 30.6 Hz).

Synthesis of (*R*)-5-((4-hydroxy-1-(3-phenylbutanoyl)piperidin-4-yl)methyl)-1-(4-(piperazin-1-yl)phenyl)-1,5-dihydro-4*H*-pyrazolo[3,4-*d*]pyrimidin-4-one TFA salt (NK210)

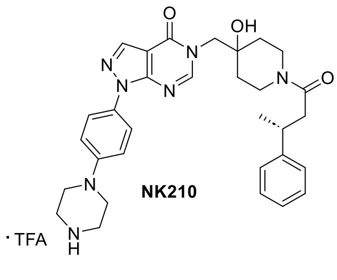

Step 1:

To a dry three necked round bottom flask under argon were added 1,4-dioxane (50 mL), NK195 (800 mg, 1.45 mmol, 1.0 eq), Boc-piperazine (379 mg, 2.03 mmol, 1.4 eq), cesium carbonate (1.42 g, 4.36 mmol, 3 eq) and RuPhosPd2G (112.9 mg, 0.15 mmol, 0.1 eq). The reaction was heated to 110 °C and stirred under argon atmosphere overnight. After completion, the mixture was filtered over Celite, and the solvents were removed under reduced pressure. The residue was resuspended with EA (100 mL) and washed with water and brine (3x 20 mL each). The organic layer was dried with $MgSO_4$, filtered and concentrated under reduced pressure. The resulting solids were then purified by reverse phase flash column chromatography (ACN/H_2O + 0.1 % TFA) which yielded the product *tert*-butyl (*R*)-

4-(4-(5-((4-hydroxy-1-(3-phenylbutanoyl)piperidin-4-yl)methyl)-4-oxo-4,5-dihydro-1*H*-pyrazolo[3,4-*d*]pyrimidin-1-yl)phenyl)piperazine-1-carboxylate (NK196) as an off-white powder.

Step 2:

NK196 (730.0 mg, 1.11 mmol) was dissolved in TFA/DCM (50:50) and stirred at rt for 3 h. After completion, the solvents were evaporated under reduced pressure. The resulting product (*R*)-5-((4-hydroxy-1-(3-phenylbutanoyl)piperidin-4-yl)methyl)-1-(4-(piperazin-1-yl)phenyl)-1,5-dihydro-4*H*-pyrazolo[3,4-*d*]pyrimidin-4-one (NK210) (723 mg, 1.08 mmol, 77 % yield over two steps) was obtained as an off-white powder and used for the next steps without further purification.

HRMS (m/z) $[M+H]^+$ calculated for $C_{31}H_{38}N_7O_3^+$: 556.3031, found: 556.3040.

^{1}H NMR (600 MHz, DMSO-d_6) δ (ppm): 8.93 (s, 2H), 8.33 – 8.27 (m, 2H), 7.86 (d, *J* = 9.1 Hz, 2H), 7.26 (dd, *J* = 8.8, 6.2 Hz, 4H), 7.20 – 7.13 (m, 3H), 4.11 – 3.90 (m, 3H), 3.65 (td, *J* = 10.8, 9.1, 4.0 Hz, 1H), 3.43 (dd, *J* = 6.6, 3.8 Hz, 4H), 3.30 – 3.25 (m, 4H), 3.17 (dt, *J* = 14.5, 7.4 Hz, 2H), 2.91 – 2.82 (m, 1H), 2.67 – 2.54 (m, 2H), 1.59 – 1.24 (m, 4H), 1.20 (dd, *J* = 7.0, 1.3 Hz, 3H).

^{13}C NMR (151 MHz, DMSO-d_6) δ (ppm): 169.12 (“d”, *J* = 2.8 Hz), 156.91 (“d”, *J* = 8.1 Hz), 151.99 (“d”, *J* = 3.3 Hz), 150.64 (“d”, *J* = 2.1 Hz), 148.88, 146.64 (“d”, *J* = 16.4 Hz), 135.72, 130.58, 128.21 (“d”, *J* = 5.0 Hz), 126.91 (“d”, *J* = 3.9 Hz), 125.95 (“d”, *J* = 5.6 Hz), 122.88, 116.13, 106.33, 69.12 (“d”, *J* = 7.2 Hz), 52.99, 45.37, 42.64, 41.02 (“d”, *J* = 17.0 Hz), 40.25 (“d”, *J* = 5.9 Hz), 36.94, 36.13 (“d”, *J* = 31.6 Hz), 34.91 (“d”, *J* = 17.4 Hz), 34.17 (“d”, *J* = 20.4 Hz), 21.99 (“d”, *J* = 30.1 Hz).

Synthesis of (*R*)-1-(4-([4,4'-bipiperidin]-1-yl)phenyl)-5-((4-hydroxy-1-(3-phenylbutanoyl)piperidin-4-yl)methyl)-1,5-dihydro-4*H*-pyrazolo[3,4-*d*]pyrimidin-4-one TFA salt (NK212)

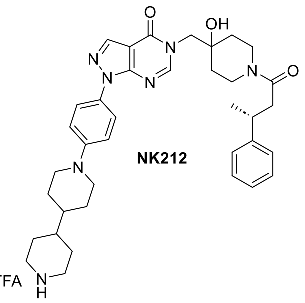

(*R*)-1-(4-([4,4'-Bipiperidin]-1-yl)phenyl)-5-((4-hydroxy-1-(3-phenylbutanoyl)piperidin-4-yl)methyl)-1,5-dihydro-4*H*-pyrazolo[3,4-*d*]pyrimidin-4-one TFA salt (NK212) was synthesized using the same method as described for NK210 (Supplementary Figure 9, exchanging *tert*-butyl piperazine-1-carboxylate for *tert*-butyl [4,4'-bipiperidine]-1-carboxylate).

HRMS (m/z) $[M+H]^+$ calculated for $C_{37}H_{48}N_7O_3^+$: 638.3813, found: 638.3806.

^{1}H NMR (600 MHz, DMSO-d_6) δ (ppm): 8.50 (d, *J* = 11.7 Hz, 1H), 8.30 (s, 1H), 8.28 (d, *J* = 14.1 Hz, 1H), 8.21 (d, *J* = 10.9 Hz, 1H), 7.84 (d, *J* = 8.7 Hz, 2H), 7.26 (dd, *J* = 8.6, 6.1 Hz, 4H), 7.19 (d, *J* = 8.5 Hz, 2H), 7.15 (ddd, *J* = 8.6, 6.4, 2.8 Hz, 1H), 4.08 – 3.98 (m, 2H), 3.98 – 3.90 (m, 1H), 3.83 – 3.78 (m, 2H), 3.70 – 3.61 (m, 1H), 3.30 (d, *J* = 12.3 Hz, 2H), 3.26 – 3.12 (m, 2H), 2.89 – 2.78 (m, 5H), 2.65 – 2.52 (m, 2H), 1.85 (d, *J* = 13.2 Hz, 2H), 1.80 (d, *J* = 10.0 Hz, 3H), 1.46 – 1.27 (m, 10H), 1.20 (dd, *J* = 7.0, 1.3 Hz, 3H).

^{13}C NMR (151 MHz, DMSO-d_6) δ (ppm): 169.11 (“d”, *J* = 2.8 Hz), 162.31, 156.90 (“d”, *J* = 8.1 Hz), 151.94 (“d”, *J* = 3.3 Hz), 150.62 (“d”, *J* = 2.0 Hz), 146.64 (“d”, *J* = 16.5 Hz), 135.69, 128.21 (“d”, *J* = 4.9 Hz), 126.90 (“d”, *J* = 4.0 Hz), 125.95 (“d”, *J* = 5.5 Hz), 122.89, 116.60 – 116.08 (m), 106.30, 69.12 (“d”, *J* = 7.2 Hz), 52.99, 49.51, 43.53, 41.01 (“d”, *J* = 16.7 Hz), 40.24 (“d”, *J* = 5.9 Hz), 37.75, 36.93, 36.12 (“d”, *J* = 31.3 Hz), 34.91 (“d”, *J* = 17.1 Hz), 34.17 (“d”, *J* = 20.2 Hz), 30.77, 28.00, 25.68, 21.99 (“d”, *J* = 30.1 Hz).

Synthesis of (*R*)-1-(4-(2,7-diazaspiro[3.5]nonan-7-yl)phenyl)-5-((4-hydroxy-1-(3-phenylbutanoyl)piperidin-4-yl)methyl)-1,5-dihydro-4*H*-pyrazolo[3,4-*d*]pyrimidin-4-one TFA salt (NK220)

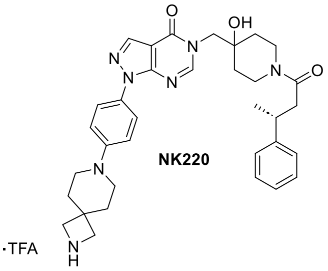

NK220

(*R*)-1-(4-(2,7-diazaspiro[3.5]nonan-7-yl)phenyl)-5-((4-hydroxy-1-(3-phenylbutanoyl)piperidin-4-yl)methyl)-1,5-dihydro-4*H*-pyrazolo[3,4-*d*]pyrimidin-4-one TFA salt (NK220) was synthesized using the same method as described for NK210 (Supplementary Figure 9, exchanging *tert*-butyl piperazine-1-carboxylate for *tert*-butyl 2,7-diazaspiro[3.5]nonane-2-carboxylate).

HRMS (m/z) $[M+H]^+$ calculated for $C_{34}H_{42}N_7O_3^+$: 596.3344, found: 596.3351.

^{1}H NMR (600 MHz, DMSO-d_6) δ (ppm): 8.71 (s, 2H), 8.29 (d, *J* = 0.8 Hz, 1H), 8.27 (d, *J* = 14.1 Hz, 1H), 7.79 (d, *J* = 9.1 Hz, 2H), 7.26 (dd, *J* = 8.3, 6.1 Hz, 4H), 7.17 – 7.10 (m, 3H), 4.07 – 3.92 (m, 3H), 3.78 – 3.74 (m, 4H), 3.66 (t, *J* = 13.1 Hz, 1H), 3.27 – 3.15 (m, 6H), 2.87 (ddt, *J* = 17.6, 10.1, 4.2 Hz, 1H), 2.64 – 2.52 (m, 2H), 1.88 – 1.85 (m, 4H), 1.56 – 1.23 (m, 4H), 1.20 (d, *J* = 6.7 Hz, 3H).

^{13}C NMR (151 MHz, DMSO-d_6) δ (ppm): 169.11 (“d”, *J* = 3.0 Hz), 156.91 (“d”, *J* = 8.1 Hz), 151.87 (“d”, *J* = 3.4 Hz), 150.51 (“d”, *J* = 2.1 Hz), 149.44, 146.64 (“d”, *J* = 16.3 Hz), 135.56, 129.47, 128.20 (“d2, *J* = 4.7 Hz), 126.90 (“d”, *J* = 3.8 Hz), 125.94 (“d”, *J* = 5.4 Hz), 122.88, 115.78, 106.23, 69.11 (“d”, *J* = 7.2 Hz), 54.79, 54.67, 52.97, 45.01, 41.00 (“d”, *J* = 16.9 Hz), 40.43, 40.23 (“d”, *J* = 6.5 Hz), 36.92, 36.22, 36.17, 36.01, 34.90 (“d”, *J* = 17.1 Hz), 34.16 (“d”, *J* = 20.0 Hz), 33.32, 21.99 (“d”, *J* = 29.8 Hz).

Synthesis of (*R*)-1-(4-(5-((4-hydroxy-1-(3-phenylbutanoyl)piperidin-4-yl)methyl)-4-oxo-4,5-dihydro-1*H*-pyrazolo[3,4-*d*]pyrimidin-1-yl)phenyl)piperidine-4-carboxylic acid (NK226)

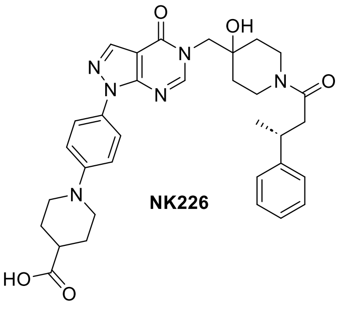
NK226

(*R*)-1-(4-(5-((4-hydroxy-1-(3-phenylbutanoyl)piperidin-4-yl)methyl)-4-oxo-4,5-dihydro-1*H*-pyrazolo[3,4-*d*]pyrimidin-1-yl)phenyl)piperidine-4-carboxylic acid (NK226) was synthesized using the same method as described for NK210 (Supplementary Figure 9, exchanging *tert*-butyl piperazine-1-carboxylate for *tert*-butyl piperidine-4-carboxylate) with an additional preparative reverse phase column chromatography (ACN/H_2O + 0.1 % TFA) purification after Step 2.

HRMS (m/z) $[M+H]^+$ calculated for $C_{33}H_{39}N_6O_5^+$: 599.2976, found: 599.2977.

^{1}H NMR (700 MHz, DMSO-d_6) δ (ppm): 8.30 (d, *J* = 1.0 Hz, 1H), 8.28 (d, *J* = 16.4 Hz, 1H), 7.84 (d, *J* = 8.5 Hz, 2H), 7.27 – 7.24 (m, 4H), 7.20 – 7.14 (m, 3H), 4.07 – 3.98 (m, 2H), 3.94 (d, *J* = 5.0 Hz, 1H), 3.72 (dt, *J* = 12.7, 3.9 Hz, 2H), 3.66 (t, *J* = 14.2 Hz, 1H), 3.25 – 3.14 (m, 2H), 2.96 – 2.84 (m, 3H), 2.64 – 2.57 (m, 2H), 1.95 (dd, *J* = 13.6, 3.7 Hz, 2H), 1.73 – 1.66 (m, 2H), 1.56 – 1.40 (m, 1H), 1.39 – 1.22 (m, 4H), 1.20 (dd, *J* = 6.9, 1.5 Hz, 3H).

^{13}C NMR (176 MHz, DMSO-d_6) δ (ppm): 175.74, 169.11 (“d”, *J* = 3.4 Hz), 156.91 (“d”, *J* = 9.5 Hz), 151.93 (“d”, *J* = 3.7 Hz), 150.60 (“d”, *J* = 1.6 Hz), 147.12, 146.64 (“d”, *J* = 19.1 Hz), 135.66, 128.20 (“d”, *J* = 5.9 Hz), 126.90 (“d”, *J* = 4.7 Hz), 125.94 (“d”, *J* = 6.9 Hz), 122.86, 116.44, 116.40, 106.29, 69.11 (“d”, *J* = 8.5 Hz), 53.00, 48.51, 41.00 (“d”, *J* = 19.5 Hz), 40.43, 40.23 (“d”, *J* = 6.9 Hz), 36.92, 36.12 (“d”, *J* = 36.4 Hz), 34.89 (“d”, *J* = 19.3 Hz), 34.17 (“d”, *J* = 23.3 Hz), 27.24, 21.98 (“d”, *J* = 34.6 Hz).

3.3 Synthesis of further functionalized USP7 inhibitors

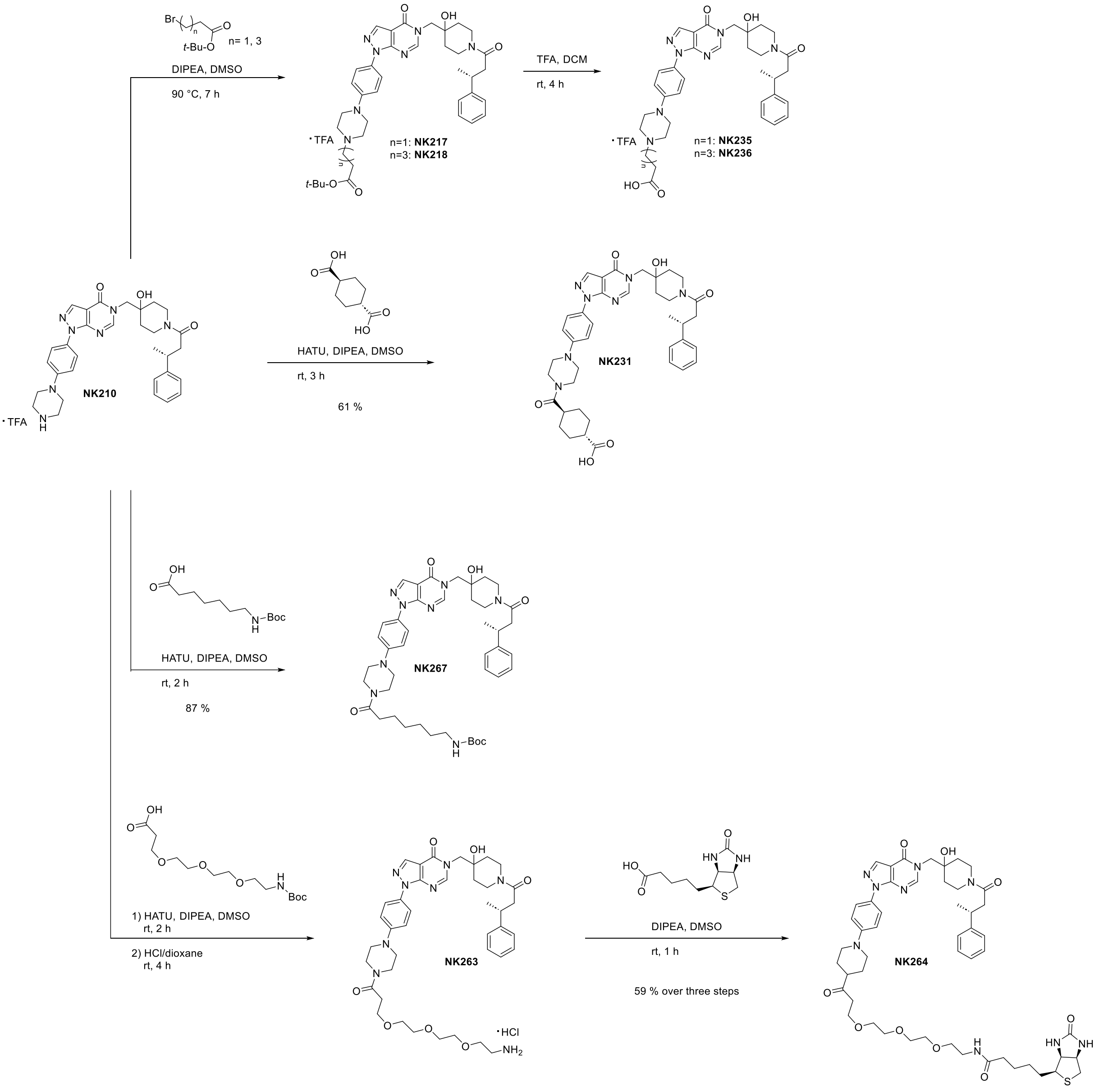

Supplementary Figure 10. Synthesis of further functionalized inhibitors NK235, NK236, NK231, NK267 and NK264.

Synthesis of (*R*)-3-(4-(4-(5-((4-hydroxy-1-(3-phenylbutanoyl)piperidin-4-yl)methyl)-4-oxo-4,5-dihydro-1*H*-pyrazolo[3,4-*d*]pyrimidin-1-yl)phenyl)piperazin-1-yl)propanoic acid TFA salt (NK235)

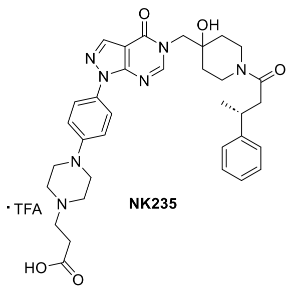

NK235

Step1:

NK210 (20.0 mg, 0.03 mmol, 1 eq), *tert*-butyl 3-bromopropanoate (12.5 mg, 0.06 mmol, 2 eq) and DIPEA (15.44 mg, 0.12 mmol, 4 eq) were dissolved in DMSO (1 mL) and stirred for 7 h at 90 °C. After cooling to rt, the crude mixture was directly purified by preparative reverse phase column chromatography (ACN/H_2O + 0.1 % TFA). The fractions containing the product *tert*-butyl (*R*)-3-(4-(4-(5-((4-hydroxy-1-(3-phenylbutanoyl)piperidin-4-yl)methyl)-4-oxo-4,5-dihydro-1*H*-pyrazolo [3,4-*d*]pyrimidin-1-yl)phenyl)piperazin-1-yl)propanoate (NK217) were combined and most of the solvents were evaporated under reduced pressure. The product was then directly used for the next step.

Step 2:

The combined fractions from the previous step that contained NK217 were diluted with DCM/TFA (50:50, 2 mL) and stirred at rt for 4 h. After completion, the solvents were evaporated under reduced pressure which yielded the crude product (*R*)-3-(4-(4-(5-((4-hydroxy-1-(3-phenylbutanoyl)piperidin-4-yl)methyl)-4-oxo-4,5-dihydro-1*H*-pyrazolo[3,4-*d*]pyrimidin-1-yl)phenyl)piperazin-1-yl)propanoic acid TFA salt (NK235) which was used directly for the next step.

Synthesis of (*R*)-5-(4-(4-(5-((4-hydroxy-1-(3-phenylbutanoyl)piperidin-4-yl)methyl)-4-oxo-4,5-dihydro-1*H*-pyrazolo[3,4-*d*]pyrimidin-1-yl)phenyl)piperazin-1-yl)pentanoic acid TFA salt (NK236)

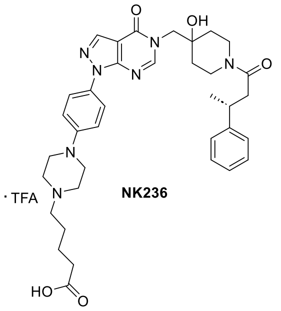

NK236

(*R*)-5-(4-(4-(5-((4-hydroxy-1-(3-phenylbutanoyl)piperidin-4-yl)methyl)-4-oxo-4,5-dihydro-1*H*-pyrazolo[3,4-*d*]pyrimidin-1-yl)phenyl)piperazin-1-yl)pentanoic acid TFA salt (NK236) was synthesized using the same method as described for NK235 (Supplementary Figure 10, exchanging *tert*-butyl 3-bromopropanoate for *tert*-butyl 5-bromopentanoate).

Synthesis of (1*r*,4*r*)-4-(4-(4-(5-((4-hydroxy-1-((*R*)-3-phenylbutanoyl)piperidin-4-yl)methyl)-4-oxo-4,5-dihydro-1*H*-pyrazolo[3,4-*d*]pyrimidin-1-yl)phenyl)piperazine-1-carbonyl)cyclohexane-1-carboxylic acid (NK231)

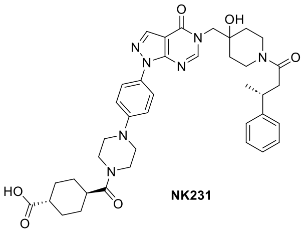

NK231

(1*r*,4*r*)-cyclohexane-1,4-dicarboxylic acid (19.8 mg, 0.11 mmol, 1.1 eq), HATU (51.7 mg, 0.14 mmol, 1.3 eq) and DIPEA (55.0 mg, 0.42 mmol, 4.0 eq) were dissolved in DMSO (1 mL) and stirred at rt for 15 min. To this was added NK210 (70.0 mg, 0.10 mmol, 1.0 eq) and the mixture was stirred at rt for 2 h. After completion, the crude product was directly purified by preparative reverse phase column chromatography (ACN/H_2O + 0.1 % TFA). This yielded the product (1*r*,4*r*)-4-(4-(4-(5-((4-hydroxy-1-((*R*)-3-phenylbutanoyl)piperidin-4-yl)methyl)-4-oxo-4,5-dihydro-1*H*-pyrazolo[3,4-*d*]pyrimidin-1-yl)phenyl)-piperazine-1-carbonyl)cyclohexane-1-carboxylic acid (NK231) (41.8 mg, 0.06 mmol, 56 % yield) as a white powder.

HRMS (m/z) $[M+H]^+$ calculated for $C_{39}H_{48}N_7O_6^+$: 710.3661, found: 710.3653.

^{1}H NMR (700 MHz, DMSO-*d*$_6$) δ (ppm): 8.29 (d, *J* = 1.1 Hz, 1H), 8.28 (d, *J* = 16.5 Hz, 1H), 7.83 (d, *J* = 9.1 Hz, 2H), 7.28 – 7.24 (m, 4H), 7.17 – 7.12 (m, 3H), 4.08 – 3.92 (m, 3H), 3.70 – 3.60 (m, 5H), 3.27 – 3.14 (m, 6H), 2.90 – 2.83 (m, 1H), 2.67 – 2.52 (m, 3H), 2.18 (ddt, *J* = 11.5, 7.1, 3.8 Hz, 1H), 1.97 – 1.89 (m, 2H), 1.76 – 1.71 (m, 2H), 1.45 – 1.22 (m, 8H), 1.20 (dd, *J* = 7.0, 1.5 Hz, 3H).

^{13}C NMR (176 MHz, DMSO-*d*$_6$) δ (ppm): 176.54, 173.18, 169.11, 156.91 (“d”, *J* = 9.5 Hz), 151.90 (“d”, *J* = 4.2 Hz), 150.56, 149.62, 146.63 (“d”, *J* = 19.2 Hz), 135.60, 129.98, 128.20 (“d”, *J* = 5.7 Hz), 126.89 (“d”, *J* = 4.6 Hz), 125.94 (“d”, *J* = 6.6 Hz), 122.84 (“d”, *J* = 1.8 Hz), 115.78, 106.26, 69.11 (“d”, *J* = 8.3 Hz), 52.99, 48.45 (“d”, *J* = 107.6 Hz), 44.46, 42.00, 41.00 (“d”, *J* = 19.5 Hz), 40.78, 40.23 (“d”, *J* = 7.1 Hz), 38.31, 36.92, 36.11 (“d”, *J* = 36.3 Hz), 34.89 (“d”, *J* = 19.6 Hz), 34.17 (“d”, *J* = 23.6 Hz), 27.98 (“d”, *J* = 69.4 Hz), 21.98 (“d”, *J* = 34.7 Hz).

Synthesis of *tert*-butyl (*R*)-(7-(4-(4-(5-((4-hydroxy-1-(3-phenylbutanoyl)piperidin-4-yl)methyl)-4-oxo-4,5-dihydro-1*H*-pyrazolo[3,4-*d*]pyrimidin-1-yl)phenyl)piperazin-1-yl)-7-oxoheptyl)carbamate (NK267)

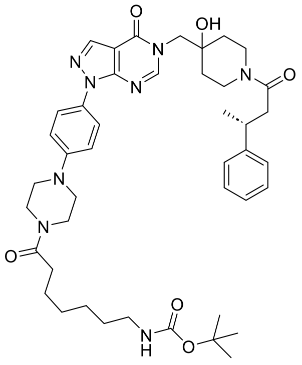

NK267

7-((*tert*-Butoxycarbonyl)amino)heptanoic acid (4.7 mg, 0.02 mmol, 1.3 eq), DIPEA (7.7 mg, 0.06 mmol, 4 eq) and HATU (7.4 mg, 0.02 mmol, 1.3 eq) were dissolved in DMSO (1 mL) and stirred for 15 min at rt. To this was added NK210 (10.0 mg, 0.01 mmol, 1 eq) and the mixture was stirred for 2 h at rt. After completion, the crude mixture was directly purified by preparative reverse phase column chromatography (ACN/H_2O + 0.1 % TFA) which yielded the product *tert*-butyl (*R*)-(7-(4-(4-(5-((4-hydroxy-1-(3-phenylbutanoyl)piperidin-4-yl)methyl)-4-oxo-4,5-dihydro-1*H*-pyrazolo[3,4-*d*]pyrimidin-1-yl)phenyl)piperazin-1-yl)-7-oxoheptyl)carbamate (NK267) (10.2 mg, 0.01 mmol, 87 % yield) as a white solid.

HRMS (m/z) $[M+H]^+$ calculated for $C_{43}H_{59}N_8O_6^+$: 783.4552, found: 783.4570.

^{1}H NMR (500 MHz, DMSO-*d*$_6$) δ (ppm): 8.29 (s, 1H), 8.27 (d, *J* = 11.7 Hz, 1H), 7.82 (d, *J* = 9.1 Hz, 2H), 7.29 – 7.24 (m, 4H), 7.19 – 7.09 (m, 3H), 6.76 (t, *J* = 5.8 Hz, 1H), 4.90 (s, 1H), 4.09 – 3.91 (m, 3H), 3.71 – 3.58 (m, 5H), 3.26 – 3.12 (m, 6H), 2.89 (p, *J* = 5.8, 5.4 Hz, 3H), 2.66 – 2.52 (m, 2H), 2.35 (t, *J* = 7.5 Hz, 2H), 1.50 (p, *J* = 7.7 Hz, 4H), 1.37 (s, 9H), 1.35 – 1.23 (m, 8H), 1.20 (d, *J* = 6.9 Hz, 3H).

^{13}C NMR (126 MHz, DMSO-*d*$_6$) δ (ppm): 171.19, 169.58, 157.39 ("d", *J* = 6.8 Hz), 156.05, 152.37 ("d", *J* = 2.4 Hz), 151.03 ("d", *J* = 1.3 Hz), 150.16, 147.11 ("d", *J* = 13.7 Hz), 136.07, 130.37, 128.68 ("d", *J* = 4.1 Hz), 127.37 ("d", *J* = 3.4 Hz), 126.42 ("d", *J* = 4.6 Hz), 123.32, 116.19, 106.73, 77.76, 69.59 ("d", *J* = 5.3 Hz), 53.47, 48.71 ("d", *J* = 51.8 Hz), 45.09, 41.47 ("d", *J* = 13.3 Hz), 41.16, 40.73, 37.39, 36.59 (""d, *J* = 25.8 Hz), 35.36 ("d", *J* = 14.6 Hz), 34.64 ("d", *J* = 15.2 Hz), 32.68, 29.87, 28.96, 28.75, 26.61, 25.23, 22.46 ("d", *J* = 24.7 Hz).

Synthesis of *N*-(2-(2-(2-(3-(4-(4-(5-((4-hydroxy-1-((*R*)-3-phenylbutanoyl)piperidin-4-yl)methyl)-4-oxo-4,5-dihydro-1*H*-pyrazolo[3,4-*d*]pyrimidin-1-yl)phenyl)piperazin-1-yl)-3-oxopropoxy)ethoxy)-ethoxy)ethyl)-5-((3*aS*,4*S*,6*aR*)-2-oxohexahydro-1*H*-thieno[3,4-*d*]imidazol-4-yl)pentanamide (NK264)

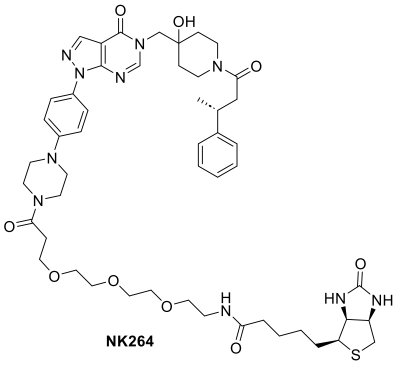

NK264

Step1:

2,2-dimethyl-4-oxo-3,8,11,14-tetraoxa-5-azaheptadecan-17-oic acid (8.6 mg, 0.03 mmol, 1.2 eq), DIPEA (11.6 mg, 0.09 mmol, 4 eq) and HATU (11.1 mg, 0.03 mmol, 1.3 eq) were dissolved in DMSO (1 mL) and stirred for 15 min at rt. To this was added NK210 (15.0 mg, 0.02 mmol, 1 eq) and the reaction was stirred for 2 h at rt. The crude mixture was directly purified by preparative reverse phase column chromatography (ACN/H_2O + 0.1 % TFA). The fractions containing the product *tert*-butyl (*R*)-(2-(2-(2-(3-(4-(4-(5-((4-hydroxy-1-(3-phenylbutanoyl)piperidin-4-yl)methyl)-4-oxo-4,5-dihydro-1*H*-pyrazolo[3,4-*d*]pyrimidin-1-yl)phenyl)piperazin-1-yl)-3-oxopropoxy)ethoxy)ethoxy)ethyl)carbamate (NK262) were combined and most of the solvents were evaporated under reduced pressure. The product was then directly used for the next step without further purification.

Step 2:

The combined fractions from the previous step that contained product NK262 were diluted with HCl/dioxane (4 M, 5 mL) and stirred at rt for 4 h. After completion, the solvents were evaporated under reduced pressure which yielded the crude product (*R*)-1-(4-(4-(3-(2-(2-(2-aminoethoxy)ethoxy)ethoxy)-propanoyl)piperazin-1-yl)phenyl)-5-((4-hydroxy-1-(3-phenylbutanoyl)piperidin-4-yl)methyl)-1,5-dihydro-4*H*-pyrazolo[3,4-*d*]pyrimidin-4-one HCl salt (NK263) which was directly used for the next step.

Step 3:

NK263 (15.0 mg, 0.02 mmol, 1 eq) was dissolved in DMSO (1 mL). To this was added DIPEA (12.2 mg, 0.09 mmol, 5 eq) and 2,5-dioxopyrrolidin-1-yl-2,5-dioxopyrrolidin-1-yl-5-((3*aS*,4*S*,6*aR*)-2-oxohexahydro-1*H*-thieno[3,4-*d*]imidazol-4-yl)pentanoate (NHS-biotin, 6.4 mg, 0.02 mmol, 1 eq) and the reaction was stirred for 1 h at rt. The crude mixture was directly purified by preparative reverse phase column chromatography (ACN/H_2O + 0.1 % TFA) which yielded *N*-(2-(2-(2-(3-(4-(4-(5-((4-hydroxy-1-((*R*)-3-phenylbutanoyl)piperidin-4-yl)methyl)-4-oxo-4,5-dihydro-1*H*-pyrazolo[3,4-*d*]pyrimidin-1-yl)phenyl)piperazin-1-yl)-3-oxopropoxy)ethoxy)ethoxy)ethyl)-5-((3*aS*,4*S*,6*aR*)-2-oxohexahydro-1*H*-thieno[3,4-*d*]imidazol-4-yl)pentanamide (NK264) (11.0 mg, 0.01 mmol, 59 % yield over three steps) as a white solid.

HRMS (m/z) $[M+Na]^+$ calculated for $C_{50}H_{68}N_{10}NaO_9S^+$: 1007.4784, found: 1007.4753.

^1H NMR (600 MHz, DMSO-d_6) δ (ppm): 8.31 – 8.26 (m, 2H), 7.85 – 7.80 (m, 3H), 7.26 (dd, *J* = 8.7, 6.2 Hz, 4H), 7.17 – 7.11 (m, 3H), 6.41 (s, 2H), 4.30 – 4.28 (m, 2H), 4.12 (dd, *J* = 7.7, 4.4 Hz, 2H), 4.07 – 4.03 (m, 3H), 3.98 (d, *J* = 13.9 Hz, 1H), 3.94 (d, *J* = 3.3 Hz, 2H), 3.67 – 3.63 (m, 6H), 3.51 (s, 8H), 3.38 (t, *J* = 5.9 Hz, 2H), 3.25 (t, *J* = 5.1 Hz, 2H), 3.18 (dt, *J* = 11.9, 5.9 Hz, 4H), 3.08 (ddd, *J* = 8.6, 6.2, 4.4 Hz, 1H), 2.89 – 2.84 (m, 1H), 2.81 (dd, *J* = 12.4, 5.1 Hz, 1H), 2.65 – 2.54 (m, 4H), 2.05 (t, *J* = 7.4 Hz, 2H), 1.62 – 1.25 (m, 10H), 1.20 (dd, *J* = 6.9, 1.2 Hz, 3H).

^{13}C NMR (151 MHz, DMSO-d_6) δ (ppm): 172.10, 169.10 (“d”, *J* = 2.9 Hz), 168.91, 162.68, 156.91 (“d”, *J* = 7.9 Hz), 151.91 (“d”, *J* = 3.3 Hz), 150.55 (“d”, *J* = 2.2 Hz), 149.58, 146.63 (“d”, *J* = 16.7 Hz), 135.60, 129.93, 128.20 (“d”, *J* = 5.0 Hz), 126.88, 125.94 (“d”, *J* = 5.6 Hz), 122.85, 115.73, 106.25, 69.78, 69.72, 69.69, 69.56, 69.16, 69.09, 66.80, 61.02, 59.18, 55.41, 53.00 – 52.95 (m), 48.43, 47.97, 44.68, 41.00 (“d”, *J* = 16.9 Hz), 40.69, 40.23 (“d”, *J* = 5.8 Hz), 38.44, 36.92, 36.11 (“d”, *J* = 31.2 Hz), 35.08, 34.89 (“d”, *J* = 16.9 Hz), 34.17 (“d”, *J* = 19.9 Hz), 32.78, 28.18, 28.03, 25.25, 21.98 (“d”, *J* = 29.9 Hz).

3.4 Synthesis of acid-functionalized VHL ligands

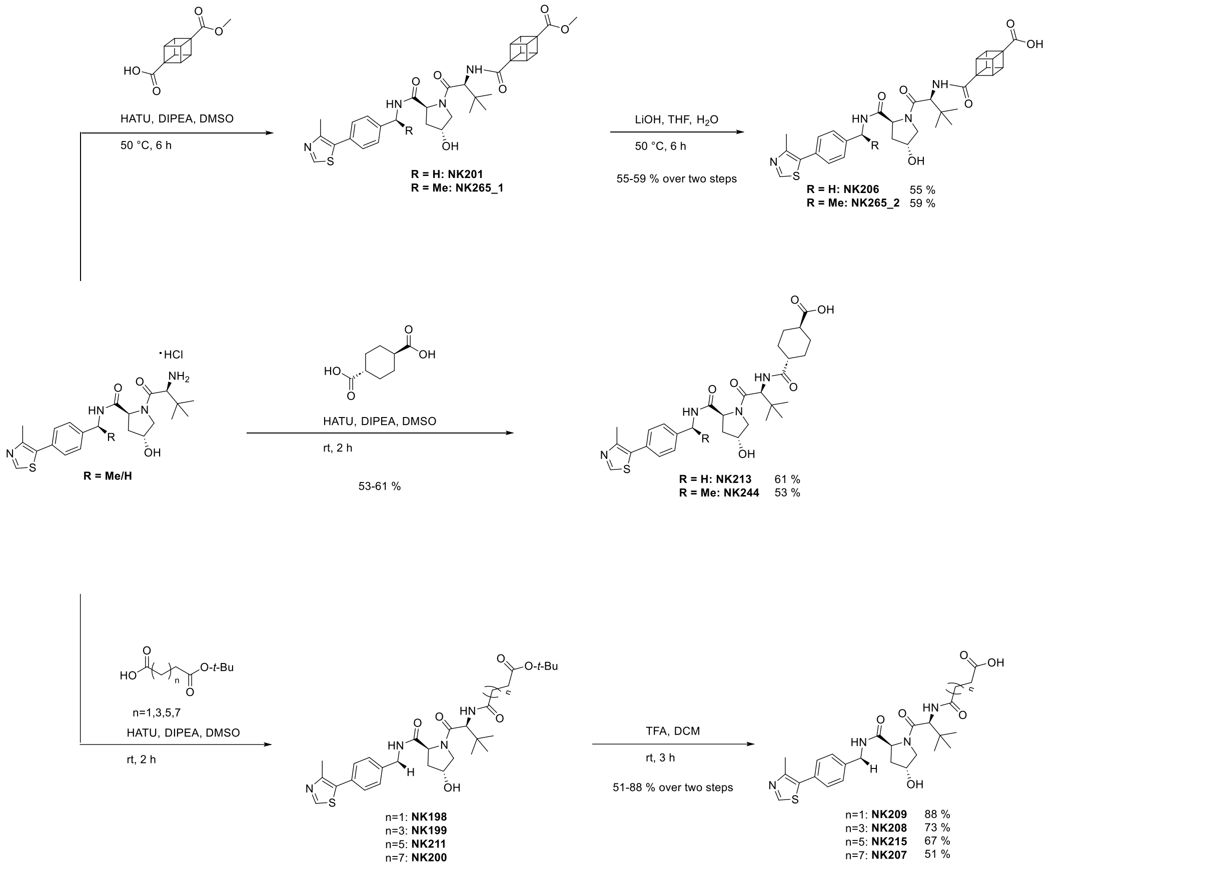

Supplementary Figure 11. Synthesis of acid-functionalized VHL-ligands NK206, NK265_2, NK213, NK244, NK209, NK208, NK215 and NK207.

Synthesis of (1*R*,2*R*,3*S*,8*S*)-4-(((*S*)-1-((2*S*,4*R*)-4-hydroxy-2-((4-(4-methylthiazol-5-yl)benzyl)carbamoyl)pyrrolidin-1-yl)-3,3-dimethyl-1-oxobutan-2-yl)carbamoyl)cubane-1-carboxylic acid (NK206)

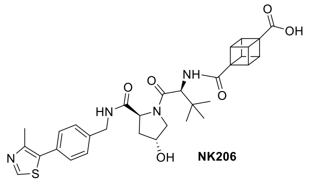

Step 1:

(2*r*,3*R*,4*s*,5*S*)-4-(Methoxycarbonyl)cubane-1-carboxylic acid (31.8 mg, 0.15 mmol, 1.2 eq), DIPEA (66.4 mg, 0.51 mmol, 4.0 eq) and HATU (63.5 mg, 0.17 mmol, 1.3 eq) were dissolved in DMSO (1 mL) and stirred for 15 min at 50 °C. To this was added (2*S*,4*R*)-1-((*S*)-2-amino-3,3-dimethylbutanoyl)-4-hydroxy-*N*-(4-(4-methylthiazol-5-yl)benzyl)pyrrolidine-2-carboxamide (60.0 mg, 0.13 mmol, 1.0 eq) and the reaction was stirred for 6 h at 50 °C. After completion, the crude product was cooled to rt and then directly purified by reverse phase flash column chromatography (ACN/H_2O + 0.1 % TFA). After evaporation of the solvents by reduced pressure, the product methyl (1*R*,2*R*,3*S*,8*S*)-4-(((*S*)-1-((2*S*,4*R*)-4-hydroxy-2-((4-(4-methylthiazol-5-yl)benzyl)carbamoyl)pyrrolidin-1-yl)-3,3-dimethyl-1-oxobutan-2-yl)carbamoyl)cubane-1-carboxylate (NK201) was directly used for the next step without further purification.

Step 2:

(1*R*,2*R*,3*S*,8*S*)-4-(((*S*)-1-((2*S*,4*R*)-4-Hydroxy-2-((4-(4-methylthiazol-5-yl)benzyl)carbamoyl)pyrrolidin-1-yl)-3,3-dimethyl-1-oxobutan-2-yl)carbamoyl)cubane-1-carboxylate (NK201) (43.7 mg, 0.07 mmol, 1 eq) was dissolved in THF (0.2 mL) to which was added LiOH in H_2O (2 M, 106 µL, 3 eq) and the reaction was stirred for 6 h at 50 °C. The reaction was quenched by the addition of 1 M HCl until a pH of 2-3 was achieved. After evaporation of the organic solvents by reduced pressure, the mixture was diluted with H_2O (10 mL) and extracted with EA (3 x 20 mL). The combined organic layers were washed with brine

(1x 20 mL), dried with $MgSO_4$, filtered and concentrated under reduced pressure which yielded the product (1*R*,2*R*,3*S*,8*S*)-4-(((*S*)-1-((2*S*,4*R*)-4-hydroxy-2-((4-(4-methylthiazol-5-yl)benzyl)carbamoyl)-pyrrolidin-1-yl)-3,3-dimethyl-1-oxobutan-2-yl)carbamoyl)cubane-1-carboxylic acid (NK206) (42.4 mg, 0.07 mmol, 55 % yield) as an off-white powder.

HRMS (m/z) $[M+Na]^+$ calculated for $C_{32}H_{36}N_4NaO_6S^+$: 627.2248, found: 627.2246.

^{1}H NMR (700 MHz, DMSO-*d*$_6$) δ (ppm): 9.01 (s, 1H), 8.59 (t, *J* = 6.1 Hz, 1H), 7.68 (d, *J* = 9.4 Hz, 1H), 7.44 – 7.35 (m, 4H), 4.59 (d, *J* = 9.3 Hz, 1H), 4.42 (dt, *J* = 15.7, 7.1 Hz, 2H), 4.34 (tt, *J* = 4.4, 2.4 Hz, 1H), 4.22 (dd, *J* = 15.8, 5.4 Hz, 1H), 4.11 – 4.06 (m, 6H), 3.67 – 3.61 (m, 2H), 2.44 (s, 3H), 2.04 (dddd, *J* = 10.3, 7.6, 4.1, 1.9 Hz, 1H), 1.89 (ddd, *J* = 12.9, 8.7, 4.6 Hz, 1H), 0.94 (s, 9H).

^{13}C NMR (176 MHz, DMSO-*d*$_6$) δ (ppm): 172.63, 171.92, 170.19, 169.45, 151.59, 147.53, 139.55, 131.26, 129.56, 128.66, 127.45, 68.84, 58.74, 57.02, 56.41, 56.15, 55.14, 46.38, 45.98, 41.66, 37.94, 35.50, 26.44, 15.86.

Synthesis of (1*R*,2*R*,3*S*,8*S*)-4-(((*S*)-1-((2*S*,4*R*)-4-hydroxy-2-(((*S*)-1-(4-(4-methylthiazol-5-yl)phenyl)ethyl)carbamoyl)pyrrolidin-1-yl)-3,3-dimethyl-1-oxobutan-2-yl)carbamoyl)cubane-1-carboxylic acid (NK265_2)

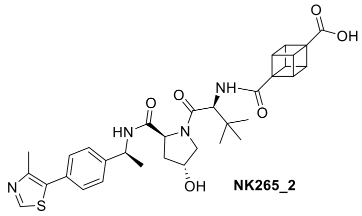

NK265_2

(1*R*,2*R*,3*S*,8*S*)-4-(((*S*)-1-((2*S*,4*R*)-4-hydroxy-2-(((*S*)-1-(4-(4-methylthiazol-5-yl)phenyl)ethyl)carbamoyl)pyrrolidin-1-yl)-3,3-dimethyl-1-oxobutan-2-yl)carbamoyl)cubane-1-carboxylic acid (NK265_2) was synthesized using the same procedure as described for NK206 (Supplementary Figure 11, exchanging (2*S*,4*R*)-1-((*S*)-2-amino-3,3-dimethylbutanoyl)-4-hydroxy-*N*-(4-(4-methylthiazol-5-yl)benzyl)pyrrolidine-2-carboxamide for (2*S*,4*R*)-1-((*S*)-2-amino-3,3-dimethylbutanoyl)-4-hydroxy-N-((*S*)-1-(4-(4-methylthiazol-5-yl)phenyl)ethyl)pyrrolidine-2-carboxamide).

HRMS (m/z) $[M+Na]^+$ calculated for $C_{33}H_{39}N_4O_6S^+$: 619.2585, found: 619.2586.

^{1}H NMR (500 MHz, DMSO-*d*$_6$) δ (ppm): 9.00 (s, 1H), 8.39 (d, *J* = 7.8 Hz, 1H), 7.57 (d, *J* = 9.3 Hz, 1H), 7.45 – 7.42 (m, 2H), 7.38 (d, *J* = 8.3 Hz, 2H), 4.98 – 4.89 (m, 1H), 4.60 – 4.53 (m, 1H), 4.43 (t, *J* = 8.1 Hz, 1H), 4.28 (dt, *J* = 4.2, 2.0 Hz, 1H), 4.14 – 4.02 (m, 6H), 3.59 (qd, *J* = 9.9, 9.1, 2.9 Hz, 2H), 2.45 (s, 3H), 2.04 – 1.94 (m, 1H), 1.79 (ddd, *J* = 13.0, 8.7, 4.6 Hz, 1H), 1.37 (d, *J* = 7.0 Hz, 3H), 0.94 (s, 9H).

^{13}C NMR (126 MHz, DMSO-*d*$_6$) δ (ppm): 172.64, 170.60, 170.11, 169.36, 151.57, 147.63, 144.65, 131.19, 129.64, 128.83, 126.41, 68.78, 58.65, 57.04, 56.37, 56.19, 55.18, 47.67, 46.35, 45.97, 37.71, 35.55, 26.50, 22.44, 15.93.

Synthesis of (1*S*,4*r*)-4-(((*S*)-1-((2*S*,4*R*)-4-hydroxy-2-((4-(4-methylthiazol-5-yl)benzyl)carbamoyl)-pyrrolidin-1-yl)-3,3-dimethyl-1-oxobutan-2-yl)carbamoyl)cyclohexane-1-carboxylic acid (NK213)

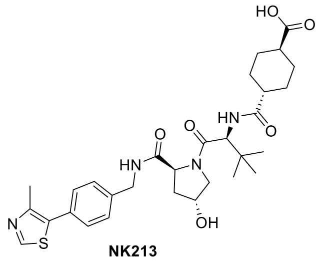

NK213

(1*r*,4*r*)-Cyclohexane-1,4-dicarboxylic acid (62.7 mg, 0.36 mmol, 1.7 eq), DIPEA (0.64 mmol, 83.0 mg, 4.0 eq) and HATU (97.7 mg, 0.26 mmol, 1.3 eq) were dissolved in DMSO (3 mL) and stirred for 15 min at rt. To this was added (2*S*,4*R*)-1-((*S*)-2-amino-3,3-dimethylbutanoyl)-4-hydroxy-N-(4-(4-methylthiazol-5-yl)benzyl)pyrrolidine-2-carboxamide (100.0 mg, 0.21 mmol, 1.0 eq) and the reaction was stirred for 2 h at rt. After completion, the crude product was directly purified by preparative reverse phase column chromatography (ACN/H_2O + 0.1 % TFA). The product (1*S*,4*r*)-4-(((*S*)-1-((2*S*,4*R*)-4-hydroxy-2-((4-(4-methylthiazol-5-yl)benzyl)carbamoyl)-pyrrolidin-1-yl)-3,3-dimethyl-1-oxobutan-2-yl)carbamoyl)cyclohexane-1-carboxylic acid (NK213) (76.2 mg, 0.13 mmol, 61 % yield) was obtained as a white powder.

HRMS (m/z) $[M+Na]^+$ calculated for $C_{30}H_{40}N_4NaO_6S^+$: 607.2561, found: 607.2552.

^{1}H NMR (600 MHz, DMSO-d_6) δ (ppm): 9.00 (s, 1H), 8.56 (t, *J* = 6.1 Hz, 1H), 7.74 (d, *J* = 9.3 Hz, 1H), 7.43 – 7.35 (m, 4H), 4.51 (d, *J* = 9.4 Hz, 1H), 4.46 – 4.41 (m, 2H), 4.37 – 4.32 (m, 2H), 4.22 (dd, *J* = 15.8, 5.4 Hz, 1H), 3.68 – 3.60 (m, 2H), 2.44 (s, 3H), 2.34 (tt, *J* = 11.8, 3.5 Hz, 1H), 2.13 (ddd, *J* = 11.8, 8.3, 3.6 Hz, 1H), 2.05 – 1.99 (m, 1H), 1.89 (dq, *J* = 12.8, 4.7 Hz, 4H), 1.79 (dq, *J* = 11.6, 2.3 Hz, 1H), 1.69 – 1.65 (m, 1H), 1.39 – 1.21 (m, 4H), 0.93 (s, 9H).

^{13}C NMR (151 MHz, DMSO-d_6) δ (ppm): 176.62, 174.73, 171.97, 169.66, 151.55, 147.61, 139.55, 131.24, 129.59, 128.65, 127.44, 68.87, 58.73, 56.35, 56.11, 42.48, 41.91, 41.65, 37.93, 35.39, 29.08, 28.10, 27.87, 27.80, 26.37, 15.90.

Synthesis of (1*S*,4*r*)-4-(((*S*)-1-((2*S*,4*R*)-4-hydroxy-2-(((*S*)-1-(4-(4-methylthiazol-5-yl)phenyl)ethyl)-carbamoyl)pyrrolidin-1-yl)-3,3-dimethyl-1-oxobutan-2-yl)carbamoyl)cyclohexane-1-carboxylic acid (NK244)

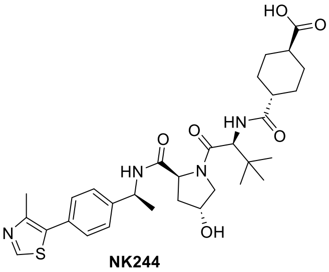

NK244

(1*S*,4*r*)-4-(((*S*)-1-((2*S*,4*R*)-4-Hydroxy-2-(((*S*)-1-(4-(4-methylthiazol-5-yl)phenyl)ethyl)-carbamoyl)pyrrolidin-1-yl)-3,3-dimethyl-1-oxobutan-2-yl)carbamoyl)cyclohexane-1-carboxylic acid (NK244) was synthesized as described for NK213 (Supplementary Figure 11, exchanging (2*S*,4*R*)-1-((*S*)-2-amino-3,3-dimethylbutanoyl)-4-hydroxy-*N*-(4-(4-methylthiazol-5-yl)benzyl)pyrrolidine-2-carboxamide for (2*S*,4*R*)-1-((*S*)-2-amino-3,3-dimethylbutanoyl)-4-hydroxy-N-((*S*)-1-(4-(4-methylthiazol-5-yl)phenyl)ethyl)pyrrolidine-2-carboxamide).

HRMS (m/z) $[M+H]^+$ calculated for $C_{31}H_{43}N_4O_6S^+$: 599.2898, found: 599.2896.

^{1}H NMR (700 MHz, DMSO-d_6) δ (ppm): 8.99 (s, 1H), 8.37 (d, *J* = 7.8 Hz, 1H), 7.68 (d, *J* = 9.3 Hz, 1H), 7.43 (d, *J* = 8.3 Hz, 2H), 7.38 (d, *J* = 8.3 Hz, 2H), 4.91 (p, *J* = 7.1 Hz, 1H), 4.48 (d, *J* = 9.3 Hz, 1H), 4.42 (t, *J* = 8.1 Hz, 1H), 4.28 (dt, *J* = 4.4, 2.0 Hz, 1H), 3.60 (s, 2H), 2.45 (s, 3H), 2.32 (td, *J* = 11.7, 5.9 Hz, 1H), 2.13 (ddt, *J* = 11.8, 8.3, 3.6 Hz, 1H), 2.02 – 1.99 (m, 1H), 1.91 – 1.87 (m, 2H), 1.78 (ddd, *J* = 12.6, 8.5, 4.4 Hz, 2H), 1.70 – 1.65 (m, 1H), 1.37 (d, *J* = 7.0 Hz, 4H), 1.35 – 1.25 (m, 4H), 0.93 (s, 9H).

^{13}C NMR (176 MHz, DMSO-d_6) δ (ppm): 176.76, 174.83, 170.78, 169.69, 151.66, 147.82, 144.81, 131.29, 129.79, 128.96, 126.52, 68.90, 58.71, 56.38, 56.33, 47.82, 42.64, 42.04, 37.84, 35.48, 29.14, 28.21, 28.00, 27.96, 26.56, 22.57, 16.08.

Synthesis of 4-(((*S*)-1-((2*S*,4*R*)-4-hydroxy-2-((4-(4-methylthiazol-5-yl)benzyl)carbamoyl)-pyrrolidin-1-yl)-3,3-dimethyl-1-oxobutan-2-yl)amino)-4-oxobutanoic acid (NK209)

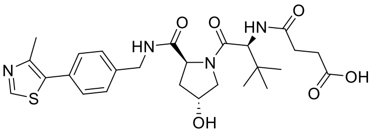

NK209

Step 1:

4-(*tert*-Butoxy)-4-oxobutanoic acid (26.9 mg, 0.15 mmol, 1.2 eq), DIPEA (66.4 mg, 0.51 mmol, 4.0 eq) and HATU (63.5 mg, 0.17 mmol, 1.3 eq) were dissolved in DMSO (1 mL) and stirred for 15 min at rt. To this was added (2*S*,4*R*)-1-((*S*)-2-amino-3,3-dimethylbutanoyl)-4-hydroxy-*N*-(4-(4-methylthiazol-5-yl)benzyl)pyrrolidine-2-carboxamide (60.0 mg, 0.13 mmol, 1 eq) and the reaction was stirred for 2 h at rt. After completion, the crude product was directly purified by preparative reverse phase column chromatography (ACN/H_2O + 0.1 % TFA). After evaporation of the solvents under reduced pressure, the product *tert*-butyl 4-(((*S*)-1-((2*S*,4*R*)-4-hydroxy-2-((4-(4-methylthiazol-5-yl)benzyl)carbamoyl)pyrrolidin-1-yl)-3,3-dimethyl-1-oxobutan-2-yl)amino)-4-oxobutanoate (NK198) was directly used for the next step without further purification.

Step 2:

NK198 was dissolved in TFA/DCM (50:50, 2 mL) and stirred for 3 h at rt. After completion, the solvents were evaporated under reduced pressure. This yielded the product 4-(((*S*)-1-((2*S*,4*R*)-4-hydroxy-2-((4-(4-methylthiazol-5-yl)benzyl)carbamoyl)-pyrrolidin-1-yl)-3,3-dimethyl-1-oxobutan-2-yl)amino)-4-oxobutanoic acid (NK209) (60.3 mg, 0.11 mmol, 88 % yield) as a white powder.

HRMS (m/z) $[M+H]^+$ calculated for $C_{26}H_{35}N_4O_6S^+$: 531.2272, found: 531.2268.

^{1}H NMR (600 MHz, DMSO-d_6) δ (ppm): 9.00 (s, 1H), 8.57 (t, *J* = 6.0 Hz, 1H), 7.93 (d, *J* = 9.3 Hz, 1H), 7.43 – 7.38 (m, 4H), 4.53 (d, *J* = 9.4 Hz, 1H), 4.48 – 4.41 (m, 2H), 4.35 (dt, *J* = 4.4, 2.2 Hz, 1H), 4.22 (dd, *J* = 15.8, 5.4 Hz, 1H), 3.67 (dd, *J* = 10.5, 4.2 Hz, 1H), 3.61 (dt, *J* = 10.9, 1.7 Hz, 1H), 2.46 – 2.33 (m, 7H), 2.05 – 2.00 (m, 1H), 1.90 (ddd, *J* = 13.0, 8.6, 4.6 Hz, 1H), 0.93 (s, 9H).

^{13}C NMR (151 MHz, DMSO-d_6) δ (ppm): 173.86, 171.96, 170.88, 169.58, 151.54, 147.62, 139.55, 131.24, 129.60, 128.65, 127.44, 68.88, 58.72, 56.45, 56.36, 41.66, 37.93, 35.38, 29.74, 29.25, 26.37, 15.90.

Synthesis of 6-(((*S*)-1-((2*S*,4*R*)-4-hydroxy-2-((4-(4-methylthiazol-5-yl)benzyl)carbamoyl)-pyrrolidin-1-yl)-3,3-dimethyl-1-oxobutan-2-yl)amino)-6-oxohexanoic acid (NK208)

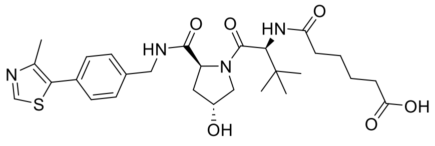

NK208

6-(((*S*)-1-((2*S*,4*R*)-4-hydroxy-2-((4-(4-methylthiazol-5-yl)benzyl)carbamoyl)-pyrrolidin-1-yl)-3,3-dimethyl-1-oxobutan-2-yl)amino)-6-oxohexanoic acid (NK208) was synthesized as described for NK209 (Supplementary Figure 11, exchanging 4-(*tert*-butoxy)-4-oxobutanoic acid for 6-(*tert*-butoxy)-6-oxohexanoic acid.

HRMS (m/z) $[M+H]^+$ calculated for $C_{28}H_{39}N_4O_6S^+$: 559.2585, found: 559.2579.

^{1}H NMR (700 MHz, DMSO-d_6) δ (ppm): 8.99 (s, 1H), 8.56 (t, *J* = 6.1 Hz, 1H), 7.86 (d, *J* = 9.4 Hz, 1H), 7.44 – 7.36 (m, 4H), 4.54 (d, *J* = 9.4 Hz, 1H), 4.45 – 4.40 (m, 2H), 4.35 (dt, *J* = 4.4, 2.0 Hz, 1H), 4.21 (dd, *J* = 15.8, 5.4 Hz, 1H), 3.69 – 3.61 (m, 2H), 2.44 (s, 3H), 2.26 (dt, *J* = 14.0, 7.2 Hz, 1H), 2.20 (td, *J* = 5.6, 4.5, 2.0 Hz, 2H), 2.14 – 2.10 (m, 1H), 2.03 (dddd, *J* = 12.3, 7.6, 2.8, 1.4 Hz, 1H), 1.90 (td, *J* = 8.4, 4.3 Hz, 1H), 1.54 – 1.44 (m, 4H), 0.93 (s, 9H).

^{13}C NMR (176 MHz, DMSO-d_6) δ (ppm): 174.39, 171.95, 171.87, 169.70, 151.51, 147.63, 139.54, 131.21, 129.60, 128.64, 127.43, 68.86, 58.69, 56.35, 56.31, 41.65, 37.93, 35.20, 34.56, 33.39, 26.38, 24.98, 24.16, 15.91.

Synthesis of 8-(((*S*)-1-((2*S*,4*R*)-4-hydroxy-2-((4-(4-methylthiazol-5-yl)benzyl)carbamoyl)-pyrrolidin-1-yl)-3,3-dimethyl-1-oxobutan-2-yl)amino)-8-oxooctanoic acid (NK215)

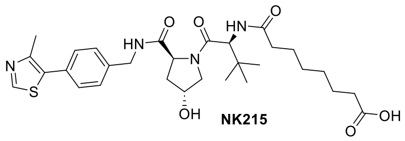

NK215

8-(((S)-1-((2*S*,4*R*)-4-hydroxy-2-((4-(4-methylthiazol-5-yl)benzyl)carbamoyl)-pyrrolidin-1-yl)-3,3-dimethyl-1-oxobutan-2-yl)amino)-8-oxooctanoic acid (NK215) was synthesized as described for NK209 (Supplementary Figure 11, exchanging 4-(*tert*-butoxy)-4-oxobutanoic acid for 8-(*tert*-butoxy)-8-oxooctanoic acid).

HRMS (m/z) $[M+H]^+$ calculated for $C_{30}H_{43}N_4O_6S^+$: 587.2898, found: 587.2892.

^{1}H NMR (700 MHz, DMSO-d_6) δ (ppm): 9.00 (s, 1H), 8.55 (t, *J* = 6.1 Hz, 1H), 7.84 (d, *J* = 9.4 Hz, 1H), 7.45 – 7.36 (m, 4H), 4.54 (d, *J* = 9.4 Hz, 1H), 4.45 – 4.41 (m, 2H), 4.35 (dt, *J* = 4.3, 2.0 Hz, 1H), 4.21 (dd, *J* = 15.8, 5.4 Hz, 1H), 3.73 – 3.61 (m, 2H), 2.44 (s, 3H), 2.25 (ddd, *J* = 14.1, 8.3, 7.0 Hz, 1H), 2.18 (td, *J* = 7.4, 5.1 Hz, 4H), 2.14 – 2.08 (m, 1H), 1.47 (d, *J* = 7.2 Hz, 4H), 1.25 (ddt, *J* = 11.5, 7.4, 4.2 Hz, 4H), 0.93 (s, 9H).

^{13}C NMR (176 MHz, DMSO-d_6) δ (ppm): 174.44, 172.06, 171.95, 169.71, 151.51, 147.62, 139.54, 131.21, 129.59, 128.64, 127.42, 68.85, 58.68, 56.33, 56.27, 41.64, 37.93, 35.20, 34.83, 33.57, 28.38, 28.22, 26.38, 25.30, 24.32, 15.90.

Synthesis of 10-(((*S*)-1-((2*S*,4*R*)-4-hydroxy-2-((4-(4-methylthiazol-5-yl)benzyl)carbamoyl)-pyrrolidin-1-yl)-3,3-dimethyl-1-oxobutan-2-yl)amino)-10-oxodecanoic acid (NK207)

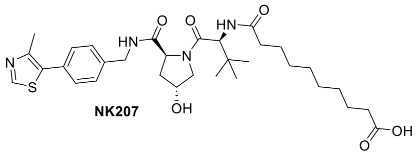

10-(((*S*)-1-((2*S*,4*R*)-4-hydroxy-2-((4-(4-methylthiazol-5-yl)benzyl)carbamoyl)-pyrrolidin-1-yl)-3,3-dimethyl-1-oxobutan-2-yl)amino)-10-oxodecanoic acid (NK207) was synthesized as described for NK209 (Supplementary Figure 11, exchanging 4-(*tert*-butoxy)-4-oxobutanoic acid for 10-(*tert*-butoxy)-10-oxodecanoic acid.

HRMS (m/z) $[M+H]^+$ calculated for $C_{32}H_{47}N_4O_6S^+$: 615.3211, found: 615.3201.

^{1}H NMR (600 MHz, DMSO-d_6) δ (ppm): 8.99 (s, 1H), 8.56 (t, *J* = 6.1 Hz, 1H), 7.83 (d, *J* = 9.3 Hz, 1H), 7.43 – 7.34 (m, 4H), 4.54 (d, *J* = 9.4 Hz, 1H), 4.45 – 4.41 (m, 2H), 4.35 (dt, *J* = 4.4, 2.1 Hz, 1H), 4.21 (dd, *J* = 15.8, 5.3 Hz, 1H), 3.68 – 3.62 (m, 2H), 2.44 (s, 3H), 2.25 (ddd, *J* = 14.1, 8.2, 7.1 Hz, 1H), 2.18 (t, *J* = 7.4 Hz, 2H), 2.10 (ddd, *J* = 14.2, 8.2, 6.2 Hz, 1H), 2.03 (dddd, *J* = 11.8, 7.6, 3.0, 1.7 Hz, 1H), 1.90 (ddd, *J* = 12.9, 8.6, 4.6 Hz, 1H), 1.47 (dq, *J* = 14.7, 7.3 Hz, 4H), 1.26 – 1.21 (m, 10H), 0.93 (s, 9H).

^{13}C NMR (151 MHz, DMSO-d_6) δ (ppm): 174.49, 172.10, 171.95, 169.72, 162.31, 147.65, 139.54, 131.21, 129.60, 128.64, 127.43, 68.86, 58.69, 56.34, 56.27, 41.64, 40.43, 37.95, 35.21, 34.85, 33.67, 28.68, 28.63, 28.54, 26.38, 25.42, 24.48, 15.92.

3.5 Synthesis of an amine-functionalized VHL ligand

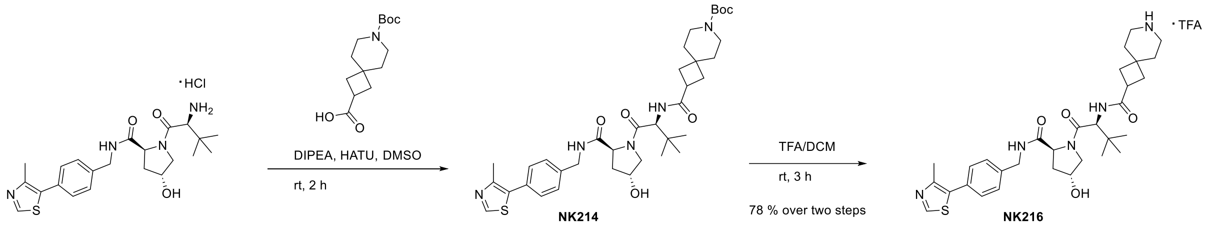

Supplementary Figure 12. Synthesis of amine-functionalized VHL ligand NK216.

Synthesis of *N*-((*S*)-1-((2*S*,4*R*)-4-hydroxy-2-((4-(4-methylthiazol-5-yl)benzyl)carbamoyl)pyrrolidin-1-yl)-3,3-dimethyl-1-oxobutan-2-yl)-7-azaspiro[3.5]nonane-2-carboxamide TFA salt (NK216)

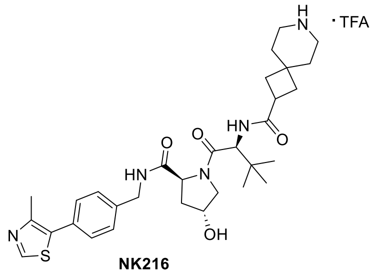

NK216

Step 1:

7-(*tert*-Butoxycarbonyl)-7-azaspiro[3.5]nonane-2-carboxylic acid (48.4 mg, 0.18 mmol, 1.2 eq), DIPEA (0.60 mmol, 77.486 mg, 4.0 eq) and HATU (74.09 mg, 0.19 mmol, 1.3 eq) were dissolved in DMSO (1 mL) and stirred for 15 min at rt. To this was added (2*S*,4*R*)-1-((*S*)-2-amino-3,3-dimethylbutanoyl)-4-hydroxy-*N*-(4-(4-methylthiazol-5-yl)benzyl)pyrrolidine-2-carboxamide (70.0 mg, 0.15 mmol, 1.0 eq) and the reaction was stirred at rt for 2 h. After completion, the crude product was directly purified by preparative reverse phase column chromatography (ACN/H_2O + 0.1 % TFA). The product *tert*-butyl 2-(((*S*)-1-((2*S*,4*R*)-4-hydroxy-2-((4-(4-methylthiazol-5-yl)benzyl)carbamoyl)pyrrolidin-1-yl)-3,3-dimethyl-1-oxobutan-2-yl)carbamoyl)-7-azaspiro[3.5]nonane-7-carboxylate (NK214) was directly used for the next step.

Step 2:

NK214 was dissolved in TFA/DCM (50:50, 2 mL) and stirred for 3 h at rt. After completion, the solvents were evaporated under reduced pressure. This yielded the product *N*-((*S*)-1-((2*S*,4*R*)-4-hydroxy-2-((4-(4-methylthiazol-5-yl)benzyl)carbamoyl)pyrrolidin-1-yl)-3,3-dimethyl-1-oxobutan-2-yl)-7-azaspiro[3.5]nonane-2-carboxamide TFA salt (NK216) (76.9 mg, 0.11 mmol, 75 % yield) as a white powder.

HRMS (m/z) $[M+H]^+$ calculated for $C_{31}H_{44}N_5O_4S^+$: 582.3109, found: 582.3096.

^{1}H NMR (700 MHz, DMSO-*d*$_6$) δ (ppm): 8.99 (s, 1H), 8.55 (t, *J* = 6.1 Hz, 1H), 8.29 (s, 2H), 7.74 (d, *J* = 9.3 Hz, 1H), 7.43 – 7.37 (m, 4H), 4.53 (d, *J* = 9.3 Hz, 1H), 4.42 (dt, *J* = 16.1, 7.5 Hz, 2H), 4.35 (dt, *J* = 4.4, 2.0 Hz, 1H), 4.21 (dd, *J* = 15.8, 5.4 Hz, 1H), 3.69 – 3.62 (m, 2H), 3.25 – 3.15 (m, 1H), 3.02 – 2.98 (m, 2H), 2.91 (s, 2H), 2.44 (s, 3H), 2.05 – 1.94 (m, 3H), 1.92 – 1.82 (m, 3H), 1.70 (t, *J* = 5.9 Hz, 2H), 1.64 (t, *J* = 5.9 Hz, 2H), 0.92 (s, 9H).

^{13}C NMR (176 MHz, DMSO-*d*$_6$) δ (ppm): 173.90, 171.91, 169.56, 151.49, 147.68, 139.51, 131.18, 129.62, 128.64, 127.42, 68.87, 58.70, 56.45, 56.36, 41.64, 40.55, 40.43, 40.30, 37.98, 35.38, 34.58, 34.08, 32.70, 32.35, 31.34, 26.33, 15.92.

3.6 Synthesis of PROTACs from acid functionalized USP7 ligands

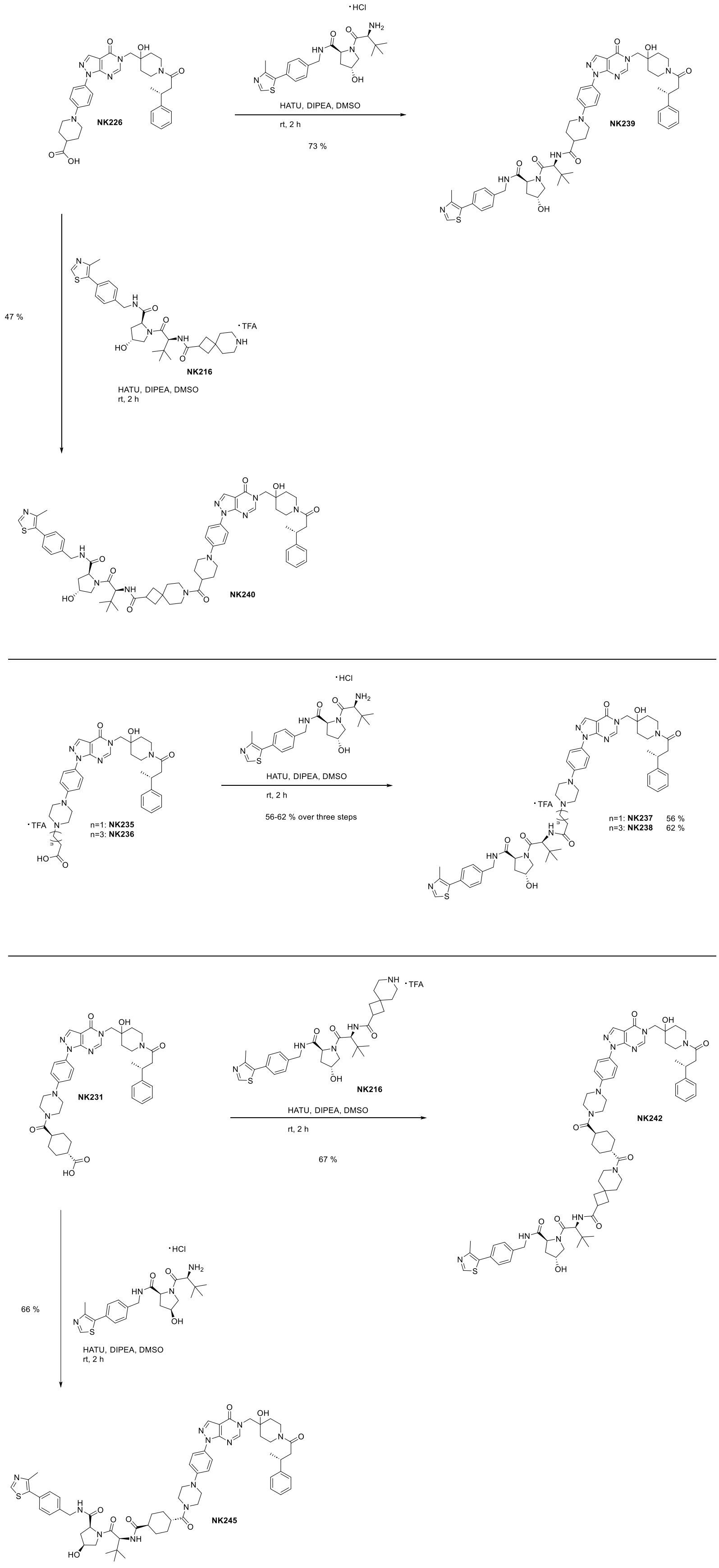

Supplementary Figure 13. Synthesis of PROTACS NK239, NK240, NK237, NK238, NK242 and of control NK245.

Synthesis of 1-(4-(5-((4-hydroxy-1-((*R*)-3-phenylbutanoyl)piperidin-4-yl)methyl)-4-oxo-4,5-dihydro-1*H*-pyrazolo[3,4-*d*]pyrimidin-1-yl)phenyl)-*N*-((*S*)-1-((2*S*,4*R*)-4-hydroxy-2-((4-(4-methylthiazol-5-yl)benzyl)carbamoyl)pyrrolidin-1-yl)-3,3-dimethyl-1-oxobutan-2-yl)piperidine-4-carboxamide (NK239)

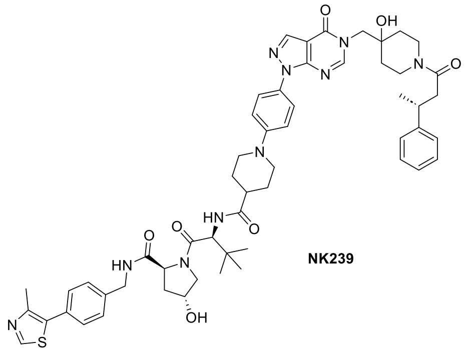

NK239

NK226 (9.9 mg, 0.02 mmol, 1.1 eq), HATU (7.4 mg, 0.02 mmol, 1.3 eq) and DIPEA (9.7 mg, 0,07 mmol, 5 eq) were dissolved in DMSO (1 mL) and stirred at rt for 15 min. To this was added (2*S*,4*R*)-1-((*S*)-2-amino-3,3-dimethyl-butanoyl)-4-hydroxy-*N*-(4-(4-methylthiazol-5-yl)benzyl)-pyrrolidine-2-carboxamide (7.0 mg, 0.01 mmol, 1 eq) and the mixture was stirred at rt for 2 h. After completion, the crude product was purified by preparative reverse phase column chromatography (ACN/H_2O + 0.1 % TFA). After evaporation of the solvents under reduced pressure, the product 1-(4-(5-((4-hydroxy-1-((*R*)-3-phenylbutanoyl)-piperidin-4-yl)methyl)-4-oxo-4,5-dihydro-1*H*-pyrazolo[3,4-*d*]pyrimidin-1-yl)phenyl)-*N*-((*S*)-1-((2*S*,4*R*)-4-hydroxy-2-((4-(4-methylthiazol-5-yl)benzyl)carbamoyl)pyrrolidin-1-yl)-3,3-dimethyl-1-oxobutan-2-yl)piperidine-4-carboxamide (NK239) (11.1 mg, 0.01 mmol, 73 % yield) was obtained as a white powder.

HRMS (m/z) $[M+2H]^{2+}$ calculated for $C_{55}H_{68}N_{10}O_7S^{2+}$: 506.2491, found: 506.2477.

^{1}H NMR (600 MHz, DMSO-*d*$_6$) δ (ppm): 9.00 (s, 1H), 8.57 (t, *J* = 6.1 Hz, 1H), 8.30 (s, 1H), 8.28 (d, *J* = 14.1 Hz, 1H), 7.93 (d, *J* = 9.3 Hz, 1H), 7.83 (d, *J* = 8.6 Hz, 2H), 7.44 – 7.38 (m, 5H), 7.26 (dd, *J* = 8.8, 6.2 Hz, 5H), 7.21 – 7.14 (m, 3H), 4.55 (d, *J* = 9.4 Hz, 1H), 4.46 – 4.41 (m, 2H), 4.35 (dq, *J* = 4.4, 2.2 Hz, 1H), 4.22 (dd, *J* = 15.8, 5.5 Hz, 1H), 4.07 – 3.93 (m, 3H), 3.83 – 3.79 (m, 2H), 3.69 – 3.62 (m, 3H), 3.25 – 3.13 (m, 2H), 2.90 – 2.81 (m, 3H), 2.67 – 2.57 (m, 2H), 2.45 (s, 3H), 2.06 – 2.02 (m, 1H), 1.93 – 1.84 (m, 2H), 1.76 – 1.67 (m, 3H), 1.56 – 1.23 (m, 5H), 1.20 (dd, *J* = 7.0, 1.2 Hz, 3H), 0.95 (s, 9H).

^{13}C NMR (151 MHz, DMSO-*d*$_6$) δ (ppm): 173.96, 171.96, 169.60, 169.11 (“d”, *J* = 2.9 Hz), 156.91 (“d”, *J* = 8.1 Hz), 151.93 (“d”), 151.51, 150.60, 147.66, 146.64 (“d”, *J* = 16.6 Hz), 139.53, 135.66, 131.20, 129.62, 128.87 (“d”, *J* = 3.4 Hz), 128.65, 128.21 (“d”, *J* = 5.0 Hz), 128.04, 127.44, 126.90 (“d”, *J* = 3.9 Hz), 125.94 (“d”, *J* = 6.1 Hz), 122.87, 120.74, 106.29, 69.12 (“d”, *J* = 7.3 Hz), 68.89, 58.74, 56.41, 56.22, 53.01, 41.65, 41.00 (“d”, *J* = 16.8 Hz), 40.52, 40.23 (“d”, *J* = 5.9 Hz), 37.95, 36.92, 36.12 (“d”, *J* = 31.2 Hz), 35.44, 34.90 (“d”, *J* = 17.1 Hz), 34.18 (“d”, *J* = 20.0 Hz), 28.57, 27.19, 26.38, 21.99 (“d”, *J* = 29.9 Hz), 15.92.

Synthesis of 7-(1-(4-(5-((4-hydroxy-1-((*R*)-3-phenylbutanoyl)piperidin-4-yl)methyl)-4-oxo-4,5-dihydro-1*H*-pyrazolo[3,4-*d*]pyrimidin-1-yl)phenyl)piperidine-4-carbonyl)-*N*-((*S*)-1-((2*S*,4*R*)-4-hydroxy-2-((4-(4-methylthiazol-5-yl)benzyl)carbamoyl)pyrrolidin-1-yl)-3,3-dimethyl-1-oxobutan-2-yl)-7-azaspiro[3.5]nonane-2-carboxamide (NK240)

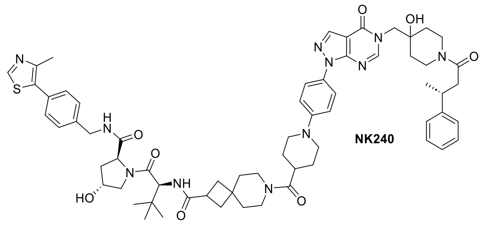

NK240

7-(1-(4-(5-((4-hydroxy-1-((*R*)-3-phenylbutanoyl)-piperidin-4-yl)methyl)-4-oxo-4,5-dihydro-1*H*-pyrazolo[3,4-*d*]pyrimidin-1-yl)phenyl)piperidine-4-carbonyl)-*N*-((*S*)-1-((2*S*,4*R*)-4-hydroxy-2-((4-(4-methylthiazol-5-yl)benzyl)carbamoyl)pyrrolidin-1-yl)-3,3-dimethyl-1-oxobutan-2-yl)-7-azaspiro[3.5]-nonane-2-carboxamide (NK240) was synthesized as described for NK239 (Supplementary Figure 13, exchanging (*N*-((*S*)-1-((2*S*,4*R*)-4-hydroxy-2-((4-(4-methylthiazol-5-yl)benzyl)carbamoyl)pyrrolidin-1-yl)-3,3-dimethyl-1-oxobutan-2-yl)-7-azaspiro[3.5]nonane-2-carboxamide (NK216) for (2*S*,4*R*)-1-((*S*)-2-amino-3,3-dimethyl-butanoyl)-4-hydroxy-*N*-(4-(4-methylthiazol-5-yl)benzyl)-pyrrolidine-2-carboxamide).

HRMS (m/z) $[M+2H]^{2+}$ calculated for $C_{64}H_{81}N_{11}O_8S^{2+}$: 581.7990, found: 581.7987.

H NMR (600 MHz, DMSO-*d*$_6$) δ (ppm): 8.99 (s, 1H), 8.56 (t, *J* = 6.1 Hz, 1H), 8.30 (s, 1H), 8.28 (d, *J* = 14.2 Hz, 1H), 7.81 (d, *J* = 8.4 Hz, 2H), 7.71 (t, *J* = 10.3 Hz, 1H), 7.43 – 7.36 (m, 4H), 7.26 (dd, *J* = 8.7,

6.2 Hz, 4H), 7.18 – 7.13 (m, 3H), 4.54 (d, *J* = 9.3 Hz, 1H), 4.43 (td, *J* = 8.8, 4.7 Hz, 2H), 4.36 (d, *J* = 3.8 Hz, 1H), 4.23 (d, *J* = 5.5 Hz, 1H), 4.20 (d, *J* = 5.4 Hz, 1H), 4.04 (d, *J* = 6.8 Hz, 1H), 3.99 (s, 1H), 3.94 (d, *J* = 3.5 Hz, 1H), 3.81 – 3.77 (m, 2H), 3.67 – 3.65 (m, 2H), 3.48 – 3.32 (m, 4H), 3.20 (dt, *J* = 34.7, 8.4 Hz, 3H), 2.87 (q, *J* = 17.4, 16.3 Hz, 4H), 2.64 – 2.54 (m, 2H), 2.44 (s, 6H), 2.06 – 1.81 (m, 6H), 1.70 (s, 4H), 1.59 – 1.31 (m, 8H), 1.20 (dd, *J* = 6.9, 1.2 Hz, 3H), 0.93 (s, 9H).

^{13}C NMR (151 MHz, DMSO-*d*$_6$) δ (ppm): 174.55, 172.61, 172.42, 170.10, 169.58 (“d”, *J* = 2.9 Hz), 157.39 (“d”, *J* = 8.1 Hz), 152.37, 151.96, 151.03, 148.17, 147.11 (“d”, *J* = 16.6 Hz), 139.99, 136.07, 131.65, 130.10, 129.12, 128.68 (“d”, *J* = 4.9 Hz), 127.90, 127.38 (“d”, *J* = 3.9 Hz), 126.42 (“d”, *J* = 5.9 Hz), 123.38, 116.46, 116.37, 106.73, 69.59 (“d”, *J* = 7.1 Hz), 69.36, 59.18, 56.92, 56.85, 53.48, 48.78, 42.11, 41.48 (“d”, *J* = 17.1 Hz), 40.71 (“d”, *J* = 5.7 Hz), 38.78, 38.43, 37.62, 37.40, 37.32, 36.59 (“d”, *J* = 31.1 Hz), 35.83, 35.39 (“d”, *J* = 11.6 Hz), 35.31, 34.65 (“d”, *J* = 19.4 Hz), 34.51, 34.05, 32.17, 28.21, 26.82, 22.46 (“d”, *J* = 29.8 Hz), 16.41.

Synthesis of (2*S*,4*R*)-4-hydroxy-1-((*S*)-2-(3-(4-(4-(5-((4-hydroxy-1-((*R*)-3-phenylbutanoyl)-piperidin-4-yl)methyl)-4-oxo-4,5-dihydro-1*H*-pyrazolo[3,4-d]pyrimidin-1-yl)phenyl)piperazin-1-yl)propanamido)-3,3-dimethylbutanoyl)-*N*-(4-(4-methylthiazol-5-yl)benzyl)pyrrolidine-2-carboxamide TFA salt (NK237)

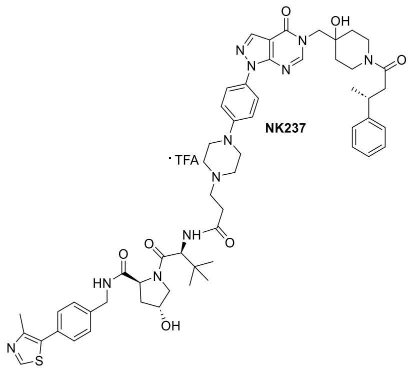

(2*S*,4*R*)-4-hydroxy-1-((*S*)-2-(3-(4-(4-(5-((4-hydroxy-1-((*R*)-3-phenylbutanoyl)-piperidin-4-yl)methyl)-4-oxo-4,5-di-hydro-1*H*-pyrazolo[3,4-d]pyrimidin-1-yl)phenyl)-piperazin-1-yl)propanamido)-3,3-dimethylbutanoyl)-*N*-(4-(4-methyl-thiazol-5-yl)benzyl)pyrrolidine-2-carboxamide TFA salt (NK237) was synthesized as described for NK239 (Supplementary Figure 13, exchanging (*R*)-1-(4-(5-((4-hydroxy-1-(3-phenylbutanoyl)piperidin-4-yl)methyl)-4-oxo-4,5-dihydro-1*H*-pyrazolo[3,4-*d*]pyrimidin-1-yl)phenyl)piperidine-4-carboxylic (NK226) acid for (*R*)-3-(4-(4-(5-((4-hydroxy-1-(3-phenylbutanoyl)piperidin-4-yl)methyl)-4-oxo-4,5-dihydro-1*H*-pyrazolo[3,4-*d*]pyrimidin-1-yl)phenyl)piperazin-1-yl)propanoic acid (NK235).

HRMS (m/z) $[M+2H]^{2+}$ calculated for $C_{56}H_{71}N_{11}O_7S^{2+}$: 520.7624, found: 520.7630.

^{1}H NMR (600 MHz, DMSO-*d*$_6$) δ (ppm): 9.62 (s, 1H), 9.00 (s, 1H), 8.59 (t, *J* = 6.1 Hz, 1H), 8.37 – 8.26 (m, 3H), 7.89 – 7.84 (m, 2H), 7.46 – 7.37 (m, 4H), 7.27 (dd, *J* = 8.4, 6.1 Hz, 4H), 7.21 – 7.14 (m, 3H), 4.59 (d, *J* = 9.3 Hz, 1H), 4.47 (d, *J* = 6.7 Hz, 1H), 4.45 (s, 1H), 4.38 (dt, *J* = 4.2, 2.1 Hz, 1H), 4.23 (dd, *J* = 15.8, 5.4 Hz, 1H), 4.09 – 3.93 (m, 6H), 3.72 – 3.55 (m, 6H), 3.43 (d, *J* = 7.7 Hz, 2H), 3.20 (ddd, *J* = 28.0, 12.6, 5.2 Hz, 4H), 3.07 (t, *J* = 12.8 Hz, 1H), 2.91 – 2.85 (m, 1H), 2.80 (dt, *J* = 14.7, 7.7 Hz, 1H), 2.65 – 2.54 (m, 2H), 2.45 (s, 3H), 2.07 – 2.03 (m, 1H), 1.93 (ddd, *J* = 13.0, 8.8, 4.6 Hz, 1H), 1.45 – 1.26 (m, 4H), 1.21 (dd, *J* = 6.9, 1.4 Hz, 3H), 0.98 (s, 9H).

^{13}C NMR (151 MHz, DMSO-*d*$_6$) δ (ppm): 172.32, 169.74, 169.58 (“d”, *J* = 2.8 Hz), 169.16, 157.36 (“d”, *J* = 8.2 Hz), 152.46, 151.97, 151.11, 148.84, 148.18, 147.11 (“d”, *J* = 16.0 Hz), 139.97, 136.22, 131.63, 131.09, 130.13, 129.12, 128.68 (“d”, *J* = 4.8 Hz), 127.89, 127.37 (“d”, *J* = 4.0 Hz), 126.42 (“d”, *J* = 5.1 Hz), 123.39, 116.56, 106.80, 69.60 (“d”, *J* = 7.2 Hz), 69.36, 59.23, 57.18, 56.95, 53.46, 52.39, 51.29, 45.80, 42.12, 41.47 (“d”, *J* = 17.2 Hz), 40.91, 40.71 (“d”, *J* = 6.2 Hz), 38.52, 37.40, 36.69, 36.48, 35.92, 35.39 (“d”, *J* = 17.3 Hz), 34.64 (“d”, *J* = 20.0 Hz), 29.69, 26.84, 22.46 (“d”, *J* = 29.8 Hz), 16.41.

Synthesis of (2*S*,4*R*)-4-hydroxy-1-((*S*)-2-(5-(4-(4-(5-((4-hydroxy-1-((*R*)-3-phenylbutanoyl)-piperidin-4-yl)methyl)-4-oxo-4,5-dihydro-1*H*-pyrazolo[3,4-*d*]pyrimidin-1-yl)phenyl)piperazin-1-yl)pentanamido)-3,3-dimethylbutanoyl)-*N*-(4-(4-methylthiazol-5-yl)benzyl)pyrrolidine-2-carboxamide TFA salt (NK238)

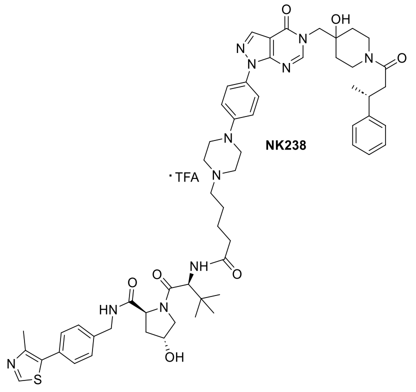

(2*S*,4*R*)-4-hydroxy-1-((*S*)-2-(5-(4-(4-(5-((4-hydroxy-1-((*R*)-3-phenylbutanoyl)-piperidin-4-yl)methyl)-4-oxo-4,5-dihydro-1*H*-pyrazolo[3,4-*d*]pyrimidin-1-yl)phenyl)piperazin-1-yl)pentanamido)-3,3-dimethylbutanoyl)-*N*-(4-(4-methylthiazol-5-yl)benzyl)pyrrolidine-2-carboxamide TFA salt (NK238) was synthesized as described for NK239 (Supplementary Figure 13, exchanging (*R*)-1-(4-(5-((4-hydroxy-1-(3-phenylbutanoyl)piperidin-4-yl)methyl)-4-oxo-4,5-dihydro-1*H*-pyrazolo[3,4-*d*]pyrimidin-1-yl)phenyl)piperidine-4-carboxylic acid (NK226) for (*R*)-5-(4-(4-(5-((4-hydroxy-1-(3-phenylbutanoyl)piperidin-4-yl)methyl)-4-oxo-4,5-dihydro-1*H*-pyrazolo[3,4-*d*]pyrimidin-1-yl)phenyl)piperazin-1-yl)pentanoic acid (NK236).

HRMS (m/z) $[M+2H]^{2+}$ calculated for $C_{58}H_{75}N_{11}O_7S^{2+}$: 534.7781, found: 534.7774.

^{1}H NMR (600 MHz, DMSO-*d*$_6$) δ (ppm): 9.48 (s, 1H), 8.98 (s, 1H), 8.56 (t, *J* = 6.1 Hz, 1H), 8.31 (s, 1H), 8.28 (d, *J* = 13.9 Hz, 1H), 7.95 (d, *J* = 9.3 Hz, 1H), 7.86 (d, *J* = 9.1 Hz, 2H), 7.44 – 7.36 (m, 4H), 7.26 (dd, *J* = 8.4, 6.1 Hz, 4H), 7.20 – 7.13 (m, 3H), 4.56 (d, *J* = 9.3 Hz, 1H), 4.43 (dt, *J* = 16.2, 7.1 Hz, 2H), 4.37 (dt, *J* = 4.5, 2.1 Hz, 1H), 4.22 (dd, *J* = 15.8, 5.5 Hz, 2H), 4.06 – 4.03 (m, 2H), 3.95 – 3.91 (m, 2H), 3.70 – 3.64 (m, 3H), 3.59 (d, *J* = 11.9 Hz, 2H), 3.24 (t, *J* = 11.3 Hz, 1H), 3.17 (dd, *J* = 9.3, 5.3 Hz, 5H), 3.08 – 3.00 (m, 2H), 2.90 – 2.83 (m, 1H), 2.65 – 2.54 (m, 2H), 2.44 (s, 3H), 2.33 (dt, *J* = 14.5, 7.3 Hz, 1H), 2.24 (dt, *J* = 14.5, 7.2 Hz, 1H), 2.07 – 2.02 (m, 1H), 1.91 (ddd, *J* = 12.9, 8.7, 4.6 Hz, 1H), 1.67 (p, *J* = 7.7 Hz, 2H), 1.59 – 1.52 (m, 2H), 1.39 – 1.23 (m, 4H), 1.20 (dd, *J* = 6.9, 1.4 Hz, 3H), 0.96 (s, 9H).

^{13}C NMR (151 MHz, DMSO-*d*$_6$) δ (ppm): 171.89, 171.60, 169.65, 169.10 (“d”, *J* = 2.8 Hz), 156.88 (“d”, *J* = 8.0 Hz), 151.98, 151.48, 150.64, 148.38, 147.70, 146.63 (“d”, *J* = 15.9 Hz), 139.49, 135.75, 131.15, 130.63, 129.64, 128.64, 128.20 (“d”, *J* = 4.7 Hz), 127.41, 126.90 (“d”, *J* = 4.1 Hz), 125.94 (“d”, *J* = 5.0 Hz), 122.92, 116.09, 106.32, 69.12 (“d”, *J* = 7.2 Hz), 68.88, 58.72, 56.45, 56.39, 55.25, 52.98, 50.68 (d, *J* = 11.5 Hz), 45.38, 41.64, 41.00 (“d”, *J* = 17.0 Hz), 40.43, 40.23 (“d”, *J* = 6.2 Hz), 38.04, 36.92, 36.11 (“d”, *J* = 31.1 Hz), 35.93, 35.27, 34.92 (“d”, *J* = 17.3 Hz), 34.23, 34.06, 26.40, 22.91, 22.44, 21.99 (“d”, *J* = 29.7 Hz), 15.93.

Synthesis of 7-((1*r*,4*S*)-4-(4-(4-(5-((4-hydroxy-1-((*R*)-3-phenylbutanoyl)piperidin-4-yl)methyl)-4-oxo-4,5-dihydro-1*H*-pyrazolo[3,4-d]pyrimidin-1-yl)phenyl)piperazine-1-carbonyl)cyclohexane-1-carbonyl)-*N*-((*S*)-1-((2*S*,4*R*)-4-hydroxy-2-((4-(4-methylthiazol-5-yl)benzyl)carbamoyl)pyrrolidin-1-yl)-3,3-dimethyl-1-oxobutan-2-yl)-7-azaspiro[3.5]nonane-2-carboxamide (NK242)

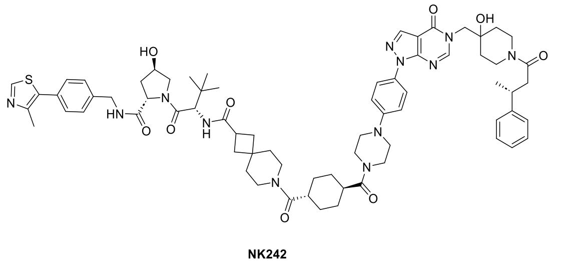

NK242

7-((1*r*,4*S*)-4-(4-(4-(5-((4-hydroxy-1-((*R*)-3-phenylbutanoyl)piperidin-4-yl)methyl)-4-oxo-4,5-dihydro-1*H*-pyrazolo[3,4-*d*]pyrimidin-1-yl)phenyl)piperazine-1-carbonyl)cyclohexane-1-carbonyl)-*N*-((*S*)-1-((2*S*,4*R*)-4-hydroxy-2-((4-(4-methyl-thiazol-5-yl)benzyl)carbamoyl)-pyrrolidin-1-yl)-3,3-dimethyl-1-oxobutan-2-yl)-7-azaspiro[3.5]nonane-2-carboxamide (NK242) was synthesized as described for NK239 (Supplementary Figure 13, exchanging (*R*)-1-(4-(5-((4-hydroxy-1-(3-phenylbutanoyl)piperidin-4-yl)methyl)-4-oxo-4,5-dihydro-1*H*-pyrazolo[3,4-*d*]pyrimidin-1-yl)phenyl)piperidine-4-carboxylic acid (NK226) for (1r,4r)-4-(4-(4-(5-((4-hydroxy-1-((R)-3-phenylbutanoyl)piperidin-4-yl)methyl)-4-oxo-4,5-dihydro-1*H*-pyrazolo[3,4-*d*]pyrimidin-1-yl)phenyl)piperazine-1-carbonyl)cyclohexane-1-carboxylic acid (NK231) and *N*-((*S*)-1-((2*S*,4*R*)-4-hydroxy-2-((4-(4-methylthiazol-5-yl)benzyl)carbamoyl)pyrrolidin-1-yl)-3,3-dimethyl-1-oxobutan-2-yl)-7-azaspiro[3.5]nonane-2-carboxamide (NK216) for 2*S*,4*R*)-1-((*S*)-2-amino-3,3-dimethyl-butanoyl)-4-hydroxy-*N*-(4-(4-methylthiazol-5-yl)benzyl)-pyrrolidine-2-carboxamide.

HRMS (m/z) $[M+2H]^{2+}$ calculated for $C_{70}H_{90}N_{12}O_9S^{2+}$: 673.3332, found: 673.3322.

^{1}H NMR (600 MHz, DMSO-d_6) δ (ppm): 8.99 (s, 1H), 8.56 (t, *J* = 6.1 Hz, 1H), 8.30 – 8.26 (m, 2H), 7.82 (d, *J* = 9.0 Hz, 2H), 7.70 (dd, *J* = 9.8, 5.2 Hz, 1H), 7.43 – 7.37 (m, 4H), 7.26 (dd, *J* = 8.7, 6.1 Hz, 4H), 7.17 – 7.12 (m, 3H), 4.53 (d, *J* = 9.3 Hz, 1H), 4.42 (td, *J* = 8.7, 4.7 Hz, 2H), 4.35 (d, *J* = 3.6 Hz, 1H), 4.21 (dd, *J* = 15.8, 5.5 Hz, 1H), 4.05 (q, *J* = 8.6, 7.0 Hz, 1H), 3.98 (d, *J* = 14.0 Hz, 1H), 3.94 (d, *J* = 3.5 Hz, 1H), 3.67 (dd, *J* = 10.9, 4.6 Hz, 6H), 3.62 (p, *J* = 3.3, 2.9 Hz, 1H), 3.41 (d, *J* = 10.5 Hz, 2H), 3.31 (s, 2H), 3.26– 3.13 (m, 8H), 2.89 – 2.84 (m, 1H), 2.66 – 2.56 (m, 2H), 2.44 (s, 3H), 2.03 (ddd, *J* = 10.2, 8.1, 2.4 Hz, 1H), 1.99 – 1.89 (m, 3H), 1.82 (d, *J* = 8.0 Hz, 2H), 1.68 (d, *J* = 26.6 Hz, 3H), 1.51 (d, *J* = 48.0 Hz, 8H), 1.39 – 1.24 (m, 6H), 1.20 (dd, *J* = 7.0, 1.2 Hz, 3H), 0.92 (s, 9H).

^{13}C NMR (151 MHz, DMSO-d_6) δ (ppm): 174.08, 173.29, 172.96, 172.94, 171.94, 169.62, 169.10 (“d”, *J* = 2.7 Hz), 156.91 (“d”, *J* = 7.9 Hz), 151.91 (“d”, *J* = 2.8 Hz), 151.48, 150.67 – 150.49 (“d”), 149.66, 147.69, 146.63 (“d”, *J* = 16.5 Hz), 139.51, 135.60, 131.17, 129.93, 129.62, 128.64, 128.20 (“d”, *J* = 5.0 Hz), 127.42, 126.90 (“d”, *J* = 3.9 Hz), 125.94 (“d”, *J* = 5.7 Hz), 122.84, 115.74, 106.25, 69.11 (“d”, *J* = 7.3 Hz), 68.88, 58.70, 56.43, 56.37, 53.55, 52.99, 48.75, 48.12, 44.48, 41.63, 41.00 (“d”, *J* = 16.8 Hz), 40.79, 40.23 (“d”, *J* = 5.7 Hz), 38.92, 38.56 (“d”, *J* = 10.0 Hz), 37.94, 36.92, 36.11 (“d”, *J* = 31.2 Hz), 35.34, 34.90, 34.84, 34.17 (d, *J* = 20.1 Hz), 34.04, 33.55, 31.68, 28.22, 28.18, 26.34, 21.99 (“d”, *J* = 29.9 Hz), 18.07, 16.73, 15.93, 12.44.

Synthesis of (2*S*,4*S*)-4-hydroxy-1-((*S*)-2-((1*r*,4*S*)-4-(4-(4-(5-((4-hydroxy-1-((*R*)-3-phenylbutanoyl)piperidin-4-yl)methyl)-4-oxo-4,5-dihydro-1*H*-pyrazolo[3,4-*d*]pyrimidin-1-yl)phenyl)piperazine-1-carbonyl)cyclohexane-1-carboxamido)-3,3-dimethylbutanoyl)-*N*-(4-(4-methylthiazol-5-yl)benzyl)pyrrolidine-2-carboxamide (NK245)

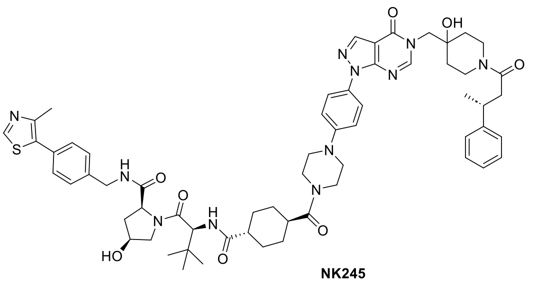

NK245

(2*S*,4*S*)-4-hydroxy-1-((*S*)-2-((1*r*,4*S*)-4-(4-(4-(5-((4-hydroxy-1-((*R*)-3-phenylbutanoyl)piperidin-4-yl)methyl)-4-oxo-4,5-dihydro-1*H*-pyrazolo[3,4-*d*]-pyrimidin-1-yl)phenyl)piperazine-1-carbonyl)-cyclohexane-1-carboxamido)-3,3-dimethylbutanoyl)-N-(4-(4-methylthiazol-5-yl)benzyl)pyrrolidine-2-carboxamide (NK245) was synthesized as described for NK239 (Supplementary Figure 13, exchanging (*R*)-1-(4-(5-((4-hydroxy-1-(3-phenylbutanoyl)piperidin-4-yl)methyl)-4-oxo-4,5-dihydro-1*H*-pyrazolo[3,4-*d*]pyrimidin-1-yl)phenyl)piperidine-4-carboxylic acid (NK226) for (1*r*,4*r*)-4-(4-(4-(5-((4-hydroxy-1-((*R*)-3-phenylbutanoyl)piperidin-4-yl)methyl)-4-oxo-4,5-dihydro-1*H*-pyrazolo[3,4-*d*]pyrimidin-1-yl)phenyl)piperazine-1-carbonyl)cyclohexane-1-carboxylic acid (NK231) and 2*S*,4*S*)-1-((*S*)-2-amino-3,3-dimethyl-butanoyl)-4-hydroxy-*N*-(4-(4-methylthiazol-5-yl)benzyl)-pyrrolidine-2-carboxamide for (2*S*,4*R*)-1-((*S*)-2-amino-3,3-dimethyl-butanoyl)-4-hydroxy-*N*-(4-(4-methylthiazol-5-yl)benzyl)-pyrrolidine-2-carboxamide .

HRMS (m/z) $[M+2H]^{2+}$ calculated for $C_{61}H_{77}N_{11}O_8S^{2+}$: 561.7833, found: 561.7828.

^{1}H NMR (700 MHz, DMSO-d_6) δ (ppm): 8.92 (s, 1H), 8.58 (t, *J* = 6.1 Hz, 1H), 8.23 (d, *J* = 1.1 Hz, 1H), 8.21 (d, *J* = 16.5 Hz, 1H), 7.76 (d, *J* = 9.0 Hz, 2H), 7.68 (d, *J* = 8.8 Hz, 1H), 7.33 (q, *J* = 8.4 Hz, 4H), 7.21 – 7.18 (m, 4H), 7.10 – 7.05 (m, 3H), 4.40 – 4.36 (m, 2H), 4.30 (dd, *J* = 8.6, 6.1 Hz, 1H), 4.19 (dd, *J* = 15.7, 5.5 Hz, 1H), 4.15 (d, *J* = 5.6 Hz, 1H), 4.00 – 3.94 (m, 2H), 3.93 – 3.88 (m, 1H), 3.89 – 3.83 (m, 1H), 3.55 (s, 6H), 3.38 (dd, *J* = 10.1, 5.3 Hz, 1H), 3.18 – 3.07 (m, 6H), 2.82 – 2.77 (m, 1H), 2.57 – 2.50 (m, 2H), 2.38 (s, 3H), 2.27 (ddd, *J* = 12.6, 8.5, 5.7 Hz, 1H), 1.72 – 1.58 (m, 5H), 1.48 – 1.24 (m, 8H), 1.14 (d, *J* = 6.9 Hz, 3H), 0.89 (s, 9H).

^{13}C NMR (176 MHz, DMSO-d_6) δ (ppm): 175.62, 173.77, 172.97, 170.41, 169.58 (“d”, *J* = 3.1 Hz), 157.39 (“d”, *J* = 8.7 Hz), 152.39, 151.97, 151.04, 150.14, 148.20, 147.11 (“d”, *J* = 19.3 Hz), 139.68, 136.09 (“d”, *J* = 5.5 Hz), 131.62, 130.41, 130.20, 129.14, 128.68 (“d”, *J* = 5.9 Hz), 127.93, 127.37 (“d”, *J* = 4.7 Hz), 126.42 (“d”, *J* = 6.3 Hz), 123.32, 116.21, 106.74, 69.61 (“d”, *J* = 3.9 Hz), 69.57, 58.99, 56.90, 56.07, 53.47, 49.20, 48.53, 44.95, 43.03, 42.26, 41.42, 41.25 (“d”, *J* = 3.1 Hz), 40.71 (“d”, *J* = 6.5 Hz), 38.85, 37.39, 36.59 (“d”, *J* = 36.1 Hz), 35.32, 34.65 (“d”, *J* = 22.2 Hz), 29.43, 28.88, 28.64, 28.28, 26.84, 22.46 (“d”, *J* = 34.6 Hz), 16.41.

3.7 Synthesis of VHL-alkylcarboxy-functionalized PROTACs

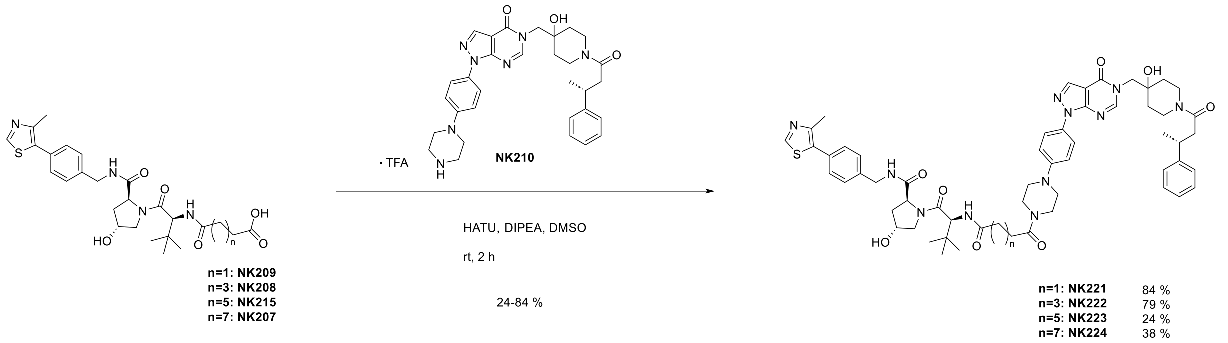

Supplementary Figure 14. Synthesis of PROTACs NK221, NK222, NK223 and NK224.

Synthesis of (2*S*,4*R*)-4-hydroxy-1-((*S*)-2-(4-(4-(4-(5-((4-hydroxy-1-((*R*)-3-phenylbutanoyl)-piperidin-4-yl)methyl)-4-oxo-4,5-dihydro-1*H*-pyrazolo[3,4-*d*]pyrimidin-1-yl)phenyl)piperazin-1-yl)-4-oxobutanamido)-3,3-dimethylbutanoyl)-*N*-(4-(4-methylthiazol-5-yl)benzyl)pyrrolidine-2-carboxamide (NK221)

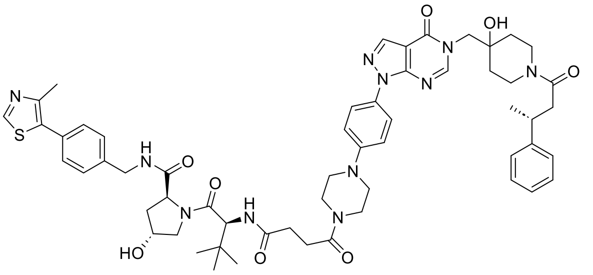

NK221

4-(((*S*)-1-((2*S*,4*R*)-4-Hydroxy-2-((4-(4-methylthiazol-5-yl)benzyl)carbamoyl)pyrrolidin-1-yl)-3,3-dimethyl-1-oxobutan-2-yl)amino)-4-oxobutanoic acid (NK209) (9.3 mg, 0.02 mmol, 1.1 eq), HATU (7.4 mg, 0.02 mmol, 1.3 eq) and DIPEA (7.2 mg, 0.06 mmol, 4 eq) were dissolved in DMSO (1 mL) and stirred at rt for 15 min. To this was added (*R*)-5-((4-hydroxy-1-(3-phenylbutanoyl)piperidin-4-yl)methyl)-1-(4-(piperazin-1-yl)phenyl)-1,5-dihydro-4*H*-pyrazolo[3,4-*d*]pyrimidin-4-one (NK210) and the reaction was stirred for 2 h at rt. After completion, the crude product was purified by preparative reverse phase column chromatography (ACN/H_2O + 0.1 % TFA). After evaporation of the solvents under reduced pressure, the product (2*S*,4*R*)-4-hydroxy-1-((*S*)-2-(4-(4-(4-(5-((4-hydroxy-1-((*R*)-3-phenylbutanoyl)-piperidin-4-yl)methyl)-4-oxo-4,5-dihydro-1*H*-pyrazolo[3,4-*d*]pyrimidin-1-yl)phenyl)piperazin-1-yl)-4-oxobutanamido)-3,3-dimethylbutanoyl)-*N*-(4-(4-methylthiazol-5-yl)benzyl)pyrrolidine-2-carboxamide (NK221) (13.4 mg, 0.01 mmol, 84 %) was obtained as a white solid.

HRMS (m/z) $[M+2H]^{2+}$ calculated for $C_{57}H_{71}N_{11}O_8S^{2+}$: 534.7599, found: 534.7592.

^{1}H NMR (500 MHz, DMSO-d_6) δ (ppm): 8.99 (s, 1H), 8.56 (t, *J* = 6.1 Hz, 1H), 8.29 (s, 1H), 8.28 (d, *J* = 11.9 Hz, 1H), 7.93 (d, *J* = 9.3 Hz, 1H), 7.82 (d, *J* = 9.2 Hz, 2H), 7.40 (q, *J* = 8.4 Hz, 4H), 7.26 (dd, *J* = 7.4, 5.6 Hz, 4H), 7.18 – 7.11 (m, 3H), 4.53 (d, *J* = 9.3 Hz, 1H), 4.47 – 4.40 (m, 2H), 4.35 (tt, *J* = 4.4, 2.3 Hz, 1H), 4.22 (dd, *J* = 15.8, 5.5 Hz, 1H), 4.08 – 3.93 (m, 3H), 3.69 – 3.59 (m, 7H), 3.29 – 3.12 (m, 6H), 2.90 – 2.85 (m, 1H), 2.64 – 2.54 (m, 5H), 2.45 (s, 3H), 2.43 – 2.40 (m, 1H), 2.06 – 2.00 (m, 1H), 1.90 (ddd, *J* = 12.9, 8.6, 4.6 Hz, 1H), 1.57 – 1.24 (m, 4H), 1.20 (d, *J* = 6.9 Hz, 3H), 0.94 (s, 9H).

^{13}C NMR (126 MHz, DMSO-d_6) δ (ppm): 171.94, 171.28, 170.05, 169.59, 169.10, 156.91 („d“, *J* = 6.7 Hz), 151.90, 151.50, 150.55, 149.62, 147.65, 146.64 („d“, *J* = 13.6 Hz), 139.53, 135.60, 131.20, 129.91, 129.61, 128.65, 128.20 („d“, *J* = 4.0 Hz), 127.43, 126.90 („d“, *J* = 3.2 Hz), 125.96, 122.86, 115.72, 106.26, 69.12 („d“, *J* = 6.0 Hz), 68.89, 58.72, 56.45, 56.30, 53.00, 48.15 („d“, *J* = 46.9 Hz), 44.48, 41.65, 40.91 („d“, *J* = 10.0 Hz), 40.23 („d“, *J* = 4.5 Hz), 37.94, 36.92, 36.12 („d“, *J* = 25.9 Hz), 35.37, 34.90 („d“, *J* = 15.6 Hz), 34.18 („d“, *J* = 17.3 Hz), 30.14, 27.97, 26.39, 21.99 („d“, *J* = 24.8 Hz), 15.92.

Synthesis of (2*S*,4*R*)-4-hydroxy-1-((*S*)-2-(6-(4-(4-(5-((4-hydroxy-1-((*R*)-3-phenylbutanoyl)-piperidin-4-yl)methyl)-4-oxo-4,5-dihydro-1*H*-pyrazolo[3,4-*d*]pyrimidin-1-yl)phenyl)piperazin-1-yl)-6-oxohexanamido)-3,3-dimethylbutanoyl)-*N*-(4-(4-methylthiazol-5-yl)benzyl)pyrrolidine-2-carboxamide (NK222)

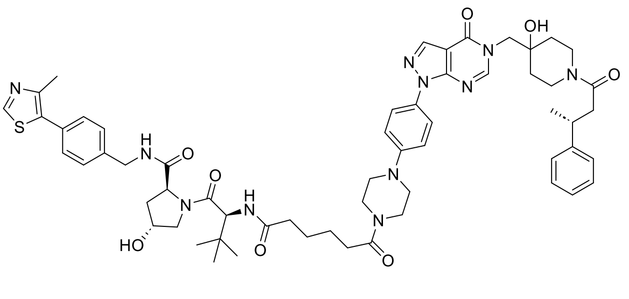

NK222

(2*S*,4*R*)-4-Hydroxy-1-((*S*)-2-(6-(4-(4-(5-((4-hydroxy-1-((*R*)-3-phenylbutanoyl)-piperidin-4-yl)methyl)-4-oxo-4,5-dihydro-1*H*-pyrazolo[3,4-*d*]pyrimidin-1-yl)phenyl)piperazin-1-yl)-6-oxohexanamido)-3,3-dimethylbutanoyl)-*N*-(4-(4-methylthiazol-5-yl)benzyl)pyrrolidine-2-carboxamide (NK222) was synthesized as described for NK221 (Supplementary Figure 14, exchanging 4-(((*S*)-1-((2*S*,4*R*)-4-hydroxy-2-((4-(4-methylthiazol-5-yl)benzyl)carbamoyl)pyrrolidin-1-yl)-3,3-dimethyl-1-oxobutan-2-yl)amino)-4-oxobutanoic acid (NK209) for 6-(((*S*)-1-((2*S*,4*R*)-4-hydroxy-2-((4-(4-methylthiazol-5-yl)benzyl)carbamoyl)-pyrrolidin-1-yl)-3,3-dimethyl-1-oxobutan-2-yl)amino)-6-oxohexanoic acid (NK208)).

HRMS (m/z) $[M+2H]^{2+}$ calculated for $C_{59}H_{75}N_{11}O_8S^{2+}$: 548.7755 Found: 548.7752.

^{1}H NMR (700 MHz, DMSO-d_6) δ (ppm): 8.98 (s, 1H), 8.55 (t, *J* = 6.1 Hz, 1H), 8.29 (d, *J* = 1.1 Hz, 1H), 8.27 (d, *J* = 16.5 Hz, 1H), 7.87 (d, *J* = 9.3 Hz, 1H), 7.83 – 7.81 (m, 2H), 7.42 (d, *J* = 8.2 Hz, 2H), 7.38 (d, *J* = 8.3 Hz, 2H), 7.27 – 7.24 (m, 4H), 7.15 (td, *J* = 6.3, 3.6 Hz, 1H), 7.13 – 7.11 (m, 2H), 4.55 (d, *J* = 9.4 Hz, 1H), 4.45 – 4.41 (m, 2H), 4.35 (dt, *J* = 4.4, 2.1 Hz, 1H), 4.21 (dd, *J* = 15.8, 5.4 Hz, 1H), 4.07 – 3.93 (m, 3H), 3.66 (dd, *J* = 12.6, 8.5 Hz, 4H), 3.62 (t, *J* = 5.3 Hz, 2H), 3.24 (t, *J* = 5.4 Hz, 2H), 3.20 – 3.14 (m, 4H), 2.90 – 2.83 (m, 1H), 2.63 – 2.53 (m, 3H), 2.44 (s, 3H), 2.37 (dt, *J* = 7.6, 3.3 Hz, 2H), 2.30 (dt, *J* = 10.5, 7.1 Hz, 1H), 2.17 – 2.12 (m, 1H), 2.05 – 2.01 (m, 1H), 1.90 (ddd, *J* = 12.9, 8.6, 4.6 Hz, 1H), 1.56 – 1.53 (m, 2H), 1.45 – 1.22 (m, 3H), 1.20 (dd, *J* = 6.9, 1.5 Hz, 3H), 0.93 (s, 9H).

^{13}C NMR (176 MHz, DMSO-d_6) δ (ppm): 171.96, 171.94, 170.62, 169.70, 169.10 (“d”, *J* = 3.0 Hz), 151.88, 151.46, 150.54, 149.68, 147.70, 146.64 (“d”, *J* = 19.7 Hz), 139.51, 135.59, 131.16, 129.89, 129.62, 128.63, 128.20 (“d”, *J* = 5.9 Hz), 127.41, 126.90 (“d”, *J* = 4.7 Hz), 125.94 (“d”, *J* = 6.6 Hz), 122.85, 122.84, 115.72, 106.25, 69.11 (“d”, *J* = 8.3 Hz), 68.86, 58.68, 56.36, 56.29, 53.00, 48.23 (“d”, *J* = 73.8 Hz), 44.60, 41.64, 40.99 (“d”, *J* = 18.7 Hz), 40.71, 40.69, 40.23 (“d”, *J* = 6.9 Hz), 40.02, 37.95, 36.97 – 36.86 (m), 36.11 (“d”, *J* = 36.5 Hz), 35.22, 34.69, 34.17 (“d”, *J* = 23.4 Hz), 31.98, 26.39, 25.22, 24.43, 21.99 (“d”, *J* = 34.8 Hz), 15.94.

Synthesis of (2*S*,4*R*)-4-hydroxy-1-((*S*)-2-(8-(4-(4-(5-((4-hydroxy-1-((*R*)-3-phenylbutanoyl)-piperidin-4-yl)methyl)-4-oxo-4,5-dihydro-1*H*-pyrazolo[3,4-*d*]pyrimidin-1-yl)phenyl)piperazin-1-yl)-8-oxooctanamido)-3,3-dimethylbutanoyl)-*N*-(4-(4-methylthiazol-5-yl)benzyl)pyrrolidine-2-carboxamide (NK223)

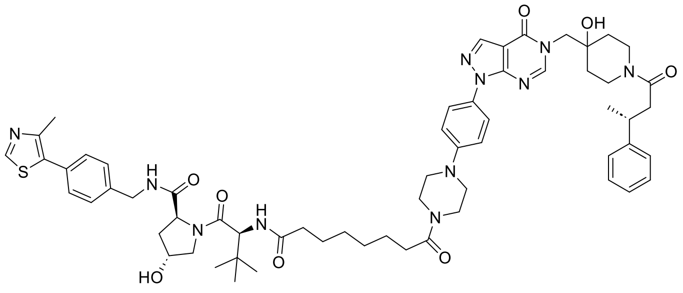

NK223

(2*S*,4*R*)-4-Hydroxy-1-((*S*)-2-(8-(4-(4-(5-((4-hydroxy-1-((*R*)-3-phenylbutanoyl)-piperidin-4-yl)methyl)-4-oxo-4,5-dihydro-1*H*-pyrazolo[3,4-*d*]pyrimidin-1-yl)phenyl)piperazin-1-yl)-8-oxooctanamido)-3,3-dimethylbutanoyl)-*N*-(4-(4-methylthiazol-5-yl)benzyl)pyrrolidine-2-carboxamide (NK223) was synthesized as described for NK221 (Supplementary Figure 14, exchanging 4-(((*S*)-1-((2*S*,4*R*)-4-hydroxy-2-((4-(4-methylthiazol-5-yl)benzyl)carbamoyl)pyrrolidin-1-yl)-3,3-dimethyl-1-oxobutan-2-yl)amino)-4-oxobutanoic acid (NK209) for 8-(((*S*)-1-((2*S*,4*R*)-4-hydroxy-2-((4-(4-methylthiazol-5-yl)benzyl)carbamoyl)-pyrrolidin-1-yl)-3,3-dimethyl-1-oxobutan-2-yl)amino)-8-oxooctanoic acid (NK215).

HRMS (m/z) $[M+2H]^{2+}$ calculated for $C_{61}H_{77}N_{11}O_8S^{2+}$: 562.7912, found: 562.7907.

^{1}H NMR (600 MHz, DMSO-*d*$_6$) δ (ppm): 8.98 (s, 1H), 8.56 (t, *J* = 6.1 Hz, 1H), 8.29 (d, *J* = 0.8 Hz, 1H), 8.28 (d, *J* = 14.2 Hz, 1H), 7.85 (d, *J* = 9.4 Hz, 1H), 7.82 (d, *J* = 9.1 Hz, 2H), 7.42 – 7.36 (m, 4H), 7.27 – 7.23 (m, 4zH), 7.16 – 7.11 (m, 3H), 4.54 (d, *J* = 9.4 Hz, 1H), 4.45 – 4.40 (m, 2H), 4.35 (dt, *J* = 4.3, 2.0 Hz, 1H), 4.21 (dd, *J* = 15.8, 5.5 Hz, 2H), 4.05 – 4.02 (m, 3H), 3.93 (d, *J* = 3.7 Hz, 2H), 3.69 – 3.64 (m, 2H), 3.61 (t, *J* = 5.1 Hz, 4H), 3.25 – 3.22 (m, 2H), 3.17 (q, *J* = 7.5, 5.7 Hz, 3H), 2.90 – 2.83 (m, 1H), 2.64 – 2.54 (m, 3H), 2.44 (s, 3H), 2.34 (t, *J* = 7.5 Hz, 2H), 2.12 (ddd, *J* = 14.2, 8.1, 6.3 Hz, 1H), 2.03 (ddd, *J* = 11.3, 7.7, 2.3 Hz, 1H), 1.50 (p, *J* = 8.7, 8.2 Hz, 4H), 1.32 – 1.23 (m, 8H), 1.20 (d, *J* = 6.8 Hz, 3H), 0.93 (s, 9H).

^{13}C NMR (151 MHz, DMSO-*d*$_6$) δ (ppm): 172.57, 172.43, 171.20, 170.19, 169.58 („d“, *J* = 2.7 Hz), 152.37, 151.94, 151.02, 150.14, 148.17, 147.11 (“d”, *J* = 16.6 Hz), 139.99, 136.07, 131.65, 130.23 (“d”, *J* = 42.3 Hz), 129.11, 128.70, 127.89, 127.38 (“d”, *J* = 4.0 Hz), 126.42 (“d”, *J* = 5.6 Hz), 123.32, 116.19, 106.73, 69.59 (“d”, *J* = 7.5 Hz), 69.34, 59.16, 56.79 (“d”, *J* = 12.1 Hz), 53.47, 48.71 (“d”, *J* = 62.5 Hz), 45.09, 42.11, 41.48 (“d”, *J* = 16.9 Hz), 41.16, 40.91, 40.69, 38.43, 37.39, 36.59 (“d”, *J* = 31.3 Hz), 35.68, 35.43, 35.32, 34.65 (“d”, *J* = 20.7 Hz), 32.69, 28.99, 26.86, 25.83, 25.17, 22.46 (“d”, *J* = 30.1 Hz), 16.41.

Synthesis of (2*S*,4*R*)-4-hydroxy-1-((*S*)-2-(10-(4-(4-(5-((4-hydroxy-1-((*R*)-3-phenylbutanoyl)-piperidin-4-yl)methyl)-4-oxo-4,5-dihydro-1*H*-pyrazolo[3,4-*d*]pyrimidin-1-yl)phenyl)piperazin-1-yl)-10-oxodecanamido)-3,3-dimethylbutanoyl)-*N*-(4-(4-methylthiazol-5-yl)benzyl)pyrrolidine-2-carboxamide (NK224)

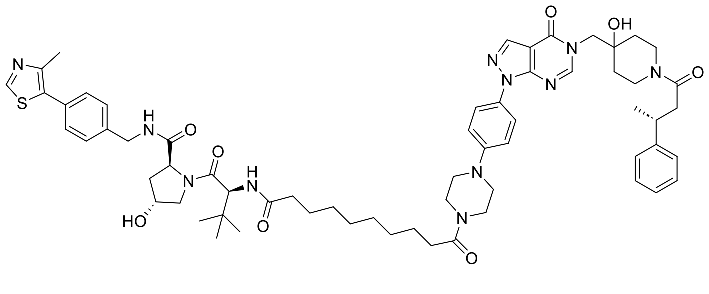

NK224

(2*S*,4*R*)-4-Hydroxy-1-((*S*)-2-(10-(4-(4-(5-((4-hydroxy-1-((*R*)-3-phenylbutanoyl)-piperidin-4-yl)methyl)-4-oxo-4,5-dihydro-1*H*-pyrazolo[3,4-*d*]pyrimidin-1-yl)phenyl)piperazin-1-yl)-10-oxodecanamido)-3,3-dimethylbutanoyl)-*N*-(4-(4-methylthiazol-5-yl)benzyl)pyrrolidine-2-carboxamide (NK224) was synthesized as described for NK221 (Supplementary Figure 14, exchanging 4-(((*S*)-1-((2*S*,4*R*)-4-hydroxy-2-((4-(4-methylthiazol-5-yl)benzyl)carbamoyl)pyrrolidin-1-yl)-3,3-dimethyl-1-oxobutan-2-yl)amino)-4-oxobutanoic acid (NK209) for 10-(((*S*)-1-((2*S*,4*R*)-4-hydroxy-2-((4-(4-methylthiazol-5-yl)benzyl)carbamoyl)-pyrrolidin-1-yl)-3,3-dimethyl-1-oxobutan-2-yl)amino)-10-oxodecanoic acid (NK207).

HRMS (m/z) $[M+2H]^{2+}$ calculated for $C_{63}H_{83}N_{11}O_8S^{2+}$ 576.8068, found: 576.8056.

^{1}H NMR (700 MHz, DMSO-*d*$_6$) δ (ppm): 8.98 (s, 1H), 8.55 (t, *J* = 6.1 Hz, 1H), 8.29 (s, 1H), 8.28 (d, *J* = 16.4 Hz, 1H), 7.84 – 7.81 (m, 3H), 7.42 – 7.37 (m, 4H), 7.28 – 7.24 (m, 4H), 7.16 – 7.11 (m, 3H), 4.54 (d, *J* = 9.4 Hz, 1H), 4.46 – 4.40 (m, 2H), 4.35 (dt, *J* = 4.3, 2.0 Hz, 1H), 4.21 (dd, *J* = 15.8, 5.5 Hz, 1H), 4.06 – 3.92 (m, 4H) 3.68 (d, *J* = 5.8 Hz, 2H), 3.66 (s, 2H), 3.61 (t, *J* = 5.3 Hz, 4H), 3.23 (d, *J* = 6.7 Hz, 1H), 3.20 – 3.15 (m, 1H), 2.87 (t, *J* = 10.9 Hz, 1H), 2.64 – 2.56 (m, 2H), 2.44 (s, 3H), 2.34 (t, *J* = 7.5 Hz, 2H), 2.25 (dt, *J* = 14.7, 7.6 Hz, 1H), 2.11 (ddd, *J* = 14.2, 8.1, 6.2 Hz, 1H), 2.05 – 2.01 (m, 1H), 1.90 (ddd, *J* = 12.9, 8.5, 4.6 Hz, 1H), 1.51 (p, *J* = 7.4 Hz, 4H), 1.32 – 1.23 (m, 12H), 1.20 (dd, *J* = 7.0, 1.5 Hz, 3H), 0.93 (s, 9H).

^{13}C NMR (176 MHz, DMSO-*d*$_6$) (ppm): δ 172.08, 171.93, 170.73, 169.70, 169.09 („d“, *J* = 3.8 Hz), 157.96 („d“, *J* = 34.4 Hz), 151.87, 151.45, 149.66, 147.69, 139.50, 135.58, 131.16, 129.88, 129.62, 128.62, 128.19 („d“, *J* = 5.4 Hz), 127.41, 126.89 („d“, *J* = 4.7 Hz), 126.34, 125.93 („d“, *J* = 6.2 Hz), 122.84, 115.70, 114.82, 106.25, 68.97 („d“, *J* = 42.5 Hz), 58.67, 56.33, 56.25, 52.98, 48.43, 48.02, 46.87, 44.61, 41.63, 40.99 („d“, *J* = 18.7 Hz), 40.68, 40.22 („d“, *J* = 7.1 Hz), 37.94, 36.91, 36.10 („d“, *J* = 36.3 Hz), 35.19, 34.85, 32.24, 28.80 („d“, *J* = 7.6 Hz), 28.69, 28.63, 26.37, 25.42, 24.78, 21.98 („d“, *J* = 34.8 Hz), 16.68, 15.93.

3.8 Synthesis of VHL-cyclohexyl-functionalized PROTACs

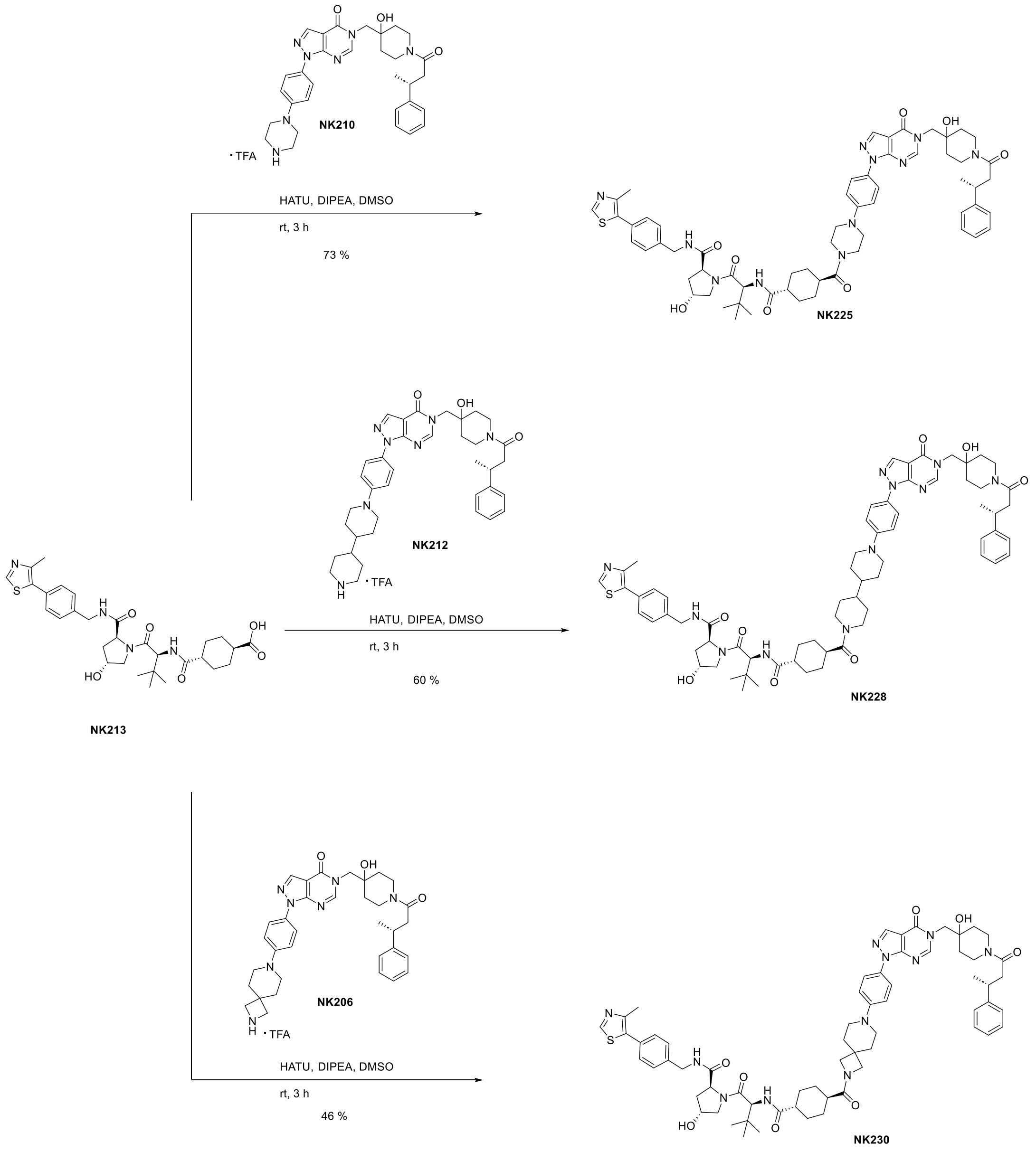

Supplementary Figure 15. Synthesis of cyclohexyl-linker PROTACs NK225, NK228 and NK230.

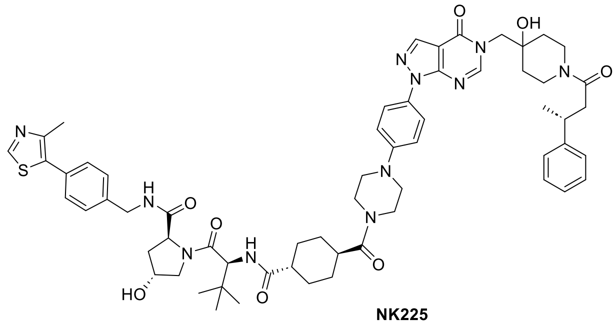

NK225

Synthesis of (2*S*,4*R*)-4-hydroxy-1-((*S*)-2-((1*r*,4*S*)-4-(4-(4-(5-((4-hydroxy-1-((*R*)-3-phenylbutanoyl)-piperidin-4-yl)methyl)-4-oxo-4,5-dihydro-1*H*-pyrazolo[3,4-*d*]pyrimidin-1-yl)phenyl)piperazine-1-carbonyl)cyclohexane-1-carboxamido)-3,3-dimethylbutanoyl)-*N*-(4-(4-methylthiazol-5-yl)benzyl)pyrrolidine-2-carboxamide (NK225)

NK213 (10.5 mg, 0.02 mmol, 1.2 eq), HATU (7.4 mg, 0.02 mmol, 1.3 eq) and DIPEA (7.7 mg, 0.06 mmol, 4.0 eq) were dissolved in DMSO (1 mL) and stirred at rt for 15 min. To this was added NK210 (10.0 mg, 0.01 mmol, 1.0 eq) and the mixture was stirred at rt for 2 h. After completion, the crude product was directly purified by preparative reverse phase column chromatography (ACN/H_2O + 0.1 % TFA). After evaporation of the solvents under reduced pressure, the product (2*S*,4*R*)-4-hydroxy-1-((*S*)-2-

((1*r*,4*S*)-4-(4-(4-(5-((4-hydroxy-1-((*R*)-3-phenylbutanoyl)-piperidin-4-yl)methyl)-4-oxo-4,5-dihydro-1*H*-pyrazolo[3,4-*d*]pyrimidin-1-yl)phenyl)piperazine-1-carbonyl)cyclohexane-1-carboxamido)-3,3-dimethylbutanoyl)-*N*-(4-(4-methylthiazol-5-yl)benzyl)pyrrolidine-2-carboxamide (NK225) (12.2 mg, 0.01 mmol, 73 % yield) was obtained as a white powder.

HRMS (m/z) $[M+2H]^{2+}$ calculated for $C_{61}H_{77}N_{11}O_8S^{2+}$: 561.7833, found: 561.7823.

^1H NMR (600 MHz, DMSO-d_6) δ (ppm): 9.00 (s, 1H), 8.58 (t, *J* = 6.1 Hz, 1H), 8.30 (s, 1H), 8.28 (d, *J* = 14.1 Hz, 1H), 7.83 (d, *J* = 9.0 Hz, 2H), 7.75 (d, *J* = 9.3 Hz, 1H), 7.45 – 7.37 (m, 4H), 7.27 (dd, *J* = 8.6, 6.1 Hz, 4H), 7.19 – 7.11 (m, 3H), 4.55 (s, 2H), 4.47 – 4.42 (m, 4H), 4.36 (dt, *J* = 4.3, 1.9 Hz, 1H), 4.23 (dd, *J* = 15.8, 5.5 Hz, 1H), 4.09 – 4.01 (m, 1H), 4.01 – 3.93 (m, 2H), 3.70 – 3.59 (m, 6H), 3.27 – 3.15 (m, 6H), 2.91 – 2.83 (m, 1H), 2.66 – 2.57 (m, 2H), 2.45 (s, 3H), 2.07 – 2.02 (m, 1H), 1.91 (ddd, *J* = 12.9, 8.6, 4.6 Hz, 1H), 1.83 – 1.79 (m, 1H), 1.71 (q, *J* = 15.1, 12.8 Hz, 3H), 1.54 – 1.30 (m, 8H), 1.21 (dd, *J* = 7.0, 1.2 Hz, 3H), 0.94 (s, 9H).

^{13}C NMR (151 MHz, DMSO-d_6) δ (ppm): 175.29, 173.78, 172.43, 170.12, 169.58 (“d”, *J* = 2.9 Hz), 157.39 (“d”, *J* = 8.1 Hz), 152.38 (“d”, *J* = 3.0 Hz), 151.98, 151.03 (“d”, *J* = 1.7 Hz), 150.12, 148.14, 147.11 (“d”, *J* = 16.5 Hz), 140.00, 136.08, 131.67, 130.42, 130.09, 129.12, 128.68 (“d”, *J* = 5.0 Hz), 127.91, 127.37 (“d”, *J* = 4.1 Hz), 126.42 (“d”, *J* = 5.5 Hz), 123.32, 116.23, 106.74, 69.59 (“d”, *J* = 7.2 Hz), 69.36, 59.19, 56.82, 56.54, 53.47, 49.23, 48.59, 44.95, 43.16, 42.12, 41.47 (“d”, *J* = 17.3 Hz), 41.25, 40.71 (“d”, *J* = 6.2 Hz), 38.87, 38.42, 37.39, 36.59 (“d”, *J* = 31.1 Hz), 35.92, 35.37 (“d”, *J* = 17.1 Hz), 34.65 (“d”, *J* = 19.5 Hz), 29.62, 28.91, 28.63, 28.19, 26.84, 22.46 (“d”, *J* = 29.9 Hz), 16.40.

Synthesis of (2*S*,4*R*)-4-hydroxy-1-((*S*)-2-((1*r*,4*S*)-4-(1'-(4-(5-((4-hydroxy-1-((*R*)-3-phenylbutanoyl)piperidin-4-yl)methyl)-4-oxo-4,5-dihydro-1*H*-pyrazolo[3,4-*d*]pyrimidin-1-yl)phenyl)-[4,4'-bipiperidine]-1-carbonyl)cyclohexane-1-carboxamido)-3,3-dimethylbutanoyl)-*N*-(4-(4-methylthiazol-5-yl)benzyl)pyrrolidine-2-carboxamide (NK228)

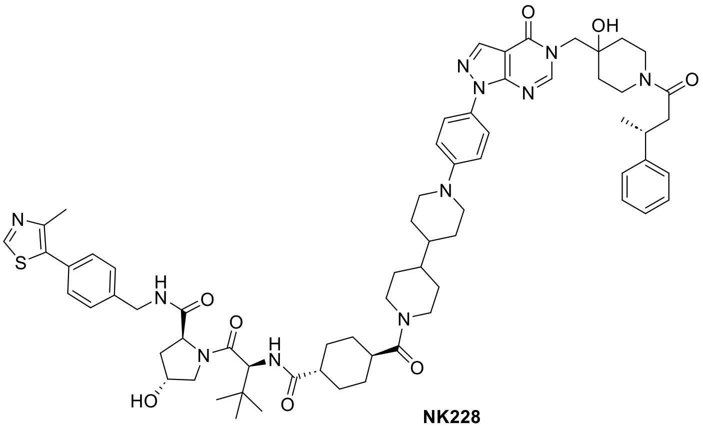

NK228

(2*S*,4*R*)-4-Hydroxy-1-((*S*)-2-((1*r*,4*S*)-4-(1'-(4-(5-((4-hydroxy-1-((*R*)-3-phenylbutanoyl)piperidin-4-yl)methyl)-4-oxo-4,5-dihydro-1*H*-pyrazolo[3,4-*d*]pyrimidin-1-yl)phenyl)-[4,4'-bipiperidine]-1-carbonyl)cyclohexane-1-carboxamido)-3,3-dimethylbutanoyl)-*N*-(4-(4-methylthiazol-5-yl)benzyl)pyrrolidine-2-carboxamide (NK228) was synthesized as described for NK239 (Supplementary Figure 15, exchanging (*R*)-5-((4-hydroxy-1-(3-phenylbutanoyl)piperidin-4-yl)methyl)-1-(4-(piperazin-1-yl)phenyl)-1,5-dihydro-4*H*-pyrazolo[3,4-*d*]pyrimidin-4-one (NK210) for (*R*)-1-(4-([4,4'-bipiperidin]-1-yl)phenyl)-5-((4-hydroxy-1-(3-phenylbutanoyl)piperidin-4-yl)methyl)-1,5-dihydro-4*H*-pyrazolo[3,4-*d*]pyrimidin-4-one TFA salt (NK212).

HRMS (m/z) $[M+2H]^{2+}$ calculated for $C_{67}H_{87}N_{11}O_8S^{2+}$: 602.8225, found: 602.8226.

^1H NMR (600 MHz, DMSO-d_6) δ (ppm): 9.00 (s, 1H), 8.57 (t, *J* = 6.1 Hz, 1H), 8.30 (s, 1H), 8.28 (d, *J* = 14.1 Hz, 1H), 7.82 (d, *J* = 8.5 Hz, 2H), 7.74 (d, *J* = 9.2 Hz, 1H), 7.44 – 7.37 (m, 4H), 7.29 – 7.24 (m, 4H), 7.18 – 7.14 (m, 3H), 4.53 (d, *J* = 9.3 Hz, 1H), 4.46 – 4.41 (m, 3H), 4.35 (td, *J* = 4.3, 2.2 Hz, 1H), 4.22 (dd, *J* = 15.8, 5.5 Hz, 1H), 4.08 – 3.96 (m, 4H), 3.94 (d, *J* = 3.9 Hz, 1H), 3.81 (d, *J* = 12.0 Hz, 2H), 3.70 – 3.65 (m, 5H), 3.26 – 3.13 (m, 3H), 2.91 (dt, *J* = 42.8, 12.9 Hz, 2H), 2.76 (s, 1H), 2.64 – 2.56 (m, 2H), 2.45 (s, 3H), 2.36 (dt, *J* = 14.0, 3.0 Hz, 1H), 2.06 – 2.01 (m, 1H), 1.91 (ddd, *J* = 12.9, 8.4, 4.6 Hz, 1H), 1.82 – 1.65 (m, 8H), 1.57 – 1.24 (m, 13H), 1.21 (d, *J* = 6.8 Hz, 3H), 0.94 (s, 9H).

^{13}C NMR (151 MHz, DMSO-d_6) δ (ppm): 174.83, 172.81, 171.96, 169.65, 169.10 (“d”, *J* = 2.7 Hz), 156.91 (“d”, *J* = 8.0 Hz), 151.91, 151.89, 151.48, 150.89, 150.56, 147.70, 146.63 (“d”, *J* = 16.4 Hz), 139.51, 135.61, 131.17, 129.63, 128.86, 128.64, 128.21 (“d”, *J* = 4.8 Hz), 127.43, 126.90 (“d”, *J* = 4.0 Hz), 125.94

(“d”, J = 5.6 Hz), 122.88, 106.26, 69.12 (“d”, J = 7.2 Hz), 68.87, 58.71, 56.20 (“d”, J = 42.5 Hz), 52.99, 45.12, 42.72, 42.46, 41.64, 41.44, 41.00 (“d”, J = 16.5 Hz), 40.43, 40.23 (“d”, J = 6.0 Hz), 38.51, 37.94, 36.92, 36.12 (“d”, J = 31.1 Hz), 35.44, 35.38, 34.90 (“d”, J = 16.4 Hz), 34.18 (“d”, J = 19.5 Hz), 29.97, 28.86, 28.33, 26.36, 21.99 (“d”, J = 29.9 Hz), 15.97, 15.94.

Synthesis of (2*S*,4*R*)-4-hydroxy-1-((*S*)-2-((1*r*,4*S*)-4-(7-(4-(5-((4-hydroxy-1-((*R*)-3-phenylbutanoyl)-piperidin-4-yl)methyl)-4-oxo-4,5-dihydro-1*H*-pyrazolo[3,4-*d*]pyrimidin-1-yl)phenyl)-2,7-diazaspiro[3.5]nonane-2-carbonyl)cyclohexane-1-carboxamido)-3,3-dimethylbutanoyl)-*N*-(4-(4-methylthiazol-5-yl)benzyl)pyrrolidine-2-carboxamide (NK230)

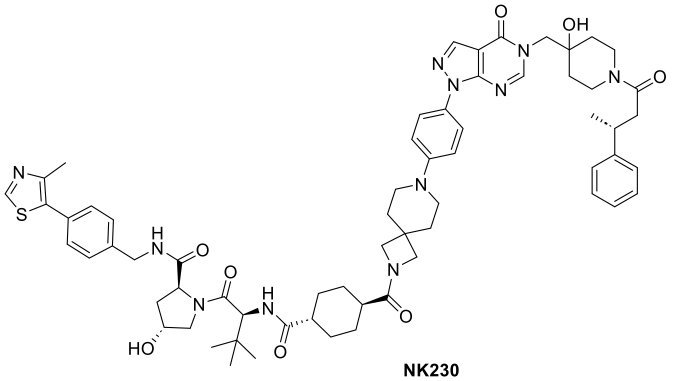

NK230

(2*S*,4*R*)-4-Hydroxy-1-((*S*)-2-((1*r*,4*S*)-4-(7-(4-(5-((4-hydroxy-1-((*R*)-3-phenylbutanoyl)-piperidin-4-yl)methyl)-4-oxo-4,5-dihydro-1*H*-pyrazolo[3,4-*d*]pyrimidin-1-yl)phenyl)-2,7-diazaspiro[3.5]nonane-2-carbonyl)-cyclohexane-1-carboxamido)-3,3-dimethylbutanoyl)-*N*-(4-(4-methylthiazol-5-yl)benzyl)pyrrolidine-2-carboxamide (NK230) was synthesized as described for NK239 (Supplementary Figure 15, exchanging (*R*)-5-((4-hydroxy-1-(3-phenylbutanoyl)piperidin-4-yl)methyl)-1-(4-(piperazin-1-yl)phenyl)-1,5-dihydro-4*H*-pyrazolo[3,4-*d*]pyrimidin-4-one (NK210) for (*R*)-1-(4-(2,7-diazaspiro[3.5]nonan-7-yl)phenyl)-5-((4-hydroxy-1-(3-phenylbutanoyl)piperidin-4-yl)methyl)-1,5-dihydro-4*H*-pyrazolo[3,4-*d*]pyrimidin-4-one TFA salt (NK220).

HRMS (m/z) $[M+2H]^{2+}$ calculated for $C_{64}H_{81}N_{11}O_8S^{2+}$: 581.7990, found: 581.7978.

^{1}H NMR (700 MHz, DMSO-d_6) δ (ppm): 8.99 (s, 1H), 8.56 (t, J = 6.1 Hz, 1H), 8.31 – 8.25 (m, 2H), 7.81 (d, J = 8.6 Hz, 2H), 7.74 (d, J = 9.3 Hz, 1H), 7.44 – 7.37 (m, 4H), 7.28 – 7.23 (m, 4H), 7.15 (ddd, J = 8.6, 6.3, 2.4 Hz, 3H), 4.52 (d, J = 9.4 Hz, 1H), 4.46 – 4.40 (m, 2H), 4.35 (tt, J = 4.5, 2.5 Hz, 2H), 4.22 (dd, J = 15.8, 5.5 Hz, 1H), 4.08 – 4.01 (m, 2H), 4.00 – 3.93 (m, 2H), 3.91 (s, 2H), 3.69 – 3.57 (m, 5H), 3.24 (d, J = 22.3 Hz, 4H), 3.19 – 3.14 (m, 1H), 2.90 – 2.83 (m, 1H), 2.64 – 2.52 (m, 2H), 2.45 (s, 3H), 2.39 – 2.33 (m, 1H), 2.20 – 2.14 (m, 1H), 2.06 – 2.00 (m, 1H), 1.90 (ddd, J = 12.9, 8.6, 4.6 Hz, 1H), 1.81 (dq, J = 18.8, 9.8, 7.7 Hz, 4H), 1.68 (q, J = 13.7, 11.1 Hz, 2H), 1.56 – 1.24 (m, 9H), 1.20 (dd, J = 6.9, 1.5 Hz, 3H), 0.93 (s, 9H).

^{13}C NMR (176 MHz, DMSO-d_6) δ (ppm): 174.83, 174.78, 171.94, 169.64, 169.10 (“d”, J = 3.1 Hz), 156.91 (“d”, J = 9.1 Hz), 151.88 (“d”, J = 3.3 Hz), 151.48, 150.54, 147.67, 146.63 (“d”, J = 19.2 Hz), 139.51, 135.58, 131.18, 129.62, 128.64, 128.20 (“d”, J = 5.6 Hz), 127.43, 126.89 (“d”, J = 4.6 Hz), 125.93 (“d”, J = 6.8 Hz), 122.87, 116.09, 114.72, 106.24, 69.11 (“d”, J = 8.6 Hz), 68.87, 59.12, 58.71, 56.90, 56.32, 56.07, 52.99, 42.52, 41.64, 41.00 (“d”, J = 19.0 Hz), 40.23 (“d”, J = 7.6 Hz), 38.20, 37.94, 36.91, 36.11 (“d”, J = 36.1 Hz), 35.41, 34.89 (“d”, J = 20.3 Hz), 34.04, 32.80, 29.13, 27.73, 27.45, 26.35, 21.98 (“d”, J = 34.4 Hz), 15.92.

3.9 Synthesis of VHL-cubane-functionalized PROTACs

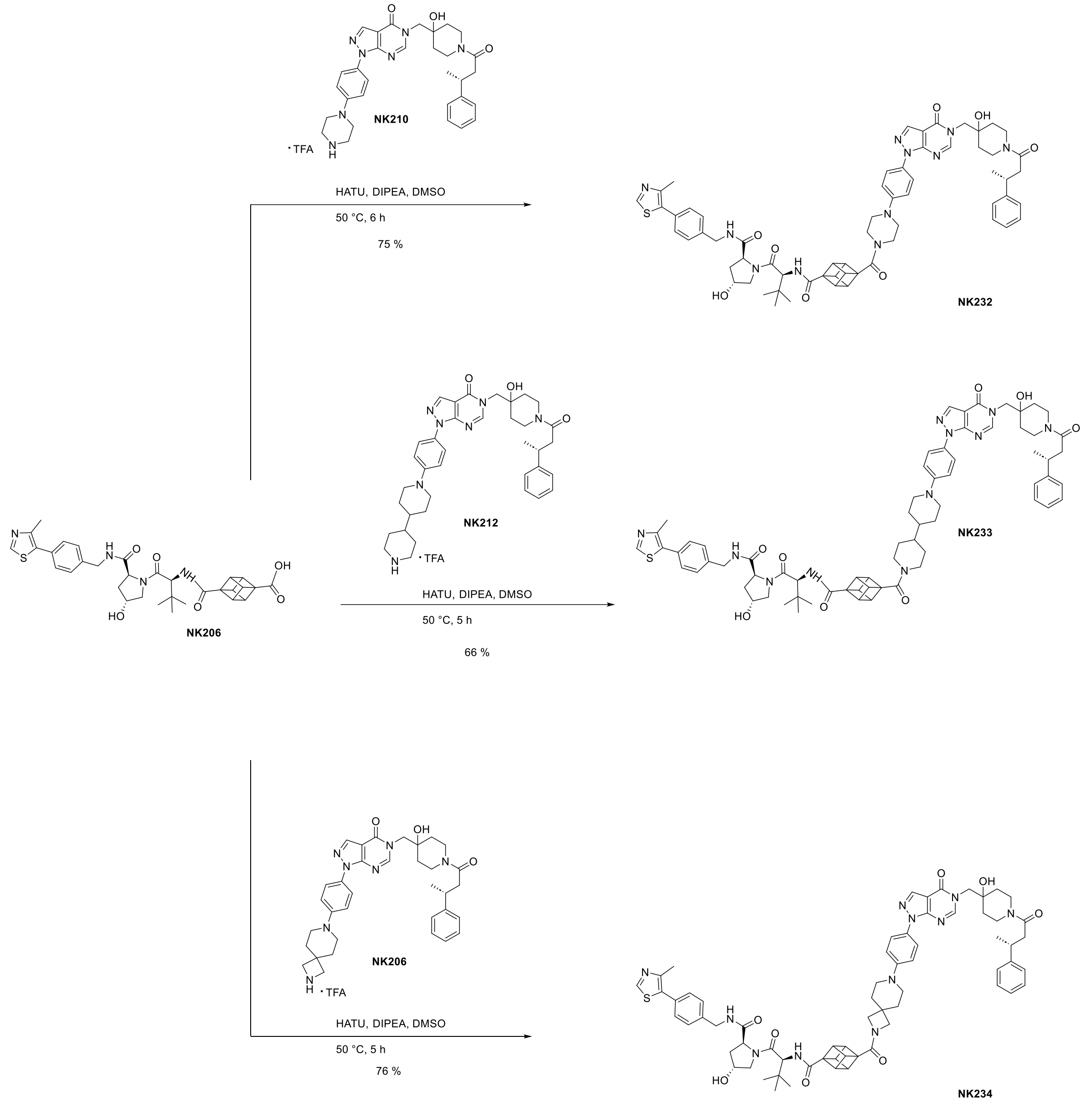

Supplementary Figure 16. Synthesis of VHL-cubane-functionalized PROTACs NK232, NK233 and NK234.

Synthesis of (2*S*,4*R*)-4-hydroxy-1-((*S*)-2-(4-(4-(4-(5-((4-hydroxy-1-((*R*)-3-phenylbutanoyl)-piperidin-4-yl)methyl)-4-oxo-4,5-dihydro-1*H*-pyrazolo[3,4-*d*]pyrimidin-1-yl)phenyl)piperazine-1-carbonyl)cubane-1-carboxamido)-3,3-dimethylbutanoyl)-*N*-(4-(4-methylthiazol-5-yl)benzyl)pyrrolidine-2-carboxamide (NK232)

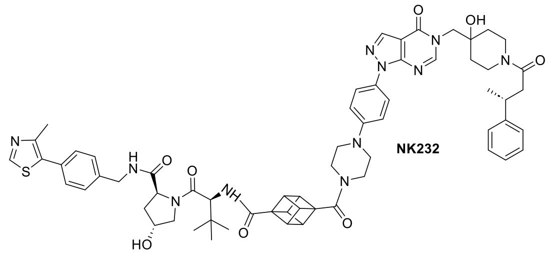

NK206 (12.6 mg, 0.02 mmol, 1.2 eq), HATU (7.4 mg, 0.02 mmol, 1.3 eq) and DIPEA (7.7 mg, 0.06 mmol, 4.0 eq) were dissolved in DMSO (1 mL) and stirred at 50 °C for 15 min. To this was added NK210 (10.0 mg, 0.01 mmol, 1.0 eq) and the mixture was stirred at 50 °C for 5 h. After completion, the crude product was directly purified by preparative reverse phase column chromatography (ACN/H_2O + 0.1 % TFA). After evaporation of the solvents, (2*S*,4*R*)-4-hydroxy-1-((*S*)-2-(4-(4-(4-(5-((4-hydroxy-1-((*R*)-3-

phenylbutanoyl)-piperidin-4-yl)methyl)-4-oxo-4,5-dihydro-1*H*-pyrazolo[3,4-*d*]pyrimidin-1-yl)phenyl)piperazine-1-carbonyl)cubane-1-carboxamido)-3,3-dimethylbutanoyl)-*N*-(4-(4-methylthiazol-5-yl)benzyl)pyrrolidine-2-carboxamide (NK232) (12.8 mg, 0.01 mmol, 75 % yield) was obtained as a white powder.

HRMS (m/z) $[M+2H]^{2+}$ calculated for $C_{63}H_{73}N_{11}O_8S^{2+}$: 571.7667, found: 571.7667.

^{1}H NMR (500 MHz, DMSO-d_6) δ (ppm): 8.93 (s, 1H), 8.50 (t, *J* = 6.1 Hz, 1H), 8.23 (s, 1H), 8.21 (d, *J* = 11.9 Hz, 1H), 7.76 (d, *J* = 9.1 Hz, 2H), 7.68 (d, *J* = 9.3 Hz, 1H), 7.38 – 7.30 (m, 4H), 7.19 (dd, *J* = 7.3, 5.6 Hz, 4H), 7.08 (ddt, *J* = 10.3, 8.0, 3.0 Hz, 3H), 4.55 (d, *J* = 9.4 Hz, 1H), 4.40 – 4.32 (m, 2H), 4.29 (tt, *J* = 4.4, 2.5 Hz, 1H), 4.16 (dd, *J* = 15.8, 5.6 Hz, 1H), 4.08 (s, 6H), 4.02 – 3.85 (m, 3H), 3.65 – 3.51 (m, 5H), 3.27 (dt, *J* = 38.2, 4.9 Hz, 4H), 3.13 (dt, *J* = 25.2, 5.9 Hz, 4H), 2.85 – 2.76 (m, 1H), 2.58 – 2.47 (m, 2H), 2.38 (s, 3H), 2.01 – 1.95 (m, 1H), 1.84 (ddd, *J* = 13.0, 8.7, 4.6 Hz, 1H), 1.38 – 1.16 (m, 4H), 1.14 (d, *J* = 6.9 Hz, 3H), 0.89 (s, 9H).

^{13}C NMR (126 MHz, DMSO-d_6) δ (ppm): 172.39, 170.77, 169.94, 169.58 (“d”, *J* = 1.8 Hz), 169.13, 157.39 (“d”, *J* = 6.8 Hz), 152.39, 152.00, 151.04 (“d”), 150.08, 148.13, 147.11 (“d”, *J* = 13.7 Hz), 139.99, 136.09, 131.68, 130.47, 130.10, 129.14, 128.68 (“d”, *J* = 4.1 Hz), 127.92, 127.37 (“d”, *J* = 3.3 Hz), 126.42 (“d”, *J* = 4.7 Hz), 123.32, 116.35, 106.75, 69.59 (“d”, *J* = 6.0 Hz), 69.35, 59.23, 57.43, 56.90, 56.67, 56.48, 53.47, 49.06, 48.52, 46.49, 44.50, 42.14, 41.48 (“d”, *J* = 13.5 Hz), 41.26, 40.71 (“d”, *J* = 4.8 Hz), 38.43, 37.40, 36.59 (“d”, *J* = 26.0 Hz), 36.00, 35.37 (“d”, *J* = 15.0 Hz), 34.65 (“d”, *J* = 17.9 Hz), 26.92, 22.46 (“d”, *J* = 25.0 Hz), 16.39.

Synthesis of (2*S*,4*R*)-4-hydroxy-1-((*S*)-2-(4-(1'-(4-(5-((4-hydroxy-1-((*R*)-3-phenylbutanoyl)-piperidin-4-yl)methyl)-4-oxo-4,5-dihydro-1*H*-pyrazolo[3,4-*d*]pyrimidin-1-yl)phenyl)-[4,4'-bipiperidine]-1-carbonyl)cubane-1-carboxamido)-3,3-dimethylbutanoyl)-*N*-(4-(4-methylthiazol-5-yl)benzyl)pyrrolidine-2-carboxamide (NK233)

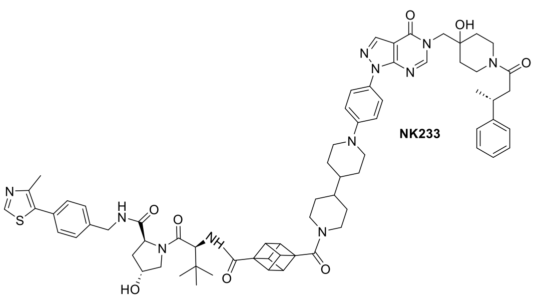

(2*S*,4*R*)-4-Hydroxy-1-((*S*)-2-(4-(1'-(4-(5-((4-hydroxy-1-((*R*)-3-phenylbutanoyl)-piperidin-4-yl)methyl)-4-oxo-4,5-dihydro-1*H*-pyrazolo[3,4-*d*]pyrimidin-1-yl)phenyl)-[4,4'-bipiperidine]-1-carbonyl)cubane-1-carboxamido)-3,3-dimethylbutanoyl)-*N*-(4-(4-methylthiazol-5-yl)benzyl)pyrrolidine-2-carboxamide (NK233) was synthesized as described for NK232 (Supplementary Figure 16, exchanging (*R*)-5-((4-hydroxy-1-(3-phenylbutanoyl)piperidin-4-yl)methyl)-1-(4-(piperazin-1-yl)phenyl)-1,5-dihydro-4*H*-pyrazolo[3,4-*d*]pyrimidin-4-one TFA salt (NK210) for (*R*)-1-(4-([4,4'-bipiperidin]-1-yl)phenyl)-5-((4-hydroxy-1-(3-phenylbutanoyl)piperidin-4-yl)methyl)-1,5-dihydro-4*H*-pyrazolo[3,4-*d*]pyrimidin-4-one TFA salt (NK212)).

HRMS (m/z) $[M+2H]^{2+}$ calculated for $C_{69}H_{83}N_{11}O_8S^{2+}$: 612.8068, found: 612.8059.

^{1}H NMR (600 MHz, DMSO-d_6) δ (ppm): 8.99 (s, 1H), 8.58 (t, *J* = 6.1 Hz, 1H), 8.30 (s, 1H), 8.28 (d, *J* = 14.2 Hz, 1H), 7.81 (d, *J* = 8.5 Hz, 2H), 7.70 (dd, *J* = 11.7, 9.5 Hz, 1H), 7.43 – 7.38 (m, 4H), 7.30 – 7.24 (m, 4H), 7.16 (ddd, *J* = 8.7, 6.3, 2.5 Hz, 3H), 4.61 (d, *J* = 9.3 Hz, 1H), 4.47 – 4.40 (m, 2H), 4.38 – 4.33 (m, 2H), 4.23 (dd, *J* = 15.8, 5.4 Hz, 1H), 4.15 – 4.07 (m, 6H), 4.06 – 3.97 (m, 2H), 3.94 (d, *J* = 4.1 Hz, 1H), 3.82 (d, *J* = 11.9 Hz, 2H), 3.70 – 3.62 (m, 4H), 3.23 – 3.14 (m, 3H), 3.04 (t, *J* = 12.5 Hz, 1H), 2.87 (t, *J* = 13.7 Hz, 1H), 2.75 (s, 1H), 2.66 – 2.57 (m, 2H), 2.45 (s, 3H), 2.09 – 1.96 (m, 2H), 1.91 (ddd, *J* = 12.9, 8.8, 4.6 Hz, 1H), 1.80 (s, 3H), 1.74 (d, *J* = 12.3 Hz, 1H), 1.44 – 1.23 (m, 10H), 1.21 (dd, *J* = 7.0, 1.5 Hz, 3H), 0.95 (s, 9H).

^{13}C NMR (151 MHz, DMSO-d_6) δ (ppm): 171.93, 170.34, 169.47, 169.12, 168.19, 158.05 (“d”, *J* = 35.0 Hz), 151.91, 151.50, 150.80, 150.55, 147.72, 146.59, 139.50, 135.51, 132.35, 131.18, 129.65, 128.68, 128.22 (“d”, *J* = 4.8 Hz), 127.45, 126.91 (“d”, *J* = 4.1 Hz), 125.96 (“d”, *J* = 6.1 Hz), 122.90, 117.01, 106.25, 69.12 (“d”, *J* = 7.5 Hz), 68.88, 58.76, 57.21, 56.43, 56.18, 56.00, 53.01, 45.97 (“d”, *J* = 11.2 Hz), 41.67, 41.48 (“d”, *J* = 4.2 Hz), 40.44, 40.24 (“d”, *J* = 5.5 Hz), 37.95, 36.93, 36.13 (“d”, *J* = 31.6 Hz),

35.53, 35.04 (“d”, *J* = 21.7 Hz), 34.19 (“d”, *J* = 20.5 Hz), 33.41, 29.99, 29.71, 29.60, 28.69, 28.38, 26.44, 22.00 (“d”, *J* = 29.7 Hz), 15.95.

Synthesis of (2*S*,4*R*)-4-hydroxy-1-((*S*)-2-(4-(7-(4-(5-((4-hydroxy-1-((*R*)-3-phenylbutanoyl)-piperidin-4-yl)methyl)-4-oxo-4,5-dihydro-1*H*-pyrazolo[3,4-*d*]pyrimidin-1-yl)phenyl)-2,7-diazaspiro[3.5]nonane-2-carbonyl)cubane-1-carboxamido)-3,3-dimethylbutanoyl)-*N*-(4-(4-methylthiazol-5-yl)benzyl)pyrrolidine-2-carboxamide (NK234)

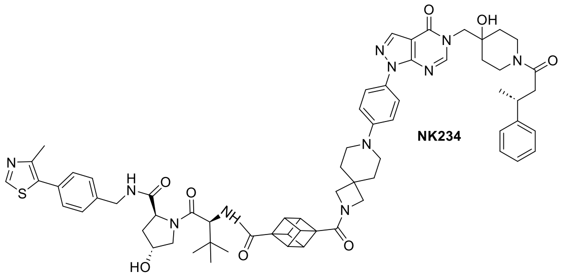

(2*S*,4*R*)-4-Hydroxy-1-((*S*)-2-(4-(7-(4-(5-((4-hydroxy-1-((*R*)-3-phenylbutanoyl)-piperidin-4-yl)methyl)-4-oxo-4,5-dihydro-1*H*-pyrazolo[3,4-*d*]pyrimidin-1-yl)phenyl)-2,7-diazaspiro[3.5]nonane-2-carbonyl)cubane-1-carboxamido)-3,3-dimethylbutanoyl)-*N*-(4-(4-methylthiazol-5-yl)benzyl)pyrrolidine-2-carboxamide (NK234) was synthesized as described for NK232 (Supplementary Figure 16, exchanging (*R*)-5-((4-hydroxy-1-(3-phenylbutanoyl)piperidin-4-yl)methyl)-1-(4-(piperazin-1-yl)phenyl)-1,5-dihydro-4*H*-pyrazolo[3,4-*d*]pyrimidin-4-one TFA salt (NK210) for (*R*)-1-(4-(2,7-diazaspiro[3.5]nonan-7-yl)phenyl)-5-((4-hydroxy-1-(3-phenylbutanoyl)piperidin-4-yl)methyl)-1,5-dihydro-4*H*-pyrazolo[3,4-*d*]pyrimidin-4-one TFA salt (NK220)).

HRMS (m/z) $[M+2H]^{2+}$ calculated for $C_{66}H_{77}N_{11}O_8S^{2+}$: 591.7833, found: 591.7835.

^{1}H NMR (700 MHz, DMSO-d_6) δ (ppm): 8.99 (s, 1H), 8.56 (t, *J* = 6.1 Hz, 1H), 8.29 (d, *J* = 1.0 Hz, 1H), 8.28 (d, *J* = 16.5 Hz, 1H), 7.82 (d, *J* = 8.6 Hz, 2H), 7.65 (d, *J* = 9.4 Hz, 1H), 7.42 – 7.38 (m, 4H), 7.28 – 7.23 (m, 4H), 7.19 – 7.14 (m, 3H), 4.59 (d, *J* = 9.4 Hz, 1H), 4.44 – 4.40 (m, 2H), 4.35 (tt, *J* = 4.3, 2.4 Hz, 1H), 4.22 (dd, *J* = 15.8, 5.5 Hz, 1H), 4.16 (dd, *J* = 5.7, 4.1 Hz, 3H), 4.11 (dd, *J* = 5.7, 4.1 Hz, 3H), 4.07 – 3.93 (m, 3H), 3.88 (s, 2H), 3.69 – 3.61 (m, 5H), 3.25 (s, 4H), 3.22 – 3.13 (m, 2H), 2.90 – 2.84 (m, 2H), 2.63 – 2.52 (m, 2H), 2.44 (s, 3H), 2.07 – 2.02 (m, 1H), 1.90 (ddd, *J* = 12.9, 8.7, 4.6 Hz, 1H), 1.87 – 1.84 (m, 4H), 1.56 – 1.24 (m, 4H), 1.20 (dd, *J* = 7.0, 1.6 Hz, 3H), 0.94 (s, 9H).

^{13}C NMR (176 MHz, DMSO-d_6) δ (ppm): 171.90, 170.50, 170.28, 169.45, 169.10 (“d”, *J* = 3.1 Hz), 156.91 (“d”, *J* = 9.4 Hz), 151.91, 151.50, 150.56, 148.54, 147.66, 146.63 (“d”, *J* = 19.1 Hz), 139.50, 135.61, 131.18, 129.63, 128.66, 128.20 (“d”, *J* = 5.8 Hz), 127.44, 126.89 (“d”, *J* = 4.8 Hz), 126.31, 125.94 (“d”, *J* = 6.2 Hz), 122.84, 116.24, 106.27, 69.12 (“d”, *J* = 8.4 Hz), 68.87, 58.74, 58.62, 57.91, 56.71, 56.41, 56.15, 56.08, 53.00, 46.30, 45.65, 41.66, 41.00 (“d”, *J* = 19.7 Hz), 40.43, 40.23 (“d”, *J* = 7.3 Hz), 37.94, 36.92, 36.11 (“d”, *J* = 36.2 Hz), 35.51, 34.90 (“d”, *J* = 19.5 Hz), 34.06 (“d”, *J* = 10.6 Hz), 26.42, 21.98 (“d”, *J* = 34.7 Hz), 15.91.

3.10 Synthesis of enhanced PROTACs NK250 and NK266

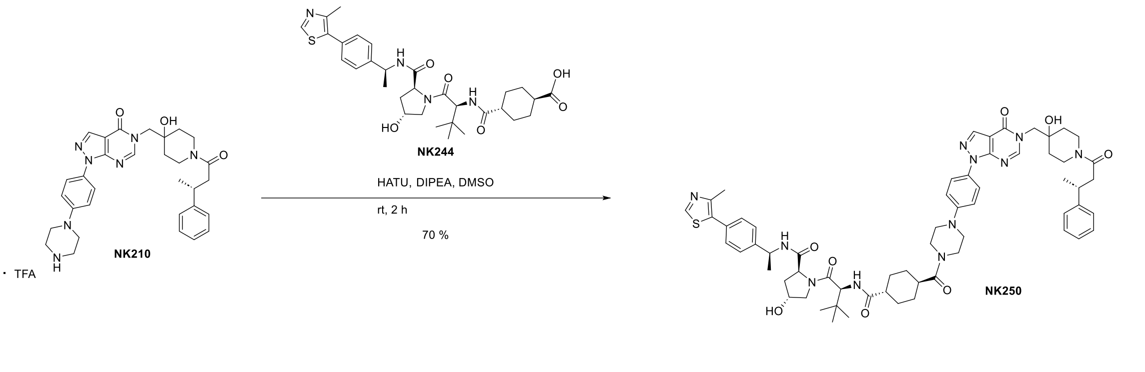

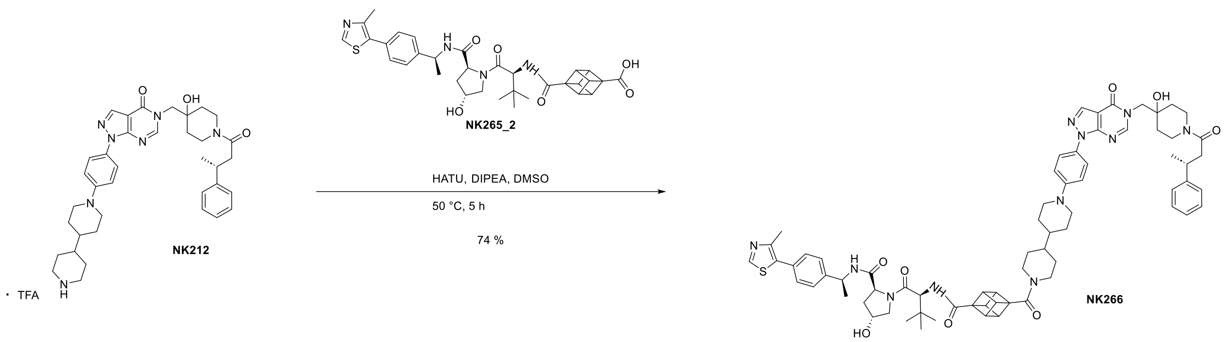

Supplementary Figure 17. Synthesis of enhanced PROTACs NK250 and NK266.

Synthesis of (2*S*,4*R*)-4-hydroxy-1-((*S*)-2-((1*r*,4*S*)-4-(4-(4-(5-((4-hydroxy-1-((*R*)-3-phenylbutanoyl)-piperidin-4-yl)methyl)-4-oxo-4,5-dihydro-1*H*-pyrazolo[3,4-*d*]pyrimidin-1-yl)phenyl)piperazine-1-carbonyl)cyclohexane-1-carboxamido)-3,3-dimethylbutanoyl)-*N*-((*S*)-1-(4-(4-methylthiazol-5-yl)phenyl)ethyl)pyrrolidine-2-carboxamide (NK250)

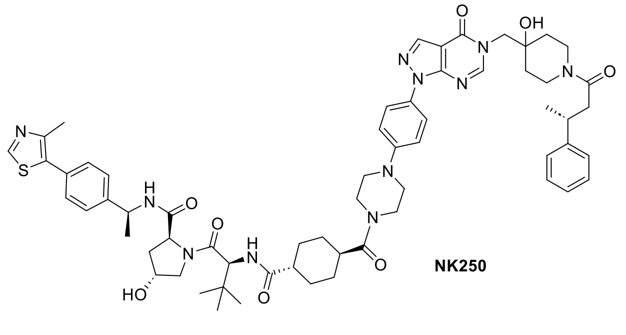

NK244 (19.7 mg, 0.03 mmol, 1.1 eq), HATU (14.8 mg, 0.04 mmol, 1.3 eq) and DIPEA (15.4 mg, 0.04 mmol, 4 eq) were dissolved in DMSO (1 mL) and stirred at rt for 20 min. To this was added NK210 (20.0 mg, 0.03 mmol, 1 eq) and the reaction was stirred for 2 h at rt. After completion, the crude product was directly purified by preparative reverse phase column chromatography (ACN/H_2O + 0.1 % TFA). After evaporation of the solvents, (2*S*,4*R*)-4-Hydroxy-1-((*S*)-2-((1*r*,4*S*)-4-(4-(4-(5-((4-hydroxy-1-((*R*)-3-phenylbutanoyl)piperidin-4-yl)methyl)-4-oxo-4,5-dihydro-1*H*-pyrazolo[3,4-*d*]pyrimidin-1-yl)phenyl)piperazine-1-carbonyl)-cyclohexane-1-carboxamido)-3,3-dimethylbutanoyl)-*N*-((*S*)-1-(4-(4-methylthiazol-5-yl)phenyl)ethyl)pyrrolidine-2-carboxamide (NK250) (23.8 mg, 0.02 mmol, 70 % yield) was obtained as a white powder.

HRMS (m/z) $[M+2H]^{2+}$ calculated for $C_{62}H_{79}N_{11}O_8S^{2+}$: 568.7912, found: 568.7909.

^{1}H NMR (600 MHz, DMSO-d_6) δ (ppm): 8.99 (s, 1H), 8.38 (d, *J* = 7.8 Hz, 1H), 8.30 (s, 1H), 8.28 (d, *J* = 14.2 Hz, 1H), 7.82 (d, *J* = 9.0 Hz, 2H), 7.69 (d, *J* = 9.3 Hz, 1H), 7.43 (d, *J* = 8.2 Hz, 2H), 7.38 (d, *J* = 8.3 Hz, 2H), 7.28 – 7.23 (m, 4H), 7.17 – 7.11 (m, 3H), 4.92 (t, *J* = 7.2 Hz, 1H), 4.50 (d, *J* = 9.3 Hz, 1H), 4.42 (t, *J* = 8.1 Hz, 1H), 4.28 (tt, *J* = 4.5, 2.5 Hz, 1H), 4.07 – 3.97 (m, 2H), 3.94 (d, *J* = 3.6 Hz, 1H), 3.70 – 3.60 (m, 6H), 3.57 (d, *J* = 10.8 Hz, 1H), 3.25 – 3.15 (m, 6H), 2.86 (q, *J* = 8.4, 5.1 Hz, 1H), 2.66 – 2.57

(m, 2H), 2.45 (s, 3H), 2.03 – 1.98 (m, 1H), 1.79 (ddd, *J* = 12.8, 8.4, 4.5 Hz, 2H), 1.70 (q, *J* = 14.8, 11.8 Hz, 3H), 1.57 – 1.29 (m, 13H), 1.20 (dd, *J* = 7.0, 1.3 Hz, 3H), 0.94 (s, 9H).

^{13}C NMR (151 MHz, DMSO-d_6) δ (ppm): 174.83, 172.81, 171.96, 169.65, 169.10 (“d”, *J* = 2.7 Hz), 156.91 (“d”, *J* = 8.0 Hz), 151.90 (“d”, *J* = 3.3 Hz), 151.48, 150.89, 150.57 (“d”, *J* = 2.4 Hz), 147.70, 146.63 (“d”, *J* = 16.4 Hz), 139.51, 135.61, 131.17, 129.63, 128.86, 128.64, 128.21 (“d”, *J* = 4.8 Hz), 128.08, 127.43, 126.90 (“d”, *J* = 4.0 Hz), 125.94 (“d”, *J* = 5.6 Hz), 122.88, 106.26, 69.12 (“d”, *J* = 7.2 Hz), 68.87, 58.71, 56.20 (“d”, *J* = 42.5 Hz), 52.99, 45.12, 42.72, 42.46, 41.64, 41.44, 41.00 (“d”, *J* = 16.5 Hz), 40.43, 40.23 (“d”, *J* = 6.0 Hz), 38.51, 37.94, 36.92, 36.12 (“d”, *J* = 31.1 Hz), 35.44, 35.38, 34.90 (“d”, *J* = 16.4 Hz), 34.18 (“d”, *J* = 19.5 Hz), 29.97, 28.86, 28.33, 26.36, 21.99 (“d”, *J* = 29.9 Hz), 15.97, 15.94.

Synthesis of (2*S*,4*R*)-4-hydroxy-1-((*S*)-2-(4-(1'-(4-(5-((4-hydroxy-1-((*R*)-3-phenylbutanoyl)-piperidin-4-yl)methyl)-4-oxo-4,5-dihydro-1*H*-pyrazolo[3,4-*d*]pyrimidin-1-yl)phenyl)-[4,4'-bipiperidine]-1-carbonyl)cubane-1-carboxamido)-3,3-dimethylbutanoyl)-*N*-((*S*)-1-(4-(4-methylthiazol-5-yl)phenyl)ethyl)pyrrolidine-2-carboxamide (NK266)

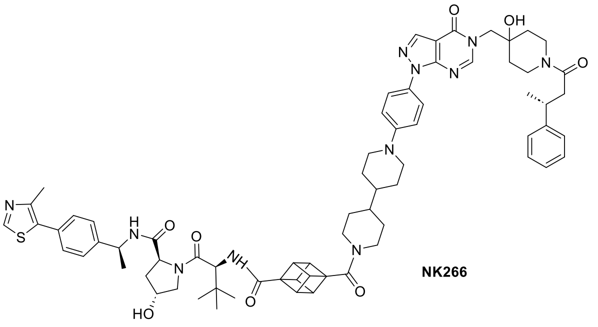

NK266

NK265_2 (8.8 mg, 0.01 mmol, 1.1 eq), HATU (6.6 mg, 0.02 mmol, 1.3 eq) and DIPEA (8.6 mg, 0.06 mmol, 5.0 eq) were dissolved in DMSO (1 mL) and stirred at 50 °C for 15 min. To this was added NK212 (10.0 mg, 0.01 mmol, 1.0 eq) and the mixture was stirred at 50 °C for 5 h. After completion, the crude product was directly purified by preparative reverse phase column chromatography (ACN/H_2O + 0.1 % TFA). After evaporation of the solvents, (2*S*,4*R*)-4-hydroxy-1-((*S*)-2-(4-(1'-(4-(5-((4-hydroxy-1-((*R*)-3-phenylbutanoyl)-piperidin-4-yl)methyl)-4-oxo-4,5-dihydro-1*H*-pyrazolo[3,4-*d*]pyrimidin-1-yl)phenyl)-[4,4'-bipiperidine]-1-carbonyl)cubane-1-carboxamido)-3,3-dimethylbutanoyl)-*N*-((*S*)-1-(4-(4-methylthiazol-5-yl)phenyl)ethyl)pyrrolidine-2-carboxamide (NK266) (12.2 mg, 0.01 mmol, 74 % yield) was obtained as an off-white solid.

HRMS (m/z) $[M+2H]^{2+}$ calculated for $C_{70}H_{85}N_{11}O_8S^{2+}$: 619.8146, found: 619.8140.

^{1}H NMR (600 MHz, DMSO-d_6) δ (ppm): 8.98 (s, 1H), 8.39 (d, *J* = 7.8 Hz, 1H), 8.30 – 8.25 (m, 2H), 7.77 (d, *J* = 8.5 Hz, 2H), 7.58 (dd, *J* = 11.3, 9.3 Hz, 1H), 7.43 (d, *J* = 8.3 Hz, 2H), 7.38 (d, *J* = 8.3 Hz, 2H), 7.26 (dd, *J* = 8.6, 6.1 Hz, 4H), 7.15 (ddd, *J* = 8.6, 6.2, 2.6 Hz, 1H), 7.10 (d, *J* = 8.5 Hz, 2H), 4.95 – 4.86 (m, 2H), 4.57 (d, *J* = 9.2 Hz, 1H), 4.43 (t, *J* = 8.1 Hz, 1H), 4.35 (d, *J* = 12.6 Hz, 1H), 4.31 – 4.26 (m, 1H), 4.13 – 4.07 (m, 6H), 4.07 – 3.92 (m, 3H), 3.81 (d, *J* = 11.8 Hz, 2H), 3.69 – 3.56 (m, 3H), 3.44 (d, *J* = 11.4 Hz, 1H), 3.23 – 3.13 (m, 2H), 3.03 (t, *J* = 12.6 Hz, 1H), 2.86 (q, *J* = 8.6, 5.4 Hz, 1H), 2.69 (s, 2H), 2.65 – 2.51 (m, 2H), 2.45 (s, 3H), 2.05 – 2.00 (m, 1H), 1.79 (td, *J* = 8.0, 4.3 Hz, 4H), 1.73 (d, *J* = 12.4 Hz, 1H), 1.57 – 1.39 (m, 2H), 1.37 (d, *J* = 7.0 Hz, 3H), 1.33 – 1.22 (m, 6H), 1.20 (dd, *J* = 7.0, 1.4 Hz, 3H), 1.16 – 1.01 (m, 2H), 0.95 (s, 9H).

^{13}C NMR (151 MHz, DMSO-d_6) δ (ppm): 171.06, 170.71, 169.82, 169.58 (“d”, *J* = 2.8 Hz), 168.63, 157.40 (“d”, *J* = 7.8 Hz), 152.31, 151.96, 150.96, 148.22, 147.11 (“d”, *J* = 16.4 Hz), 145.08, 135.98, 131.58, 130.17, 129.30, 128.68 (“d”, *J* = 4.8 Hz), 127.37 (“d”, *J* = 3.8 Hz), 126.87, 126.41 (“d”, *J* = 5.6 Hz), 123.37, 116.07, 106.67, 69.59 (“d”, *J* = 7.1 Hz), 69.25, 59.12, 57.73, 56.82, 56.67, 56.47, 53.46, 49.32, 48.13, 46.41 (“d”, *J* = 15.4 Hz), 45.15, 41.94, 41.47 (“d”, *J* = 16.7 Hz), 40.94, 40.71 (“d”, *J* = 6.0 Hz), 38.18, 37.39, 36.59 (“d”, *J* = 31.2 Hz), 36.03, 35.37 (“d”, *J* = 17.1 Hz), 34.65 (“d”, *J* = 20.2 Hz), 30.18, 29.02 (“d”, *J* = 36.3 Hz), 26.97, 22.90, 22.46 (“d”, *J* = 29.8 Hz), 16.45.

3.11 Synthesis of a VHL inhibitor

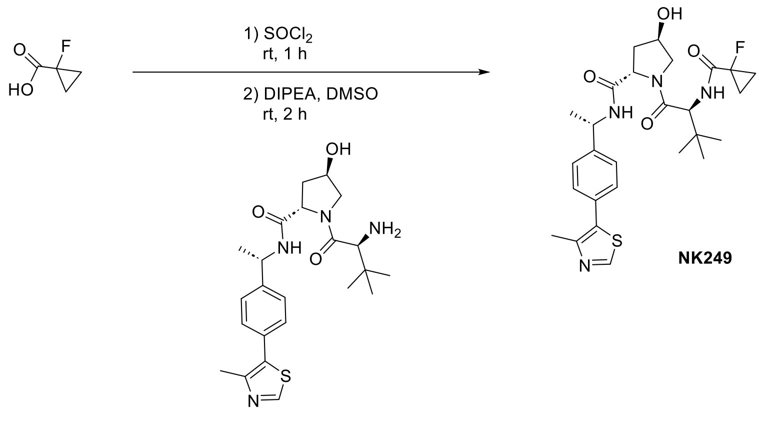

76 %

Supplementary Figure 18. Synthesis of VHL inhibitor NK249.

Synthesis of (2*S*,4*R*)-1-((*S*)-2-(1-fluorocyclopropane-1-carboxamido)-3,3-dimethylbutanoyl)-4-hydroxy-*N*-((*S*)-1-(4-(4-methylthiazol-5-yl)phenyl)ethyl)pyrrolidine-2-carboxamide (NK249)

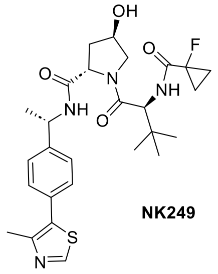

NK249

1-Fluorocyclopropane-1-carboxylic acid (5.6 mg, 0.05 mmol, 1.3 eq), was dissolved in $SOCl_2$ (1 mL) and stirred at rt for 1 h. After completion, the solvent was evaporated under reduced pressure. The crude product was dissolved in DMSO (1 mL) and to this were added (2*S*,4*R*)-1-((*S*)-2-amino-3,3-dimethylbutanoyl)-4-hydroxy-*N*-((*S*)-1-(4-(4-methylthiazol-5-yl)phenyl)ethyl)-pyrrolidine-2-carboxamide HCl salt (20.0 mg, 0.04 mmol, 1.0 eq) and DIPEA (58.1 mg, 0.45 mmol, 10.0 eq), and the mixture was stirred at rt for 2 h. After completion, the crude product was directly purified by preparative reverse phase column chromatography (ACN/H_2O + 0.1 % TFA). After evaporation of the solvents under reduced pressure, the (2*S*,4*R*)-1-((*S*)-2-(1-fluorocyclopropane-1-carboxamido)-3,3-dimethylbutanoyl)-4-hydroxy-*N*-((*S*)-1-(4-(4-methylthiazol-5-yl)phenyl)ethyl)pyrrolidine-2-carboxamide (NK249) (18.2 mg, 0.03 mmol, 76 % yield) was obtained as a white powder.

HRMS (m/z) $[M+H]^+$ calculated for $C_{27}H_{36}FN_4O_4S^+$: 531.2436, found: 531.2442.

^{1}H NMR (500 MHz, DMSO-d_6) δ (ppm): 8.99 (s, 1H), 8.47 (d, *J* = 7.8 Hz, 1H), 7.46 – 7.42 (m, 2H), 7.37 (d, *J* = 8.4 Hz, 2H), 7.25 (dd, *J* = 9.2, 2.9 Hz, 1H), 4.91 (p, *J* = 7.1 Hz, 1H), 4.58 (dd, *J* = 9.3, 1.2 Hz, 1H), 4.48 (dd, *J* = 8.9, 7.8 Hz, 1H), 4.28 (dt, *J* = 4.1, 2.0 Hz, 1H), 3.62 – 3.54 (m, 2H), 2.46 (s, 3H), 2.07 (ddt, *J* = 12.6, 7.8, 1.8 Hz, 1H), 1.77 (ddd, *J* = 13.2, 9.0, 4.5 Hz, 1H), 1.40 – 1.33 (m, 5H), 1.23 – 1.20 (m, 2H), 0.97 (s, 9H).

^{13}C NMR (126 MHz, DMSO-d_6) δ (ppm): 170.86, 169.23, 168.47 (d, *J* = 20.4 Hz), 151.98, 148.19, 145.20, 131.61, 130.15, 129.32, 126.81, 78.61 (d, *J* = 232.5 Hz), 69.28, 59.11, 57.11, 57.03, 48.23, 38.19, 36.58, 26.70, 22.97, 16.45, 13.44 (d, *J* = 10.1 Hz), 13.16 (d, *J* = 10.1 Hz).

4. NMR spectra of compounds

^{1}H NMR spectra of NK188.

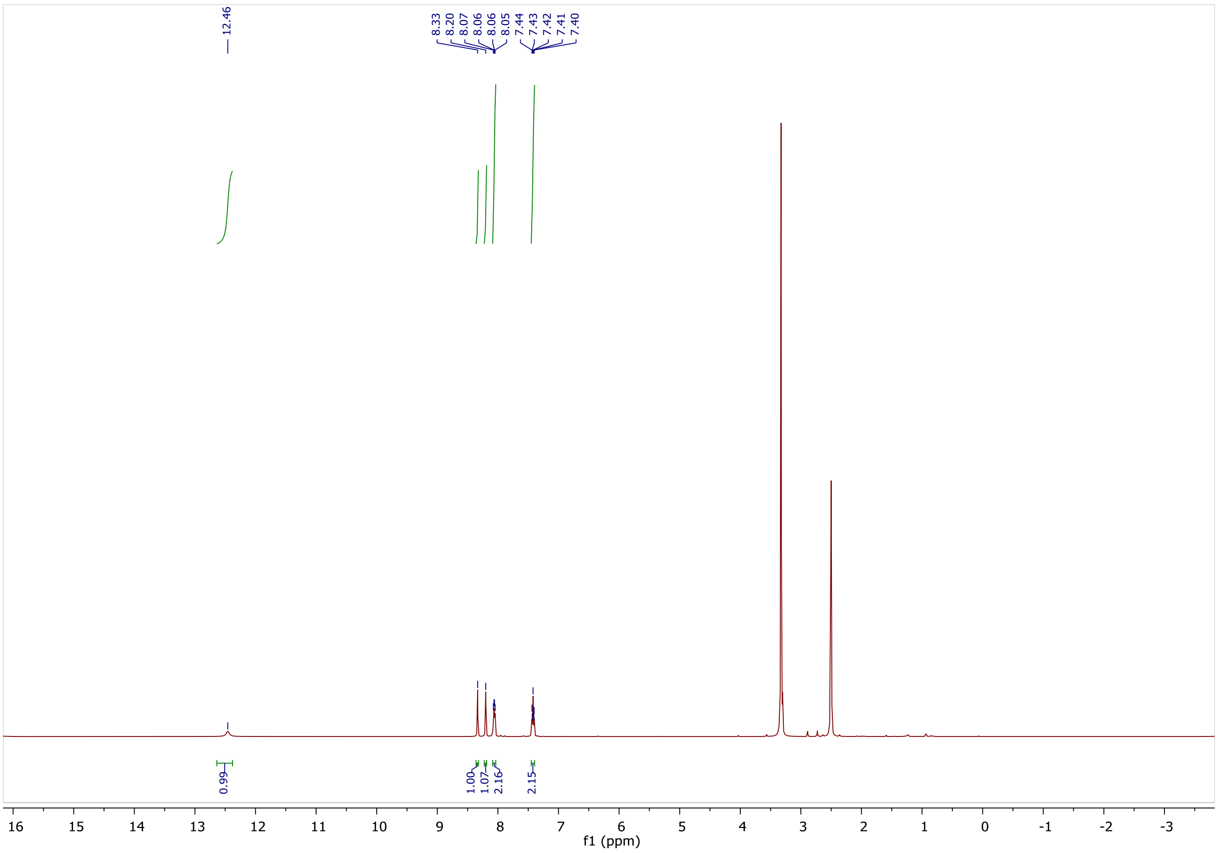

^{13}C NMR spectra of NK188.

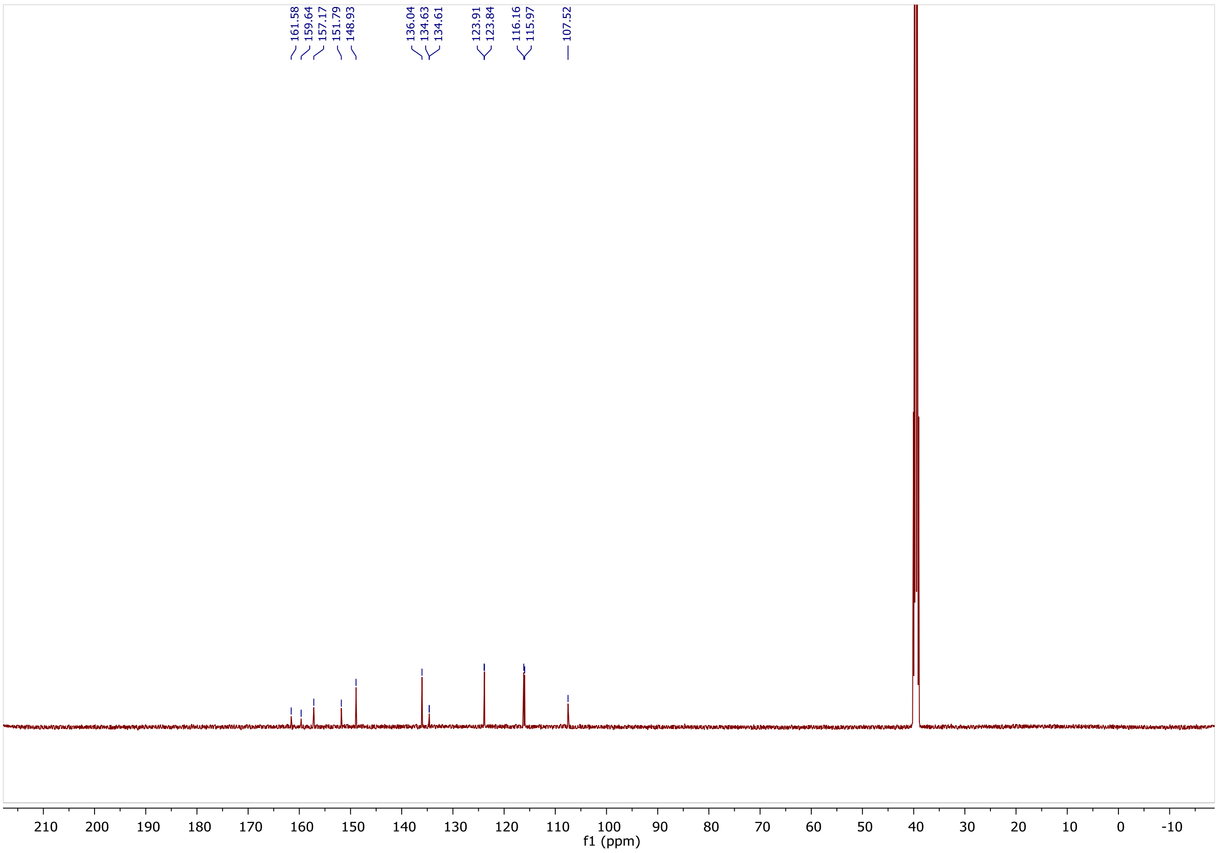

^{1}H NMR spectra of NK189.

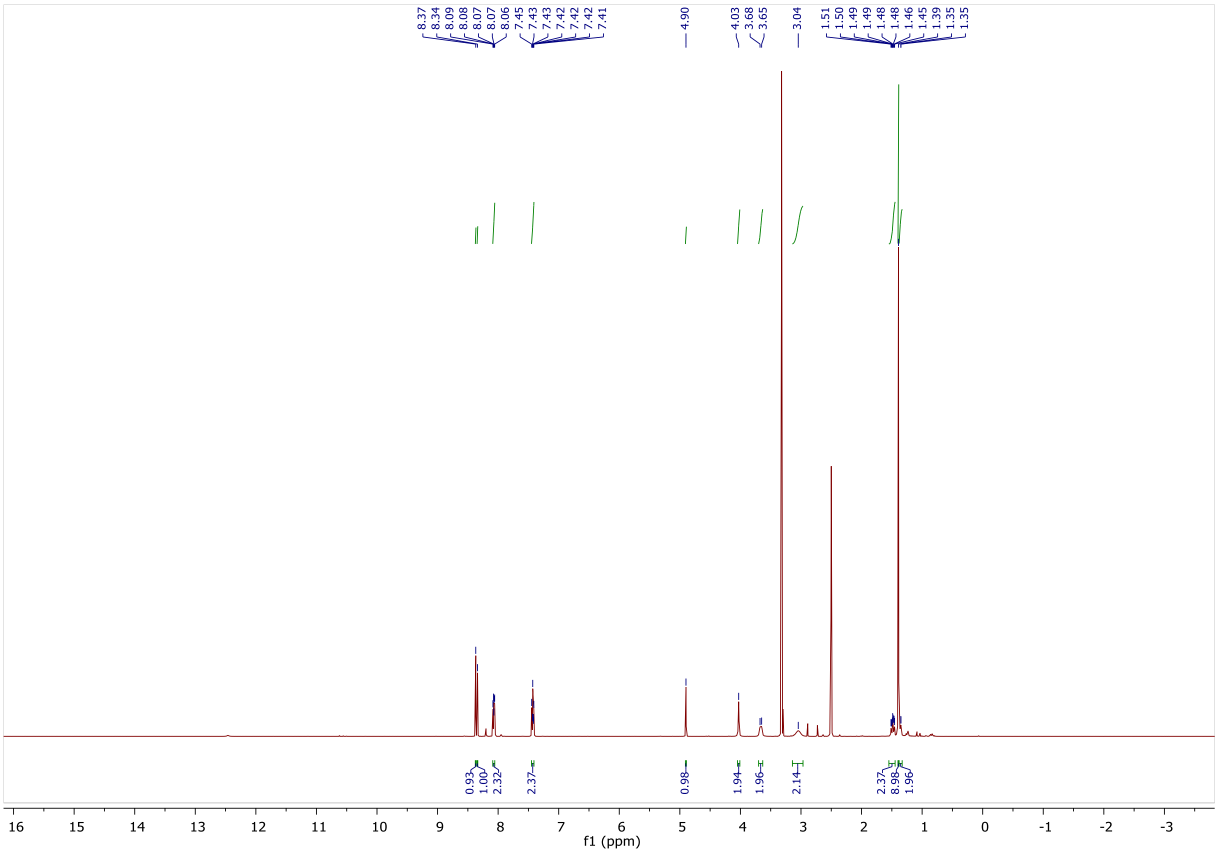

^{13}C NMR spectra of NK189.

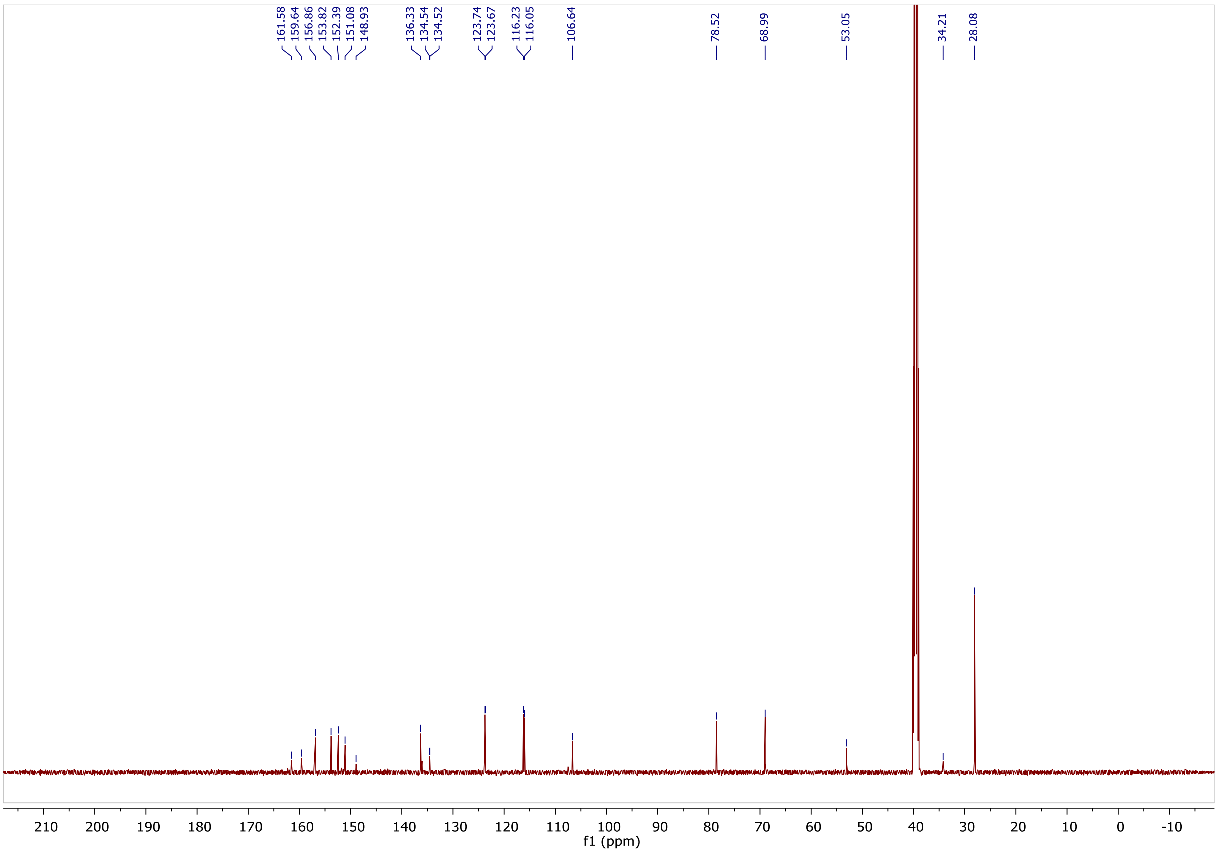

^{1}H NMR spectra of NK192.

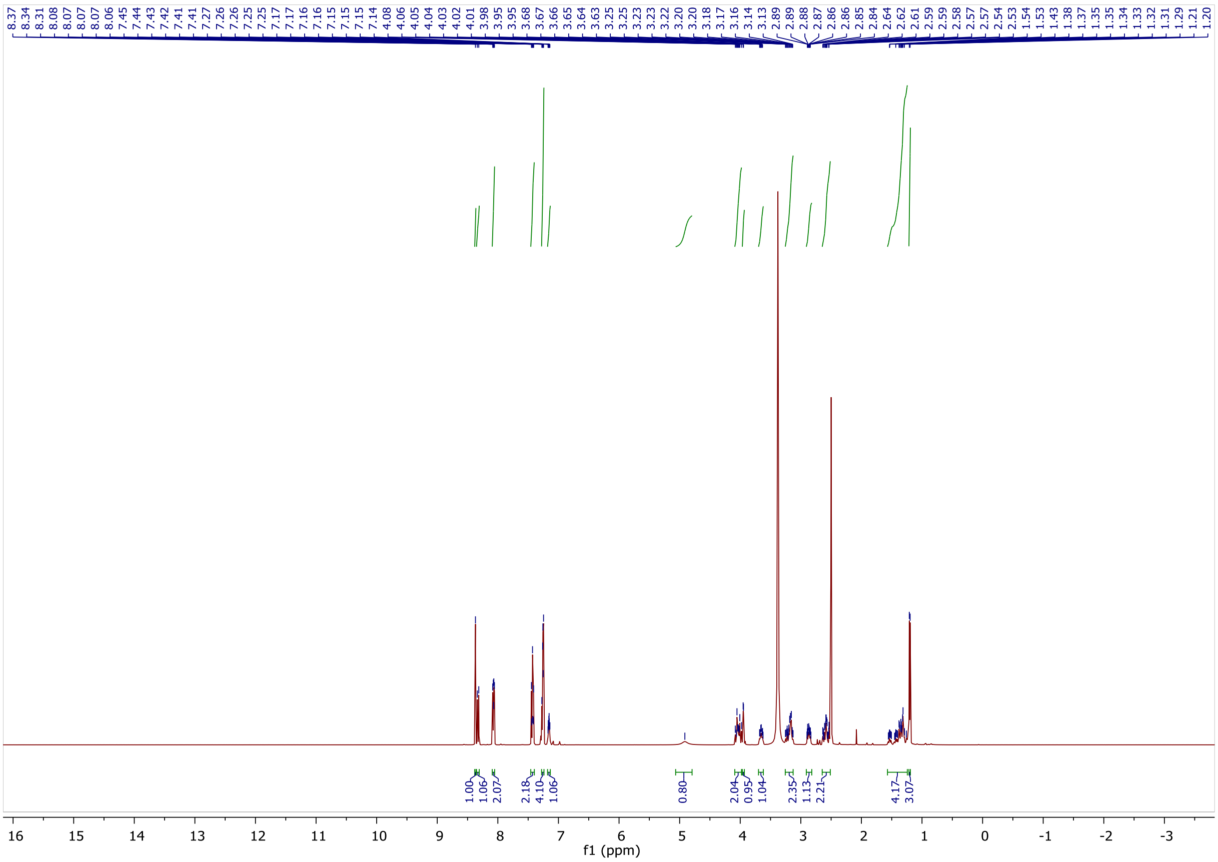

^{13}C NMR spectra of NK192.

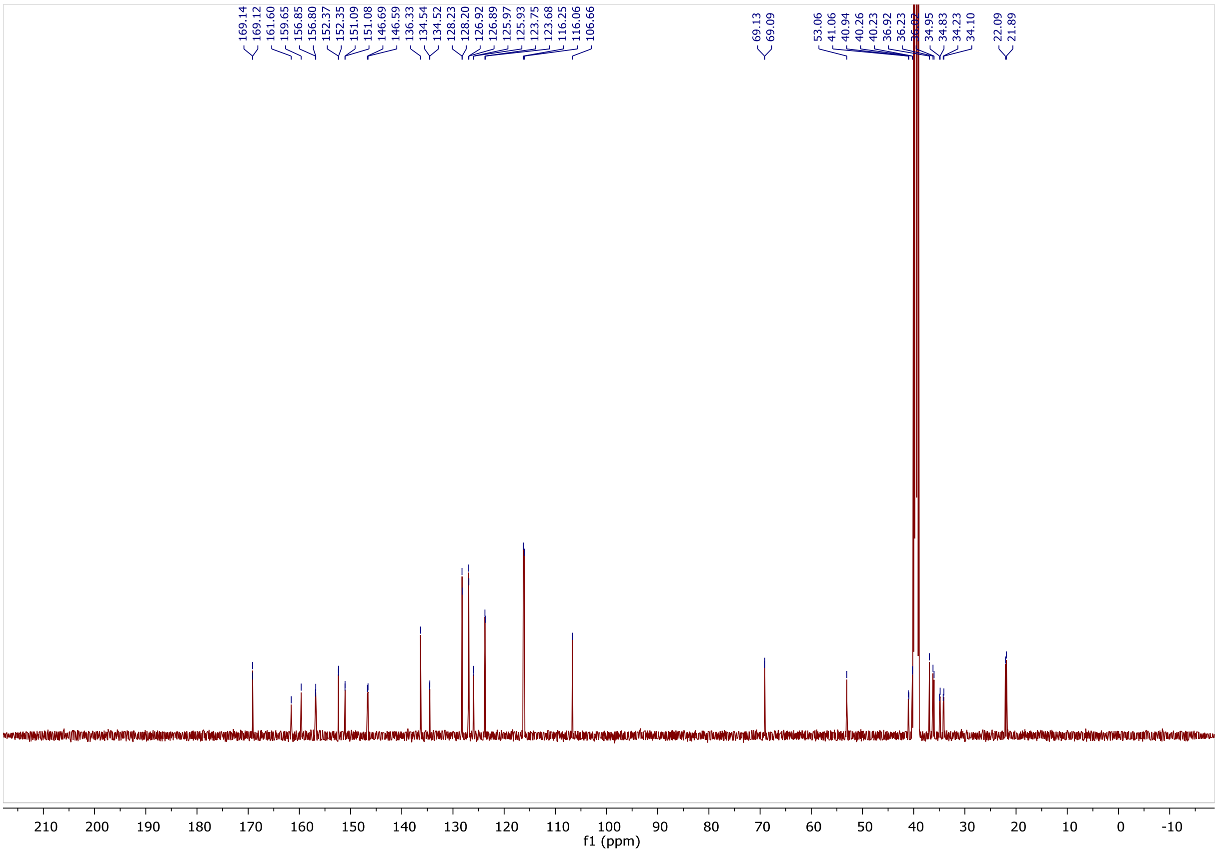

^{1}H NMR spectra of NK135.

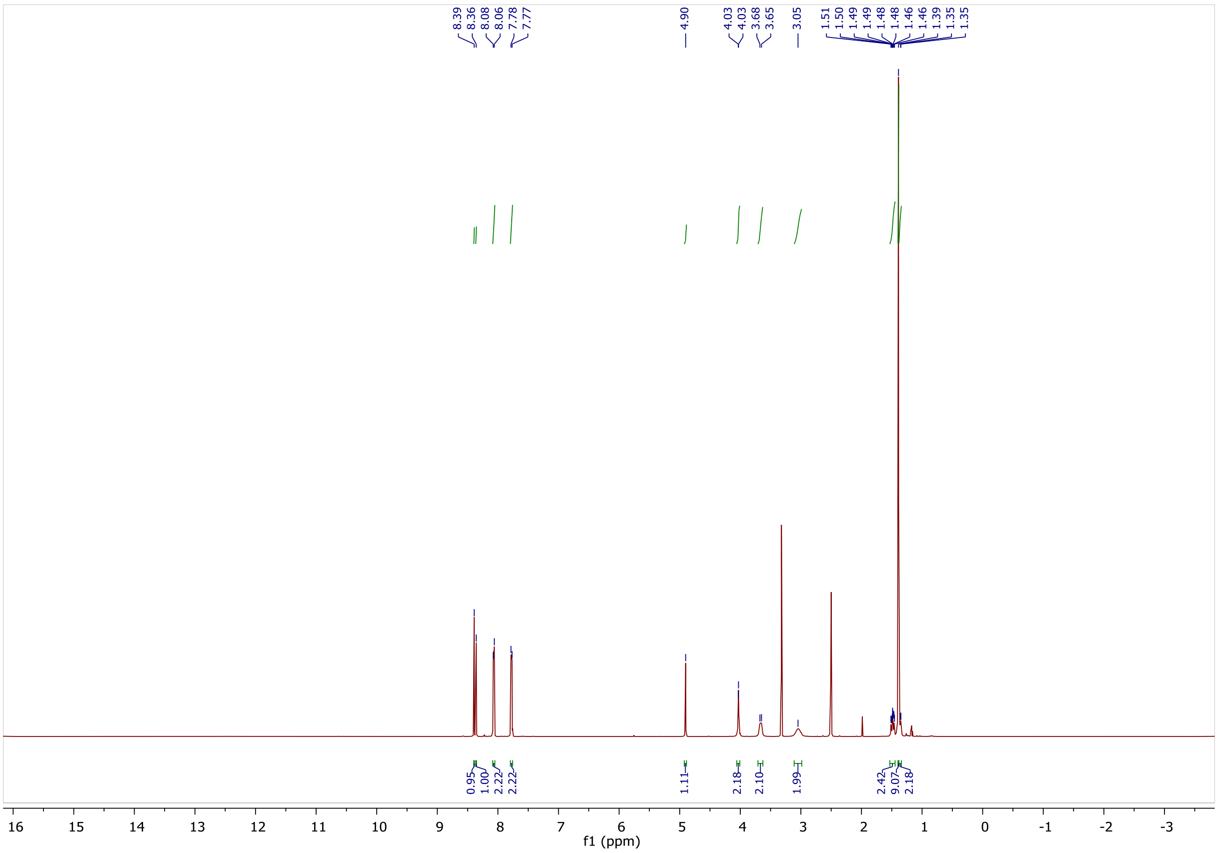

^{13}C NMR spectra of NK135.

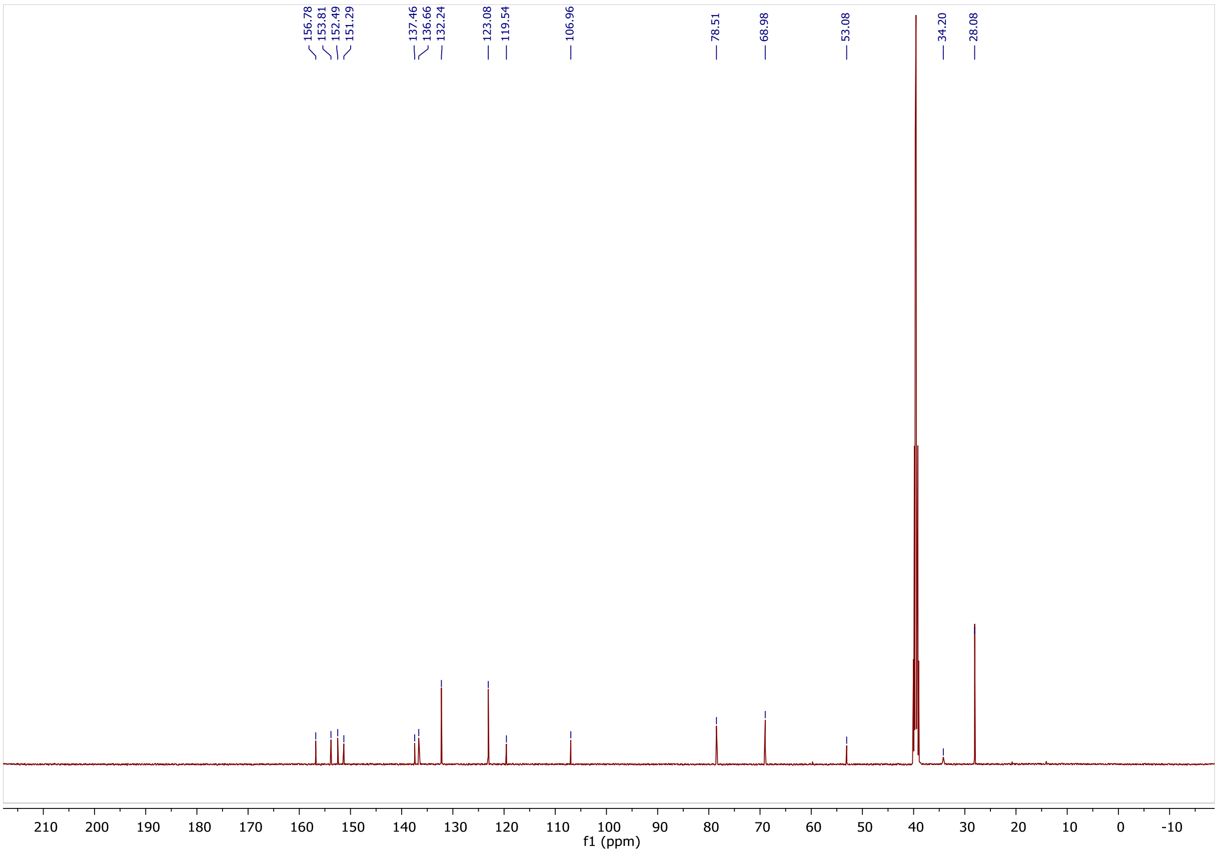

^{1}H NMR spectra of NK137.

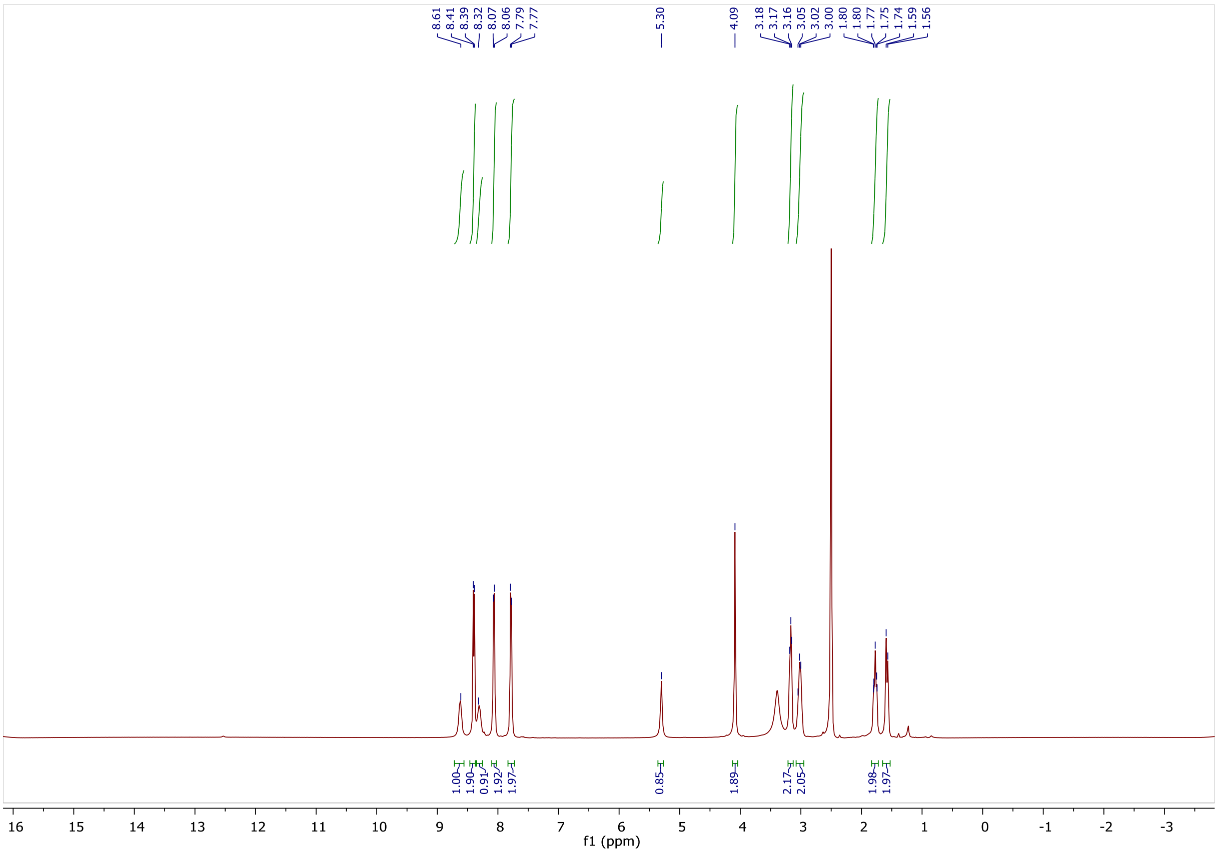

^{13}C NMR spectra of NK137.

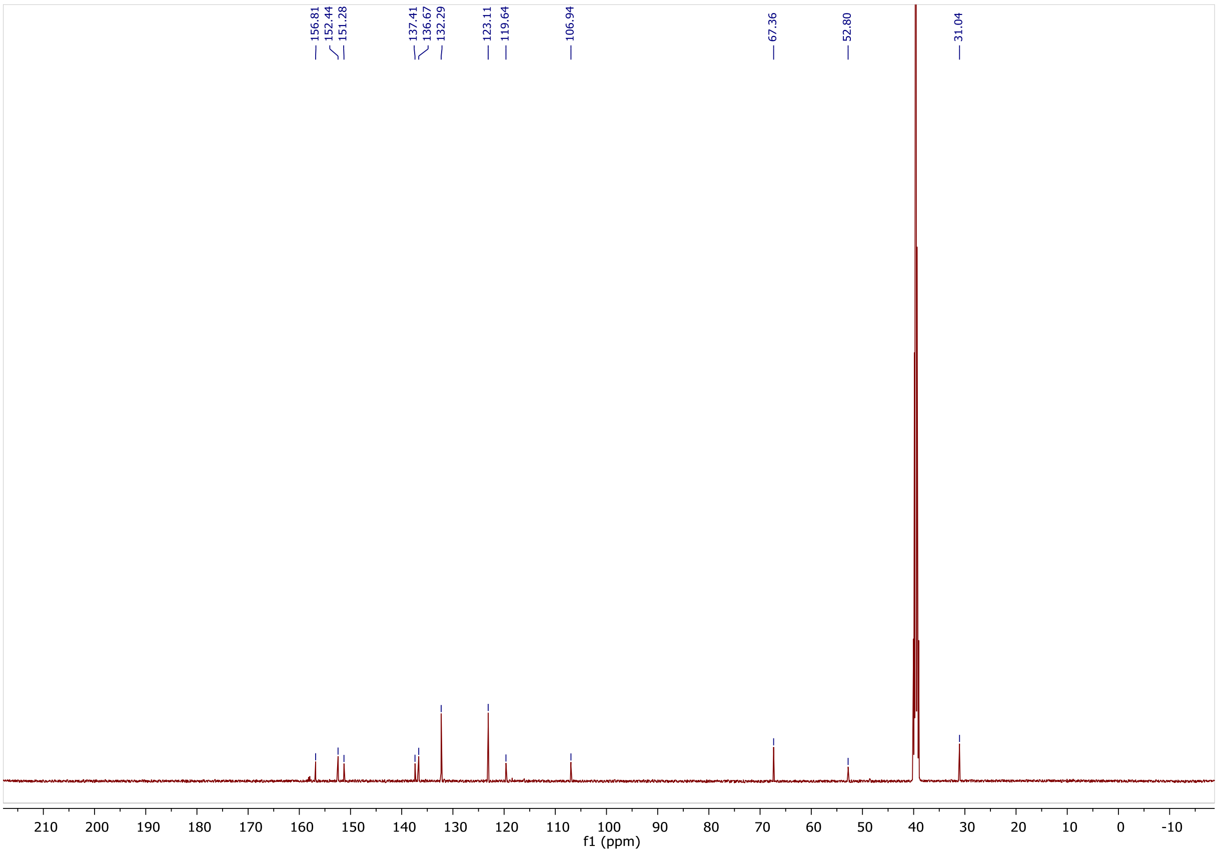

^{1}H NMR spectra of NK195.

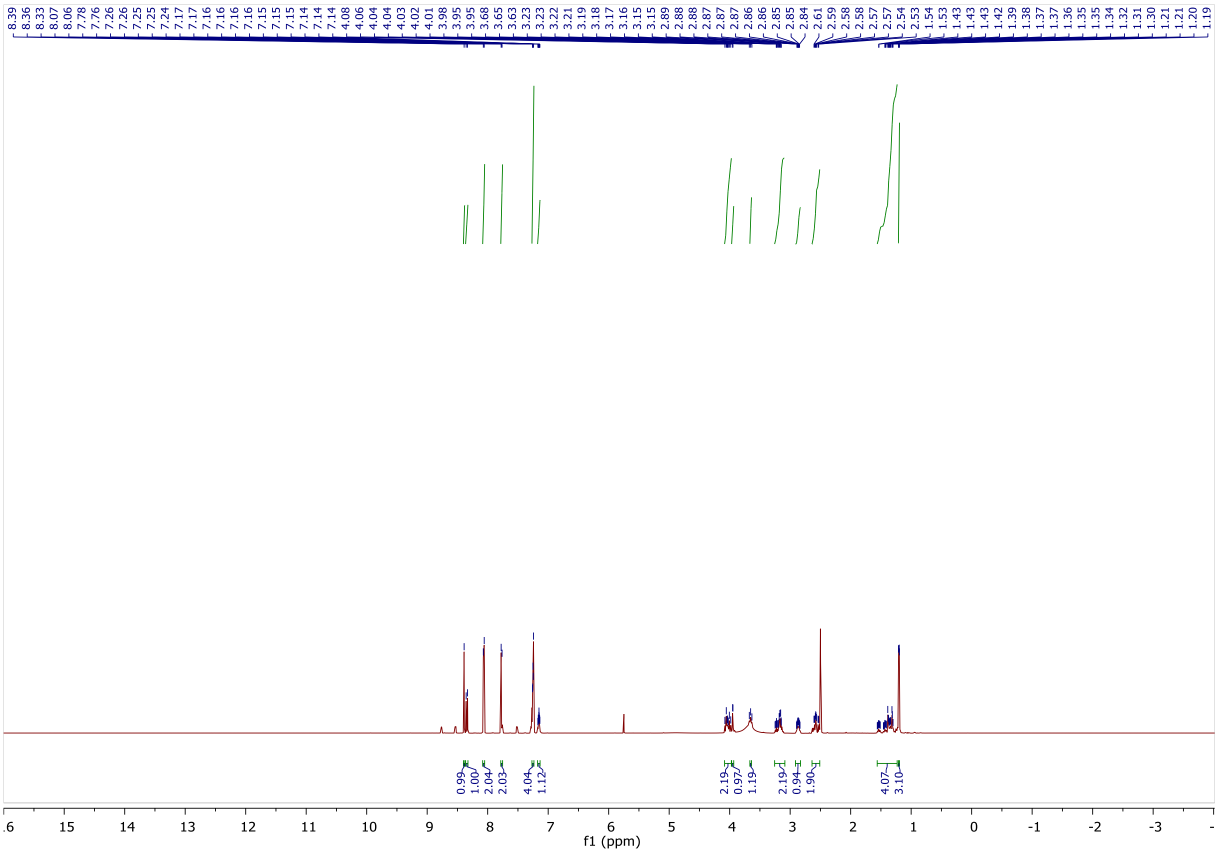

^{13}C NMR spectra of NK195.

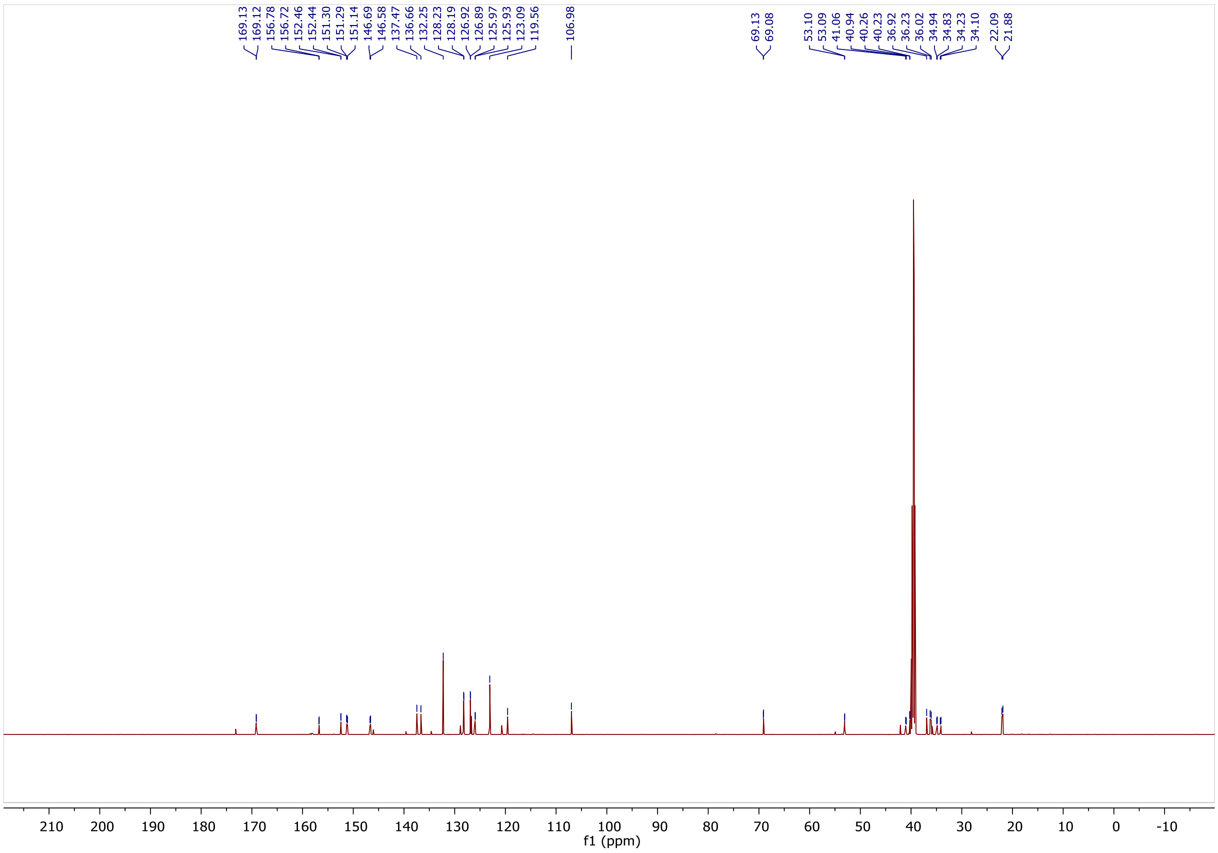

^{1}H NMR spectra of NK210.

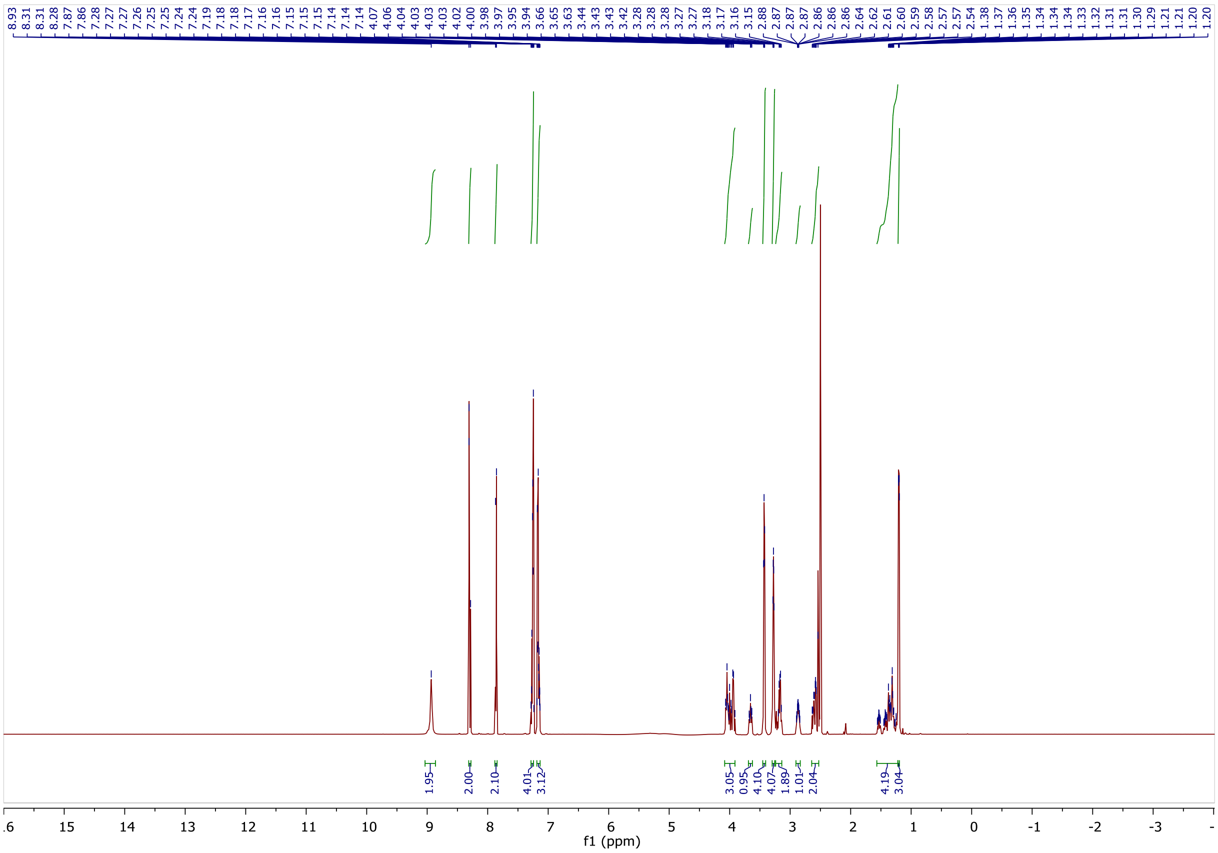

^{13}C NMR spectra of NK210.

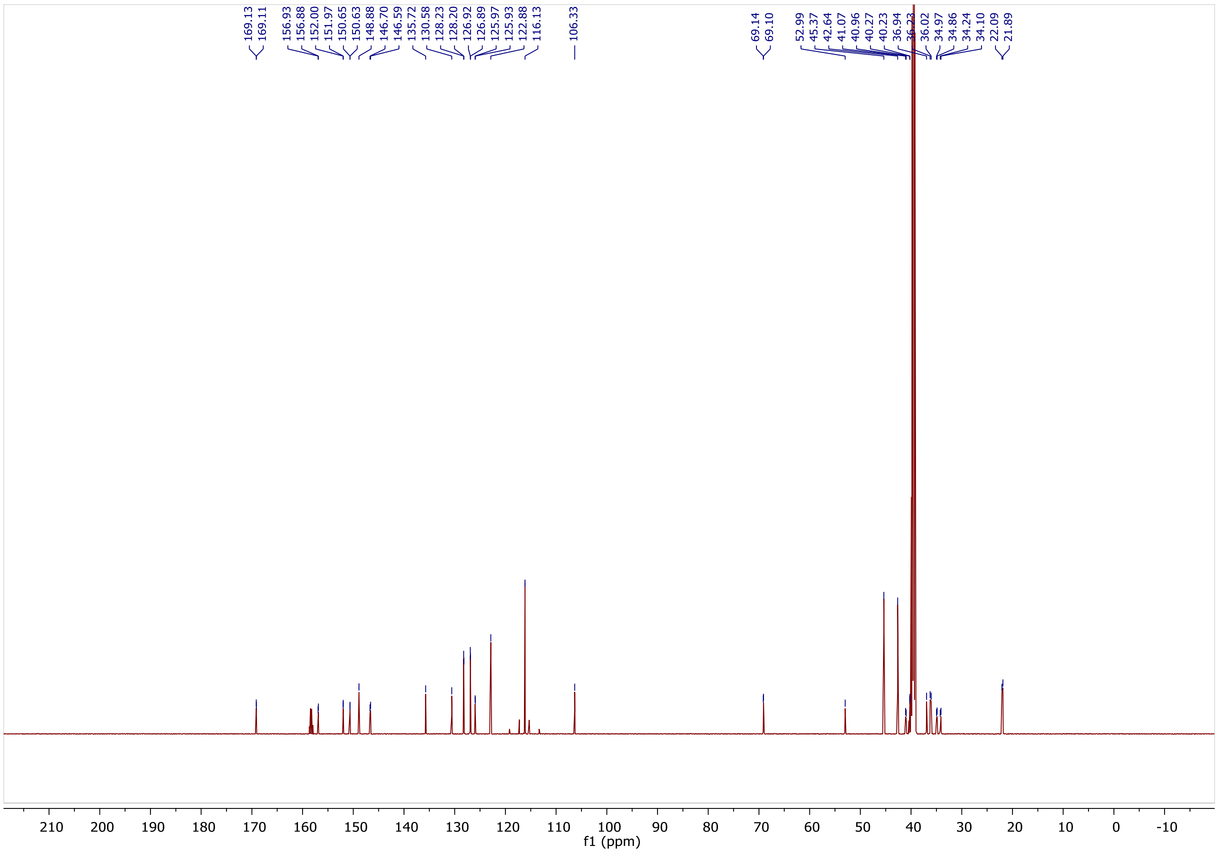

^{1}H NMR spectra of NK212.

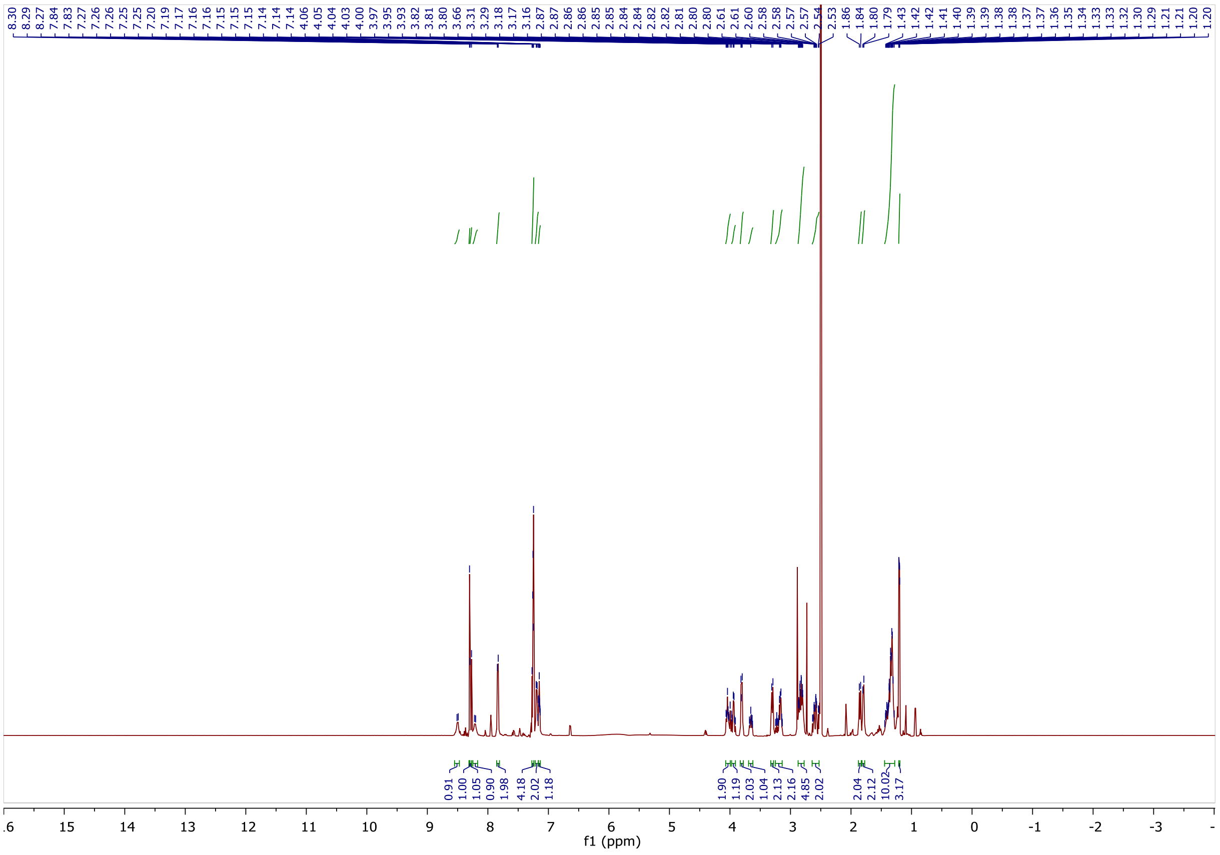

^{13}C NMR spectra of NK212.

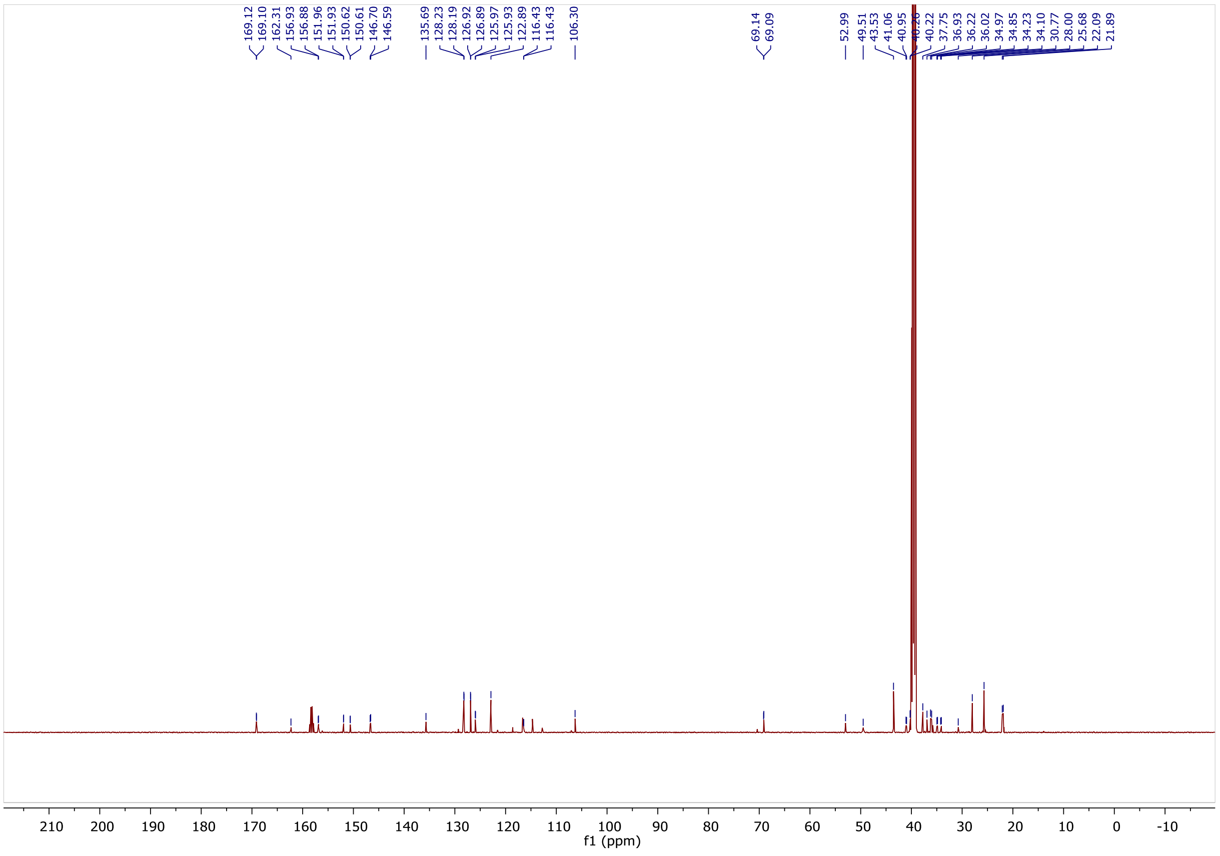

^{1}H NMR spectra of NK220.

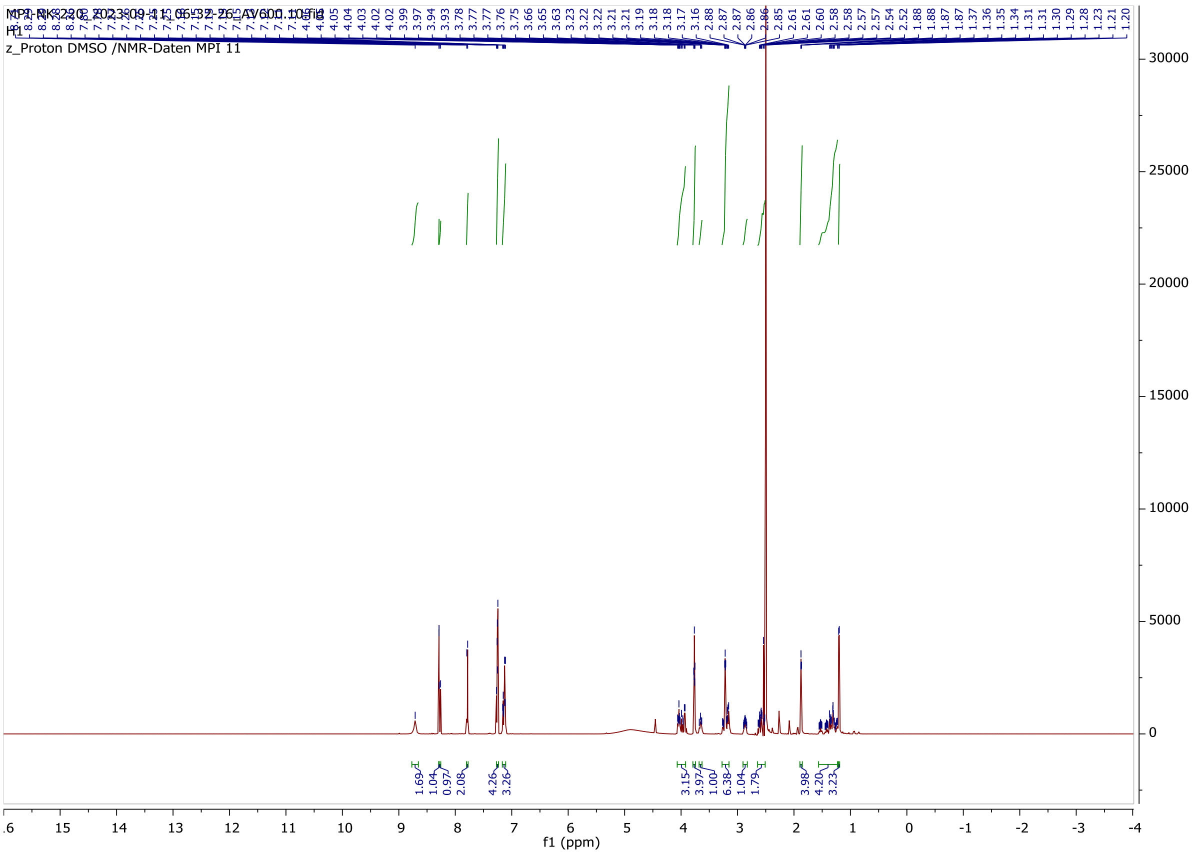

^{13}C NMR spectra of NK220.

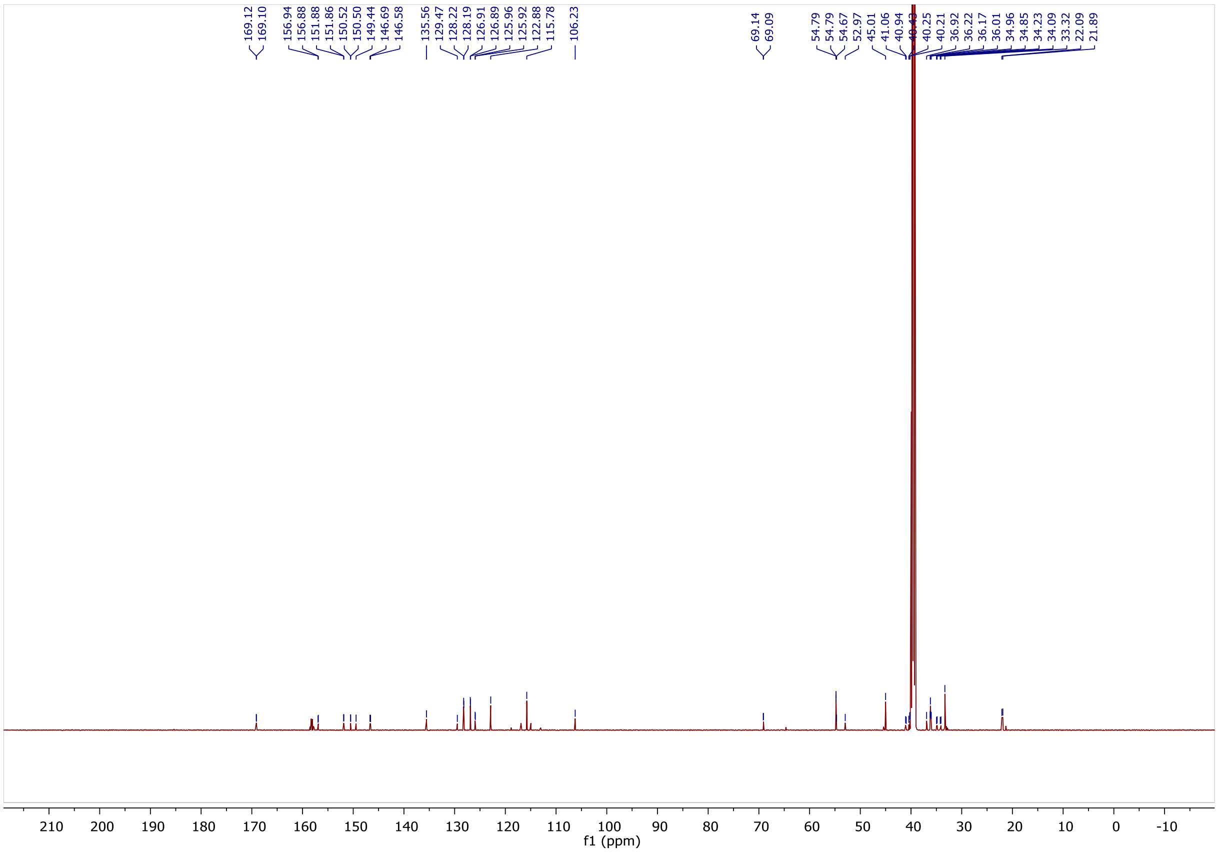

^{1}H NMR spectra of NK226.

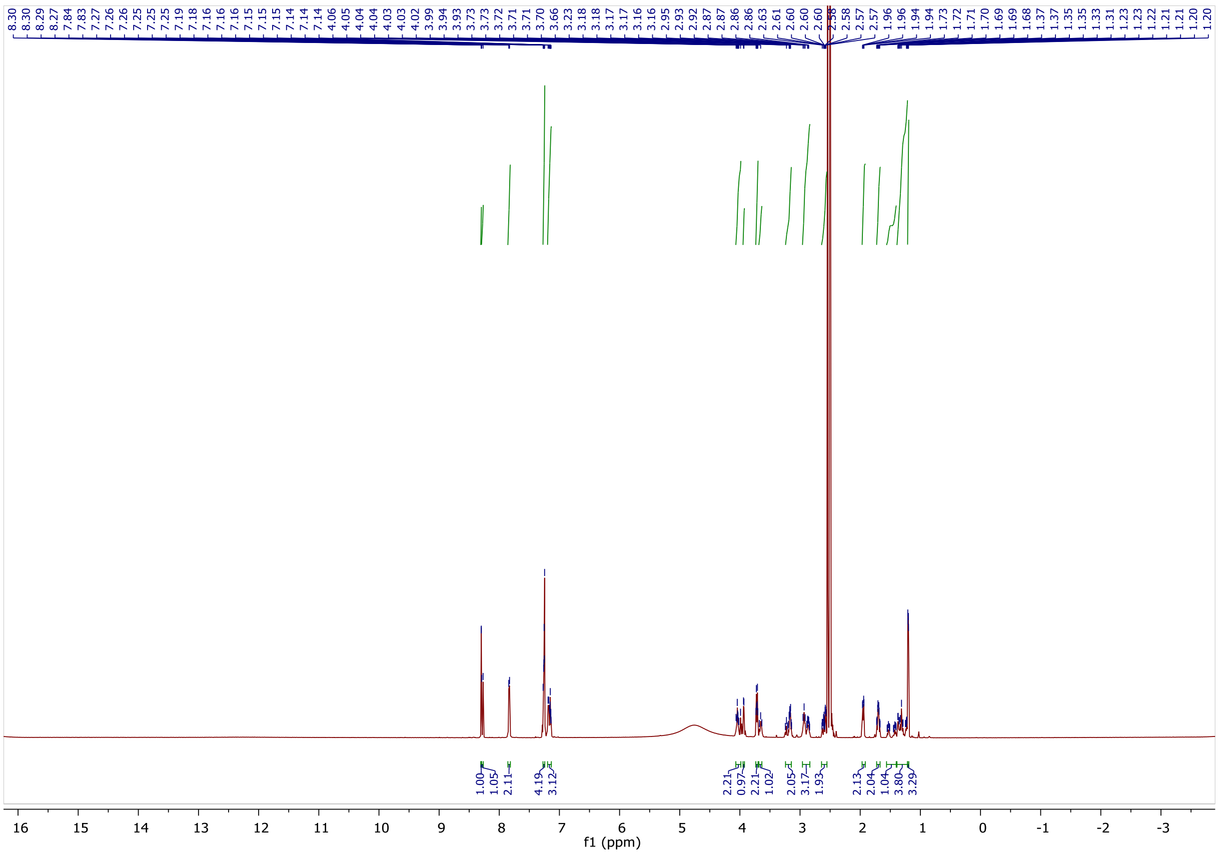

^{13}C NMR spectra of NK226.

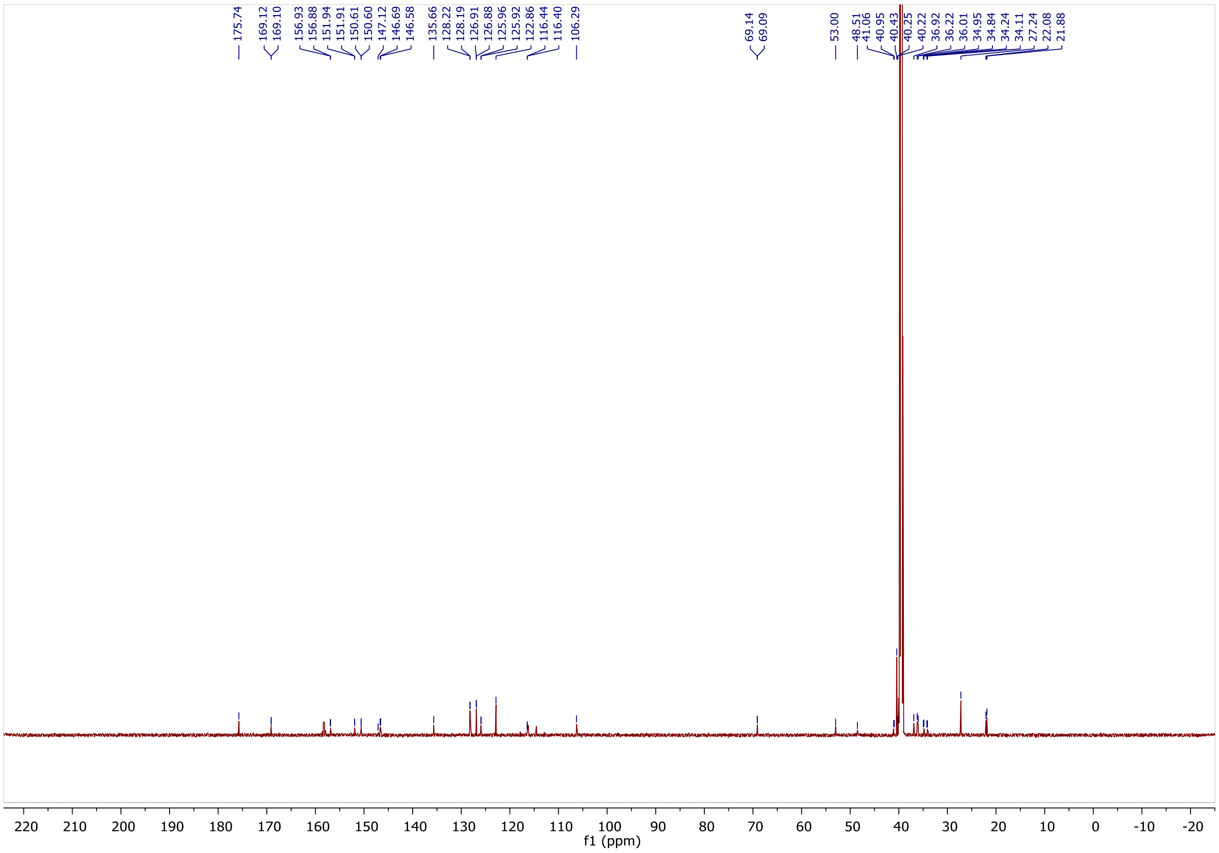

^{1}H NMR spectra of NK231.

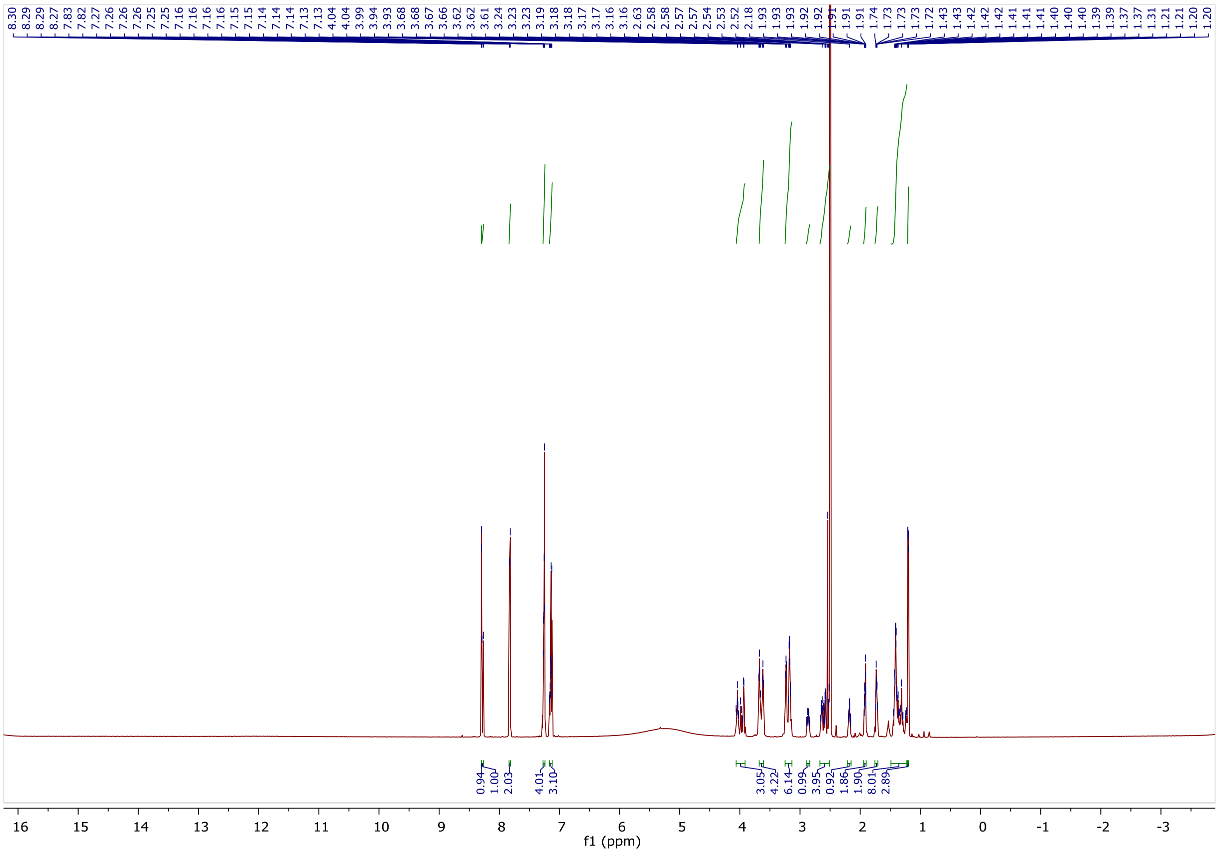

^{13}C NMR spectra of NK231.

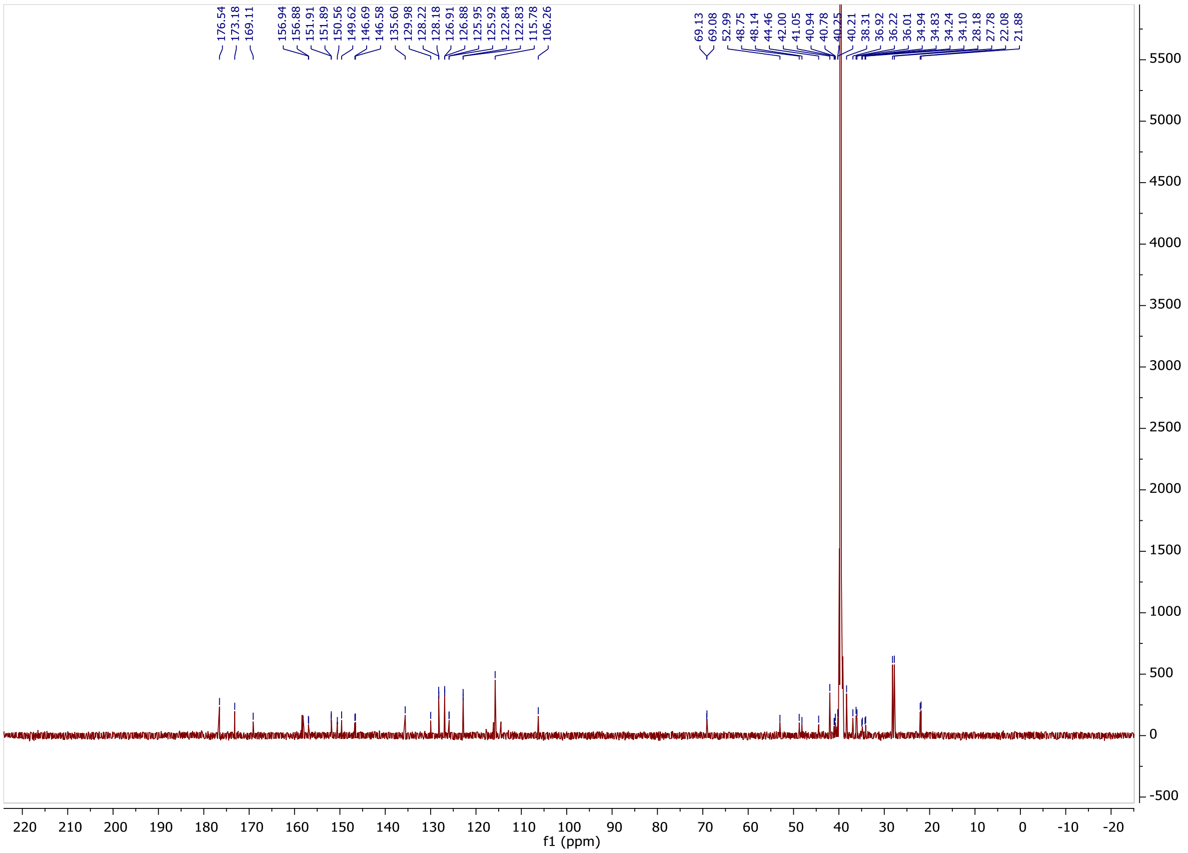

^{1}H NMR spectra of NK267.

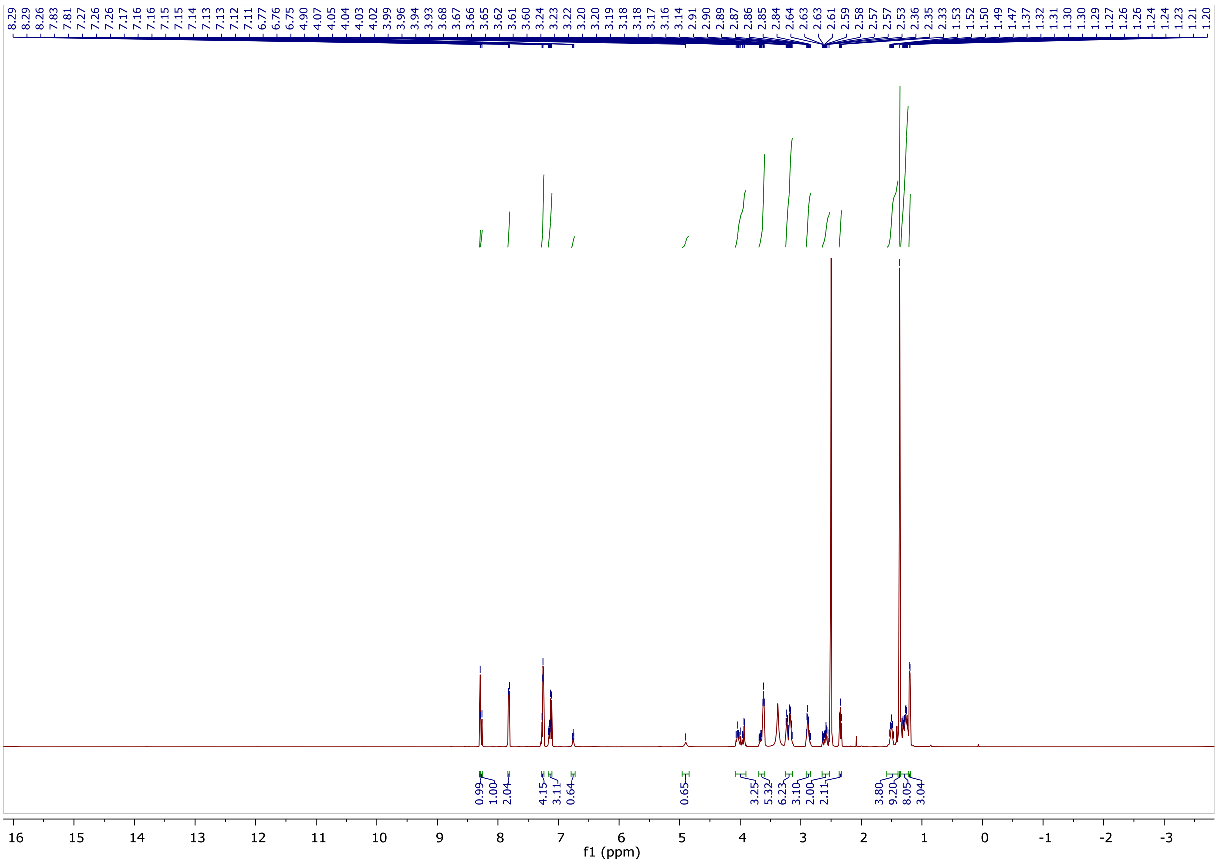

^{13}C NMR spectra of NK267.

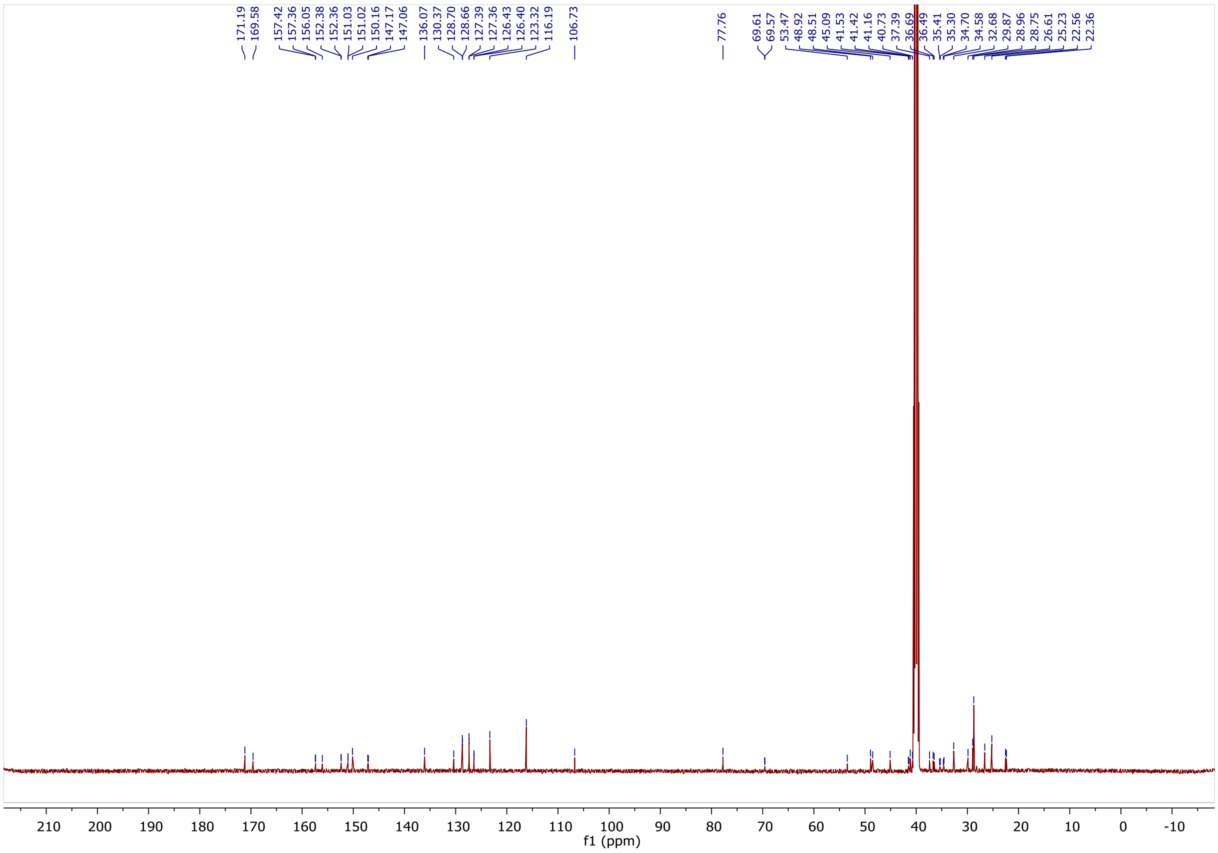

^{1}H NMR spectra of NK264.

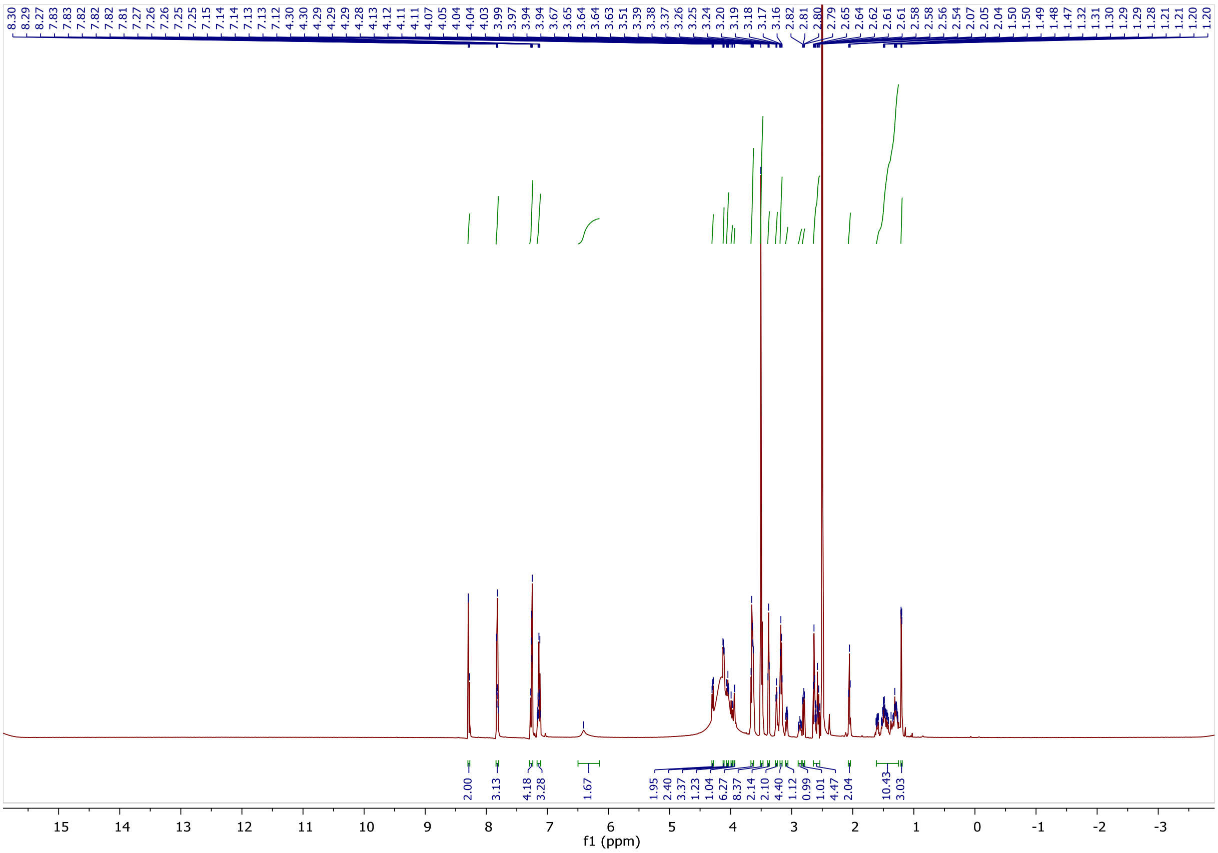

^{13}C NMR spectra of NK264.

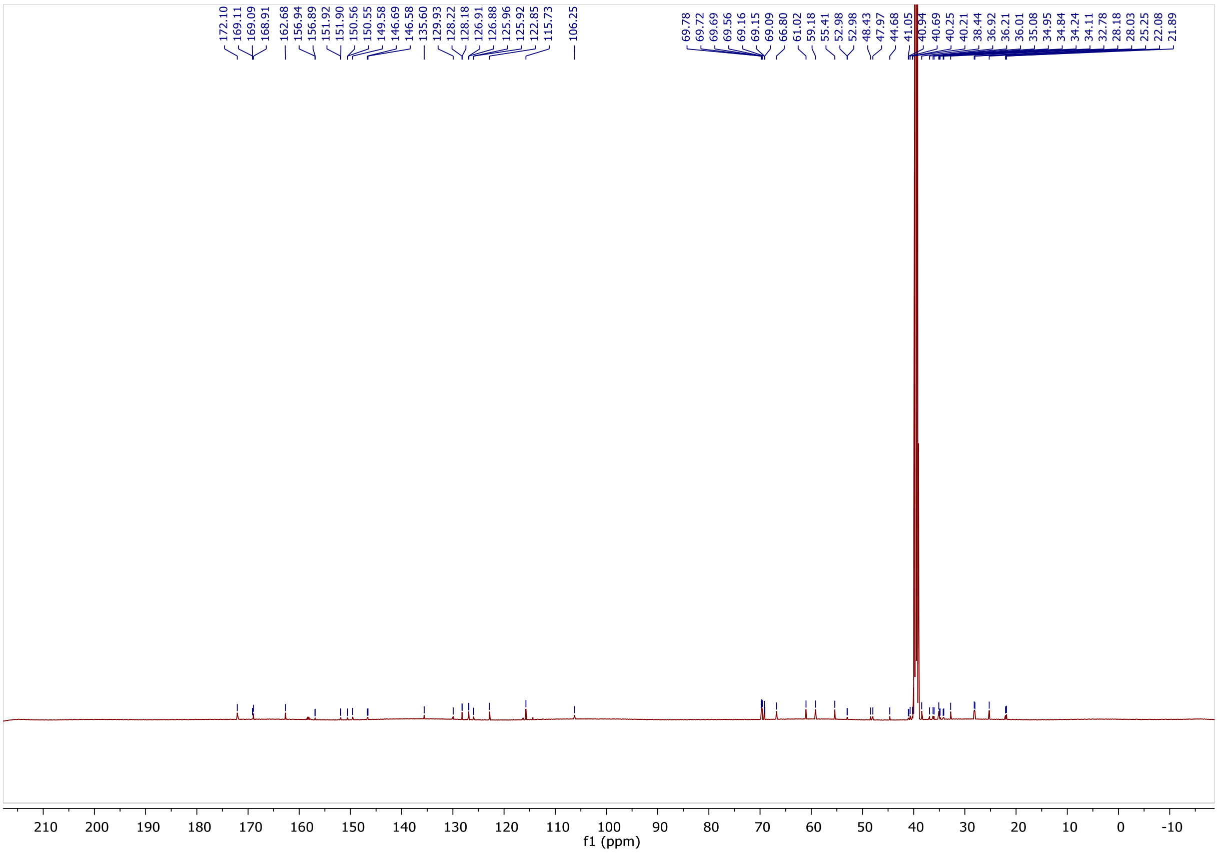

^{1}H NMR spectra of NK206.

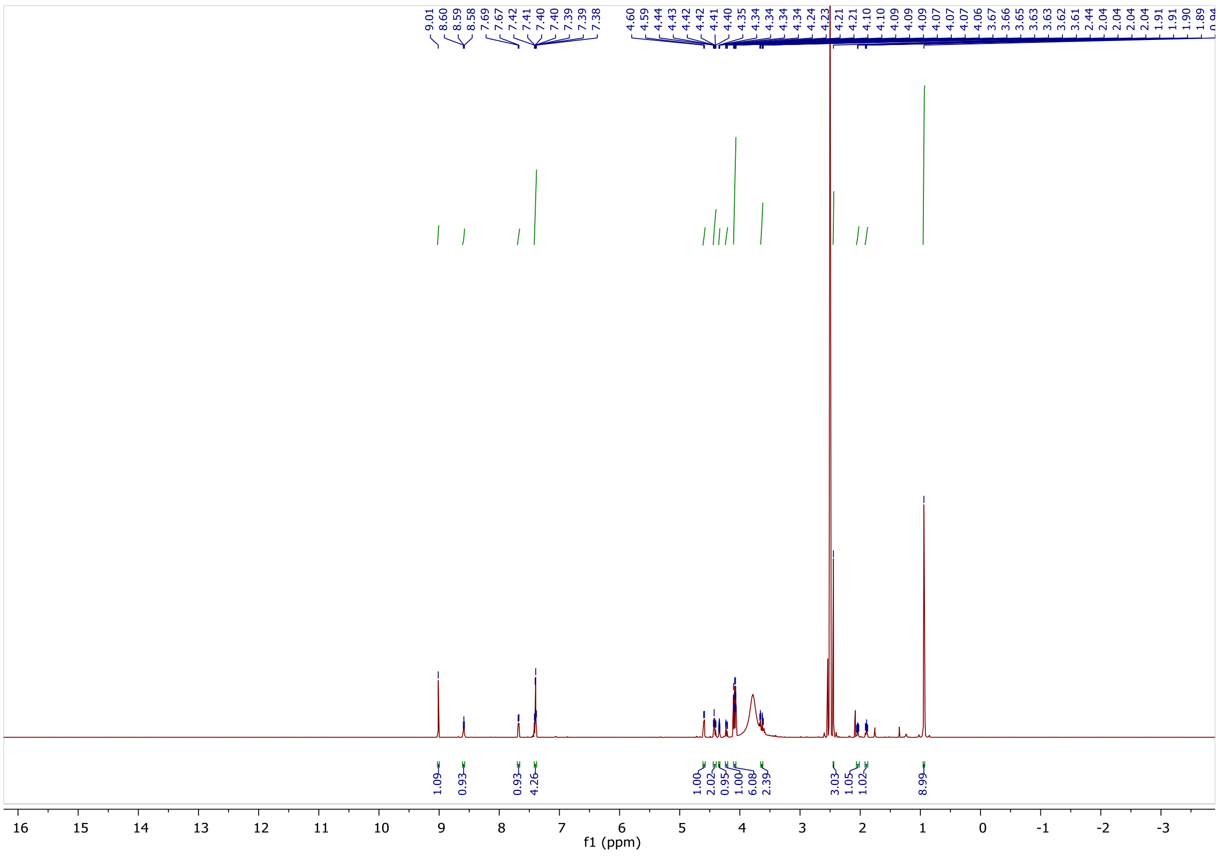

^{13}C NMR spectra of NK206.

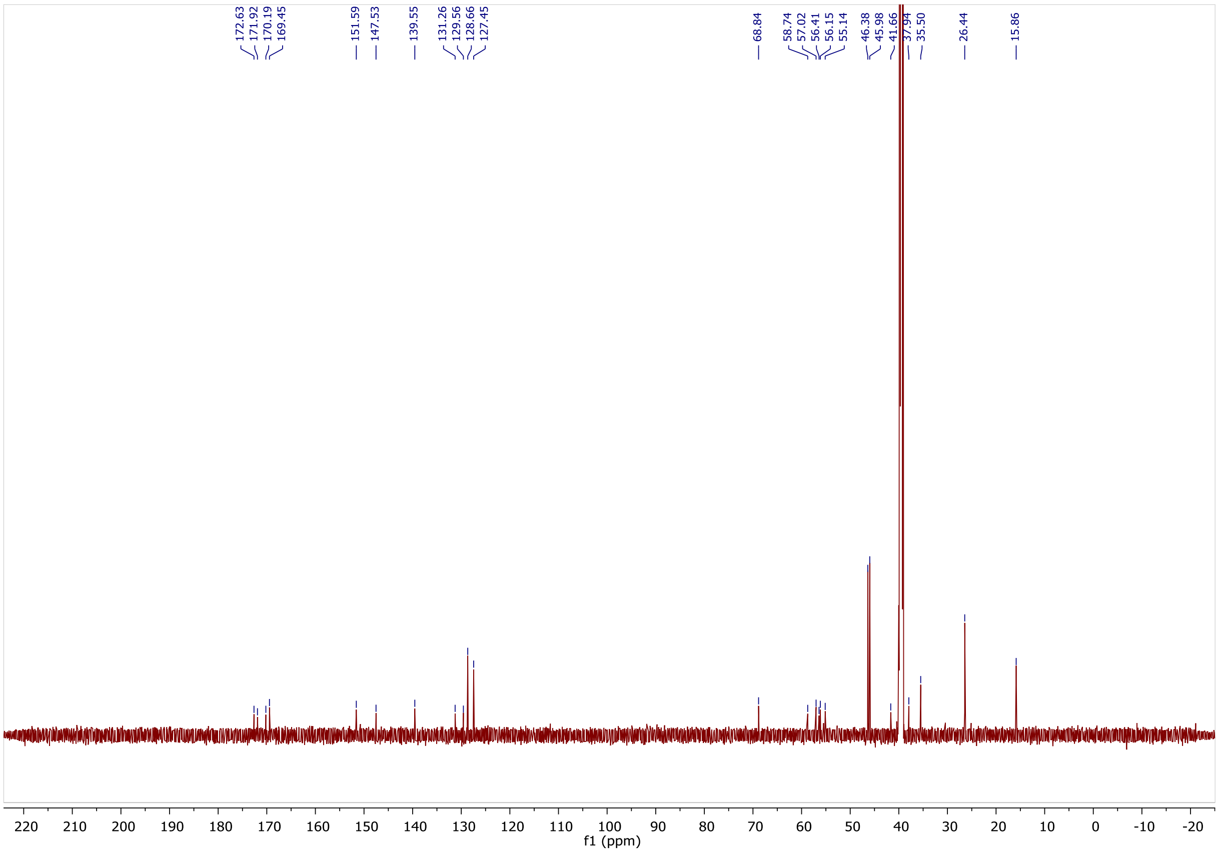

^{1}H NMR spectra of NK265_2.

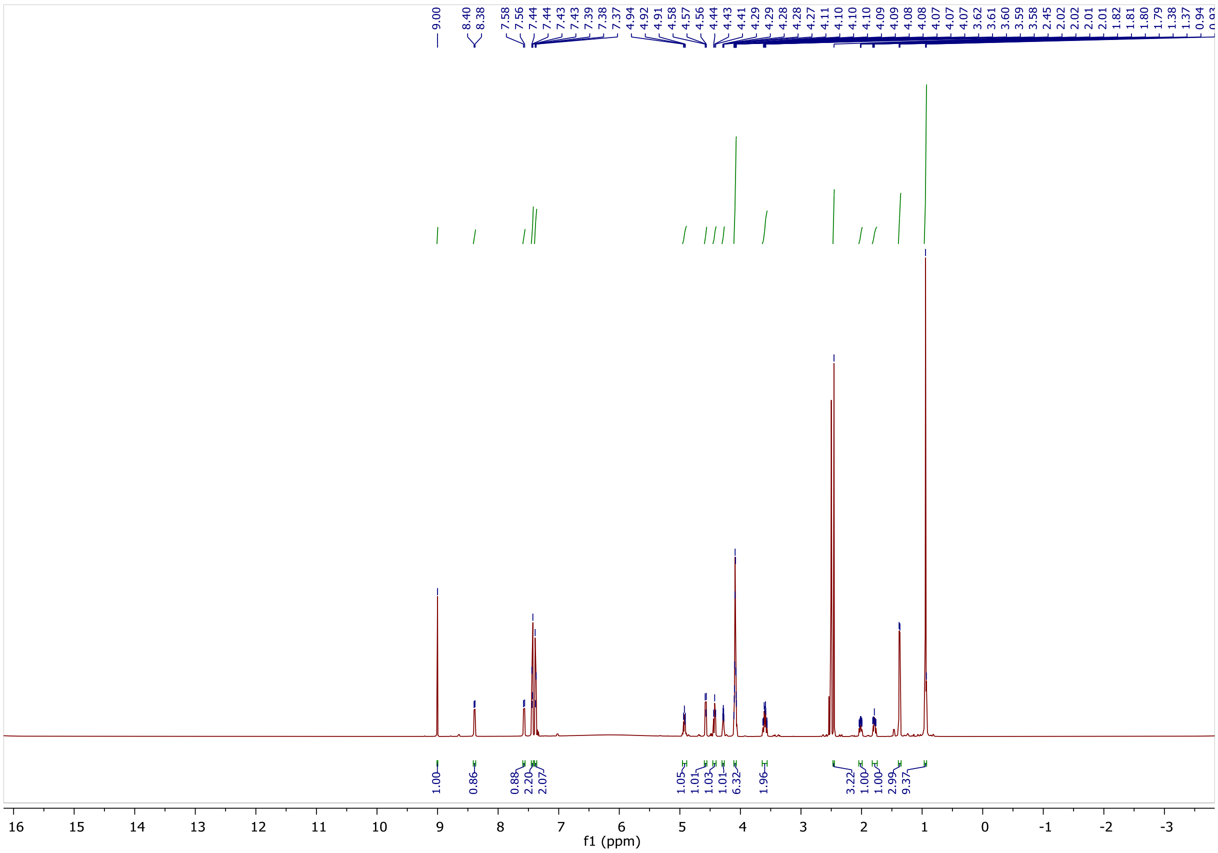

^{13}C NMR spectra of NK265_2.

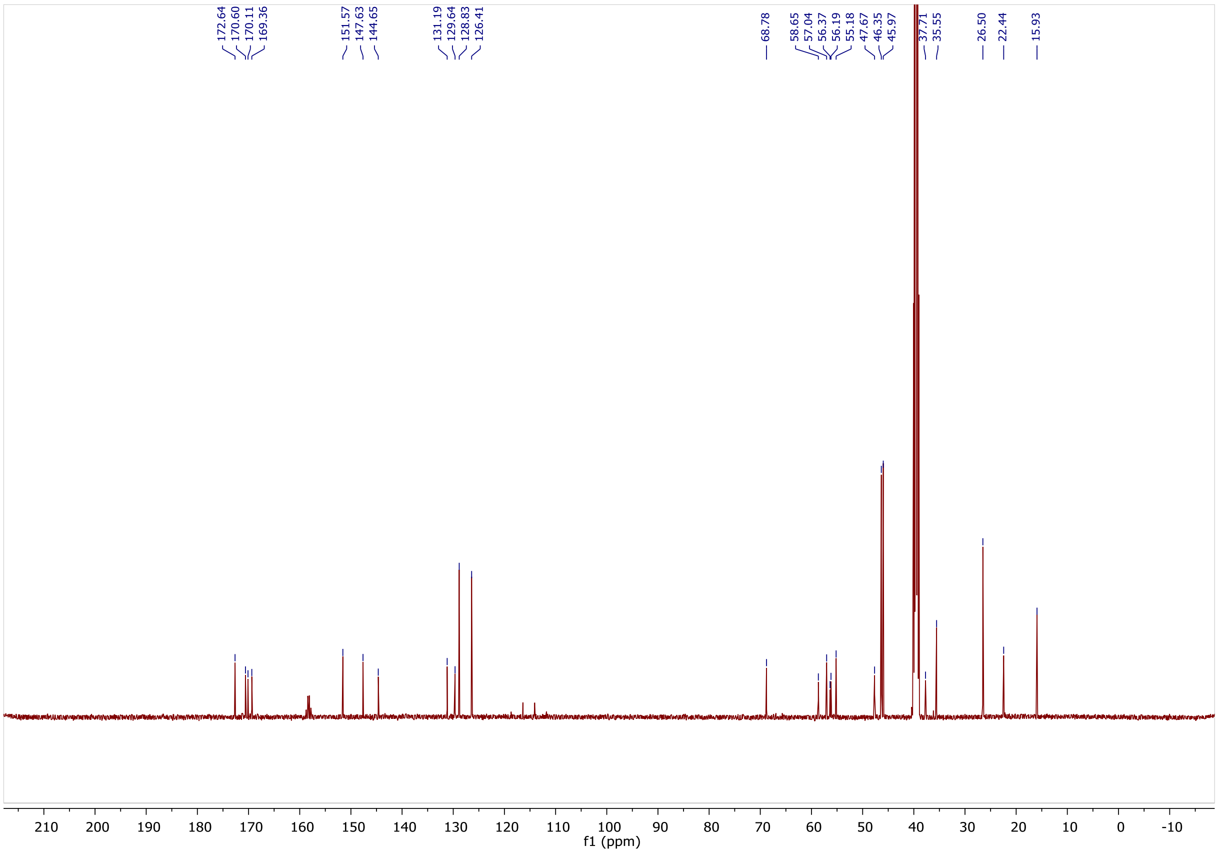

^{1}H NMR spectra of NK213.

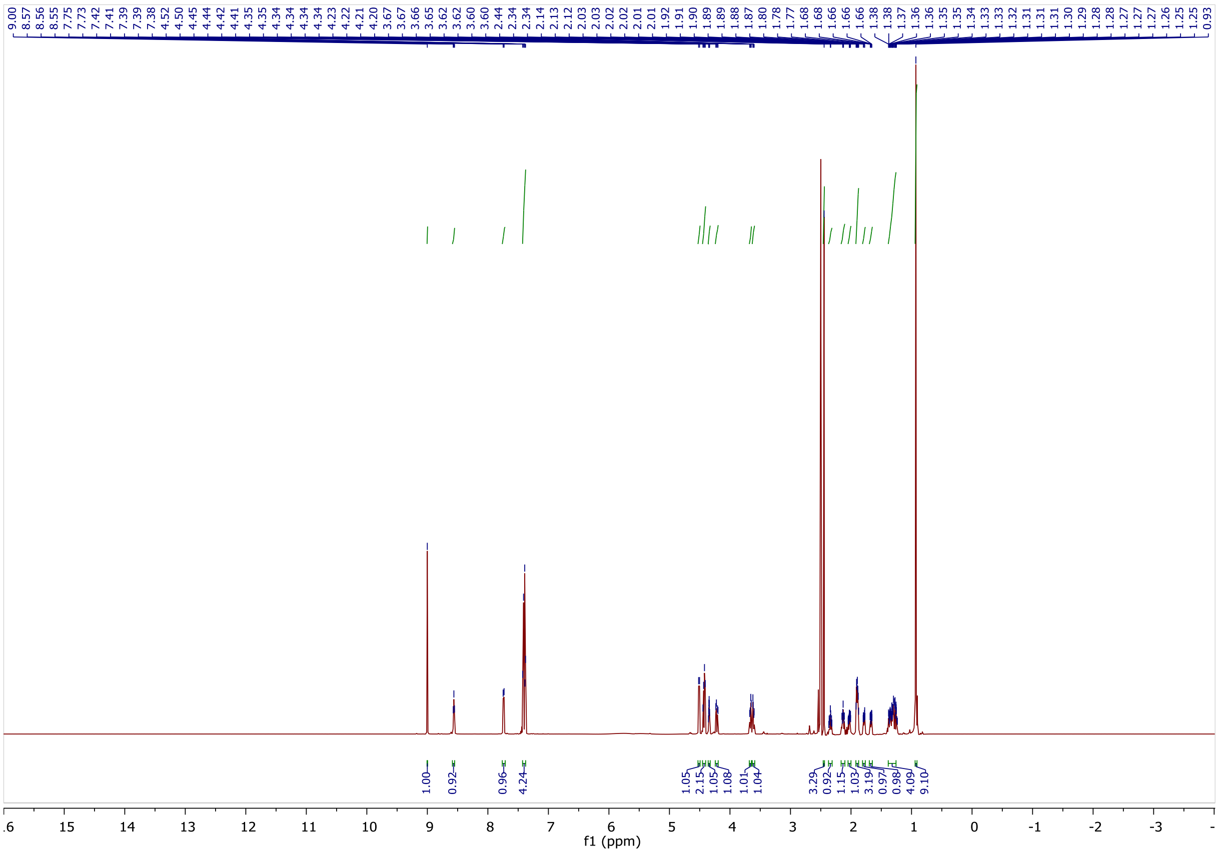

^{13}C NMR spectra of NK213.

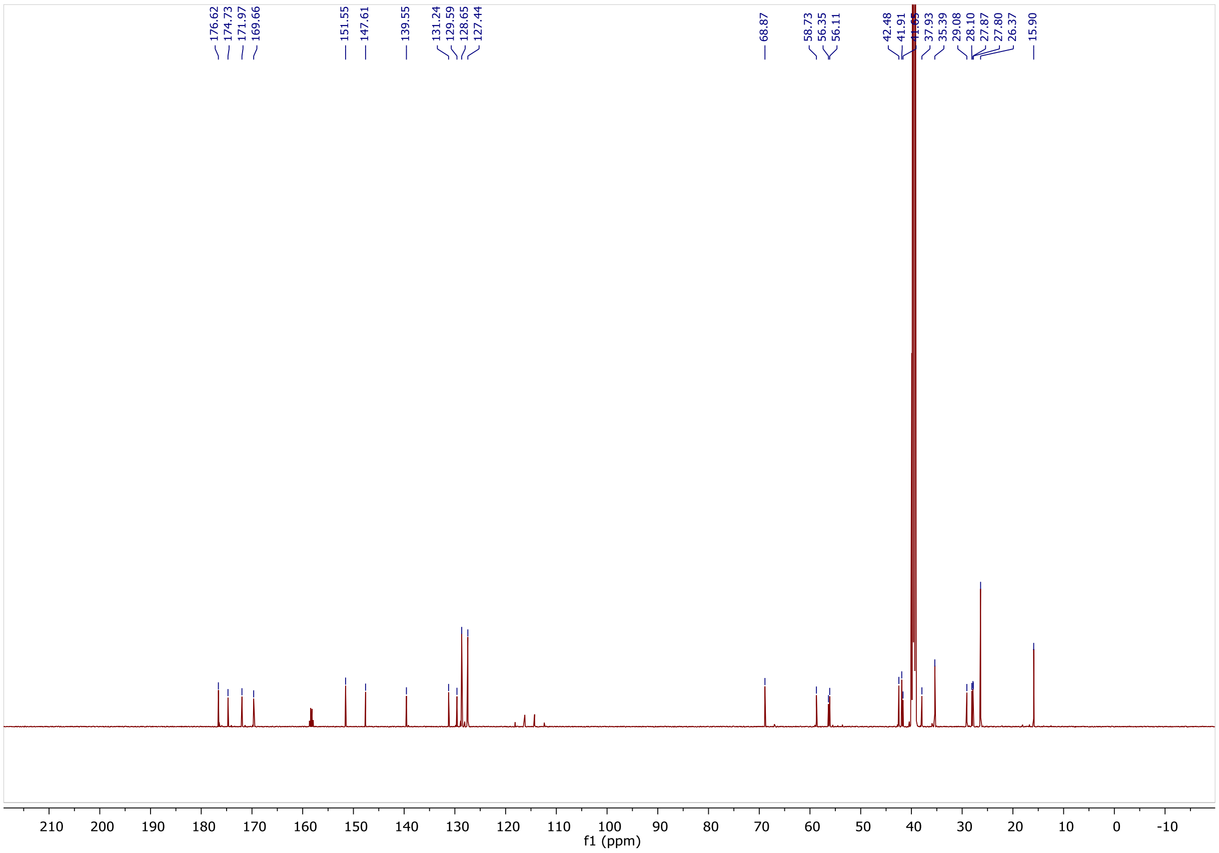

1H NMR spectra of NK244.

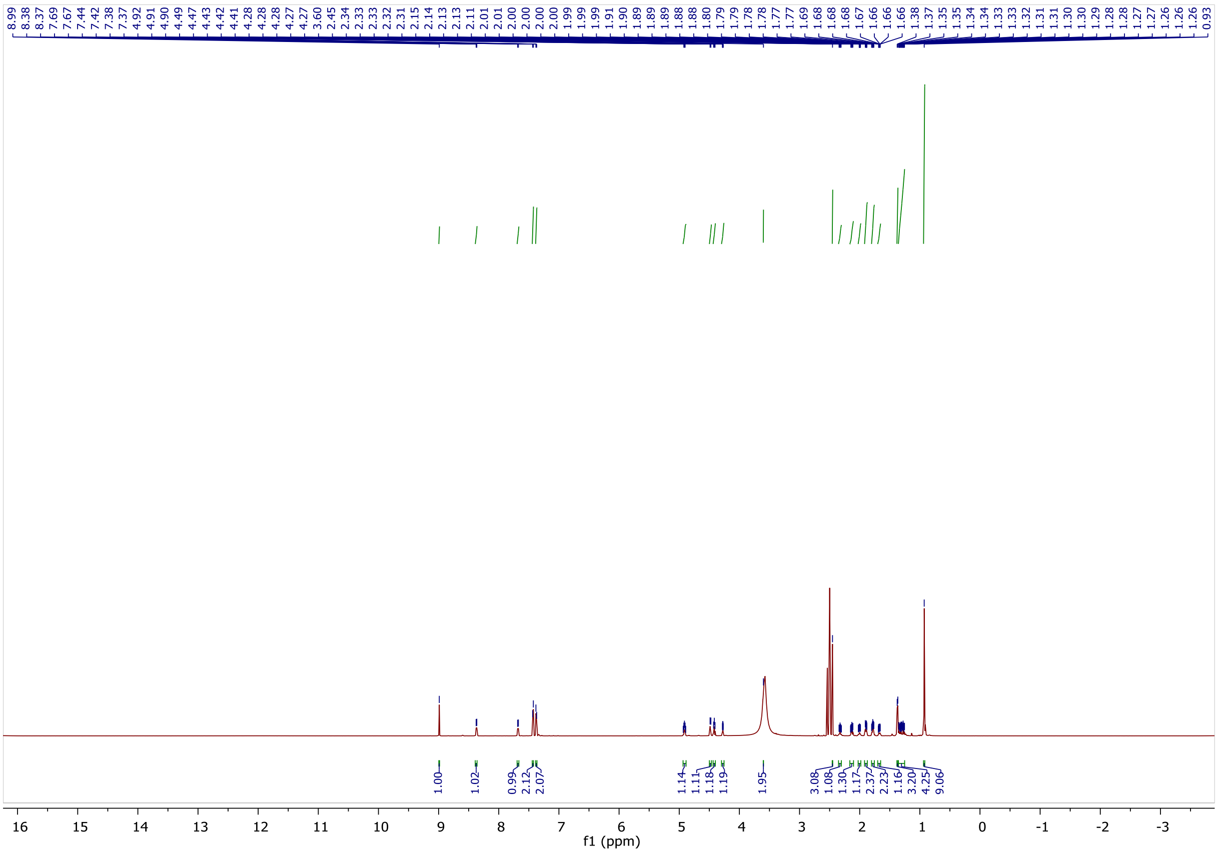

13C NMR spectra of NK244.

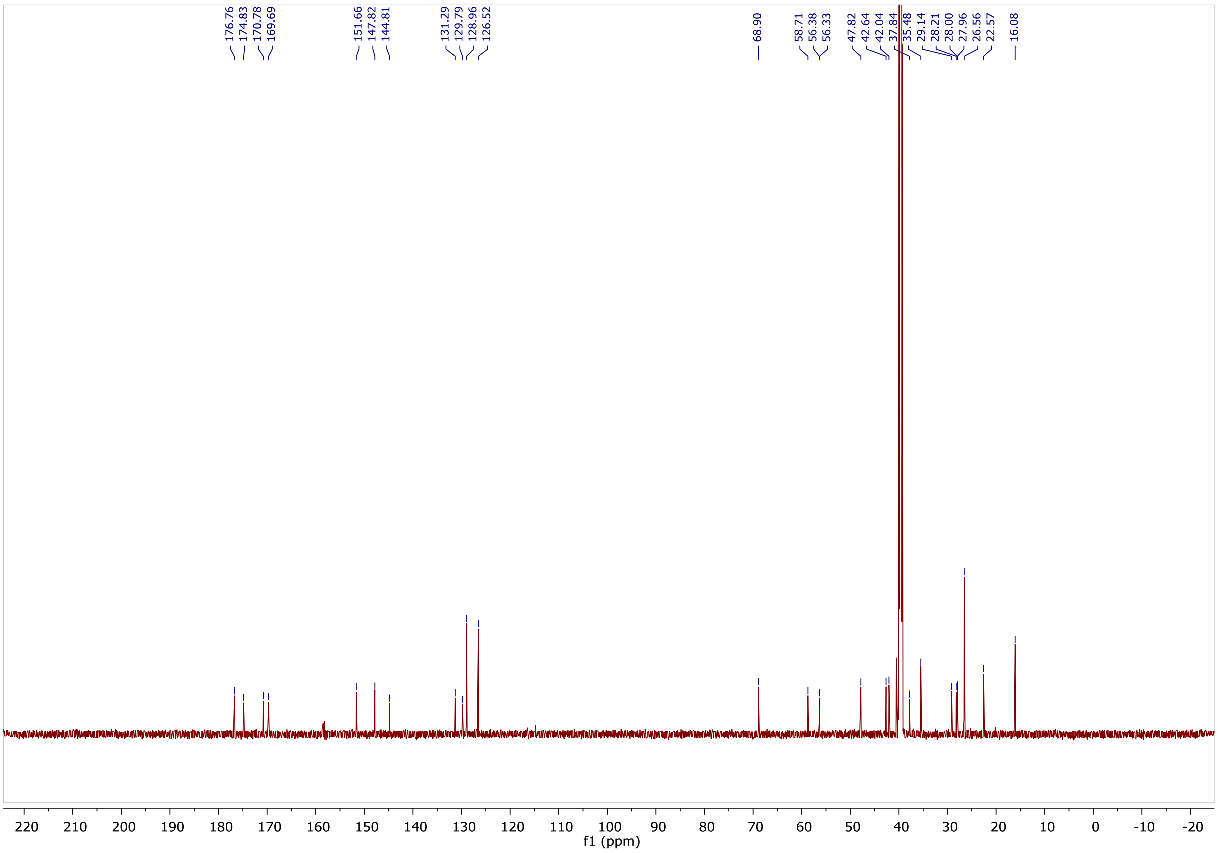

^{1}H NMR spectra of NK209.

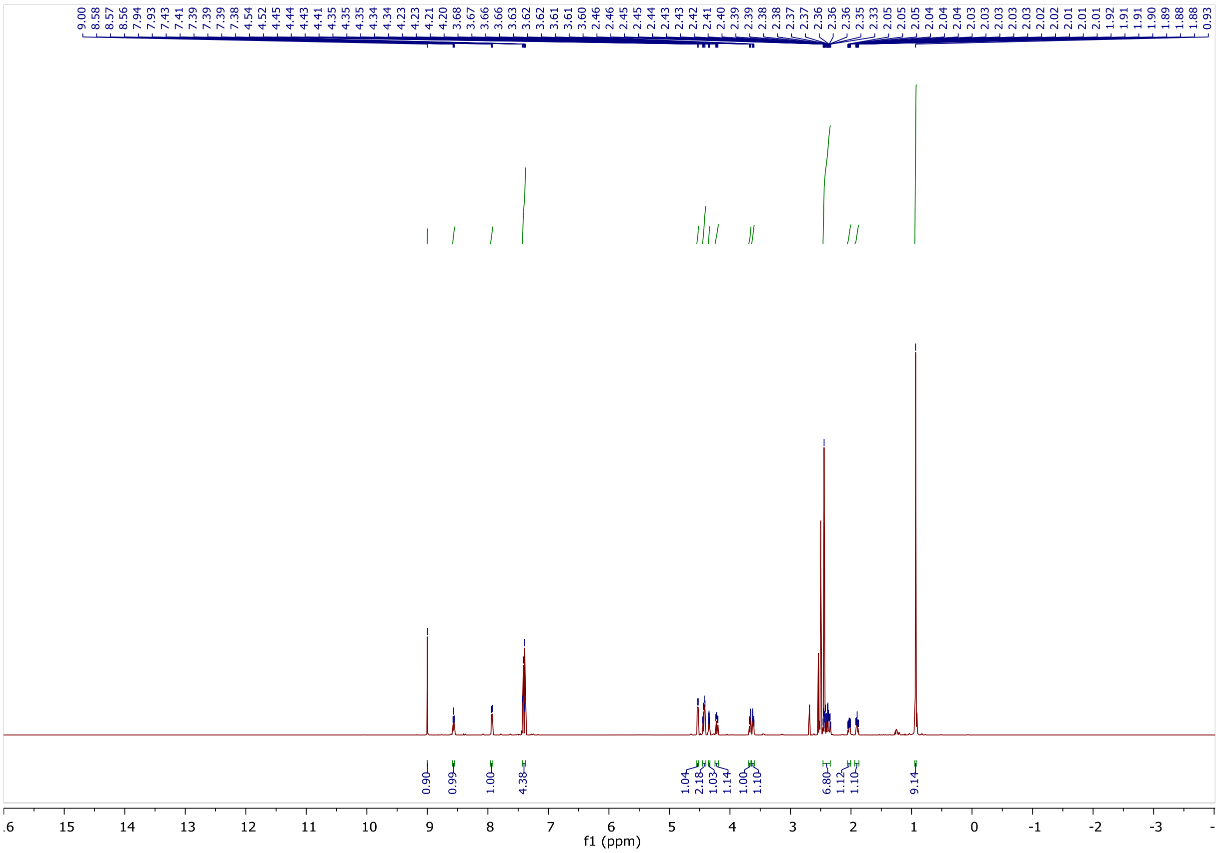

^{13}C NMR spectra of NK209.

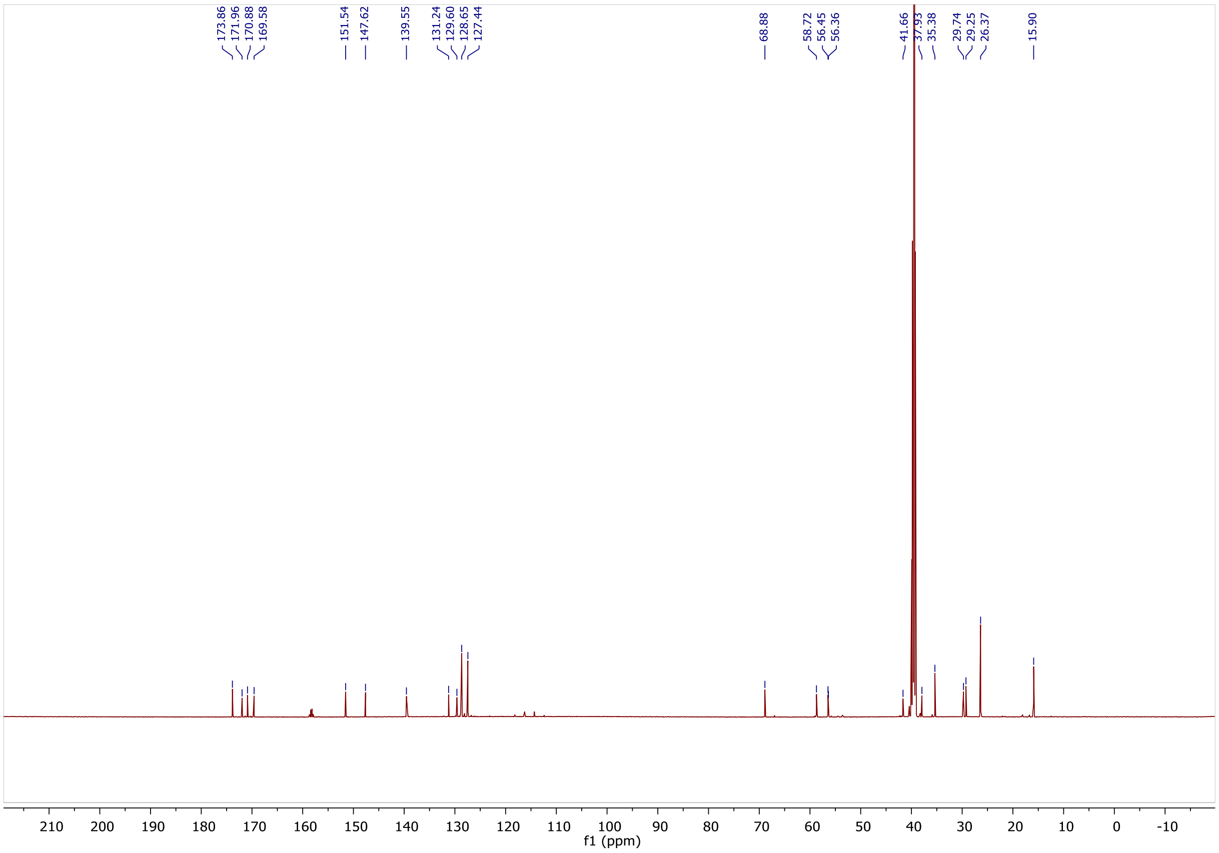

^{1}H NMR spectra of NK208.

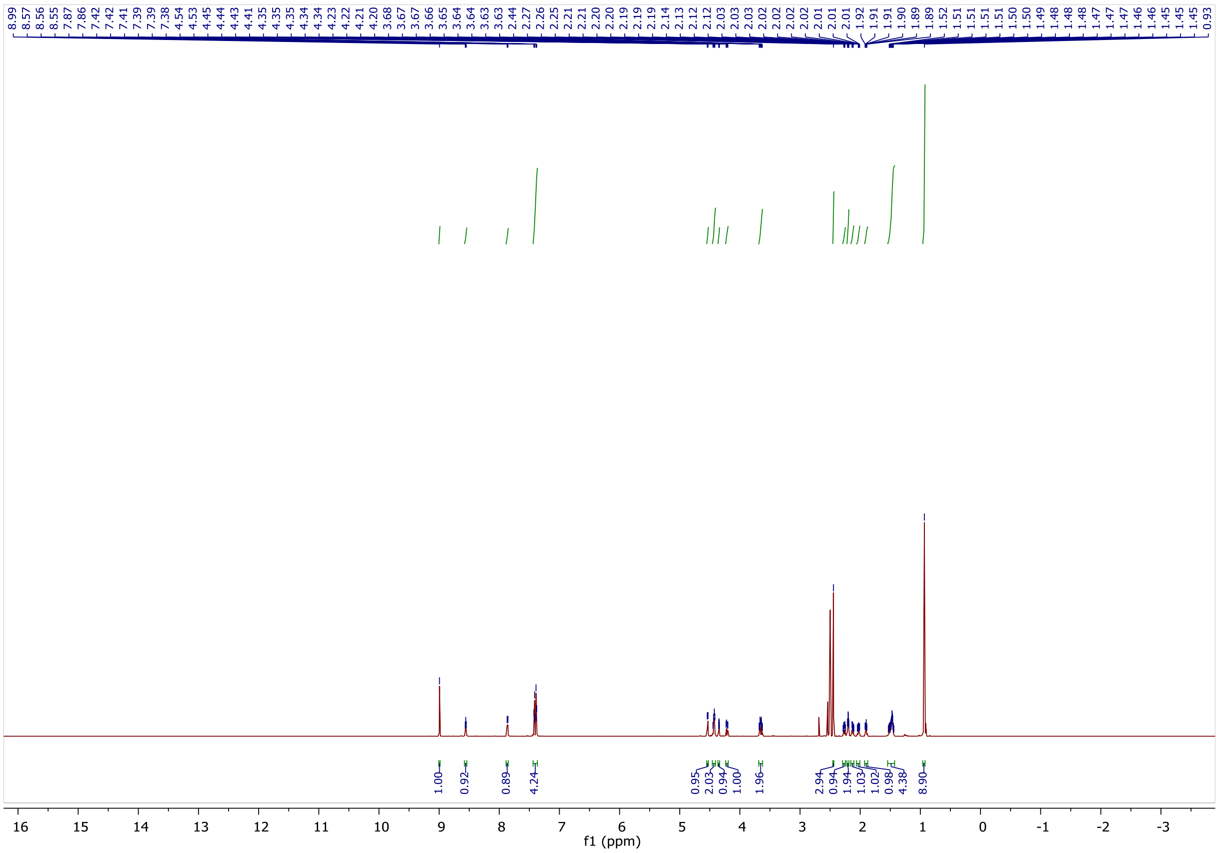

^{13}C NMR spectra of NK208.

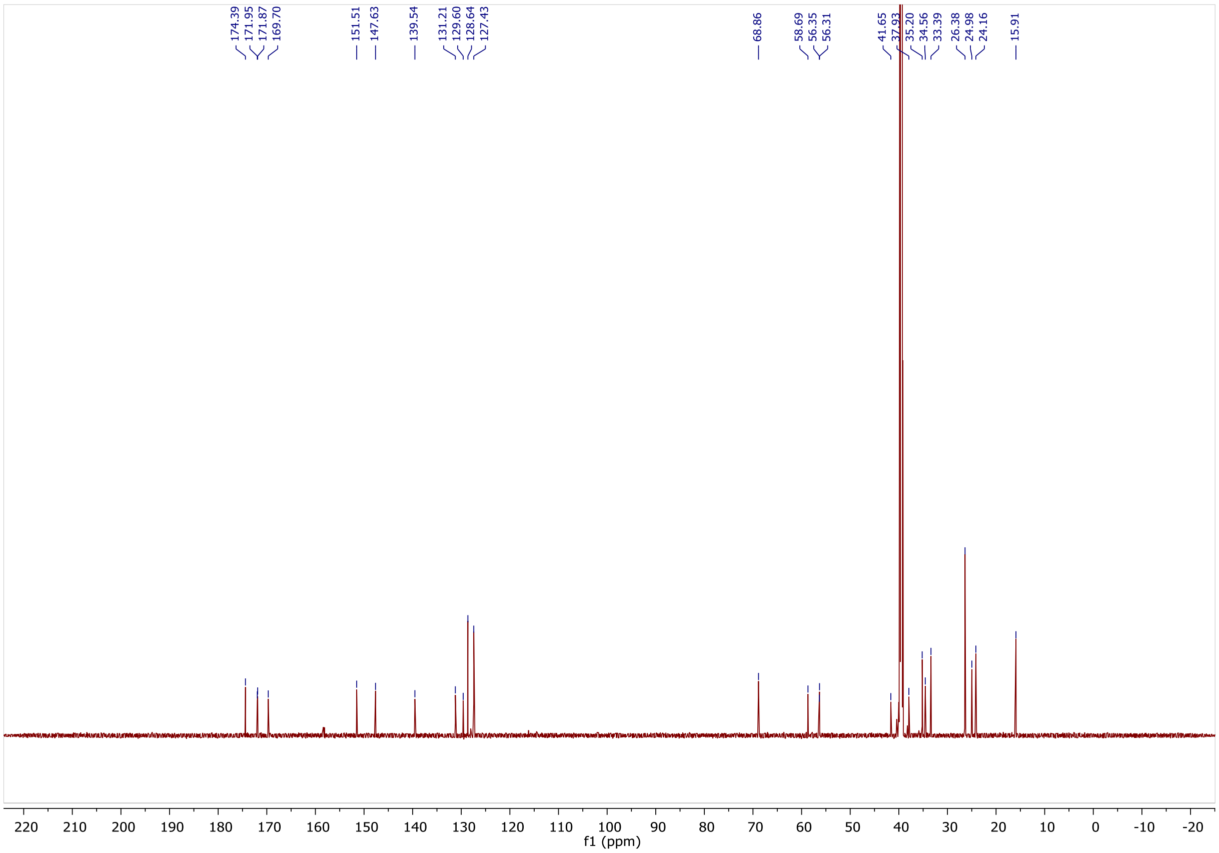

^1H NMR spectra of NK215.

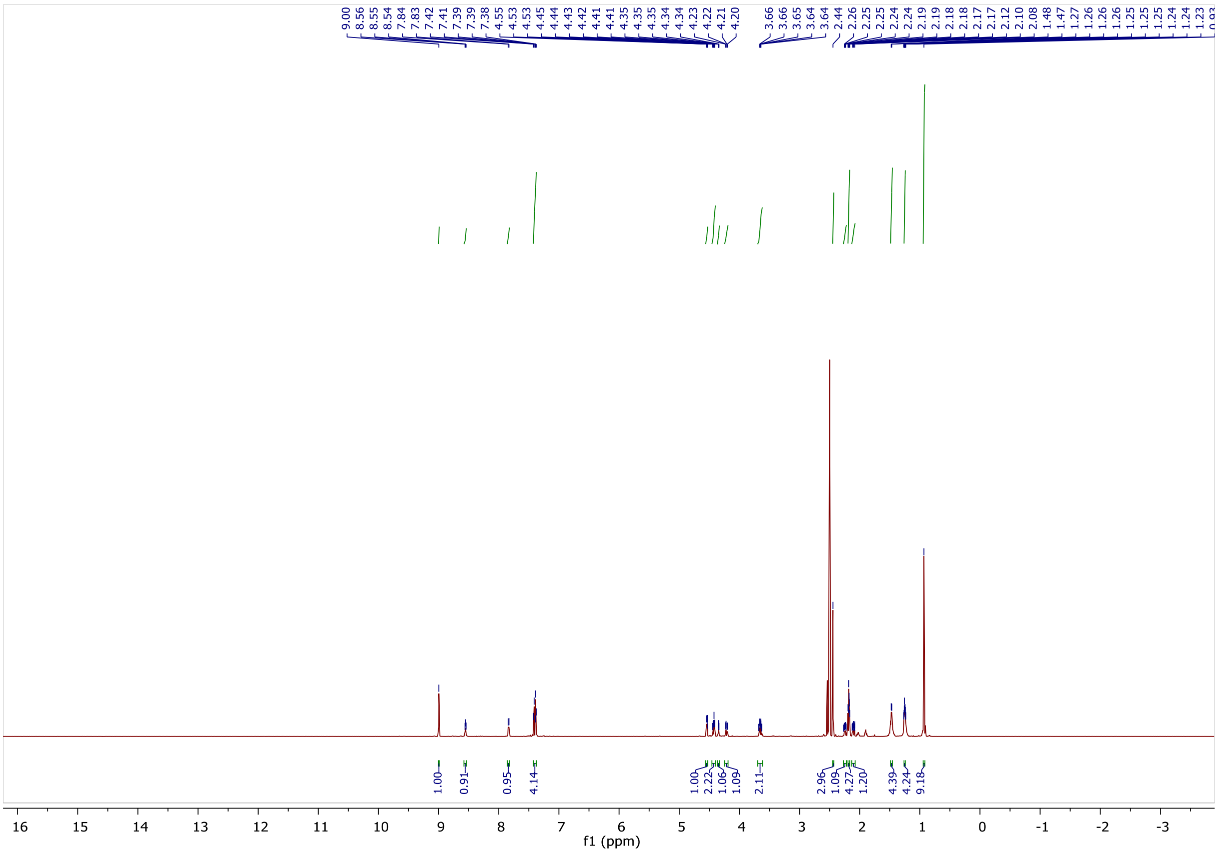

^{13}C NMR spectra of NK215.

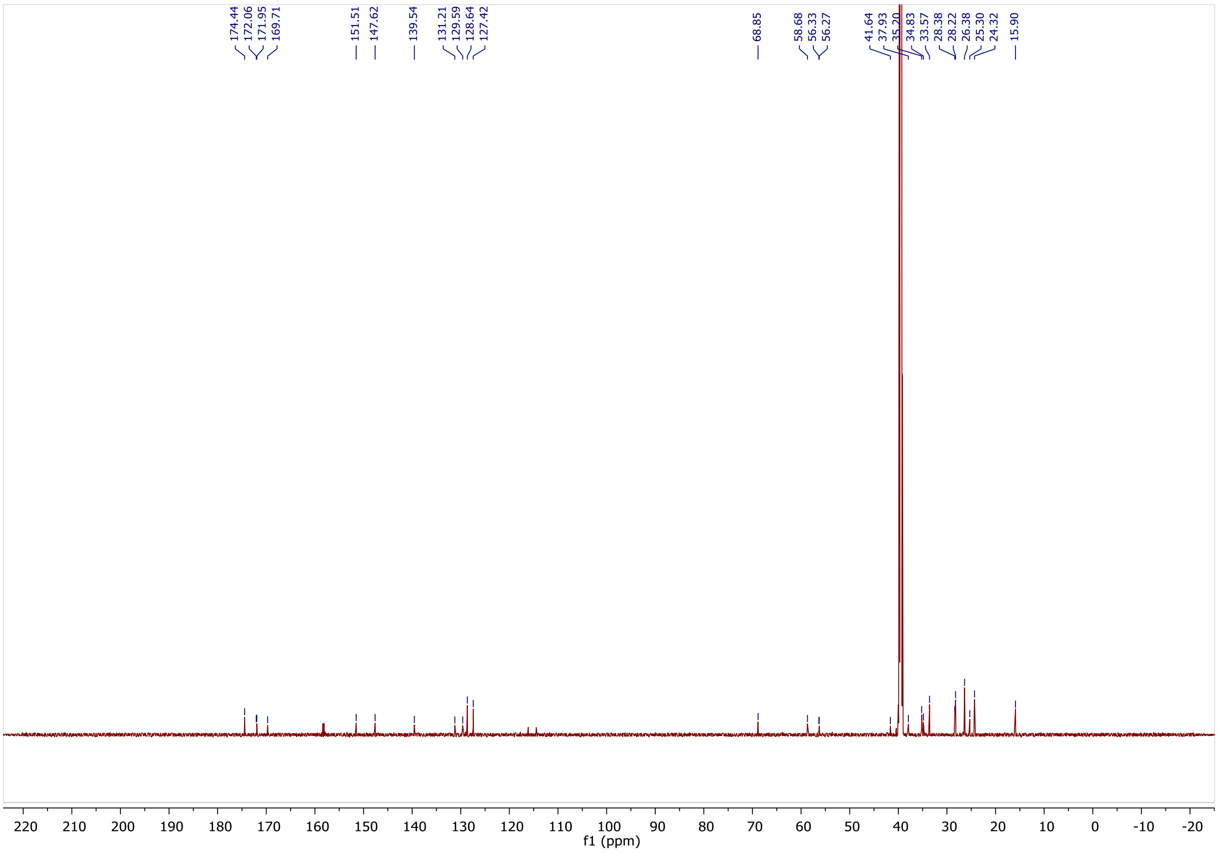

^{1}H NMR spectra of NK207.

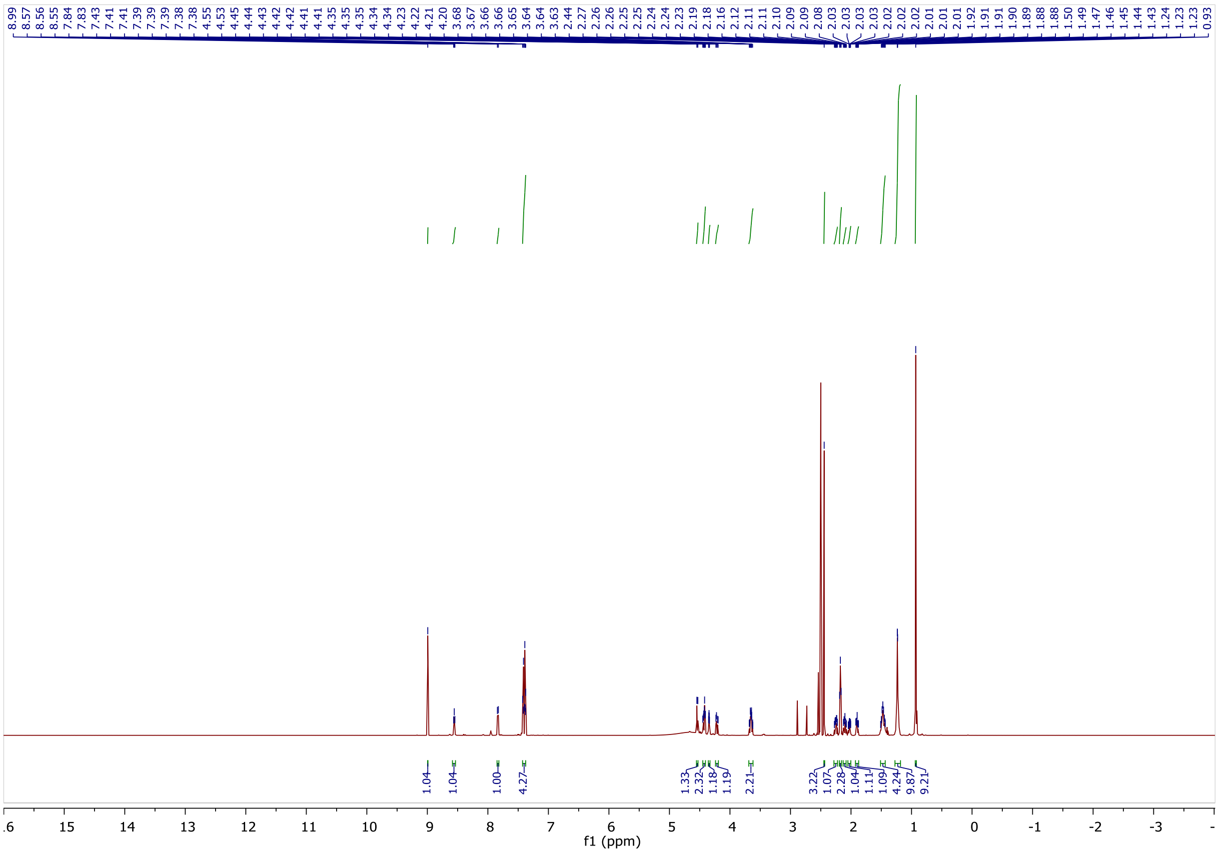

^{13}C NMR spectra of NK207.

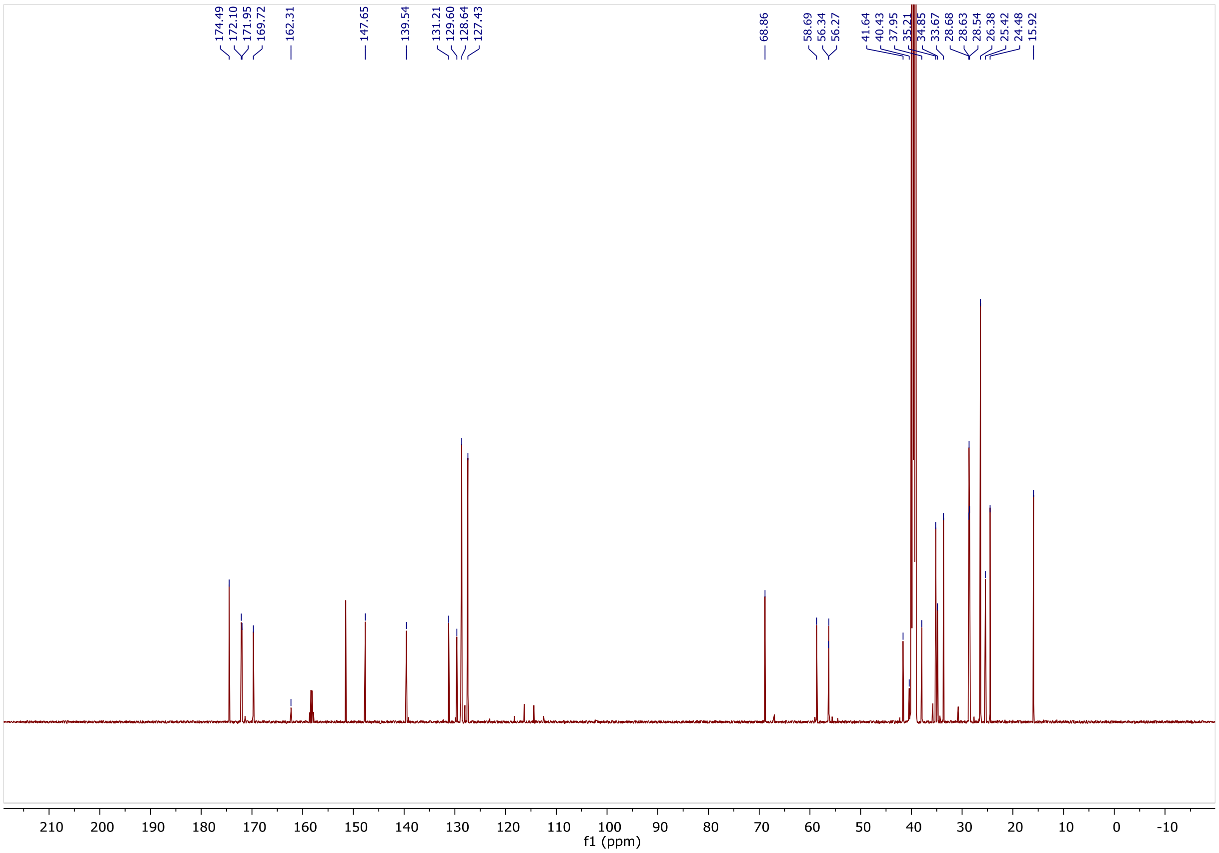

^{1}H NMR spectra of NK216.

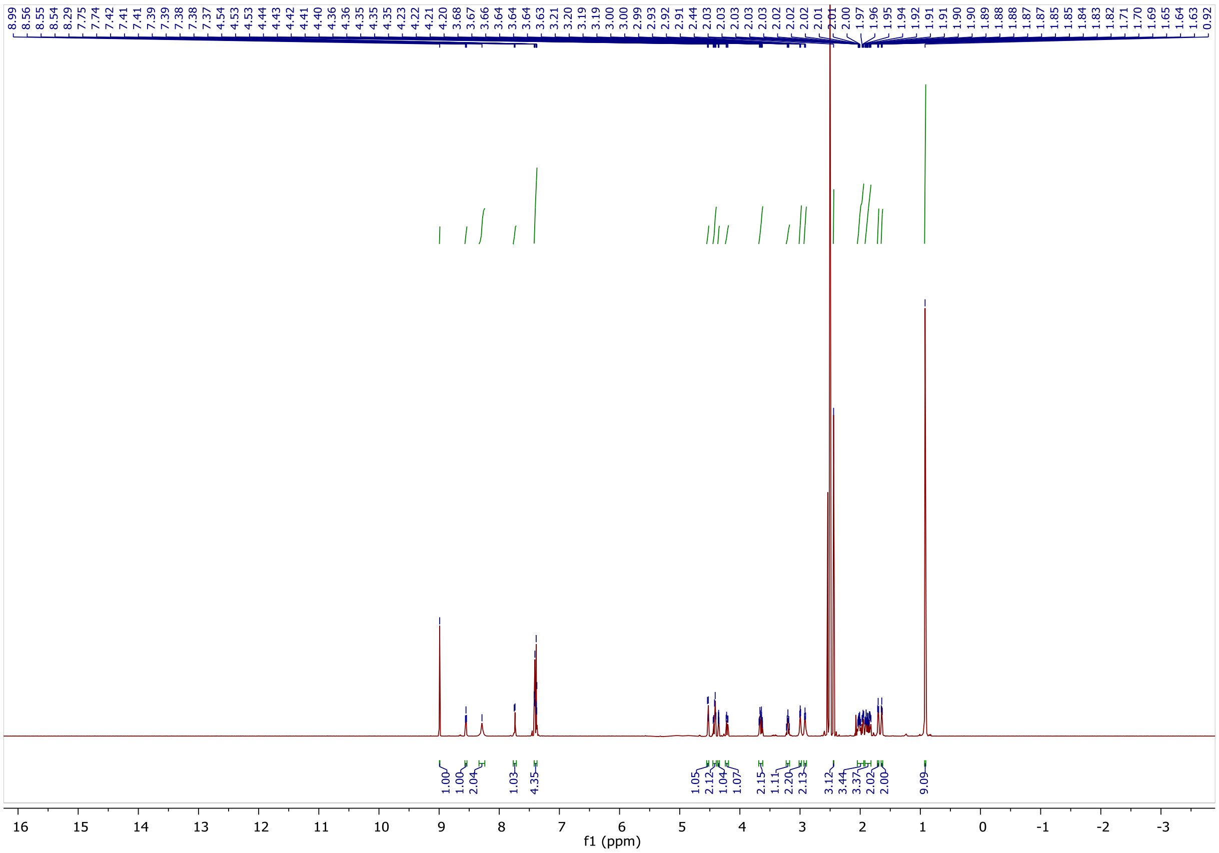

^{13}C NMR spectra of NK216.

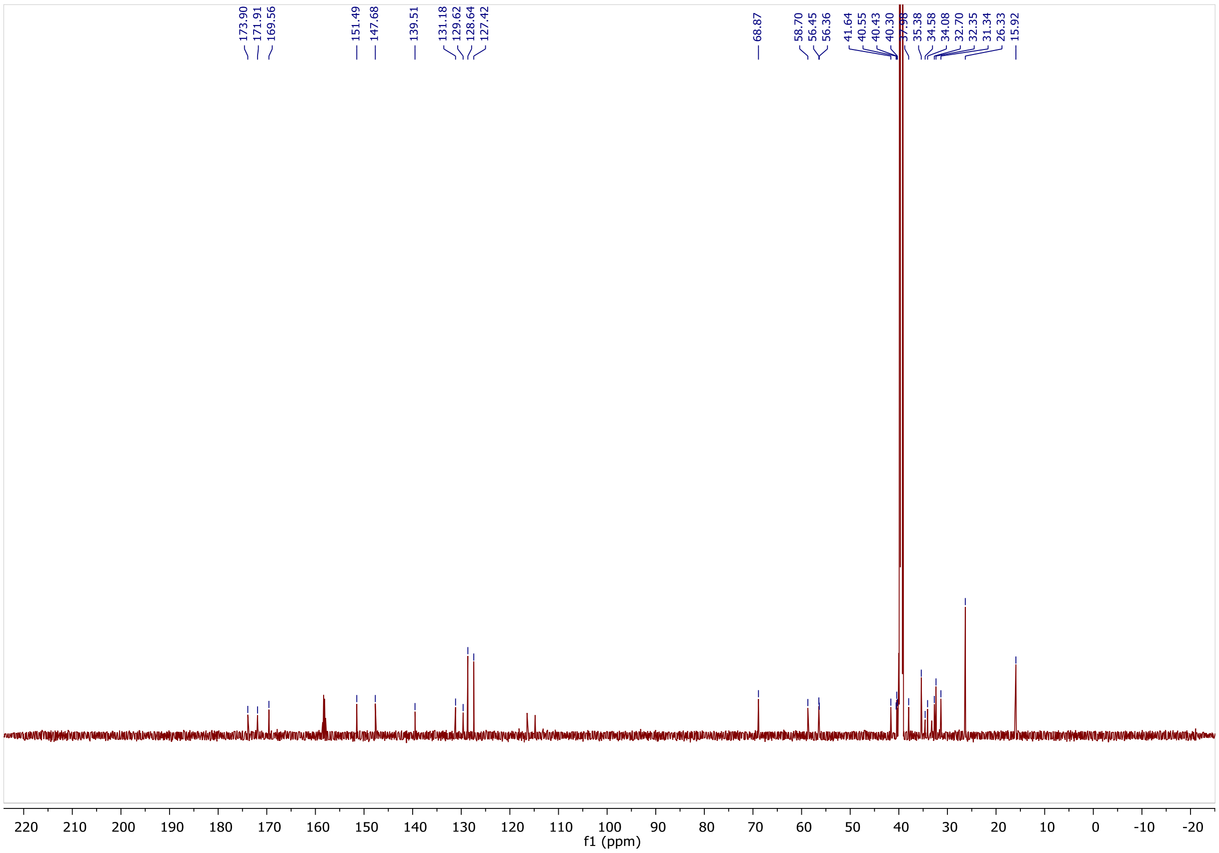

^{1}H NMR spectra of NK239.

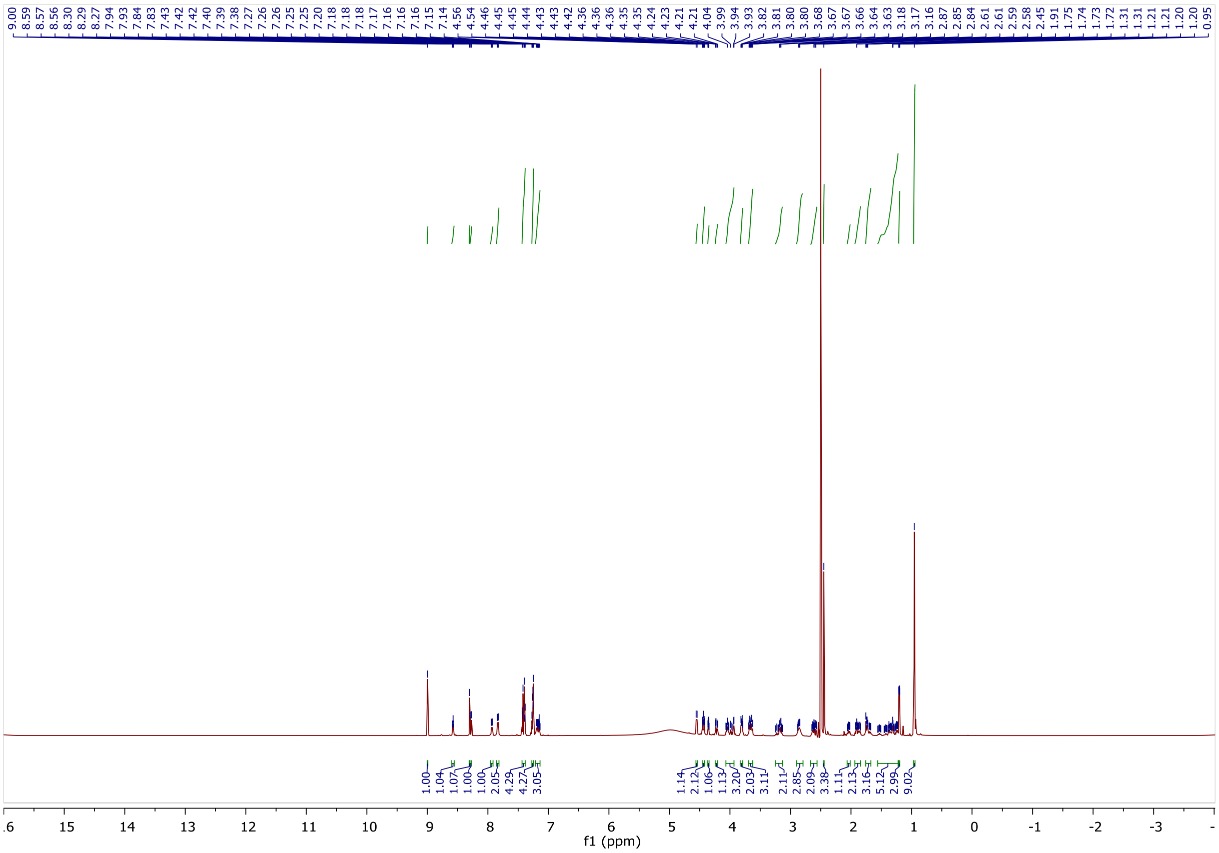

^{13}C NMR spectra of NK239.

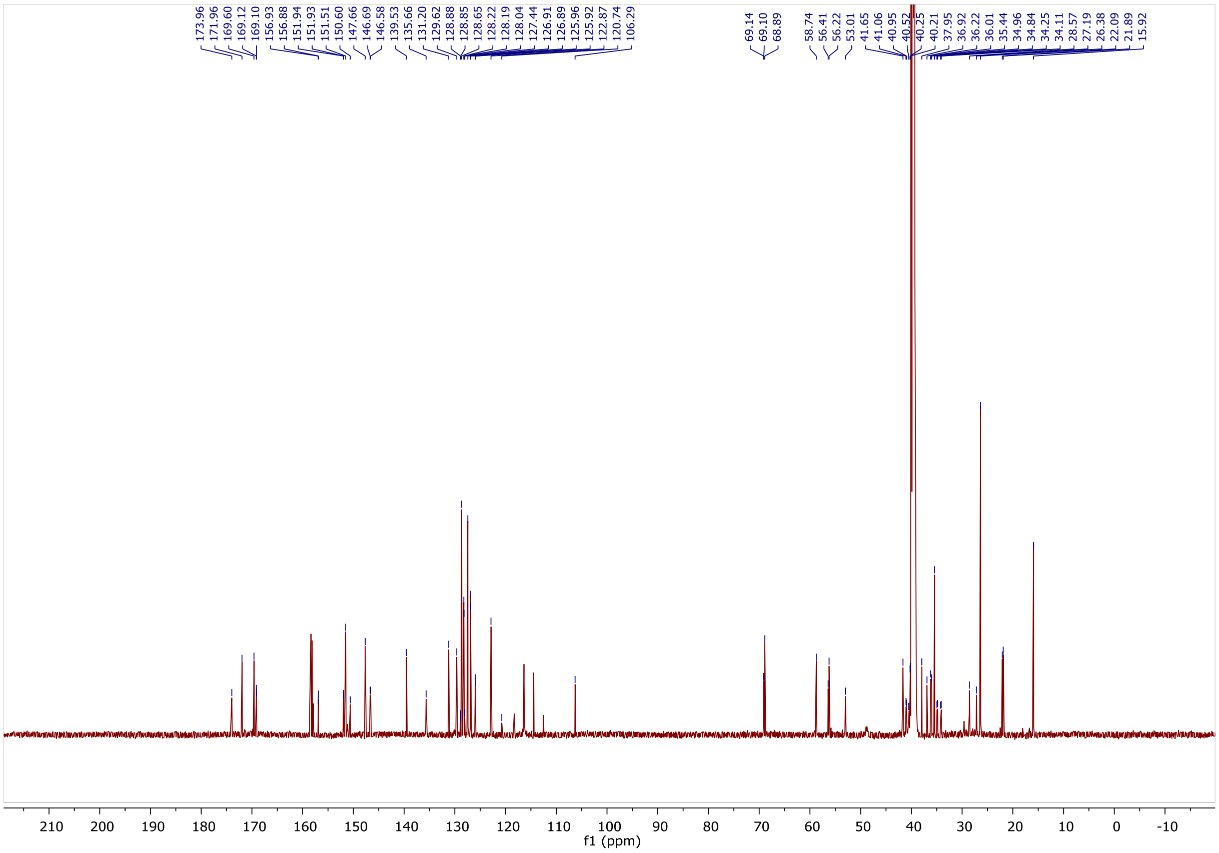

^{1}H NMR spectra of NK240.

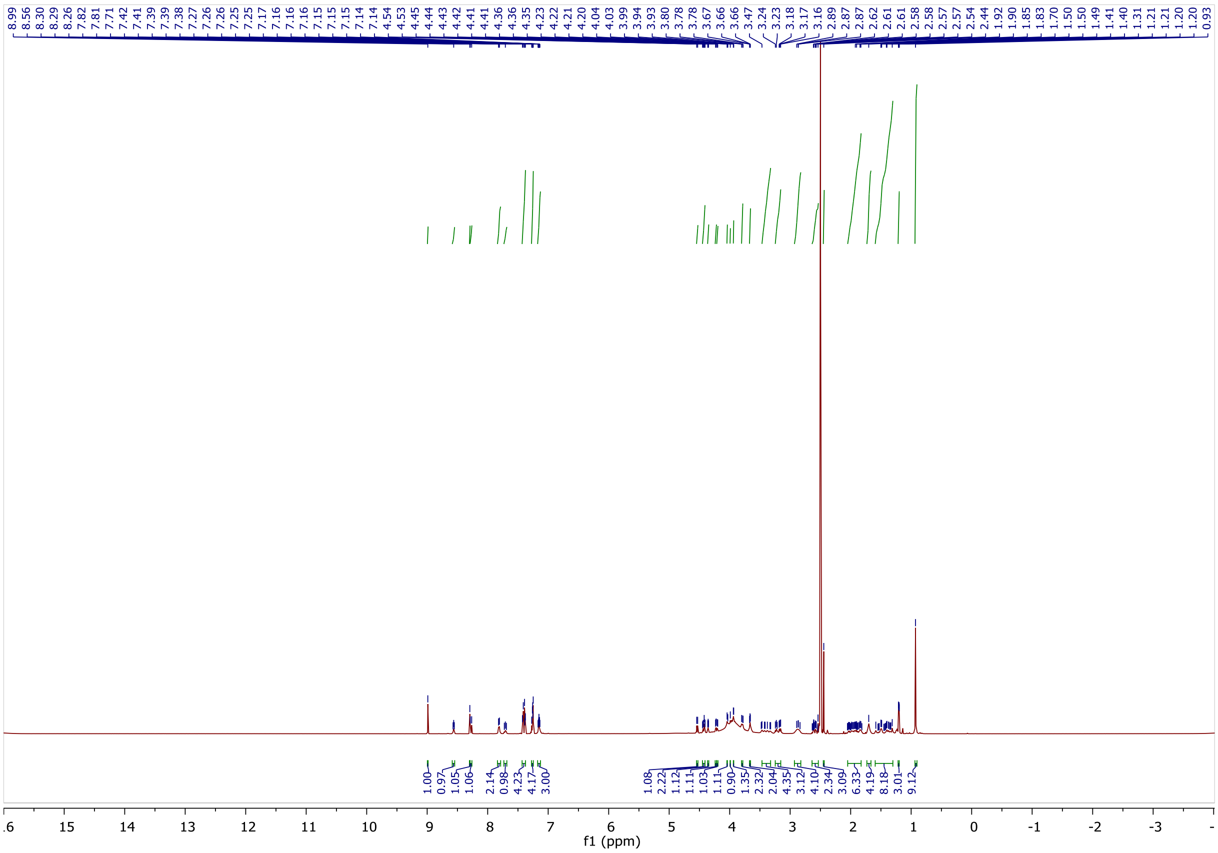

^{13}C NMR spectra of NK240.

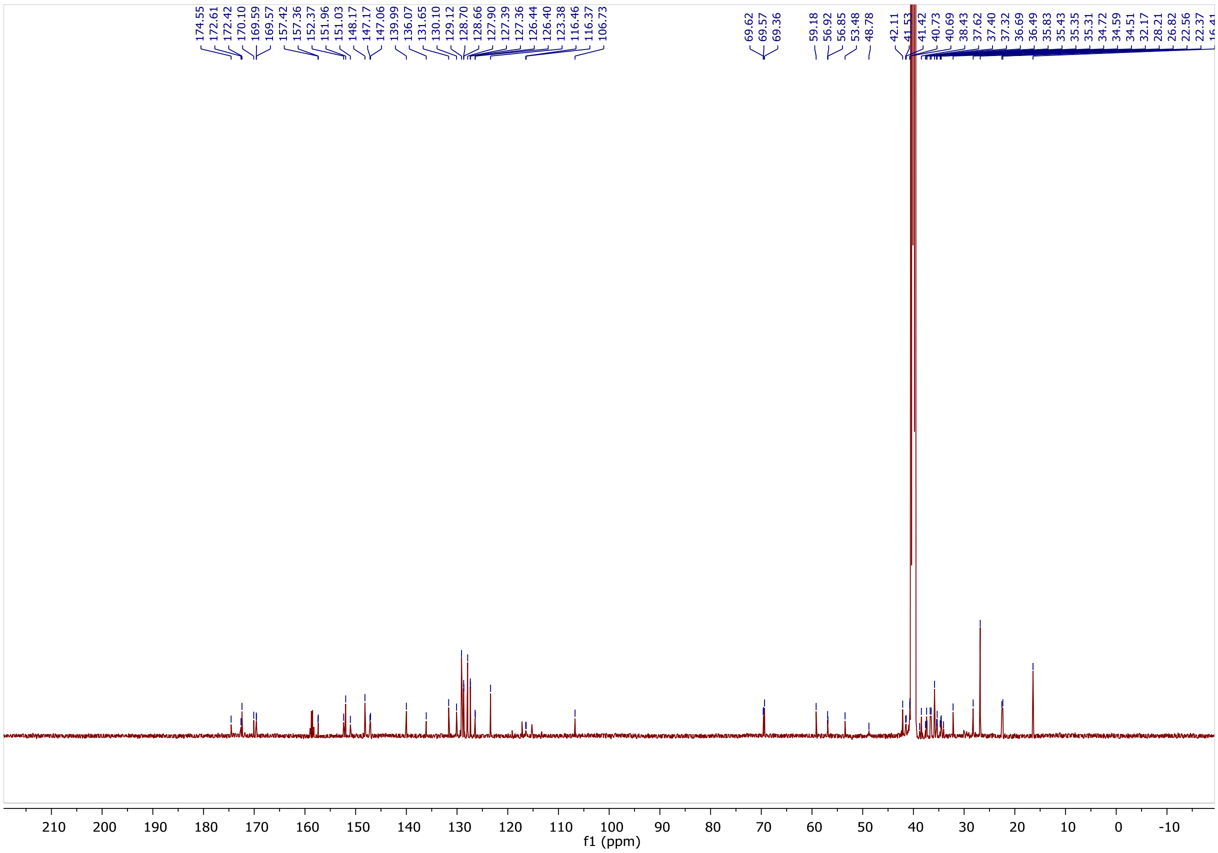

^{1}H NMR spectra of NK237.

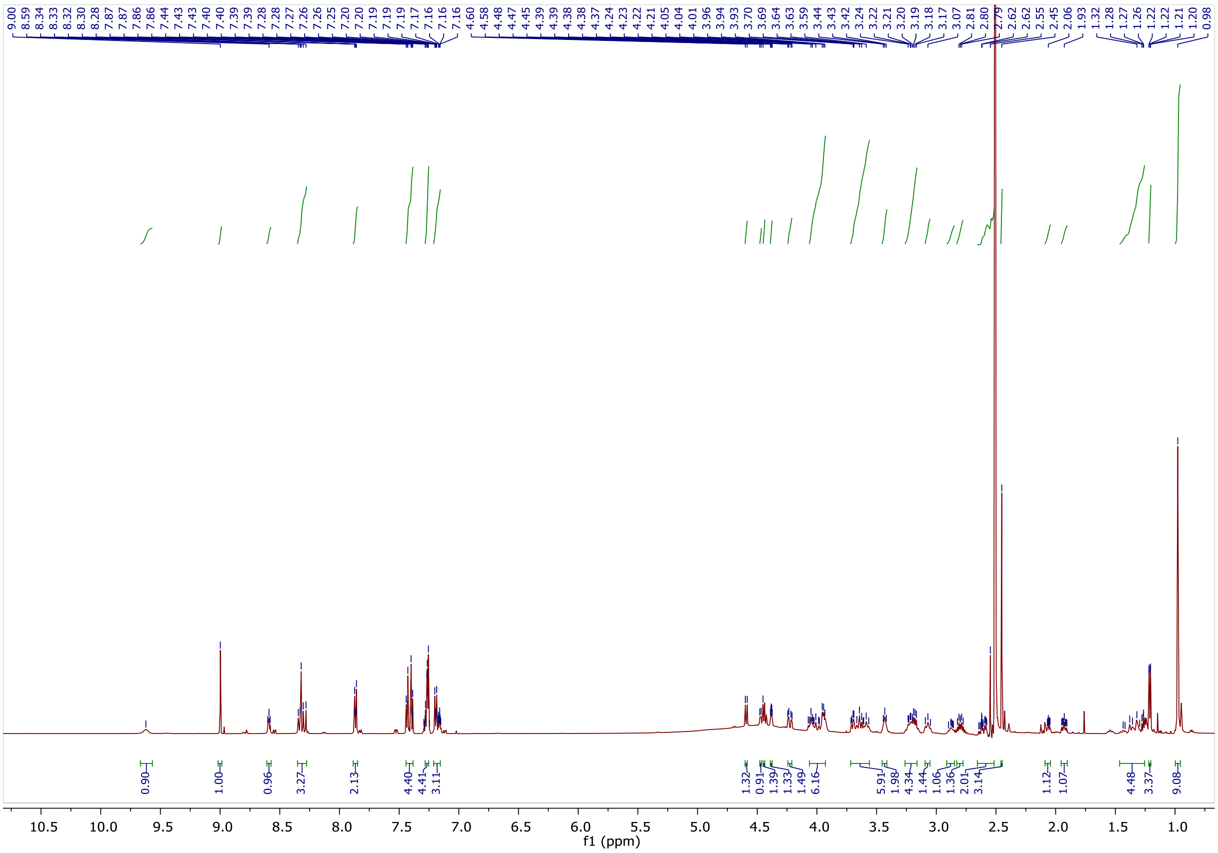

^{13}C NMR spectra of NK237.

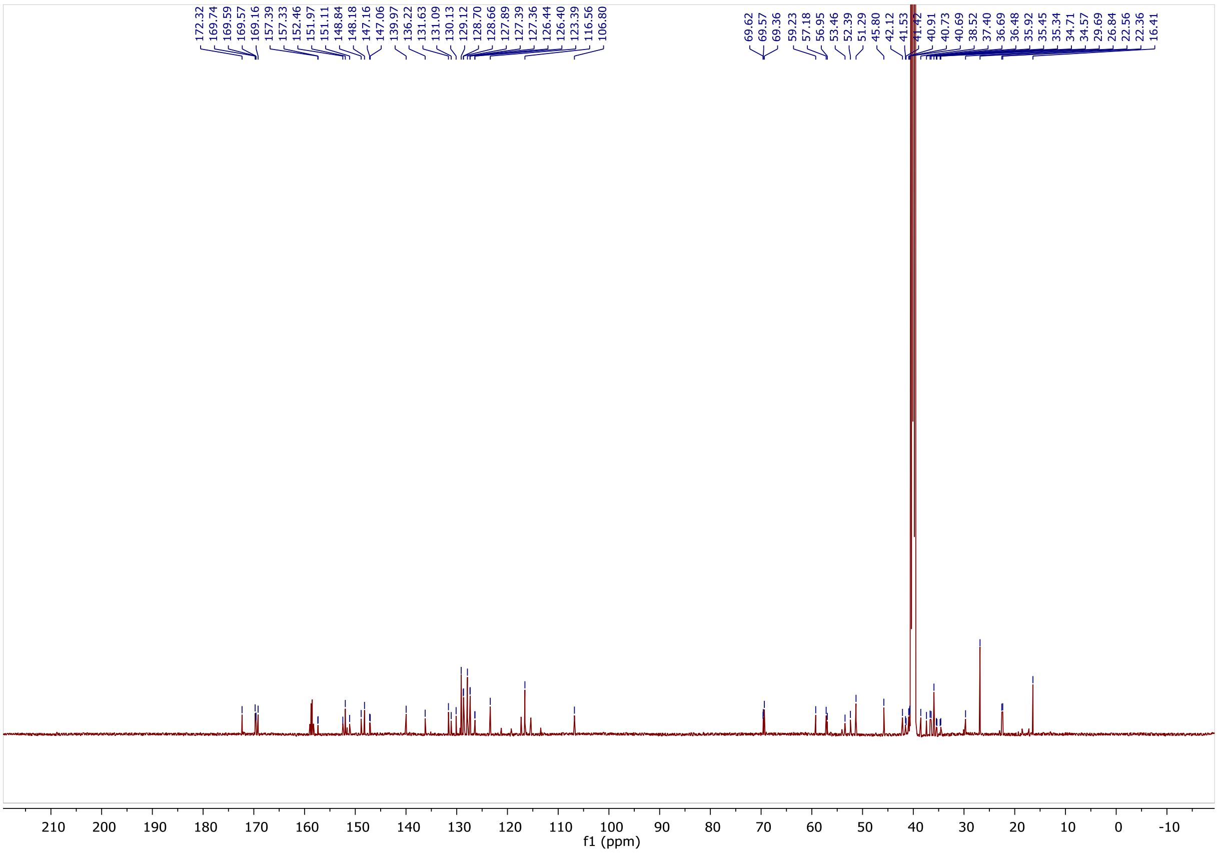

^{1}H NMR spectra of NK238.

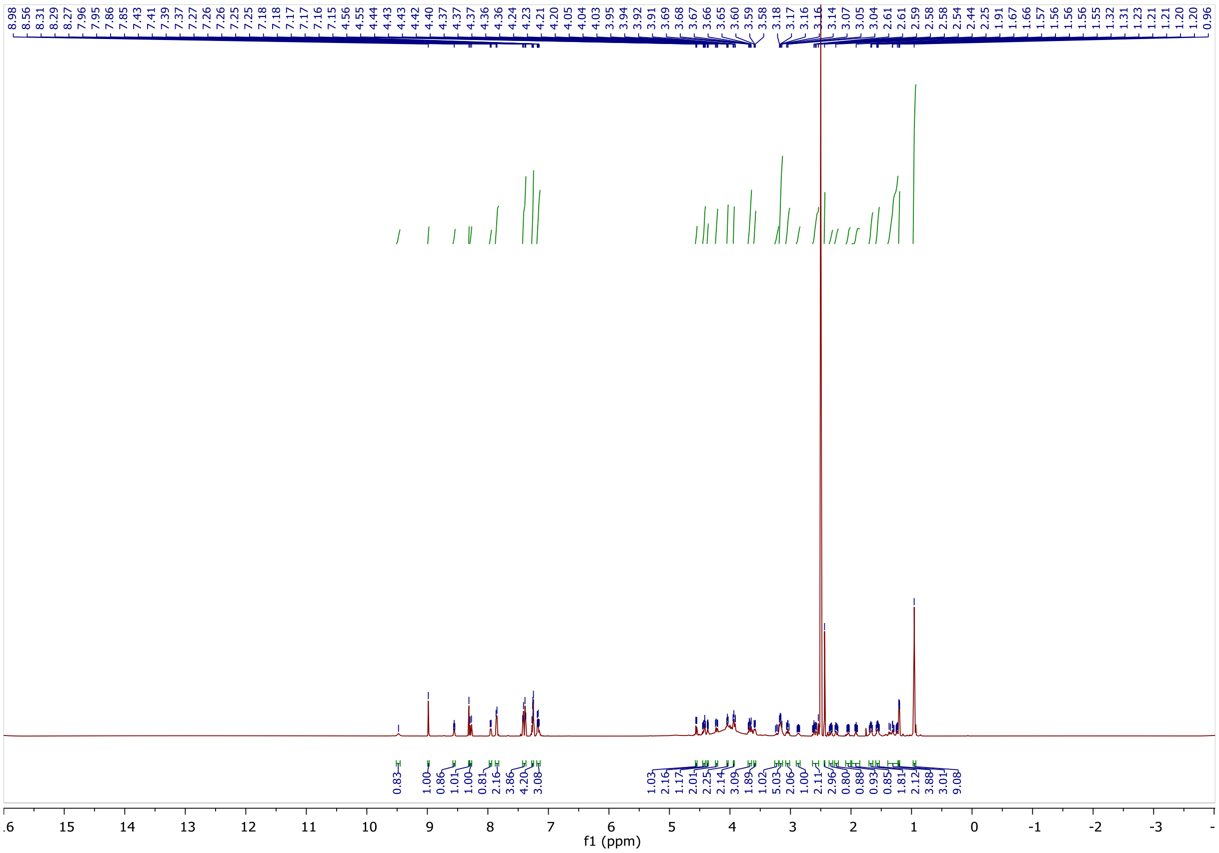

^{13}C NMR spectra of NK238.

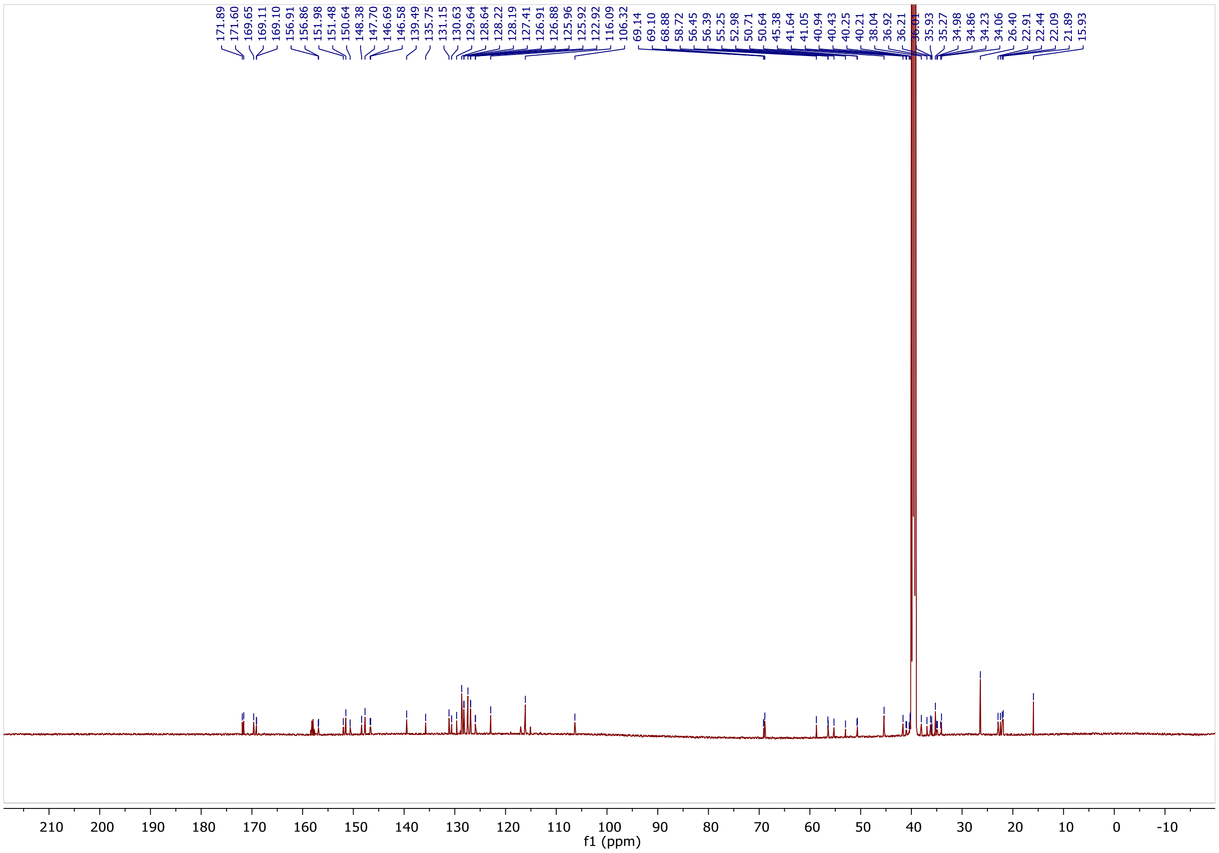

^{1}H NMR spectra of NK242.

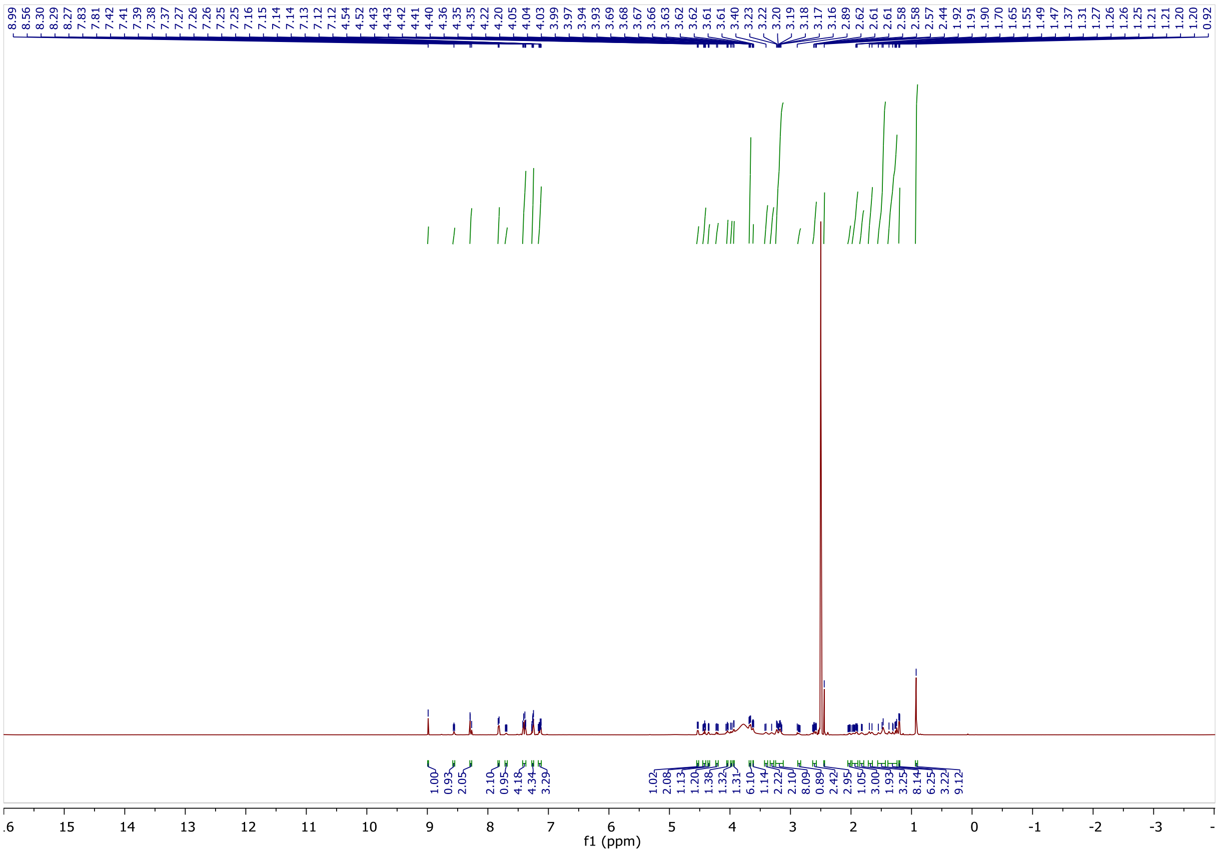

^{13}C NMR spectra of NK242.

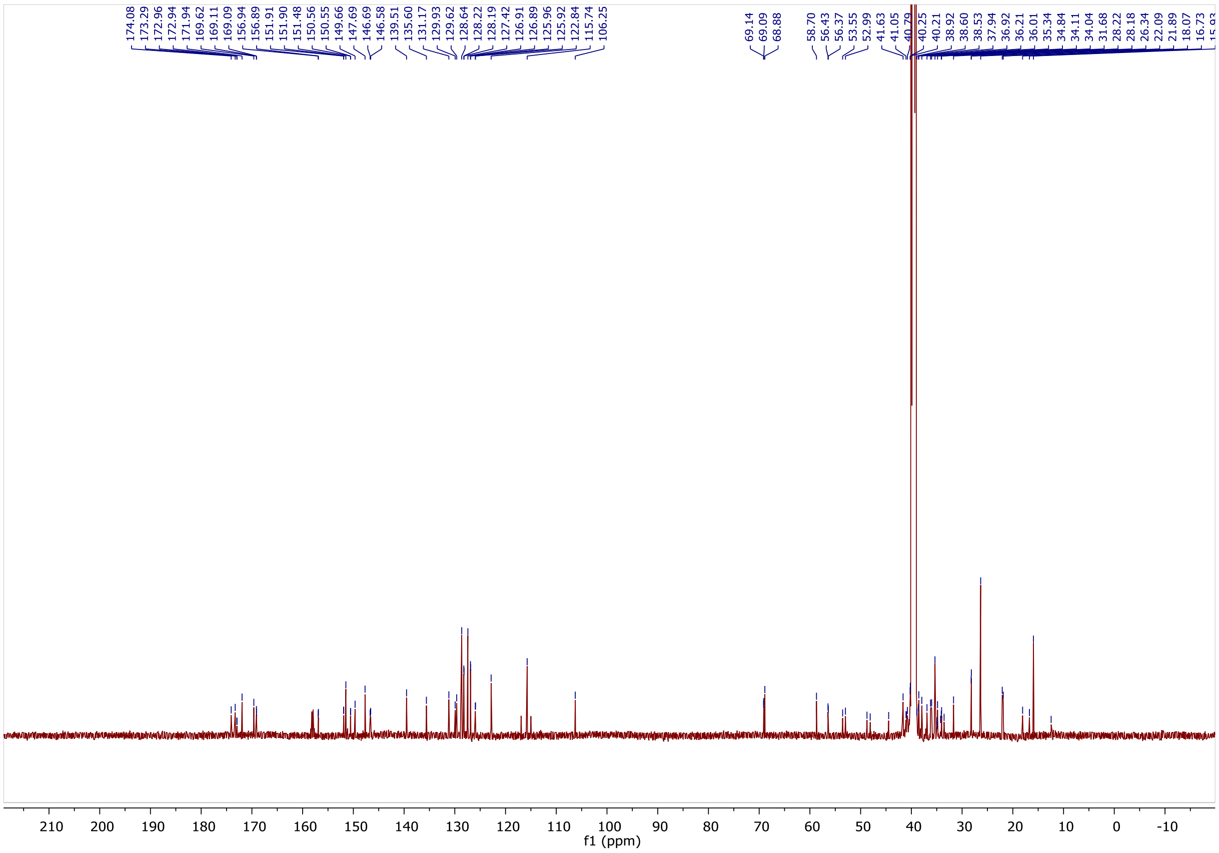

^{1}H NMR spectra of NK245.

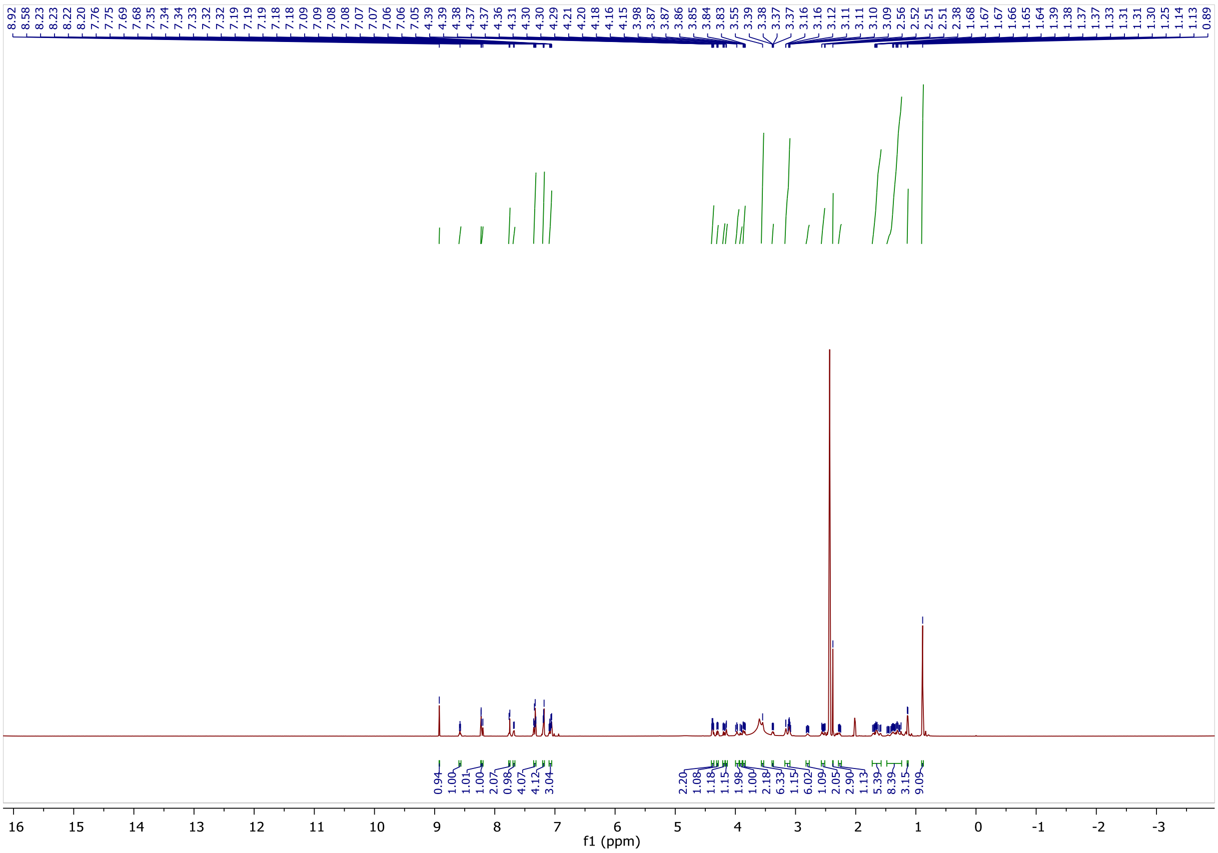

^{13}C NMR spectra of NK245.

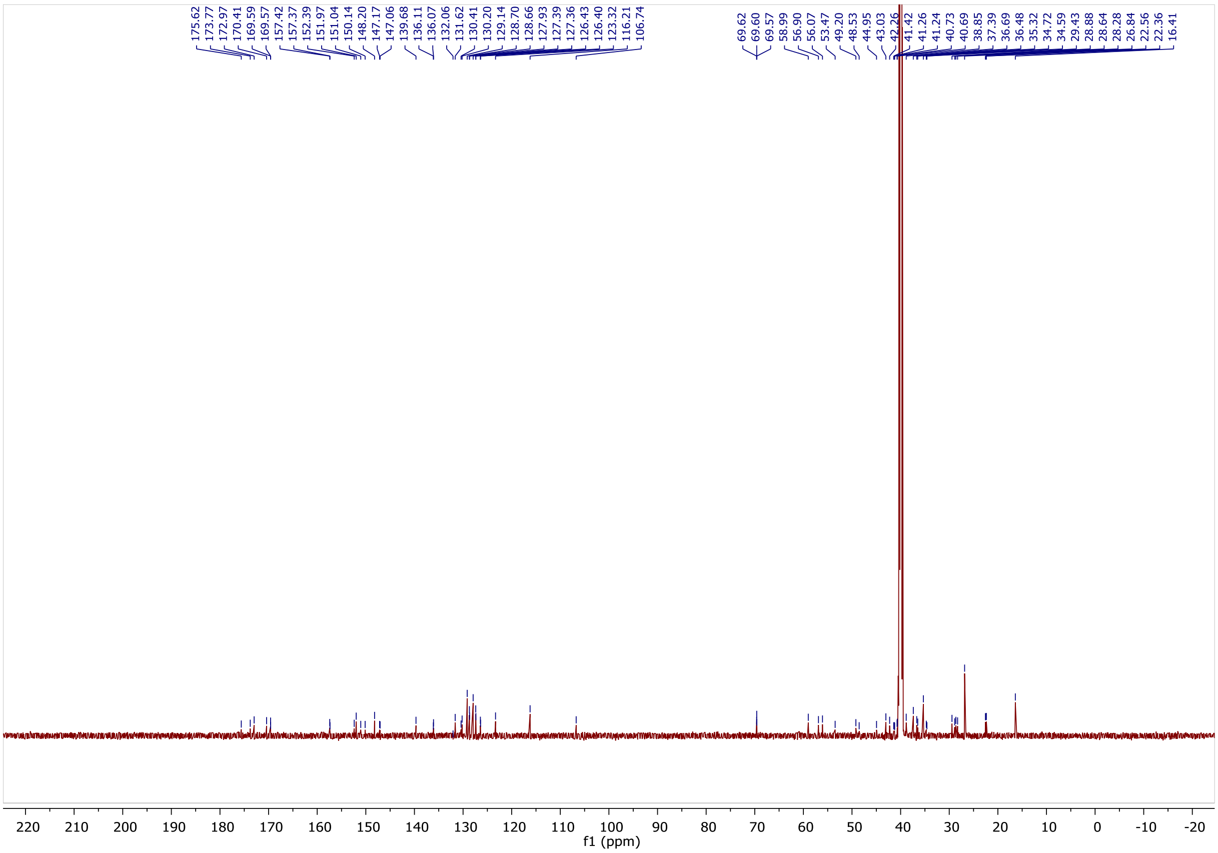

^{1}H NMR spectra of NK221.

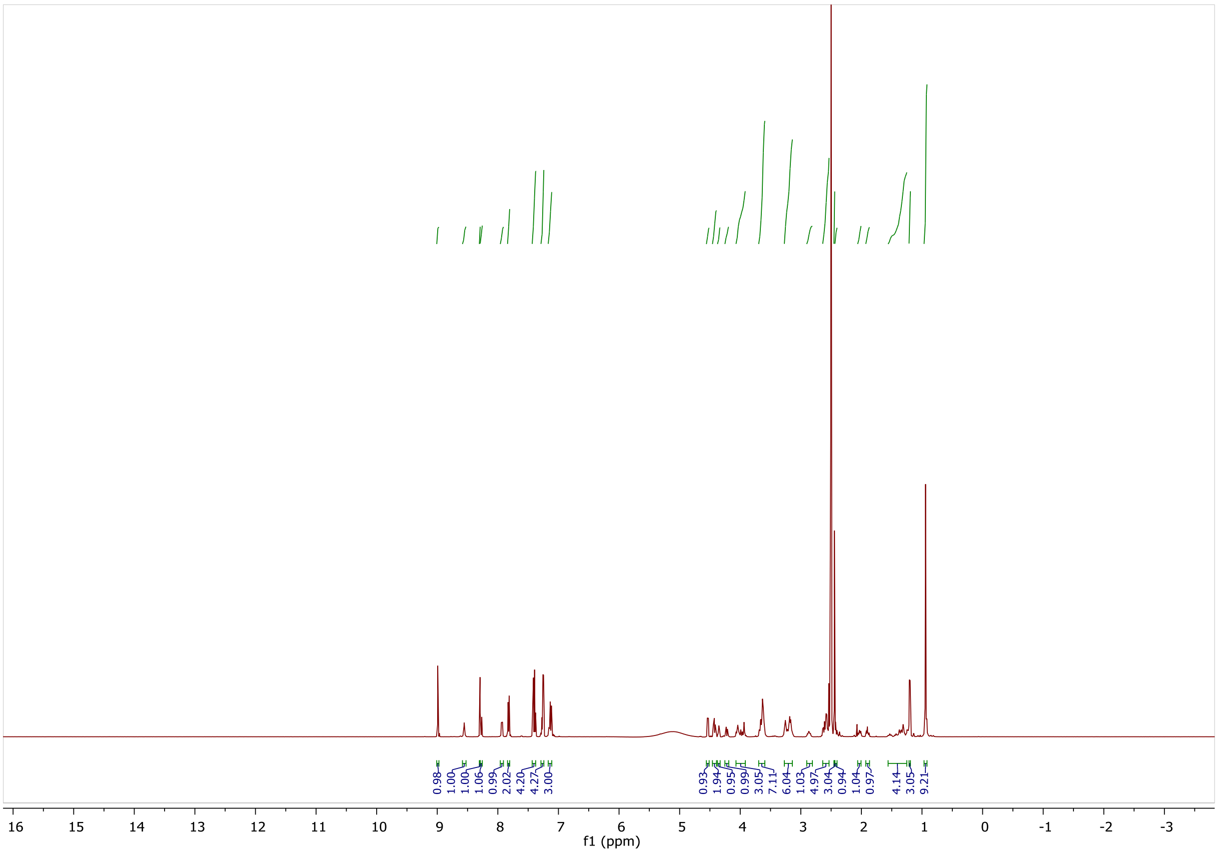

^{13}C NMR spectra of NK221.

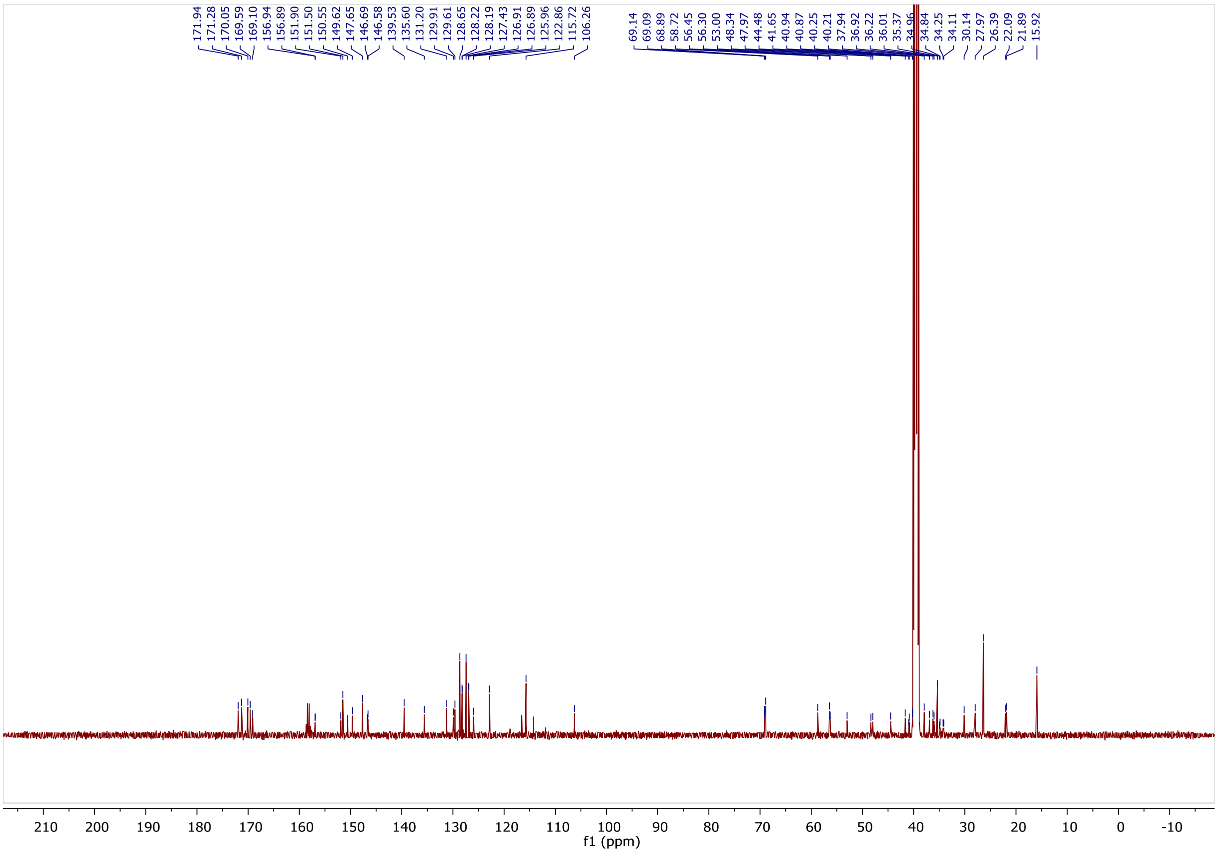

^{1}H NMR spectra of NK222.

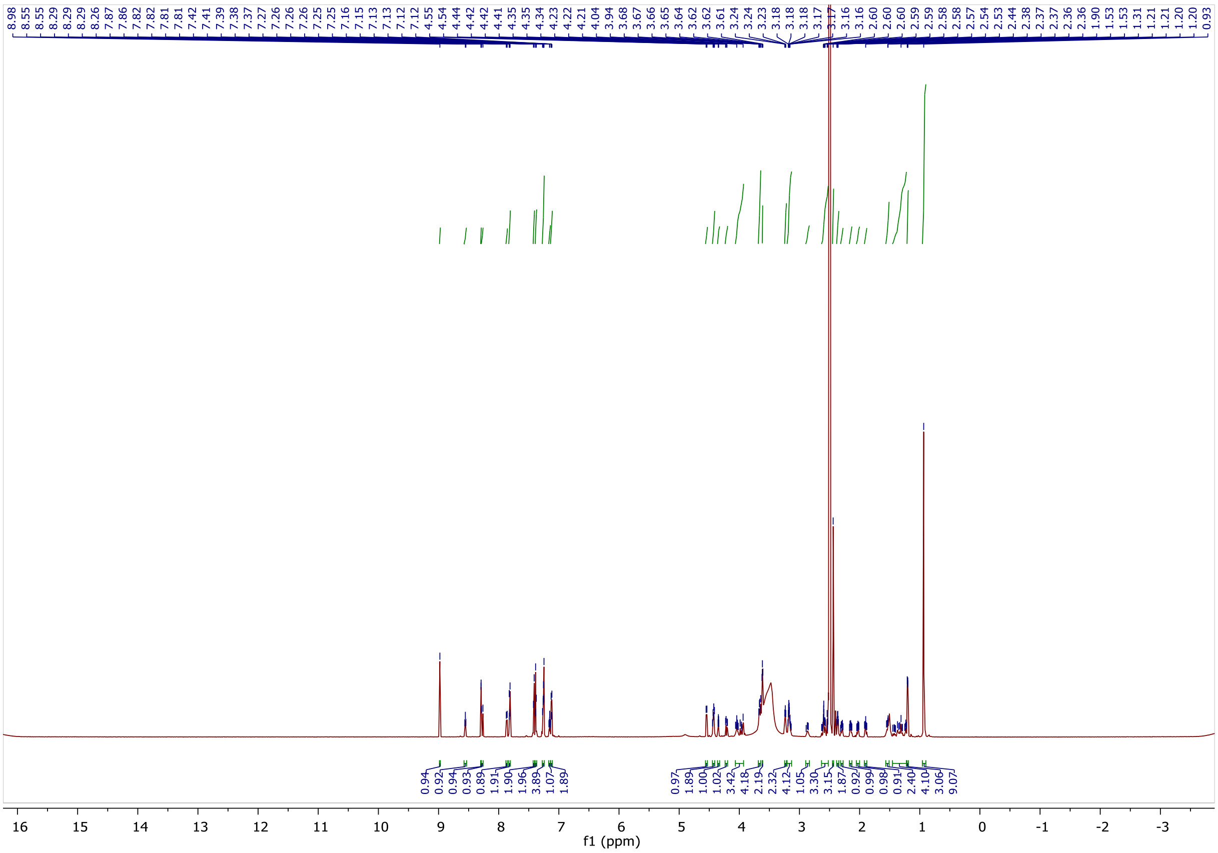

^{13}C NMR spectra of NK222.

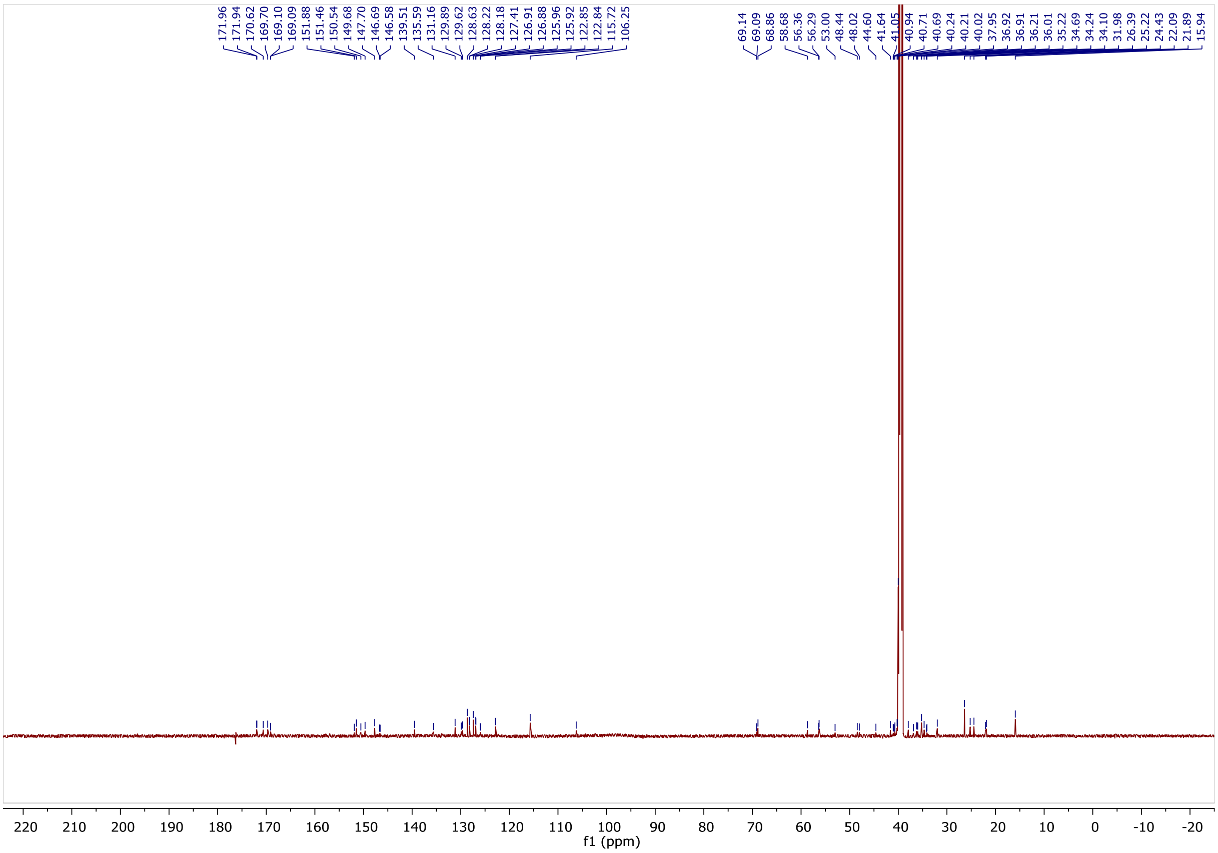

^{1}H NMR spectra of NK223.

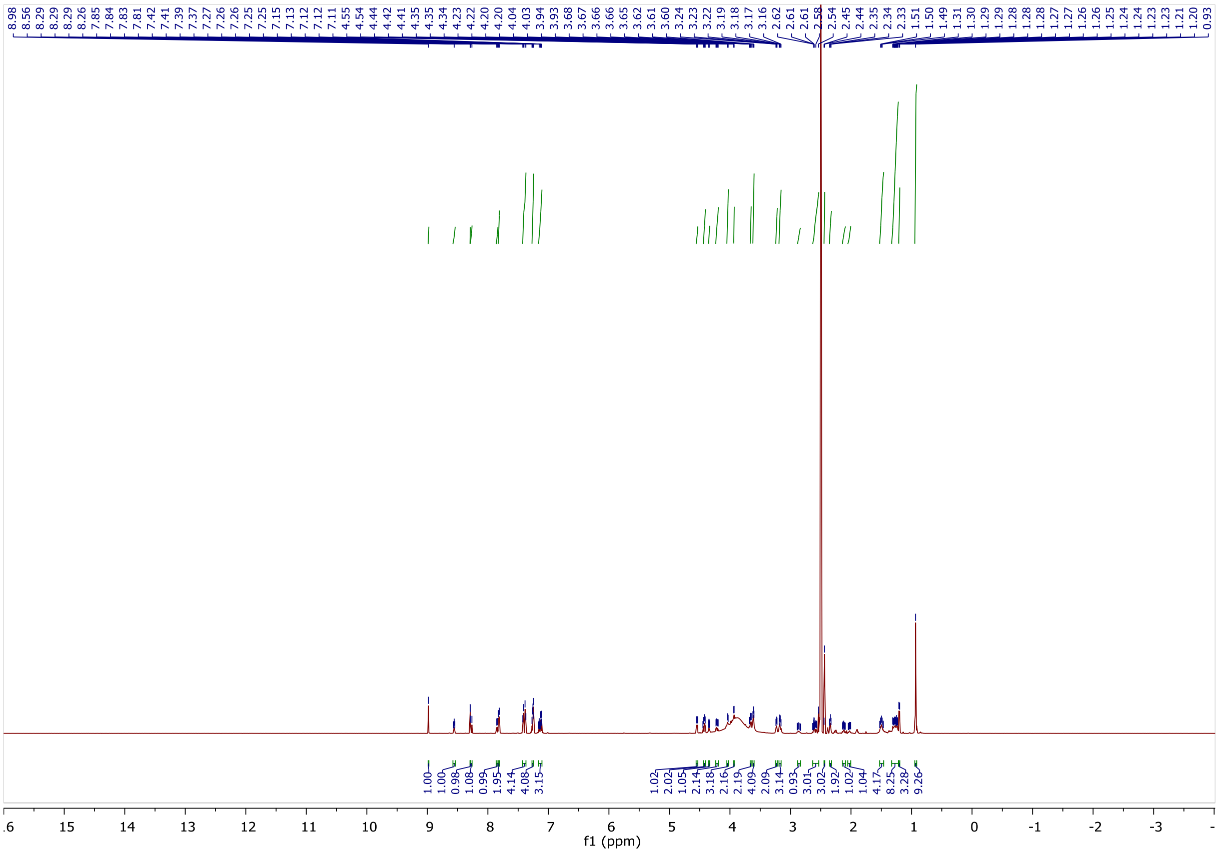

^{13}C NMR spectra of NK223.

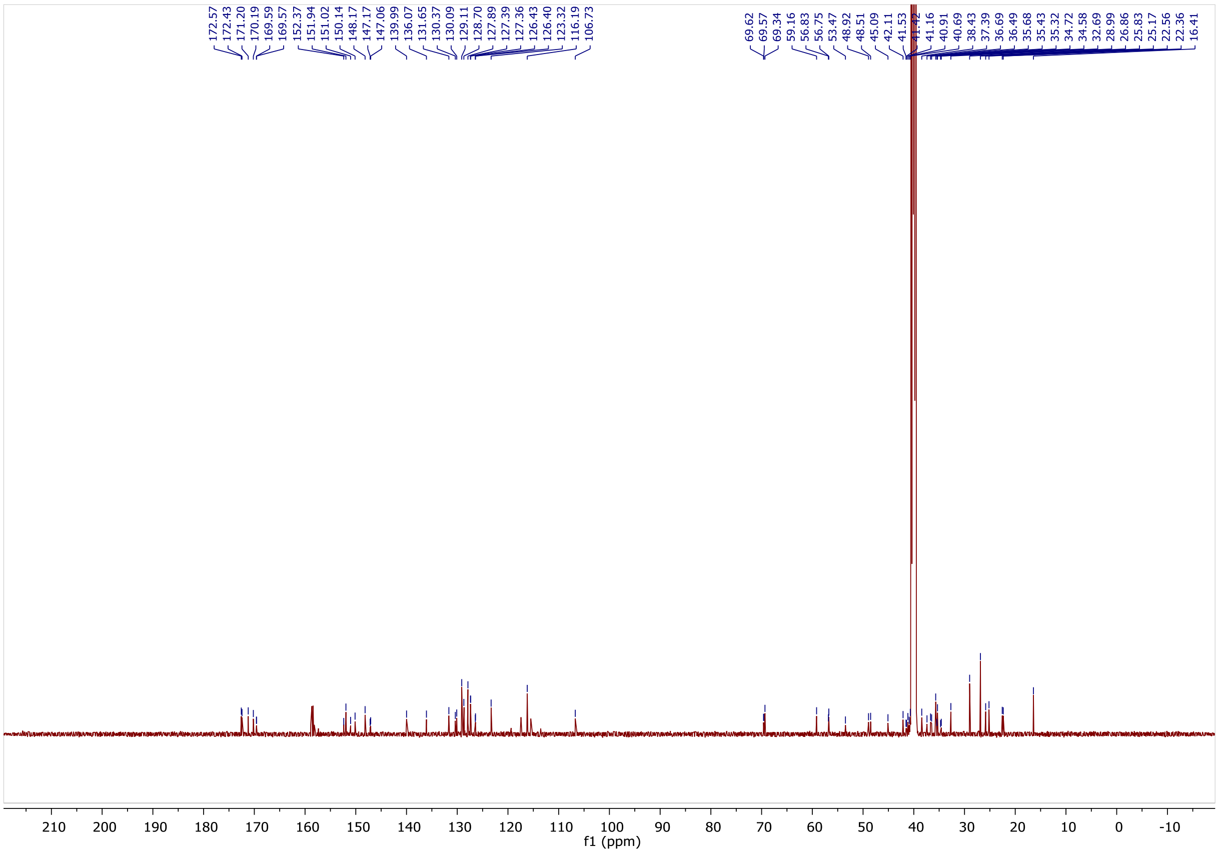

^{1}H NMR spectra of NK224.

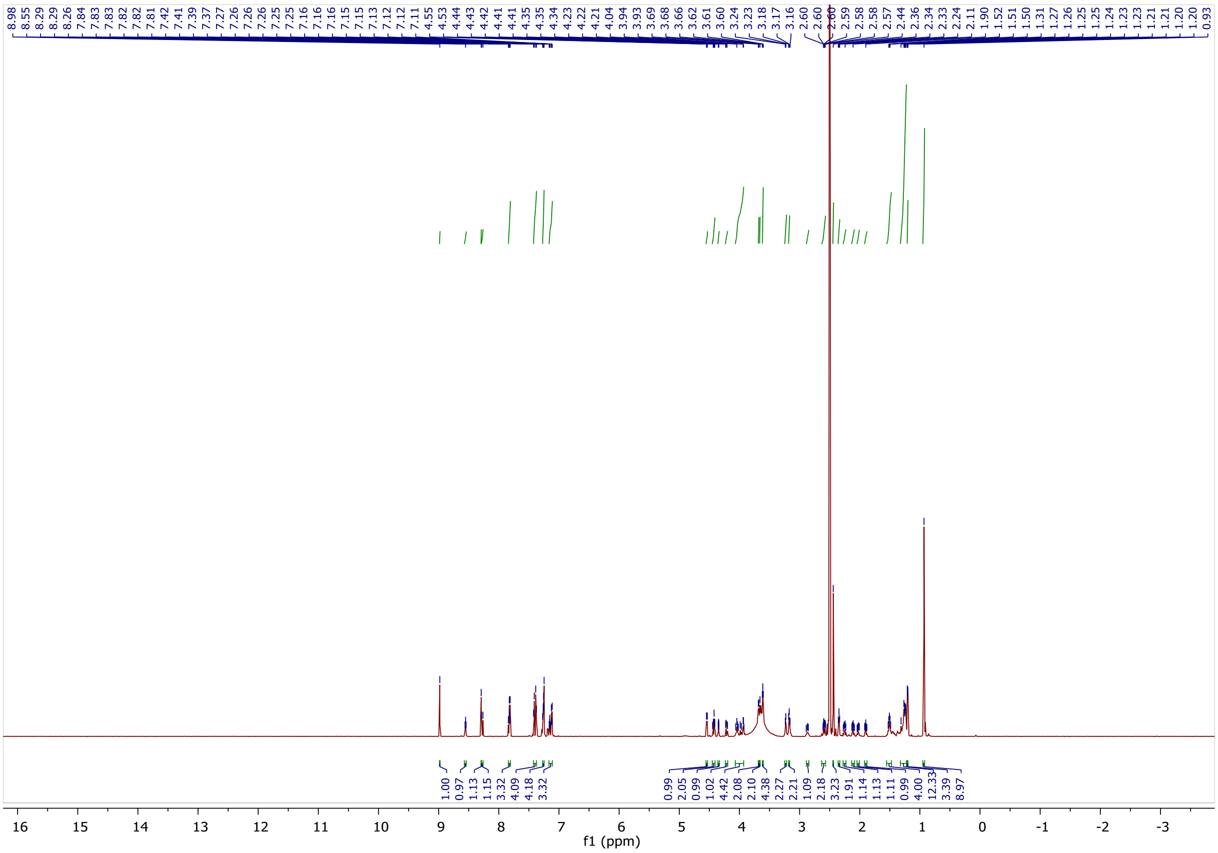

^{13}C NMR spectra of NK224.

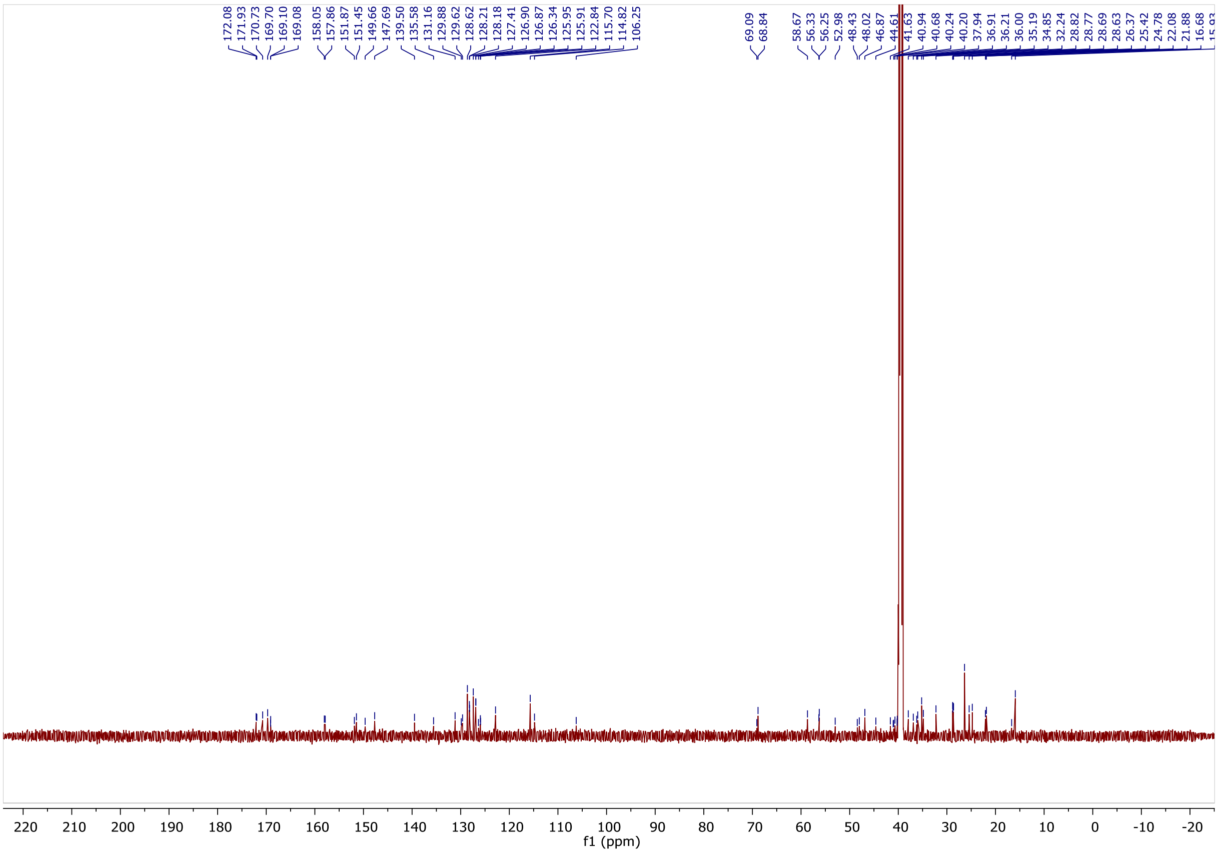

^{1}H NMR spectra of NK225.

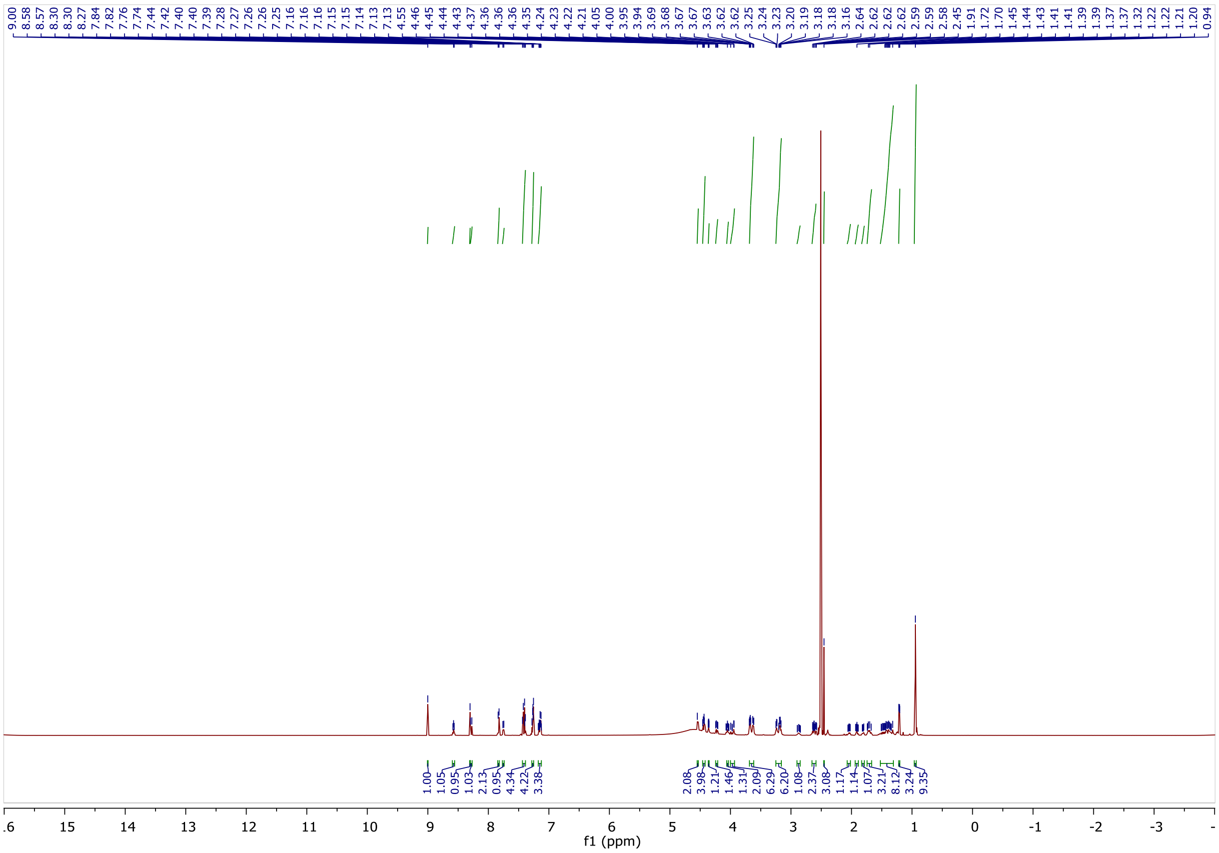

^{13}C NMR spectra of NK225.

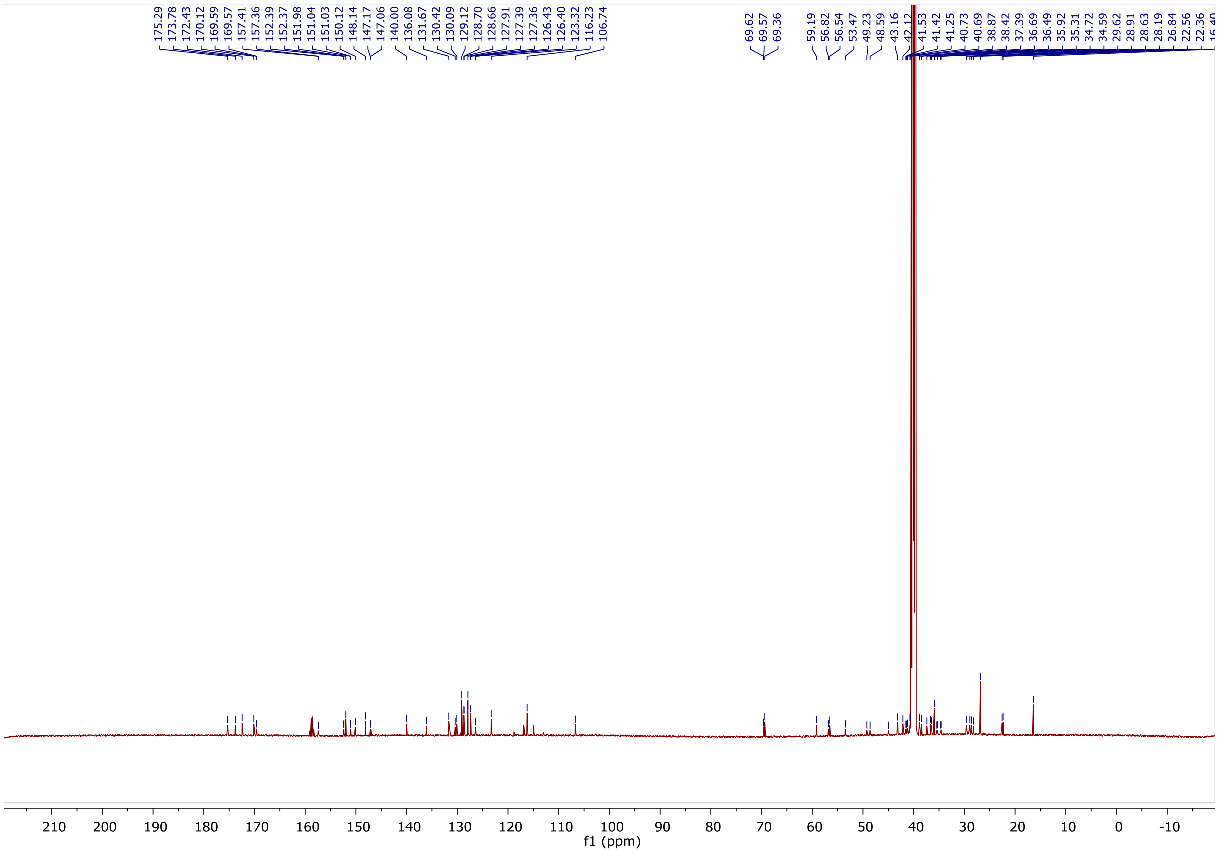

^{1}H NMR spectra of NK228.

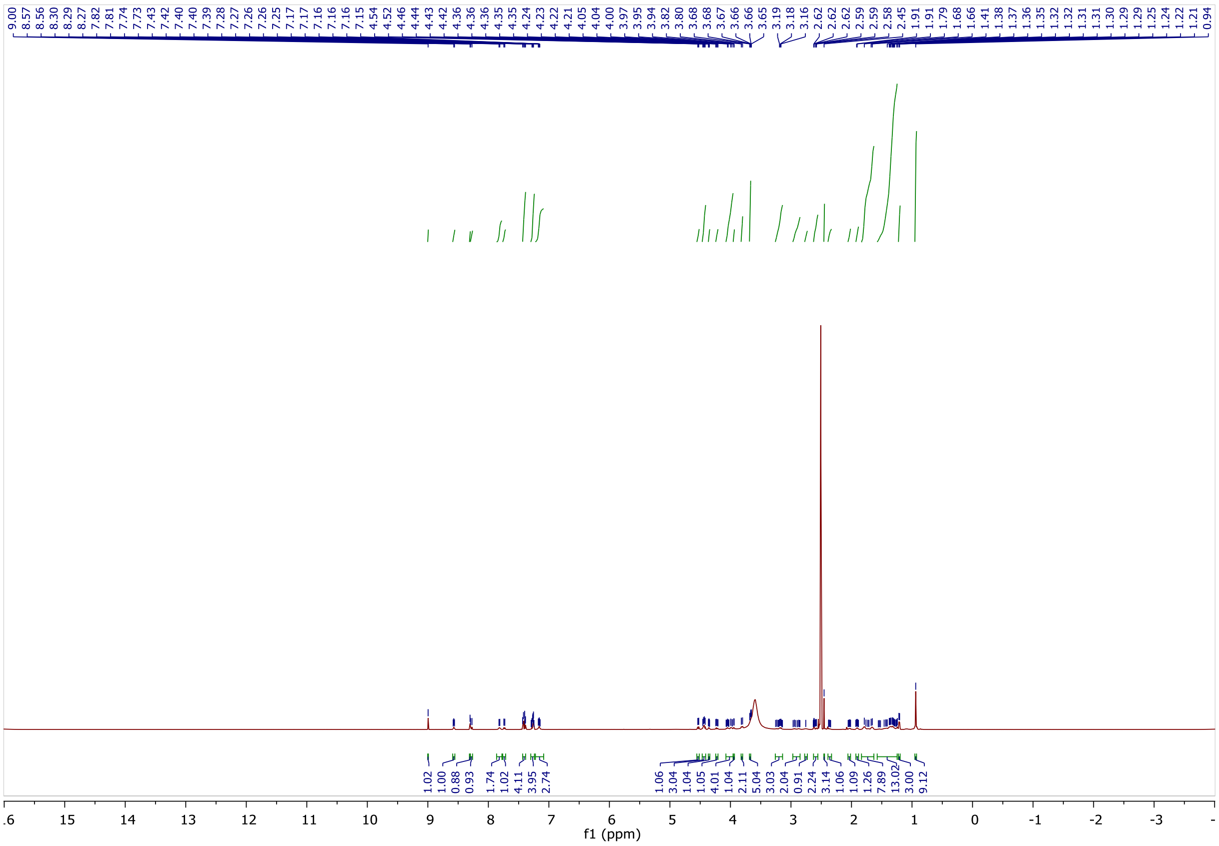

^{13}C NMR spectra of NK228.

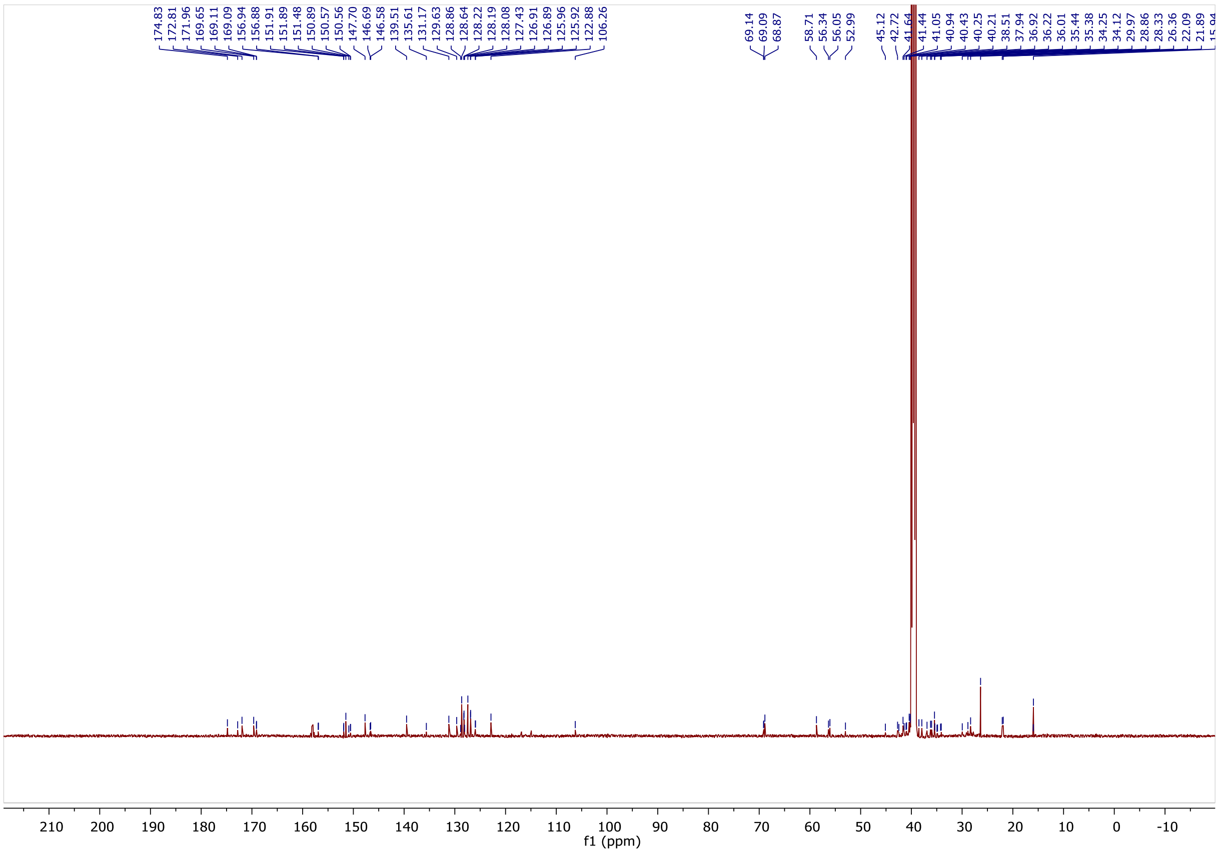

^{1}H NMR spectra of NK230.

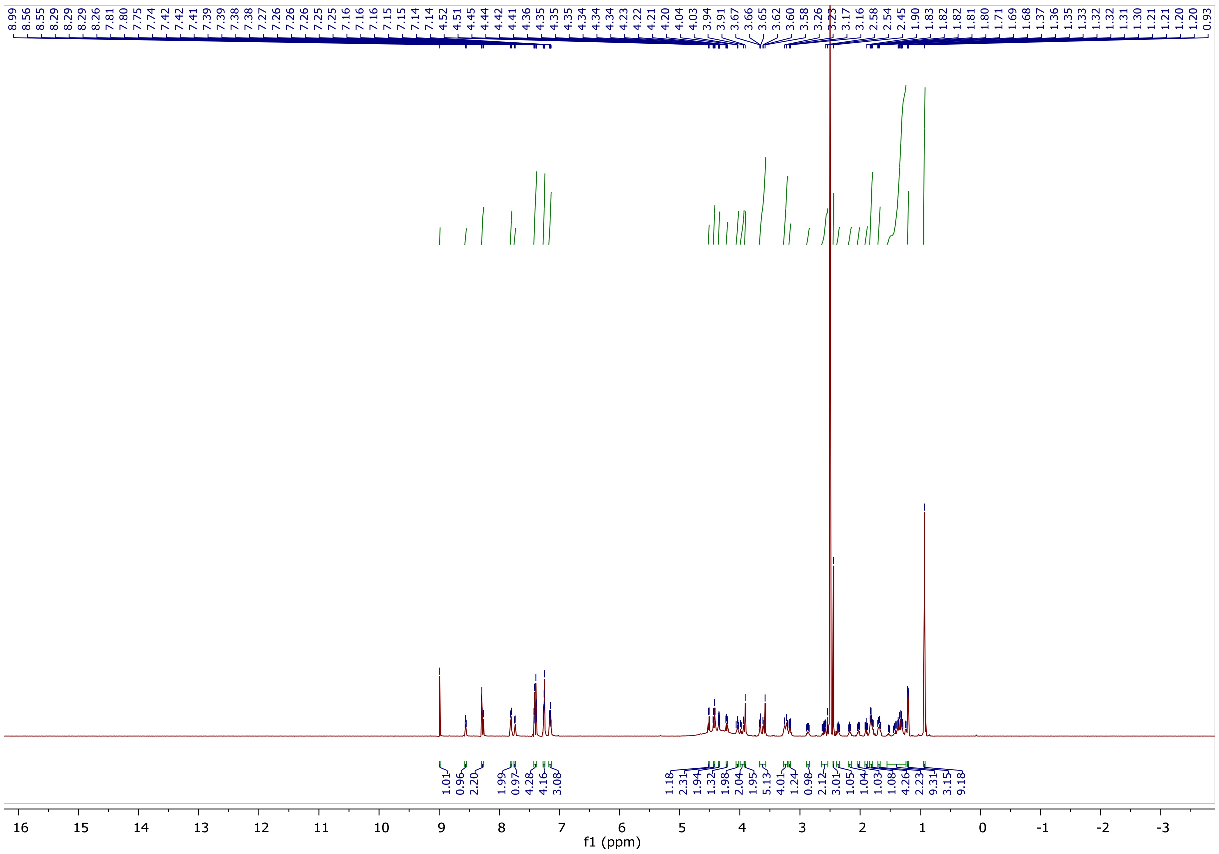

^{13}C NMR spectra of NK230.

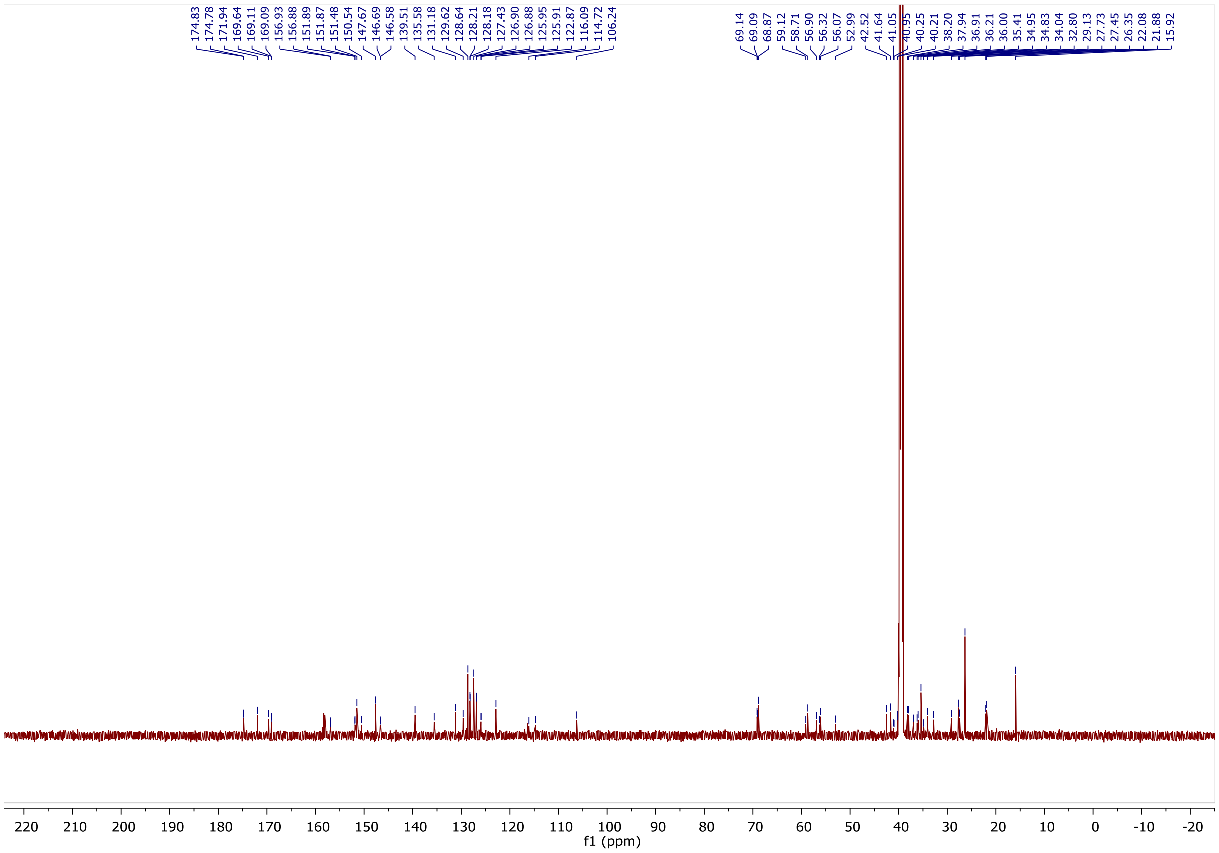

^{1}H NMR spectra of NK232.

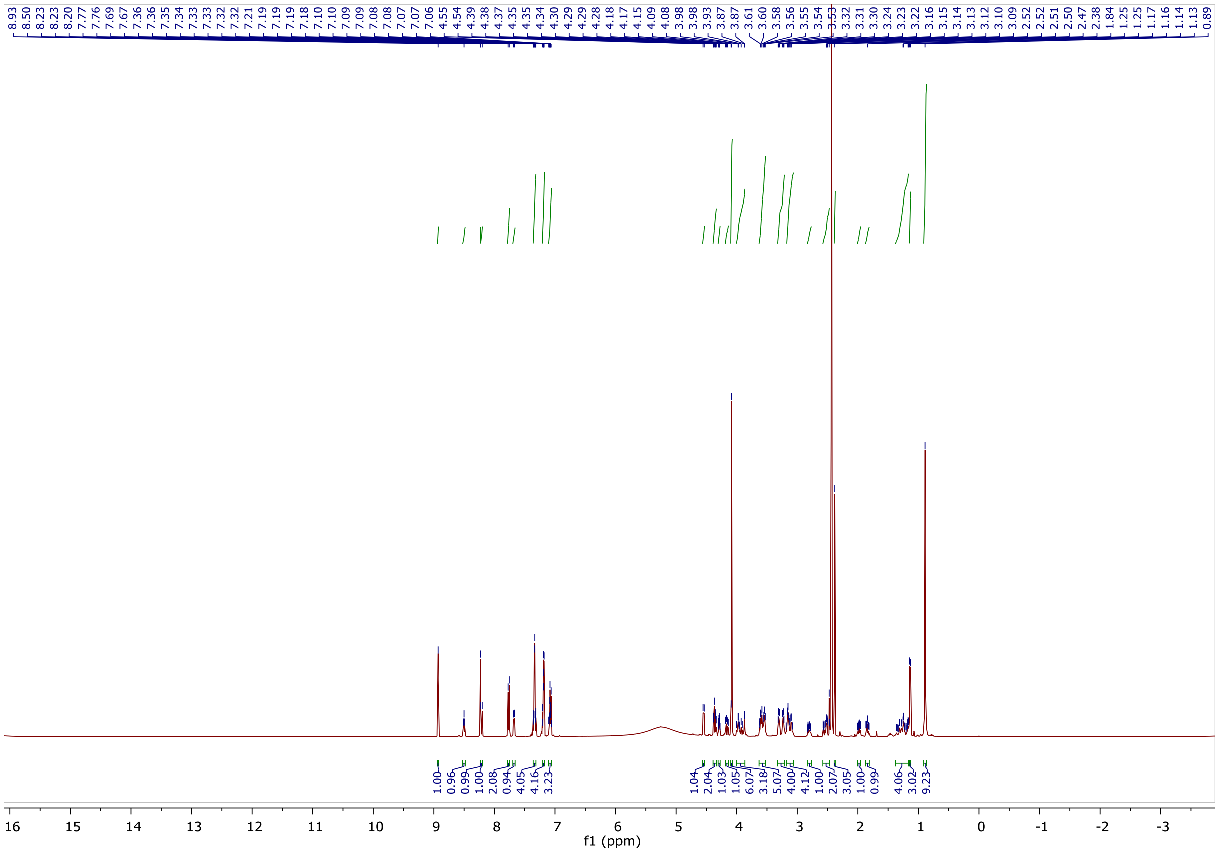

^{13}C NMR spectra of NK232.

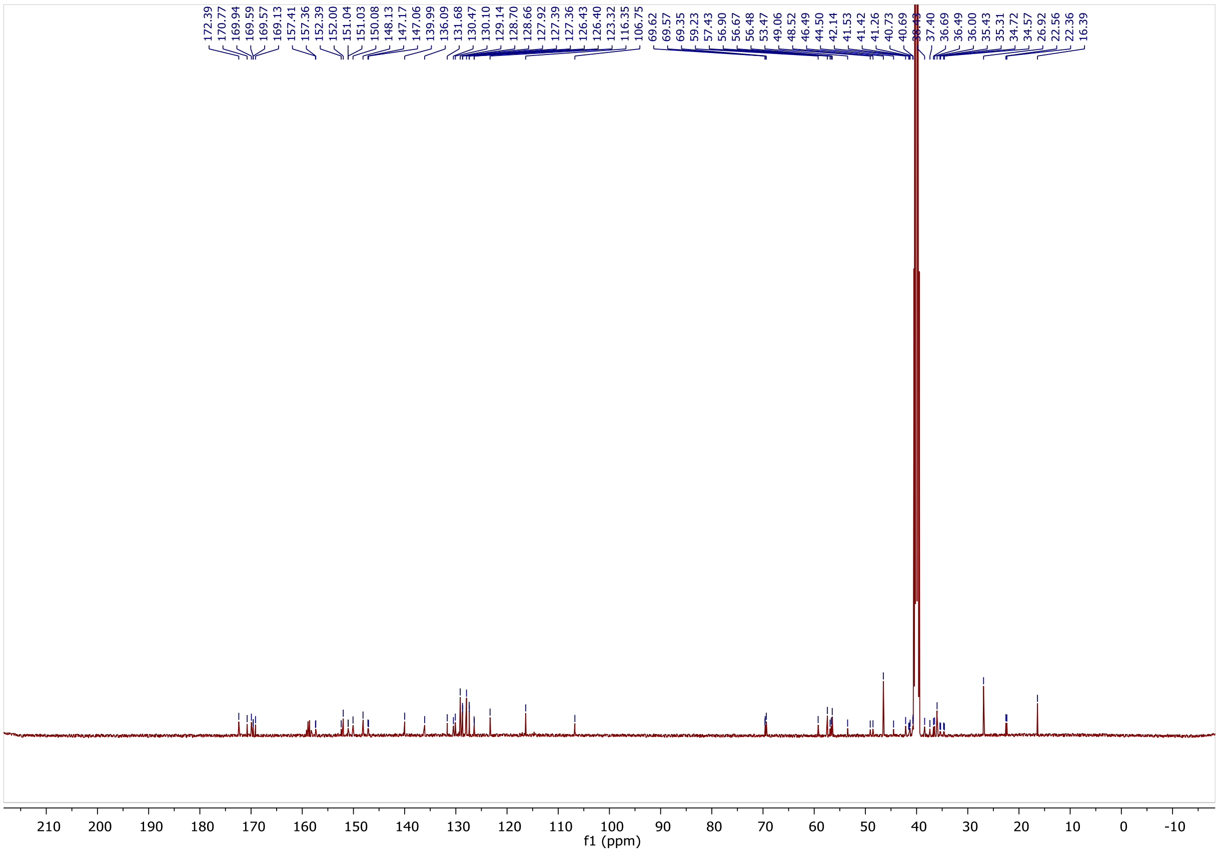

^1H NMR spectra of NK233.

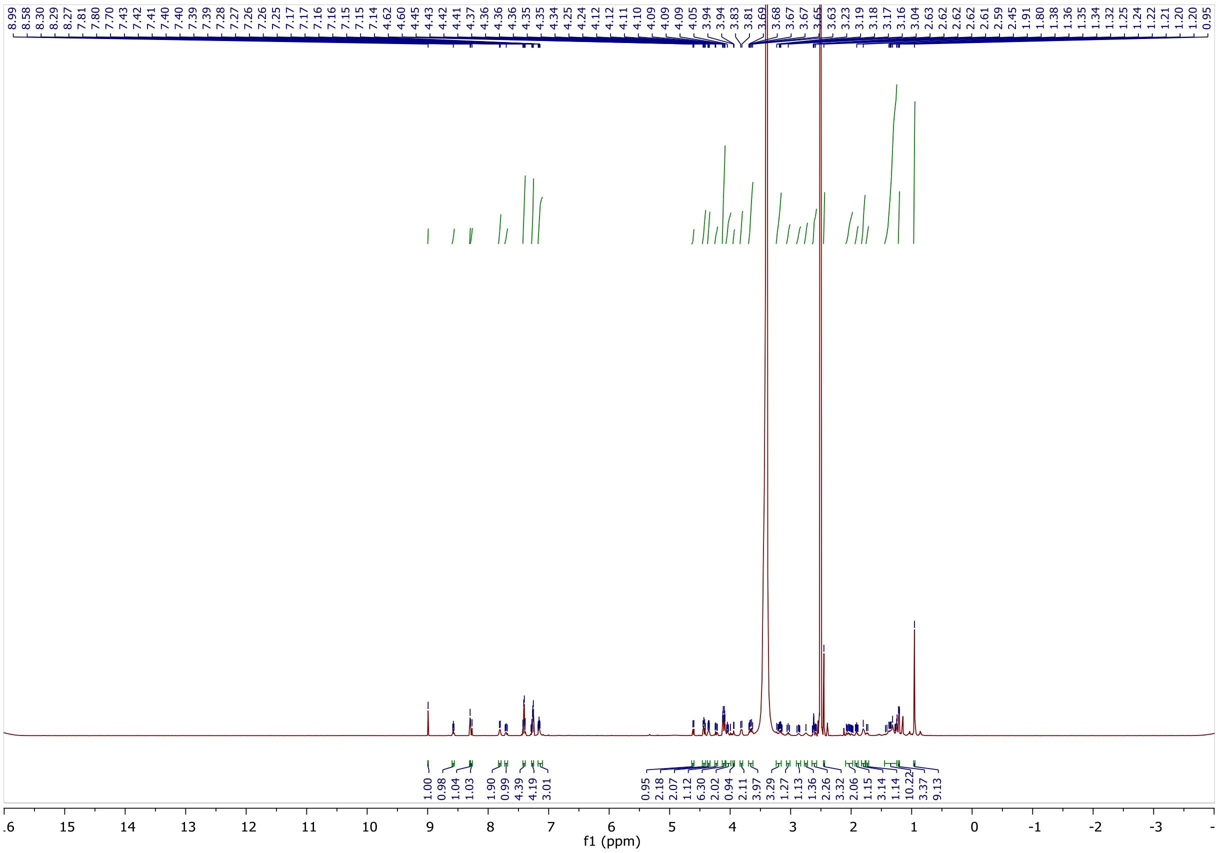

^{13}C NMR spectra of NK233.

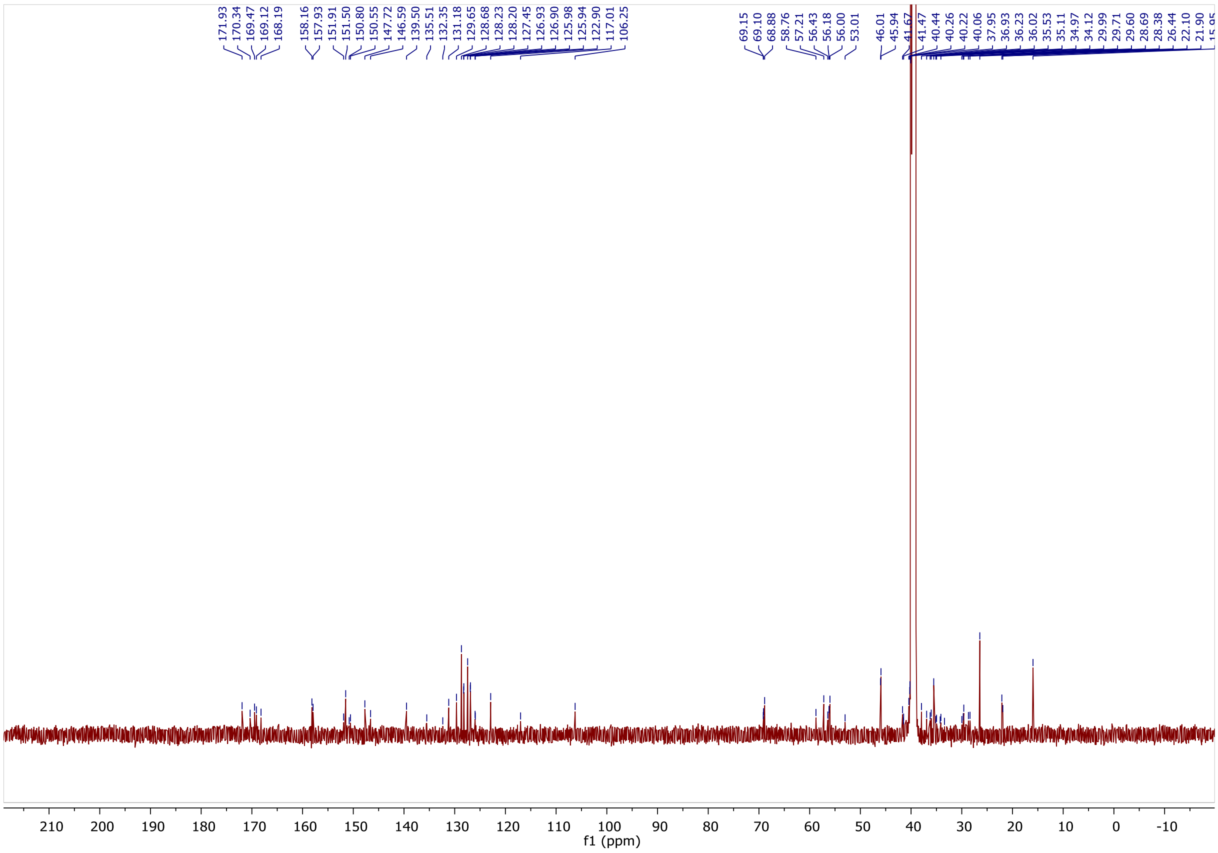

^{1}H NMR spectra of NK234.

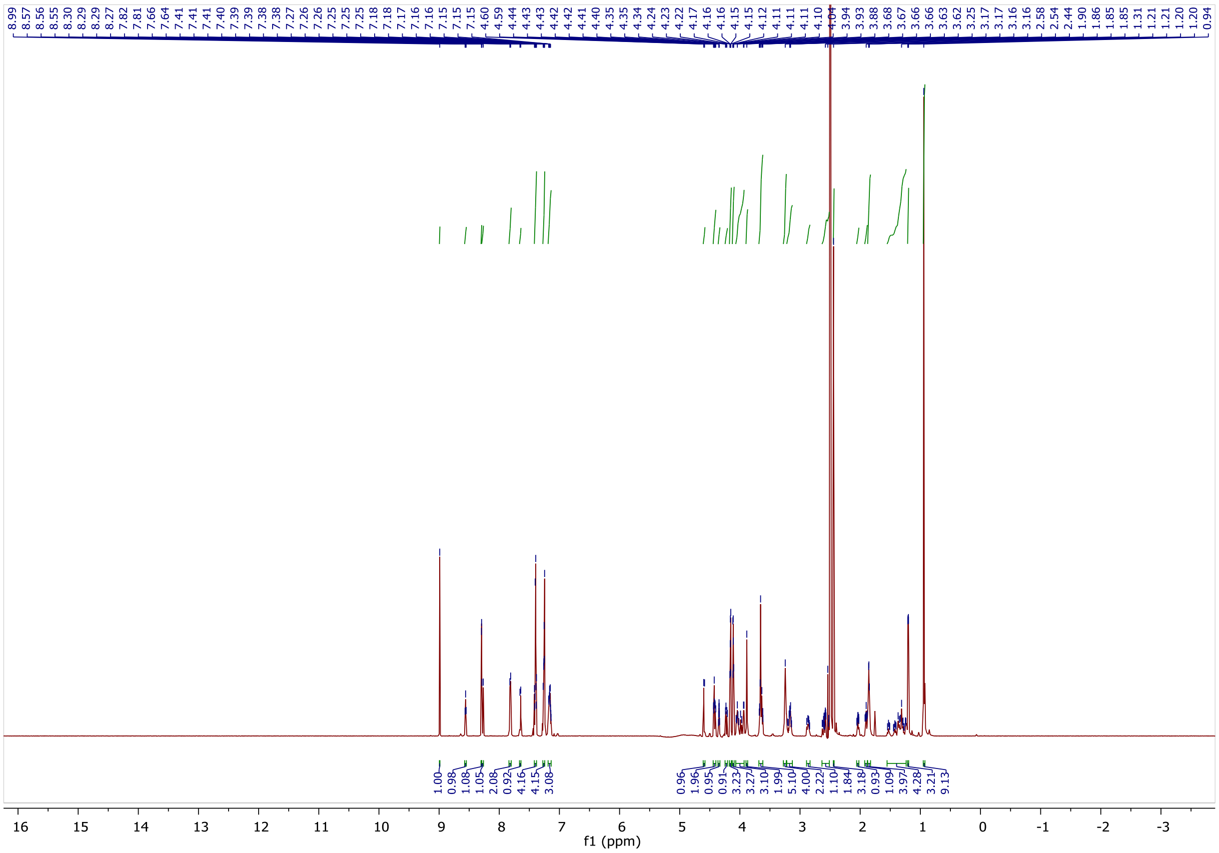

^{13}C NMR spectra of NK234.

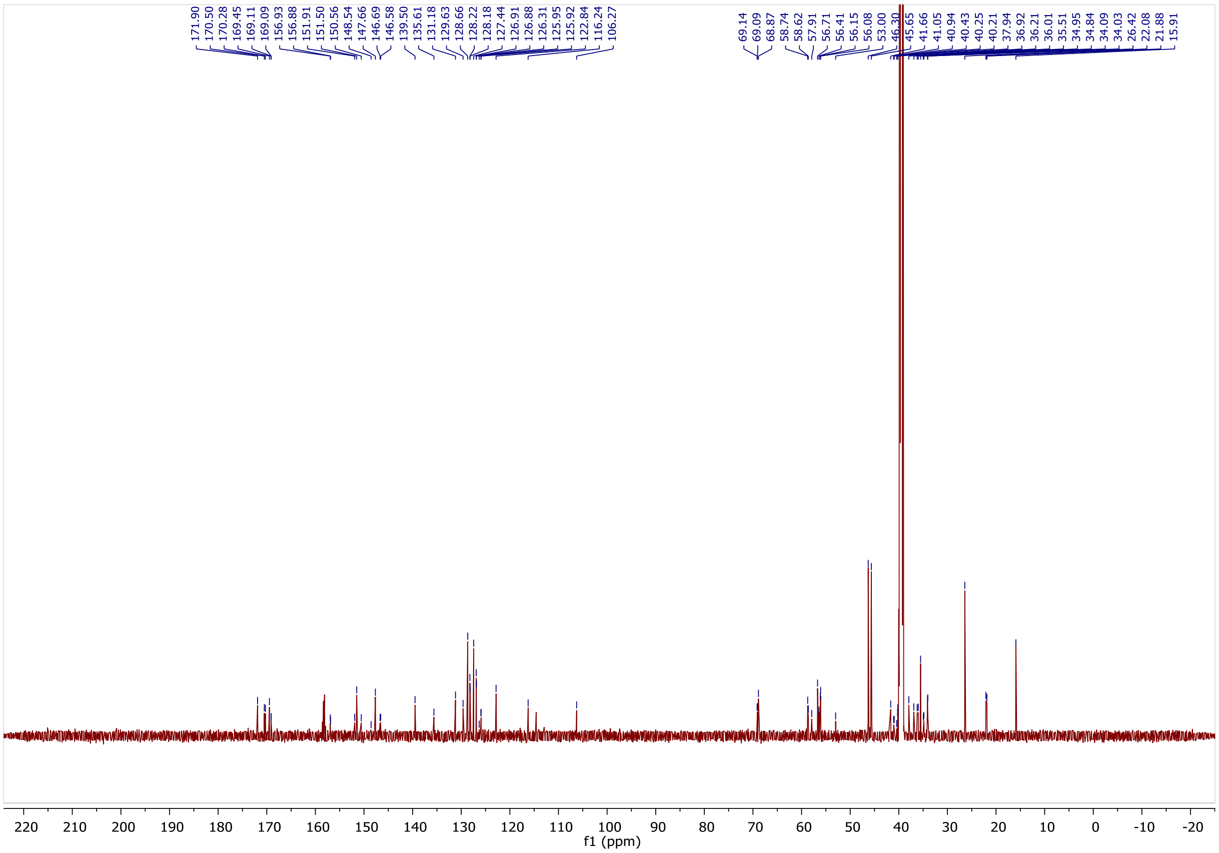

^1H NMR spectra of NK250.

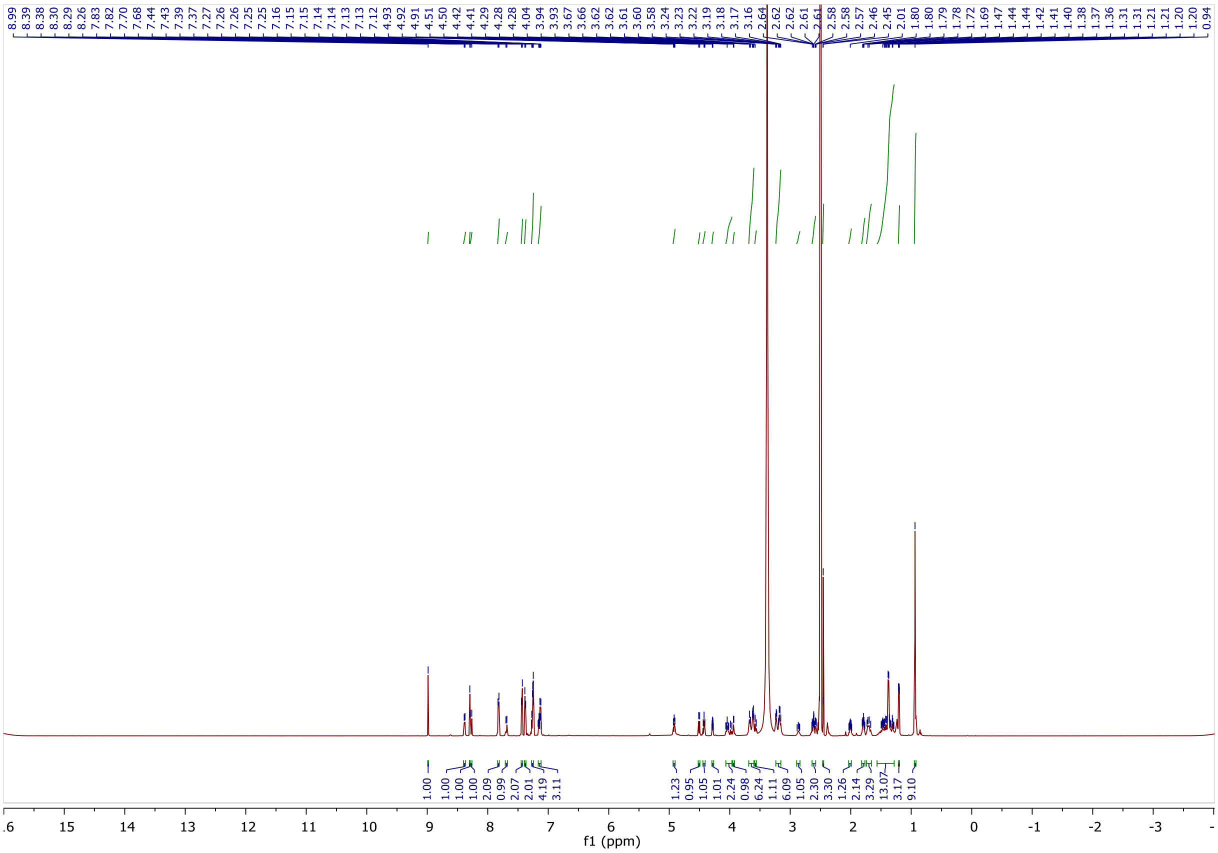

^{13}C NMR spectra of NK250.

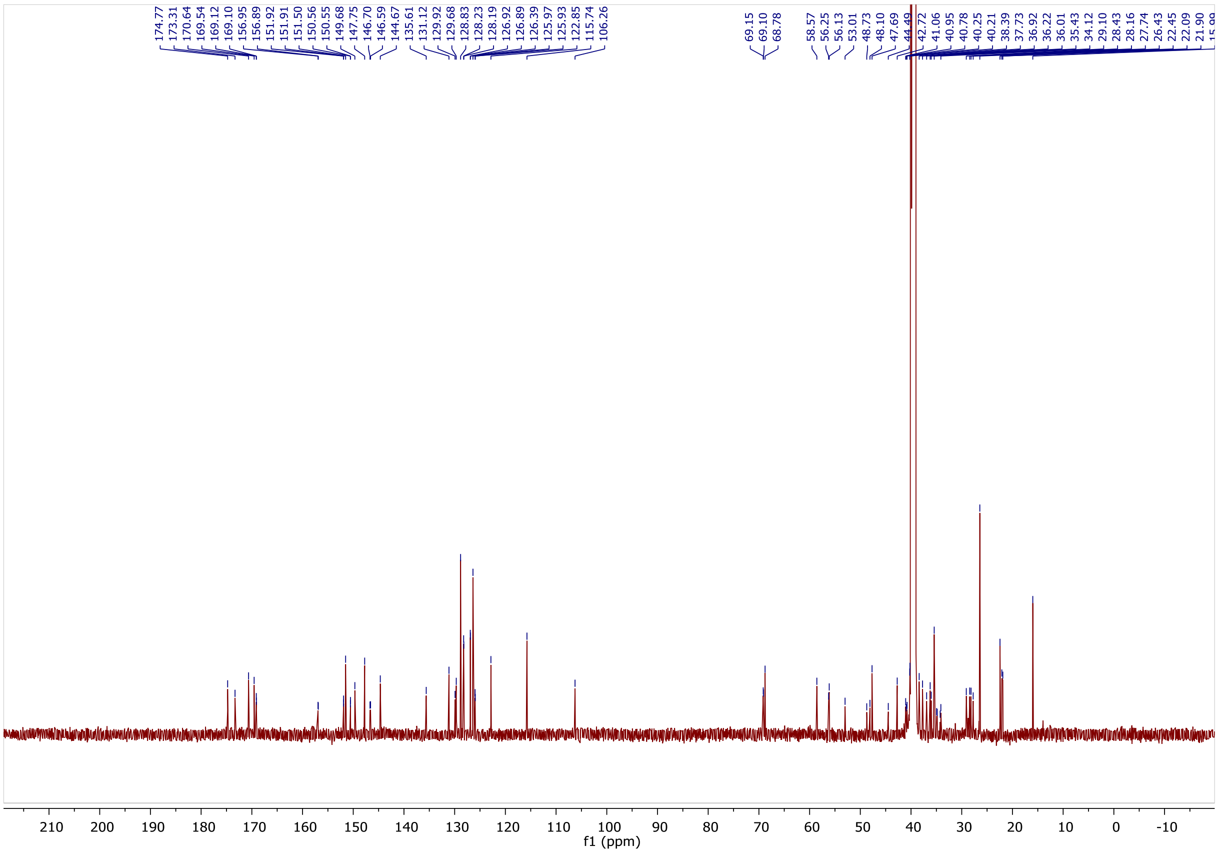

^{1}H NMR spectra of NK266.

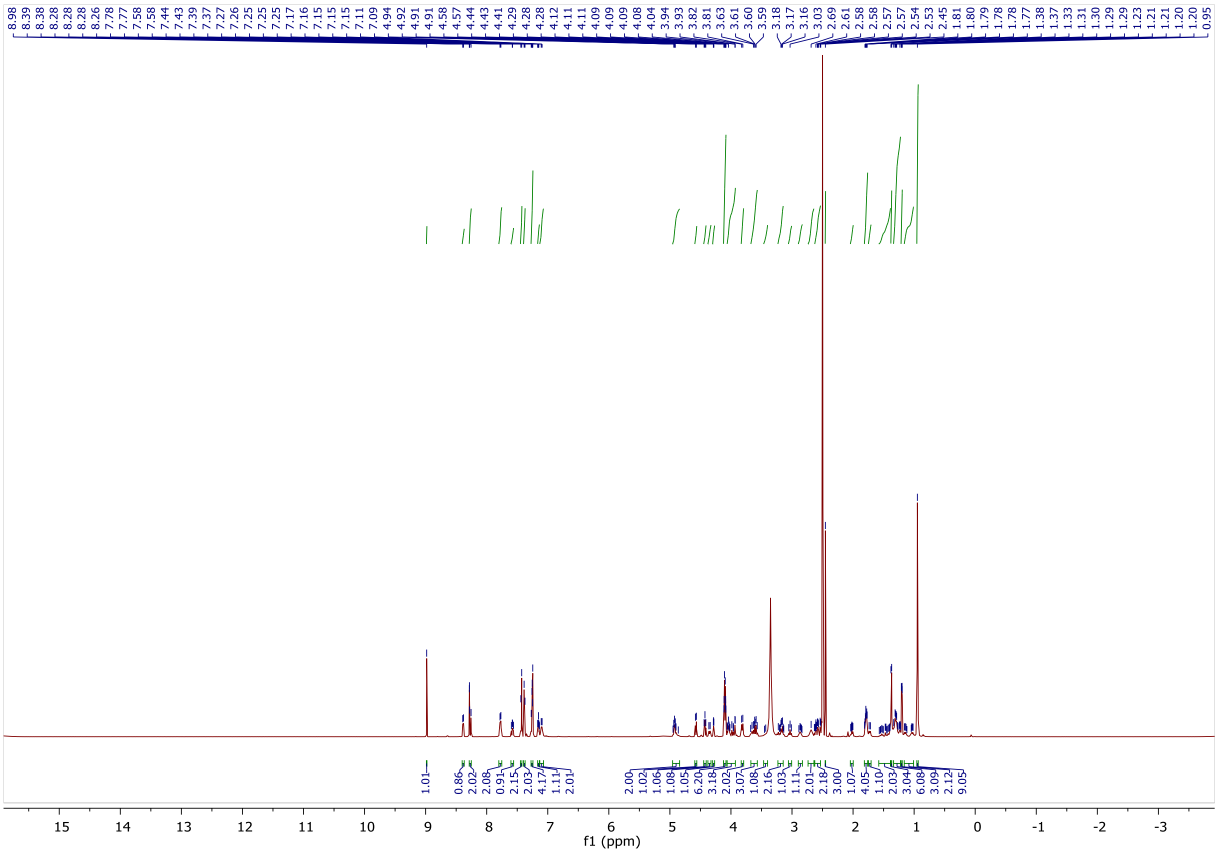

^{13}C NMR spectra of NK266.

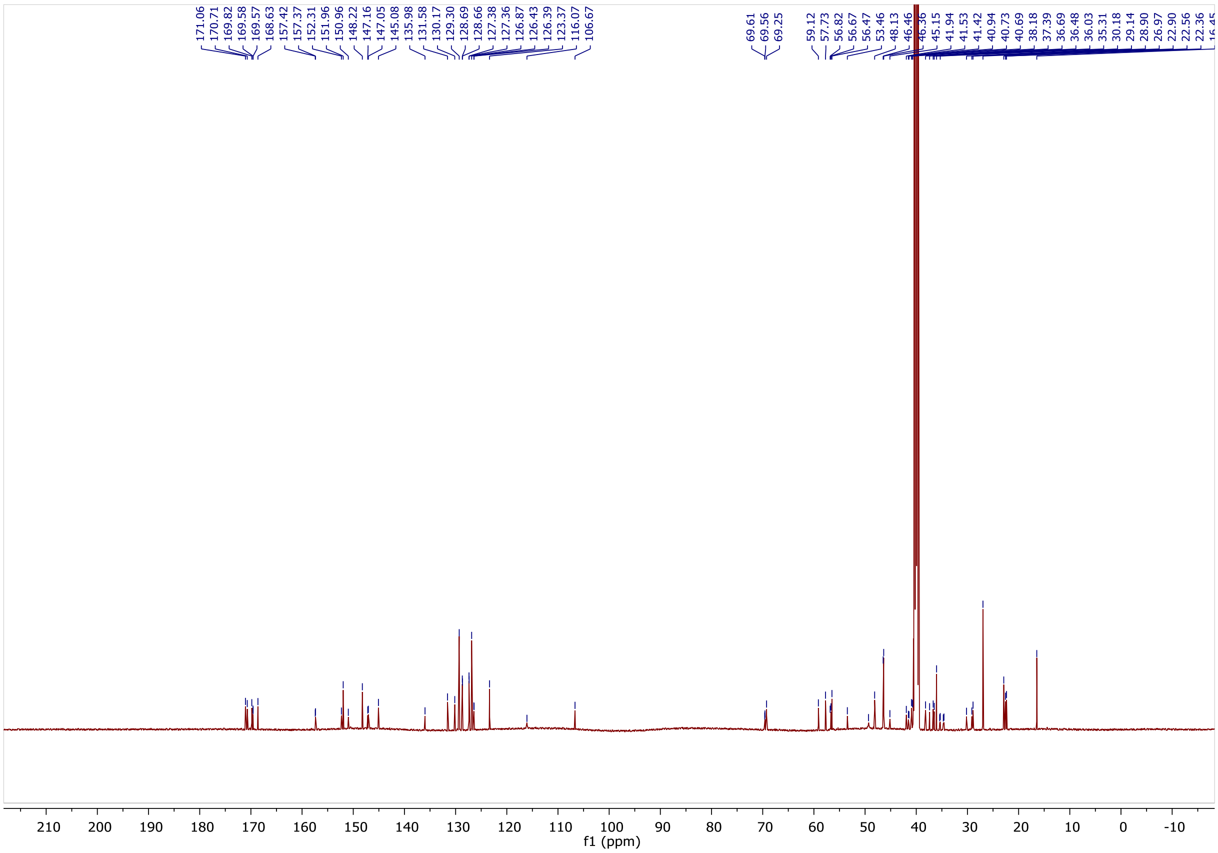

^{1}H NMR spectra of NK249.

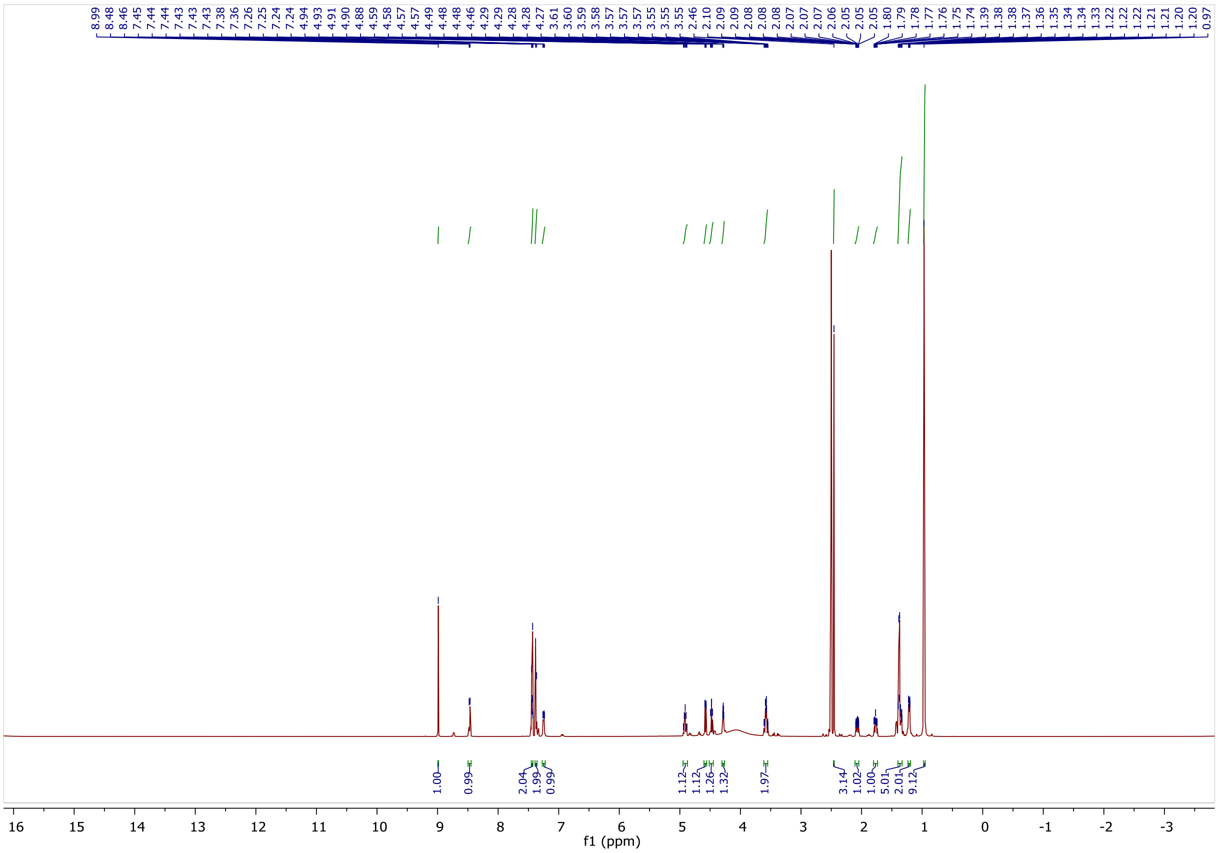

^{13}C NMR spectra of NK249.

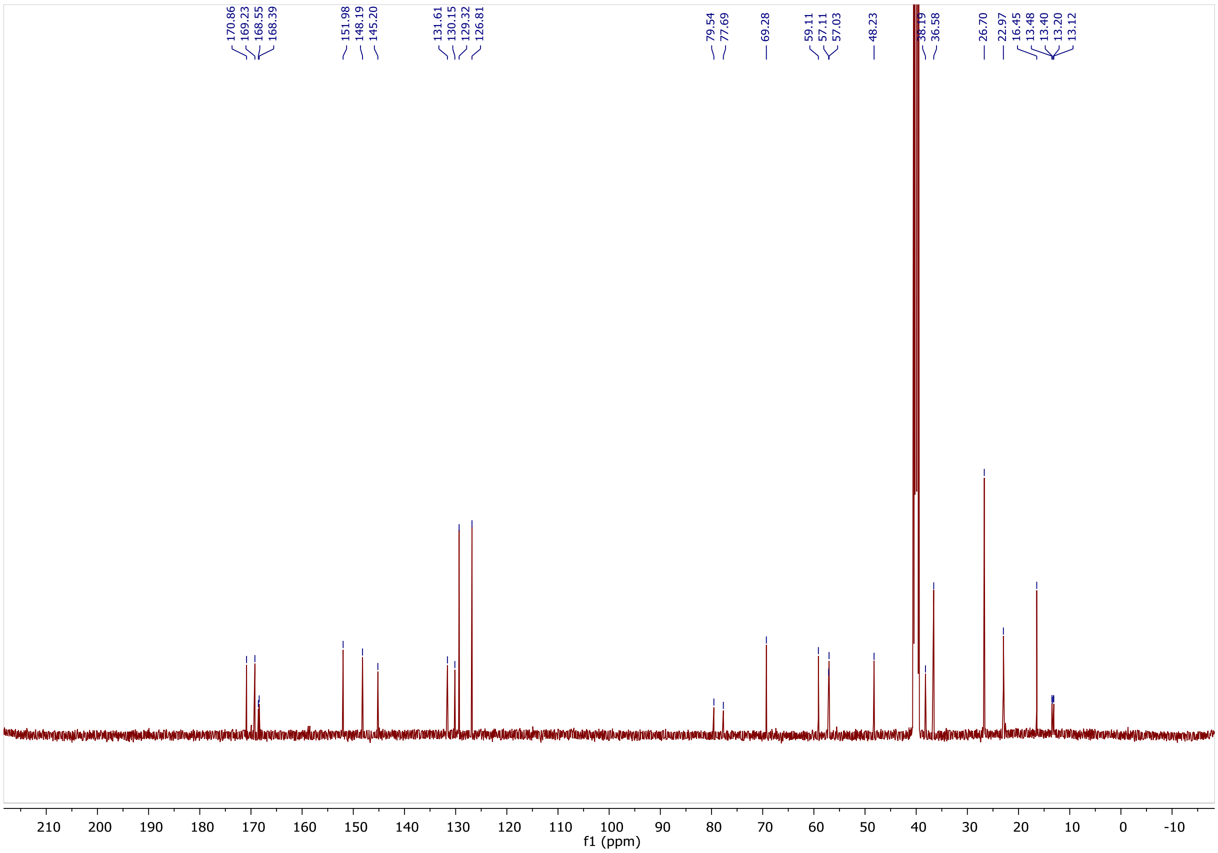

5. Supplementary References

1. Gavory, G., O'Dowd, C.R., Helm, M.D., Flasz, J., Arkoudis, E., Dossang, A., Hughes, C., Cassidy, E., McClelland, K., Odrzywol, E., Page, N., Barker, O., Miel, H. & Harrison, T. Discovery and characterization of highly potent and selective allosteric USP7 inhibitors. *Nat Chem Biol* **14**, 118-125 (2018).

2. Turnbull, A.P. et al. Molecular basis of USP7 inhibition by selective small-molecule inhibitors. *Nature* **550**, 481-486 (2017).

3. Murgai, A., Sosic, I., Gobec, M., Lemnitzer, P., Proj, M., Wittenburg, S., Voget, R., Gutschow, M., Kronke, J. & Steinebach, C. Targeting the deubiquitinase USP7 for degradation with PROTACs. *Chem Commun (Camb)* **58**, 8858-8861 (2022).
