# Supplementary material for: Targeted degradation of USP7 in solid cancer cells reveals distinct effects of deubiquitinase degraders and inhibitors
Source: Nat Commun. 2026 May 13;17:4331. doi: 10.1038/s41467-026-72295-x (PMC13172357; doi:10.1038/s41467-026-72295-x)
Supplement: Supplementary file 8 — Reporting Summary [file 41467_2026_72295_MOESM8_ESM.pdf]

nature portfolio

Corresponding author(s): Annette Paschen, Barbara M. Grüner, Malte Gersch

Last updated by author(s): Mar 31, 2026

Reporting Summary

Nature Portfolio wishes to improve the reproducibility of the work that we publish. This form provides structure for consistency and transparency in reporting. For further information on Nature Portfolio policies, see our Editorial Policies and the Editorial Policy Checklist.

Statistics

For all statistical analyses, confirm that the following items are present in the figure legend, table legend, main text, or Methods section.

n/a	Confirmed	
☐	☒	The exact sample size (n) for each experimental group/condition, given as a discrete number and unit of measurement
☐	☒	A statement on whether measurements were taken from distinct samples or whether the same sample was measured repeatedly
☐	☒	The statistical test(s) used AND whether they are one- or two-sided<br>*Only common tests should be described solely by name; describe more complex techniques in the Methods section.*
☒	☐	A description of all covariates tested
☒	☐	A description of any assumptions or corrections, such as tests of normality and adjustment for multiple comparisons
☐	☒	A full description of the statistical parameters including central tendency (e.g. means) or other basic estimates (e.g. regression coefficient) AND variation (e.g. standard deviation) or associated estimates of uncertainty (e.g. confidence intervals)
☐	☒	For null hypothesis testing, the test statistic (e.g. F, t, r) with confidence intervals, effect sizes, degrees of freedom and P value noted<br>*Give P values as exact values whenever suitable.*
☒	☐	For Bayesian analysis, information on the choice of priors and Markov chain Monte Carlo settings
☒	☐	For hierarchical and complex designs, identification of the appropriate level for tests and full reporting of outcomes
☒	☐	Estimates of effect sizes (e.g. Cohen's d, Pearson's r), indicating how they were calculated

Our web collection on statistics for biologists contains articles on many of the points above.

Software and code

Policy information about availability of computer code

Data collection	Agilent OpenLab CDS (version 2.4); Synentec NyOne YT (version 1.5.8267); Tecan SparkControl (version 2.3); Thermo Scientific Orbitrap Fusion Lumos Tune Application (version 4.1.4244); Thermo Scientific Xcalibur (version 4.7.69.37)
Data analysis	Agilent Wave (version 2.6.1); BD Biosciences FlowJo (version 10); Beckman Coulter Kaluza (version 2.4); Bio-Rad Image Lab (version 6.0); Biovenn.nl; cutadapt (version 1.18); DIA-NN (version 1.8.1 and 2.2.0); edgeR (version 4.0.16); FastQC (version 0.12.1); GenomicAlignments package (version 1.38.2); GraphPad Prism (version 10); GSEA (version 4.3.3); ImageJ (version 1.54p); MaxQuant (version 2.5.2.0); MestreNova (version 12.0.0-20080); Microsoft Excel (version 16); Omero (version web 5.28.0); Perseus (version 1.6.10.0); ProteoWizard; R (version 4.3.3); samtools (version 1.6); STAR (version 2.7.10b); UMI-tools (version 1.0.1)

For manuscripts utilizing custom algorithms or software that are central to the research but not yet described in published literature, software must be made available to editors and reviewers. We strongly encourage code deposition in a community repository (e.g. GitHub). See the Nature Portfolio guidelines for submitting code & software for further information.

Data

Policy information about availability of data

All manuscripts must include a data availability statement. This statement should provide the following information, where applicable:

- Accession codes, unique identifiers, or web links for publicly available datasets
- A description of any restrictions on data availability
- For clinical datasets or third party data, please ensure that the statement adheres to our policy

Mass spectrometry raw data generated in this study have been deposited to the ProteomeXchange Consortium through the PRIDE partner repository under accession codes PXD063889, PXD063917, PXD063920, PXD063913 and PXD070431. 3' mRNA-sequencing data generated in this study have been deposited to the Biostudies database under accession number E-MTAB-16302. Processed mass spectrometry data are enclosed as Supplementary Data 1 (for Fig. 1d), Supplementary Data 2 (for Fig. 5a-f), Supplementary Data 3 (for Fig. 5g-l), Supplementary Data 4 (for Supplementary Fig. 4) Supplementary Data 5 (for Supplementary Fig. 5a-e) and Supplementary Data 6 (for Supplementary Fig. 5f-j). Uncropped blots are provided as Source Data. Numerical Source Data are provided with this paper. Chemical compound characterization data are provided in the Supplementary Information. The H. sapiens reference proteome used in this study is available in the Uniprot database under accession code UP000005640_9606_OPPG. The RNA-seq data of the MGA knock-out used in this study is available in the BioStudies database under accession code E-MTAB-6005. The crystal structure of USP7 in complex with FT671 used in this study is available in the pdb database under accession code 5NGE.

Research involving human participants, their data, or biological material

Policy information about studies with human participants or human data. See also policy information about sex, gender (identity/presentation), and sexual orientation and race, ethnicity and racism.

Reporting on sex and gender	Human research participants were not involved in this study.
Reporting on race, ethnicity, or other socially relevant groupings	Human research participants were not involved in this study.
Population characteristics	Human research participants were not involved in this study.
Recruitment	Human research participants were not involved in this study.
Ethics oversight	Human research participants were not involved in this study.

Note that full information on the approval of the study protocol must also be provided in the manuscript.

Field-specific reporting

Please select the one below that is the best fit for your research. If you are not sure, read the appropriate sections before making your selection.

☒ Life sciences ☐ Behavioural & social sciences ☐ Ecological, evolutionary & environmental sciences

For a reference copy of the document with all sections, see nature.com/documents/nr-reporting-summary-flat.pdf

Life sciences study design

All studies must disclose on these points even when the disclosure is negative.

Sample size	For quantitative experiments, a sample size of 3 to 6 was chosen in line with what is the standard in the field of molecular biosciences.
Data exclusions	Proteomics data analysis including data filtering was carried out as described in the methods and as is implemented in the respective software as automatic processes. For Seahorse experiments, cells were seeded in quintuplicates. At least triplicates were used for data analysis. Data points for some measurements were excluded when injections of the different mitochondrial process inhibitors into wells did not work due to technical issues with the device. The standard criterion was no change in baseline OCR levels upon complex V inhibitor for this well, but for all other replicate wells.
Replication	Observations were made in at least two, typically three, independent experiments, which for numerical assays were each typically carried out with technical triplicates of separate samples, all with consistent results. All replication attempts for each assay were successful. Moreover, western blots determining PROTAC degradation efficiency were performed in three different laboratories, further confirming reproducibility.
Randomization	Randomization was not applicable as no experiments involving humans/animals were performed, and no experiment was sensitive to the order of measurement/treatment.
Blinding	Blinding was not carried out as no subjective scoring was performed during any data collection.

Reporting for specific materials, systems and methods

We require information from authors about some types of materials, experimental systems and methods used in many studies. Here, indicate whether each material, system or method listed is relevant to your study. If you are not sure if a list item applies to your research, read the appropriate section before selecting a response.

Materials & experimental systems

n/a	Involved in the study
☐	☒ Antibodies
☐	☒ Eukaryotic cell lines
☒	☐ Palaeontology and archaeology
☒	☐ Animals and other organisms
☒	☐ Clinical data
☒	☐ Dual use research of concern
☒	☐ Plants

Methods

n/a	Involved in the study
☒	☐ ChIP-seq
☐	☒ Flow cytometry
☒	☐ MRI-based neuroimaging

Antibodies

Antibodies used

Rabbit monoclonal anti-USP7 Clone D17C6 (Cell Signaling Technology, Cat# 4833; RRID: AB_10557113)
Rabbit polyclonal anti-USP7 (Abcam, Cat# ab190183; RRID: AB_2752248)
Rabbit monoclonal anti-GAPDH Clone 14C10 (Cell Signaling Technology, Cat# 2118; RRID: AB_561053)
Mouse monoclonal anti-GAPDH Clone 6C5 (Thermo Fisher Scientific, Cat# AM4300; RRID: AB_2536381)
Mouse monoclonal anti-PCGF6 Clone A-6 (Santa Cruz Biotechnology, Cat# sc-518220; RRID: n/a)
Rabbit polyclonal anti-TRIP12 (Proteintech, Cat# 25303-1-AP; RRID: AB_2880020)
Rabbit monoclonal anti-TRIM27 Clone D5S4O (Cell Signaling Technology, Cat# 15099; RRID: AB_2798707)

Donkey anti-rabbit IgG HRP-linked (Sigma-Aldrich, Cat# GENA934; RRID: AB_2722659)
Sheep anti-mouse IgG HRP-linked (Sigma-Aldrich, Cat# NXA931; RRID: AB_772209)
Goat anti-rabbit IgG Alexa Fluor Plus 488-linked (Thermo Fisher Scientific, Cat# A32731; RRID: AB_2633280)
Goat anti-rabbit IgG HRP-linked (Cell Signaling Technology, Cat# 7074; RRID: AB_2099233)
Goat anti-mouse IgG HRP-linked (Jackson ImmunoResearch Labs, Cat# 115-035-003; RRID: AB_10015289)

Validation

Primary antibodies were validated for their respective applications as indicated on the manufacturers' websites:

Rabbit monoclonal anti-USP7:
https://www.cellsignal.com/products/primary-antibodies/hausp-d17c6-xp-rabbit-mab/4833
"Application: Western Blotting"

Rabbit polyclonal anti-USP7:
https://www.abcam.com/en-us/products/primary-antibodies/hausp-usp7-antibody-ab190183
"Application: Western blot on human samples: tested (two check marks); IF on human samples: tested (two check marks)"

Rabbit monoclonal anti-GAPDH:
https://www.cellsignal.com/products/primary-antibodies/gapdh-14c10-rabbit-mab/2118
"Application: Western Blotting"

Mouse monoclonal anti-GAPDH:
https://www.thermofisher.com/antibody/product/GAPDH-Antibody-clone-6C5-Monoclonal/AM4300
"Applications: Western Blot (WB)"

Rabbit polyclonal anti-TRIP12:
https://www.ptglab.com/products/TRIP12-Antibody-25303-1-AP.htm
"Application: WB"

Mouse monoclonal anti-TRIM27:
https://www.cellsignal.com/products/primary-antibodies/trim27-d5s4o-rabbit-monoclonal-antibody/15099
"Application: Western Blotting"

Eukaryotic cell lines

Policy information about cell lines and Sex and Gender in Research

Cell line source(s)

Ma-Mel-47; established previously from metastatic lesions from patient tumor tissues after written informed consent
Panc89; RRID: CVCL_4056, also referenced as T3M-455
MV4-11; RRID: CVCL_0064 (Eilers Lab)
HEK293; RRID: CVCL_0045 (ATCC)
HEK293T; RRID: CVCL_0063 (ATCC)

Authentication	Ma-Mel-47 and Panc89 were authenticated by STR analysis performed by the Microsynth Seqlab GmbH. All other cell lines were used without authentication.
Mycoplasma contamination	All cells were tested for mycoplasma contamination with a negative result.
Commonly misidentified lines (See ICLAC register)	No commonly misidentified lines were used in this study.

Plants

Seed stocks	No plants were used in this study.
Novel plant genotypes	No plants were used in this study.
Authentication	No plants were used in this study.

Flow Cytometry

Plots

Confirm that:

- [x] The axis labels state the marker and fluorochrome used (e.g. CD4-FITC).
- [x] The axis scales are clearly visible. Include numbers along axes only for bottom left plot of group (a 'group' is an analysis of identical markers).
- [x] All plots are contour plots with outliers or pseudocolor plots.
- [x] A numerical value for number of cells or percentage (with statistics) is provided.

Methodology

Sample preparation	Eukaryotic cell lines Panc89 and Ma-Mel-47 were harvested for FACS anaylsis in FACS tubes, centrifuged and stained with FITC Annexin V (1:30; BD Biosciences 556419) and DAPI (1:1000) in custom made binding buffer (10x stock: 0.1 M HEPES, 1.4 M NaCl and 25 mM CaCl2, pH: 7.0).<br>Ma-Mel-47 CRISPR/Cas9 USP7 knockout cells were harvested for FACS analysis in FACS tubes, centrifuged and stained in the same buffer containing Annexin V-APC (1:40; Thermo Fisher Scientific A35110) and propidium iodide (PI; BD Pharmingen Propidium Iodide Staining Solution 556463) and incubated at room temperature in the absence of light for 15 min. Afterward, PBS was added, and cells were immediately analysed.
Instrument	Stained Panc89 and Ma-Mel-47 cells were measured on a BD FACSCelesta Cell Analyzer from BD Biosciences.<br>Ma-Mel-47 USP7 knockout cells were measured on a Gallios flow cytometer from Beckman Coulter.
Software	For Panc89 and Ma-Mel-47: FACS data were analyzed using the FlowJo software v10 from BD Biosciences.<br>For Ma-Mel-47 USP7 knockout cells: FACS data were analyzed using the KaluzaAnalysis Software v2.4 from Beckman Coulter.
Cell population abundance	For each sample at least 10000 events were recorded, from which 80-95% were singe cells. Depending on whether cells were cultured in high or low glucose medium the fraction of cells in each of the four quadrants (AnnV+/DAPI-; AnnV-/DAPI+, AnnV-/DAPI- or AnnV+/DAPI+) differed.<br><br>For Panc89 cells:<br><br>High glucose:<br>AnnV+/DAPI-: 5-10%<br>AnnV-/DAPI+: 0.5-1.5%<br>AnnV-/DAPI-: 80-90%<br>AnnV+/DAPI+: 4-7%<br><br>Low glucose:<br>AnnV+/DAPI-: 2-8%<br>AnnV-/DAPI+: 0.1-0.7%<br>AnnV-/DAPI-: 68-95%<br>AnnV+/DAPI+: 2.5-16%<br><br>For Ma-Mel-47 cells:

High glucose:
AnnV+/DAPI-: 2-3%
AnnV-/DAPI+: 0.2-0.5%
AnnV-/DAPI-: 88-97%
AnnV+/DAPI+: 0.7-6%

Low glucose:
AnnV+/DAPI-: 2-47%
AnnV-/DAPI+: 0.1-1.5%
AnnV-/DAPI-: 23-97%
AnnV+/DAPI+: 1-28%

For Ma-Mel-47 USP7 K.O. cells:

For each sample at least 20000 events were recorded, from which between 80-95% were singe cells. Depending on whether cells were cultured in high or low glucose medium the fraction of cells in each of the four quadrants (AnnV+/PI-; AnnV-/PI+, AnnV+/PI- or AnnV+/PI+) differed.

High glucose:
AnnV-/PI-: 29-94%
AnnV-/PI+: 1.7-25%
AnnV+/PI-: 0.15-0.75%
AnnV+/PI+: 1.4-19%

Low glucose:
AnnV-/PI-: 33-95 %
AnnV-/PI+: 2.8-35 %
AnnV+/PI-: 0.1-1.7%
AnnV+/PI+: 1.4-41 %

Gating strategy

1. Gate on single cells to exclude doublets with FSC-H vs. FSC-A
2. Quadrant gating with Pacific blue-A (DAPI) vs. FITC-A (AnnV) or respectively mCherry (PI) vs. APC-A (AnnV)

☒ Tick this box to confirm that a figure exemplifying the gating strategy is provided in the Supplementary Information.
