# Supplementary material for: Targeted degradation of USP7 in solid cancer cells reveals distinct effects of deubiquitinase degraders and inhibitors
Source: Nat Commun. 2026 May 13;17:4331. doi: 10.1038/s41467-026-72295-x (PMC13172357; doi:10.1038/s41467-026-72295-x)

Uncropped blots

Fig. 2c

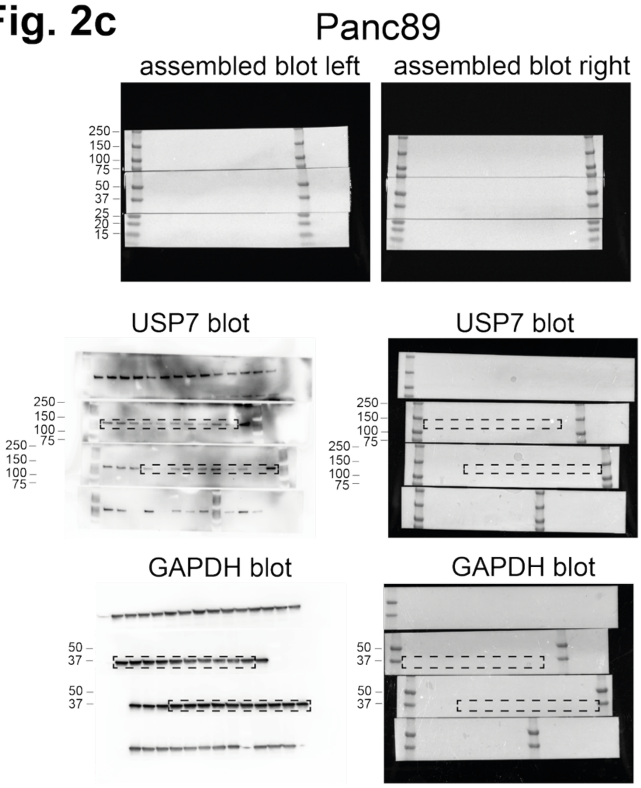

Fig. 2d

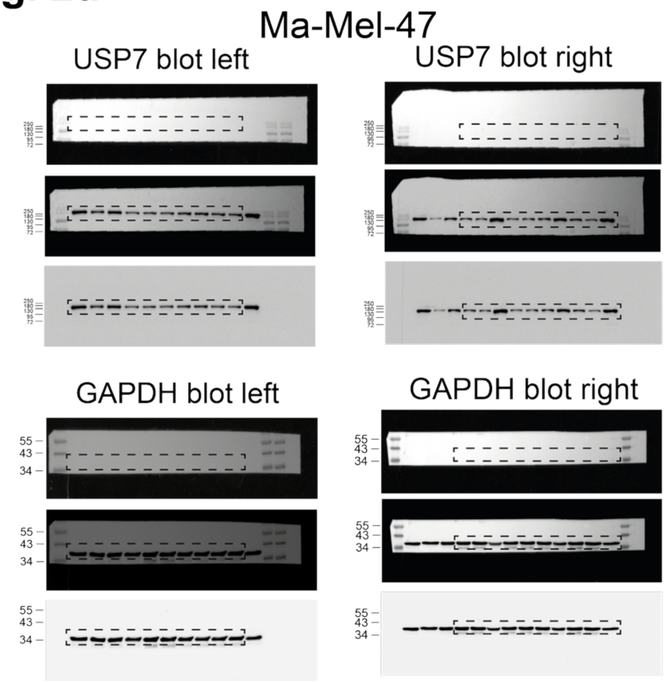

Fig. 3b

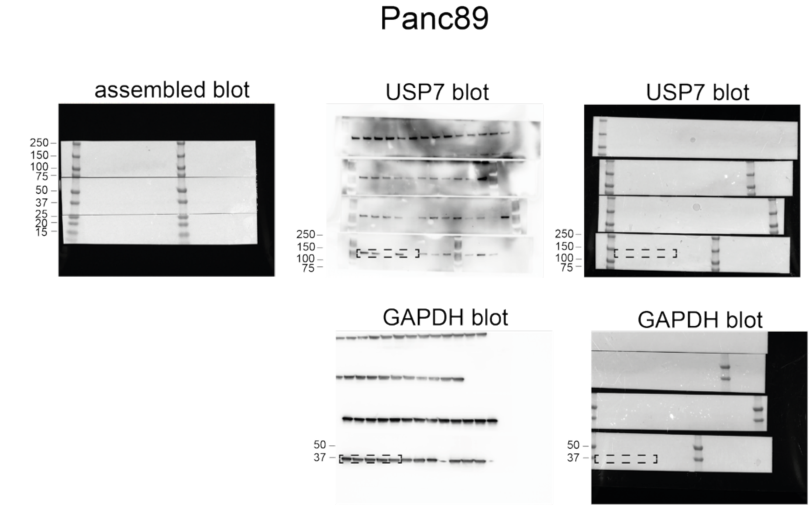

Fig. 3c

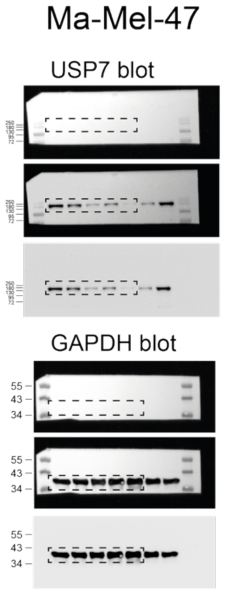

Fig. 3d

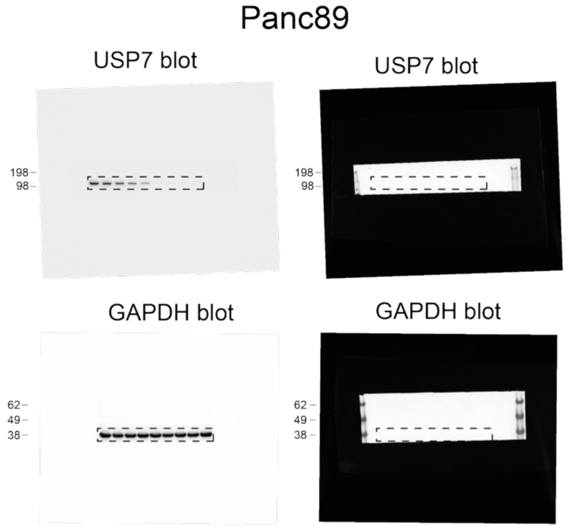

Fig. 3e

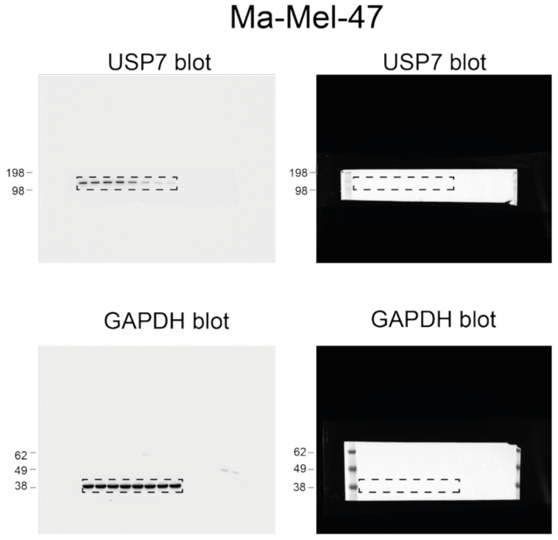

Fig. 3f

Panc89

Blots used for quantification

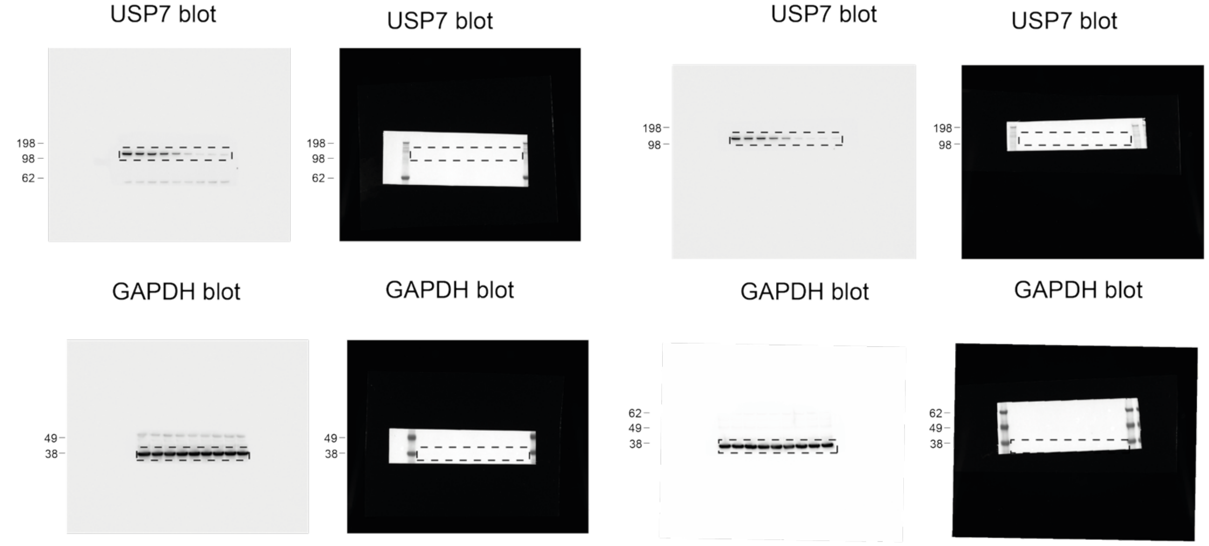

Fig. 3g

Ma-Mel-47

Blots used for quantification

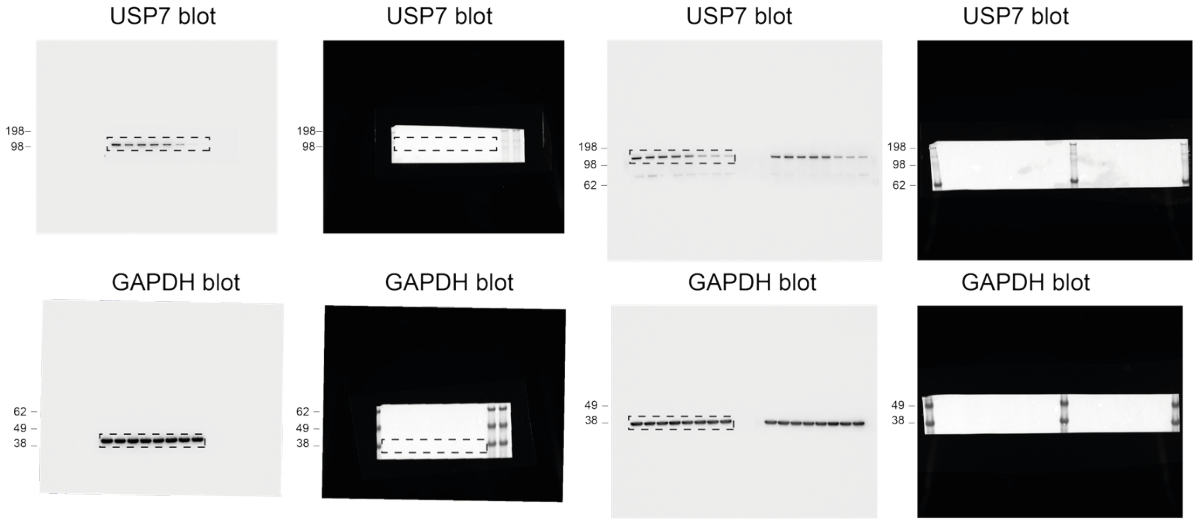

Fig. 3h

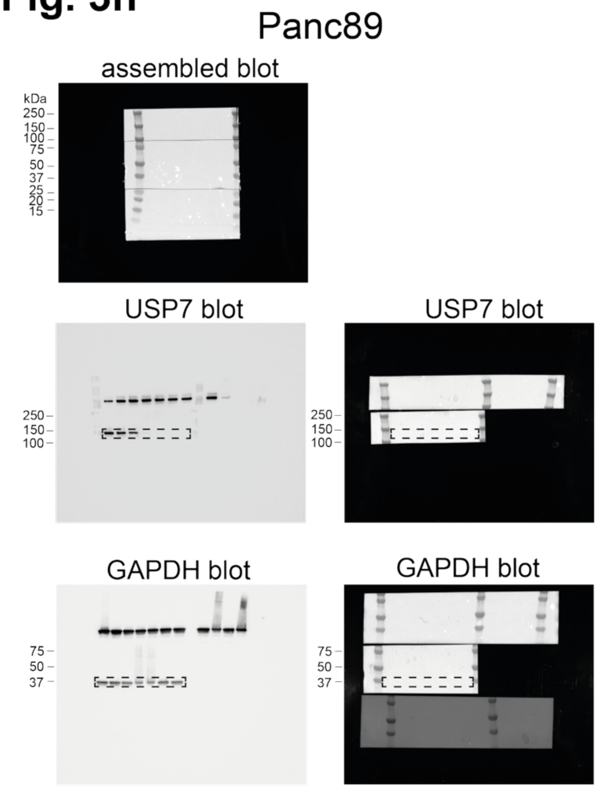
Panc89
assembled blot
kDa
250
150
100
75
50
37
25
20
15
USP7 blot
250
150
100
USP7 blot
250
150
100
GAPDH blot
75
50
37
GAPDH blot
75
50
37

Fig. 3i

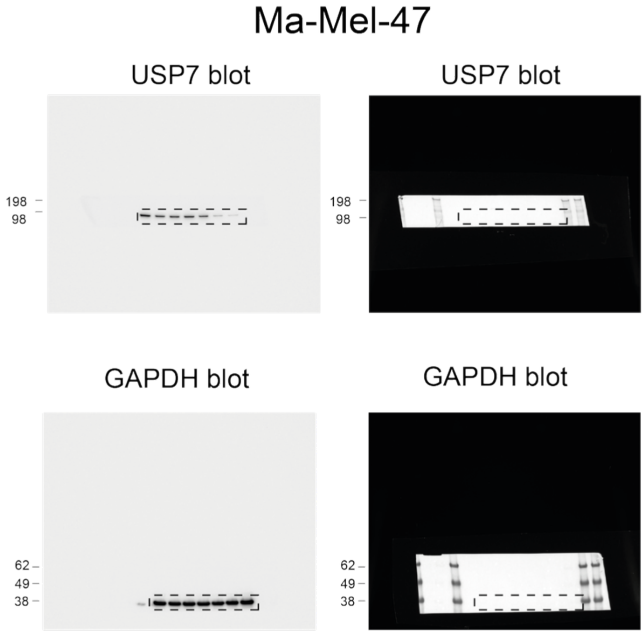
Ma-Mel-47
USP7 blot
198
98
USP7 blot
198
98
GAPDH blot
62
49
38
GAPDH blot
62
49
38

Fig. 3j

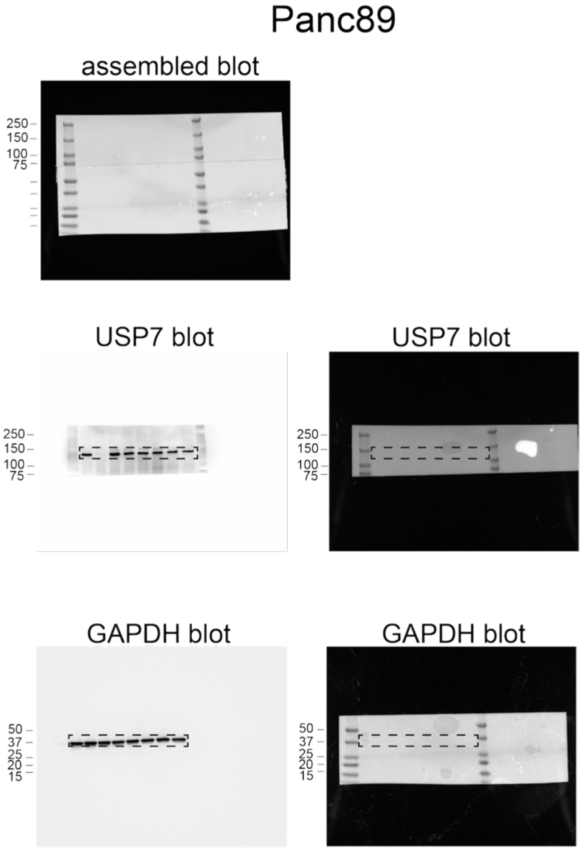

Panc89
assembled blot
250
150
100
75
USP7 blot
250
150
100
75
USP7 blot
250
150
100
75
GAPDH blot
50
37
25
20
15
GAPDH blot
50
37
25
20
15

Fig. 3k

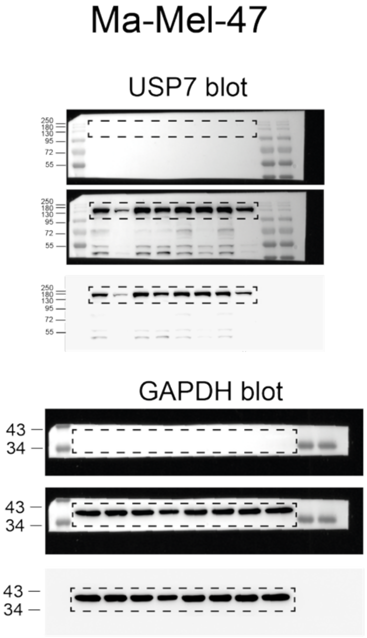

Ma-Mel-47
USP7 blot
250
180
130
95
72
55
GAPDH blot
43
34

Fig. 6a

Panc89

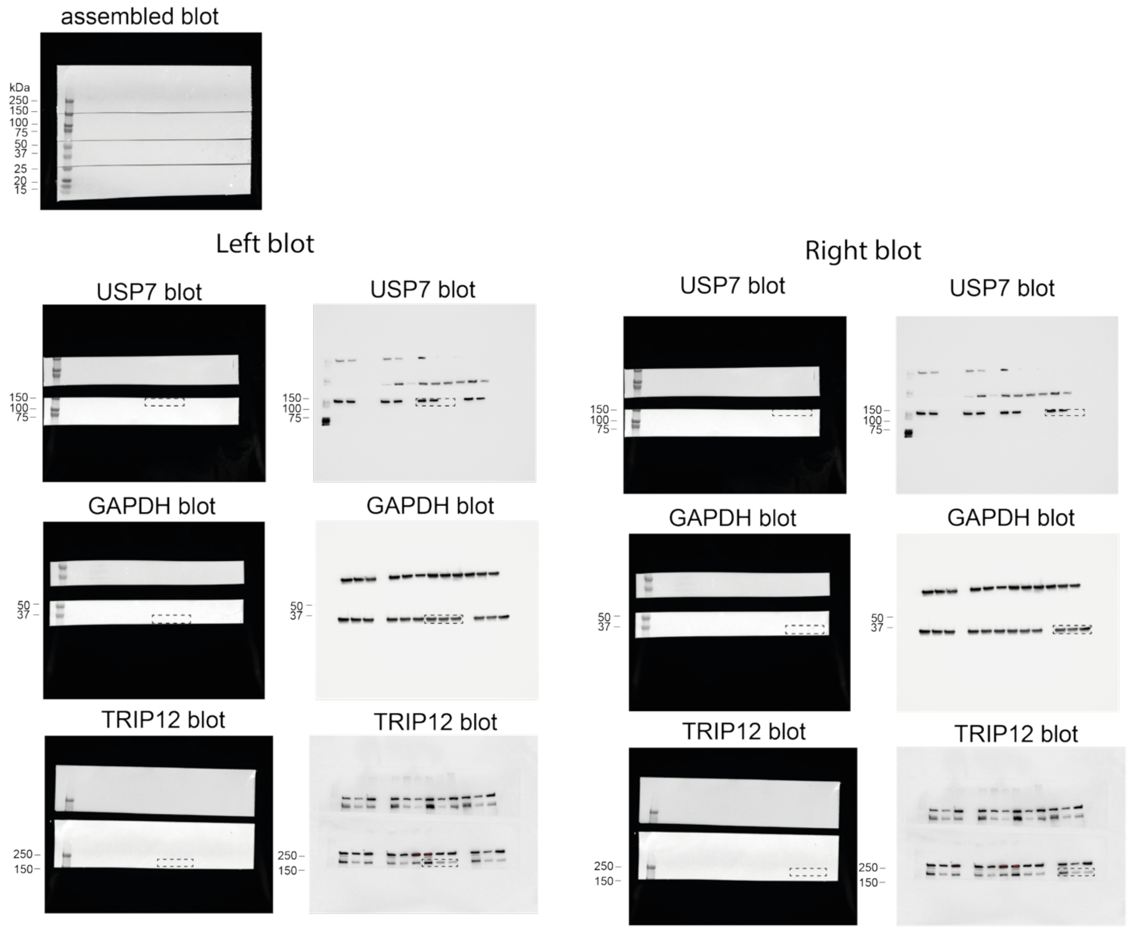

Fig. 6b

Ma-Mel-47

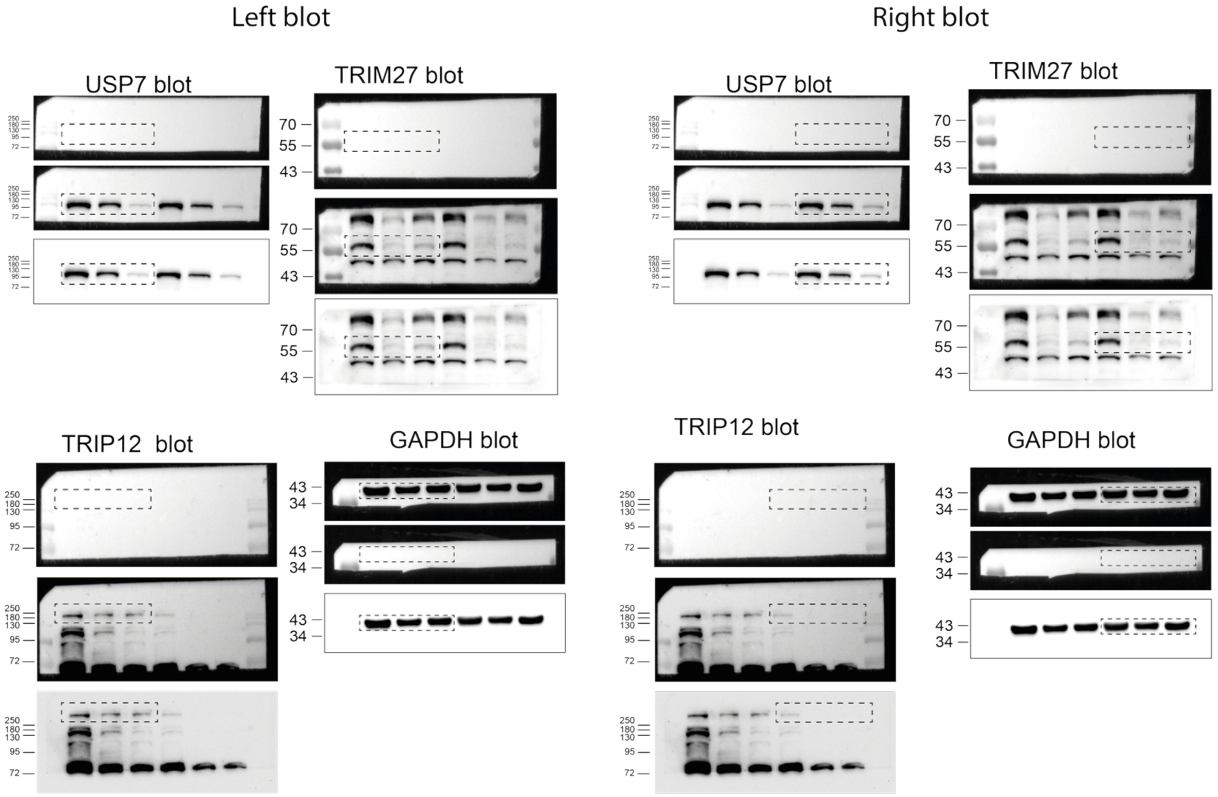

Fig. 6c

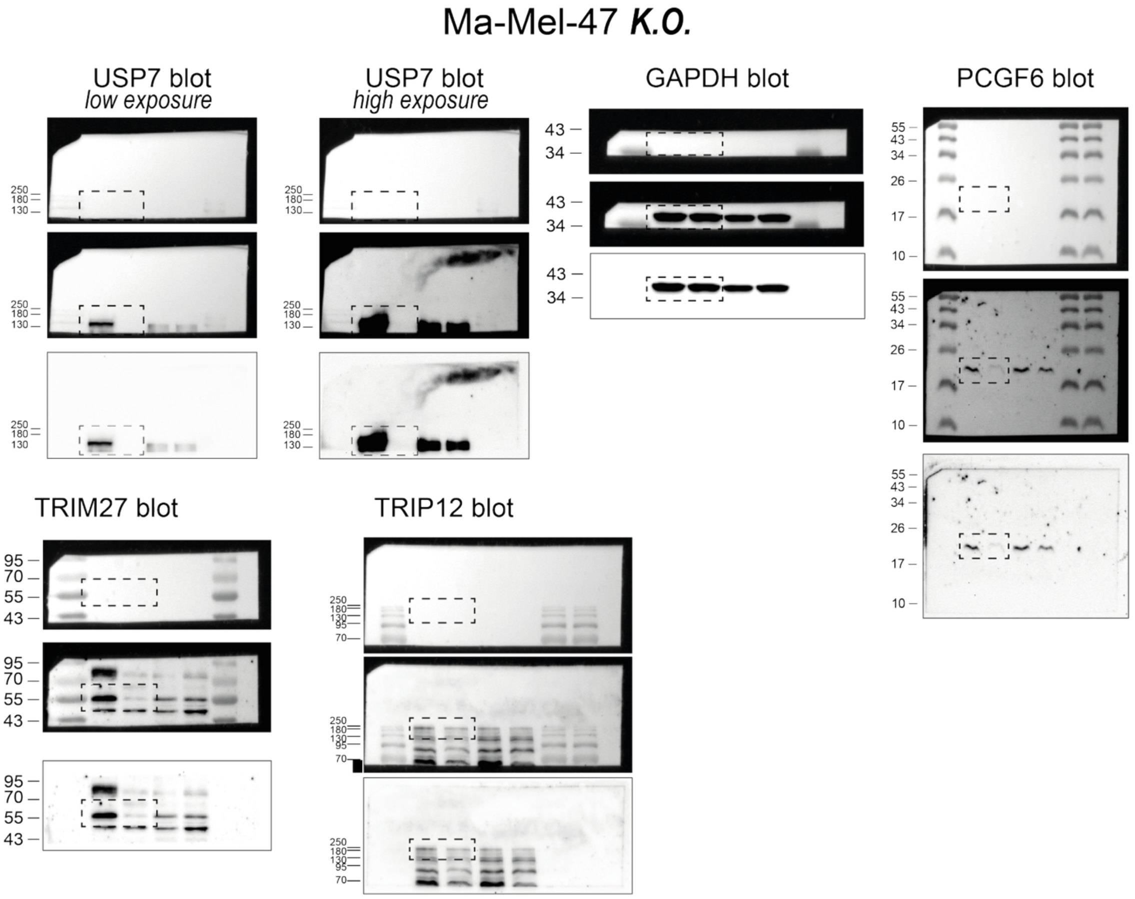

Fig. 7c

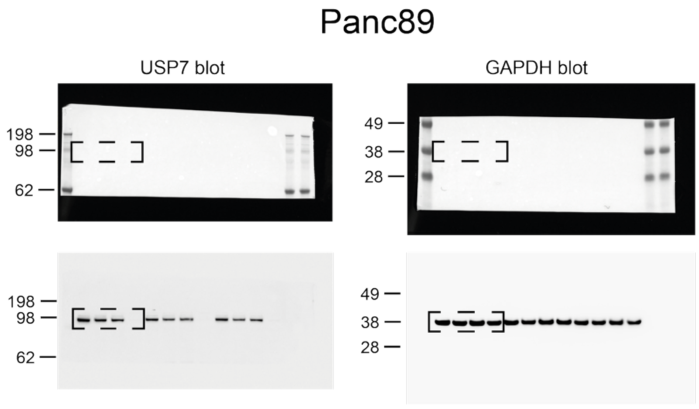

Fig. 7f

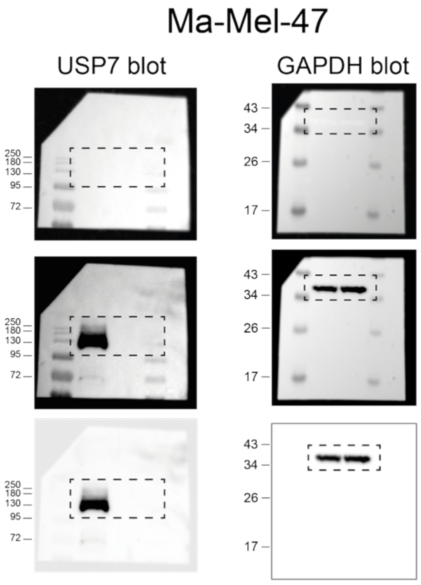

Supplementary Fig. 2a

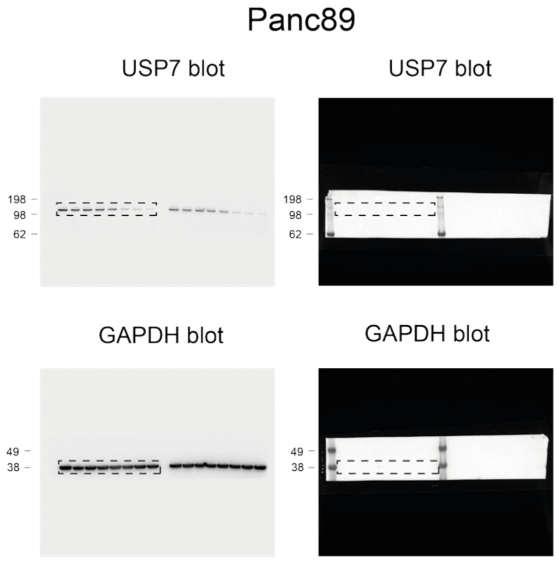

Supplementary Fig. 2b

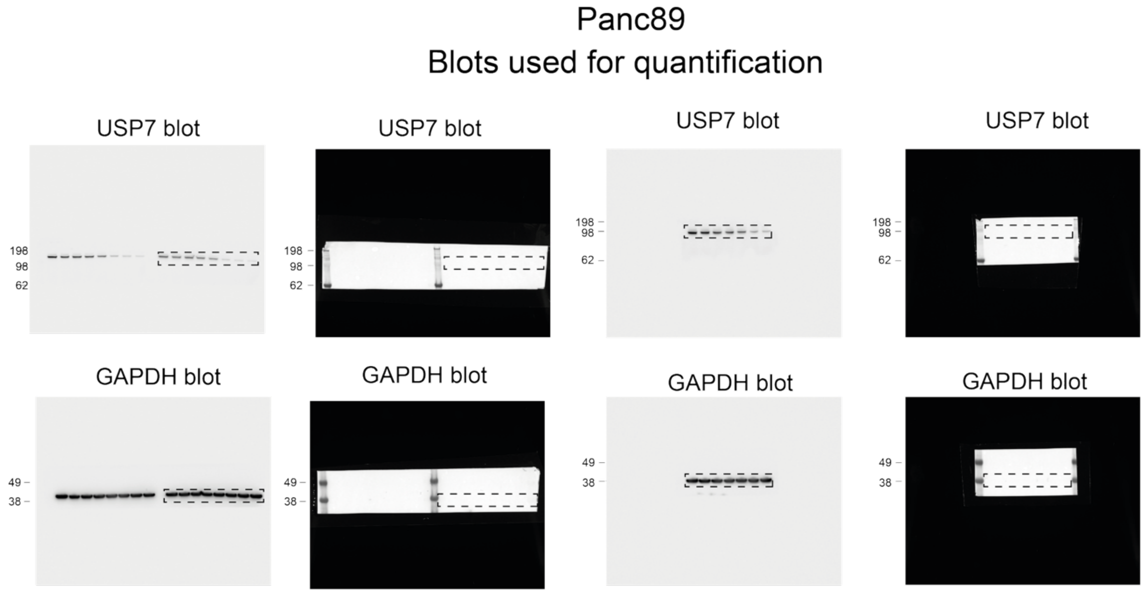

Supplementary Fig. 2c

MaMel47

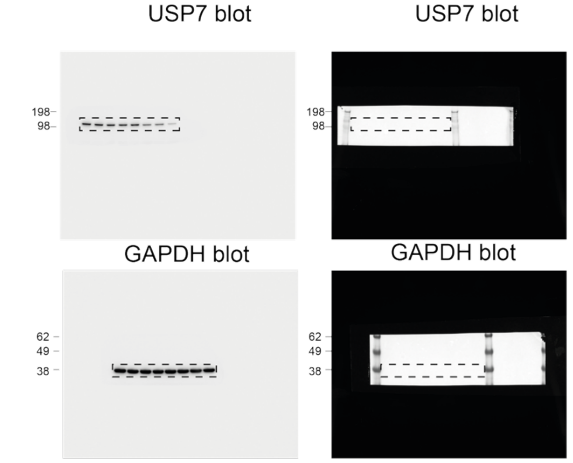

Supplementary Fig. 2e

Panc89

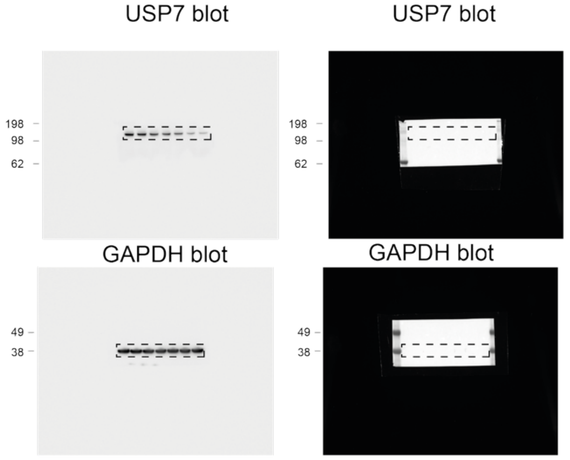

Supplementary Fig. 2d

MaMel47

Blots used for quantification

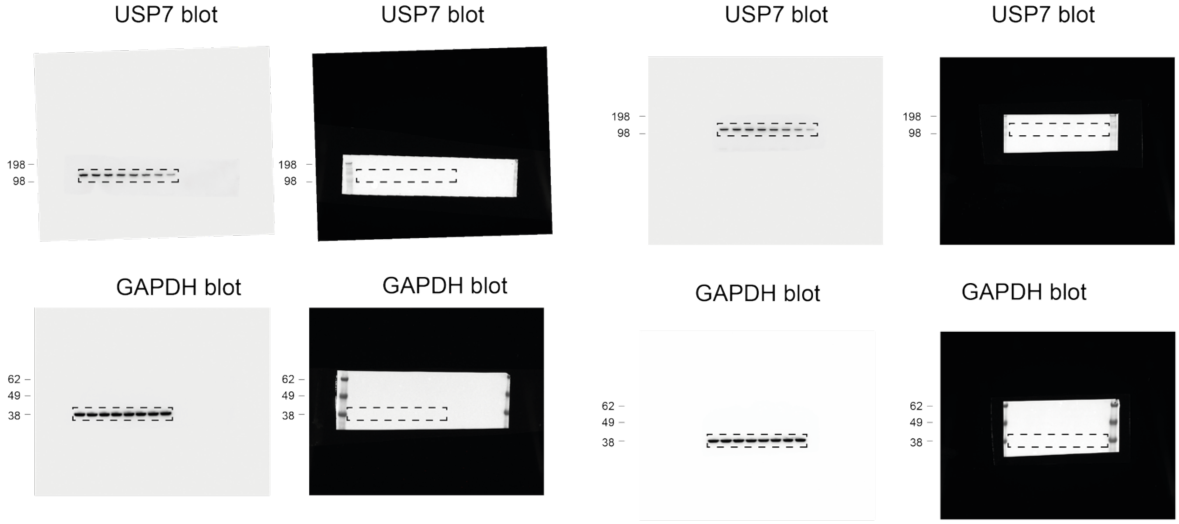

Supplementary Fig. 2f

MaMel47

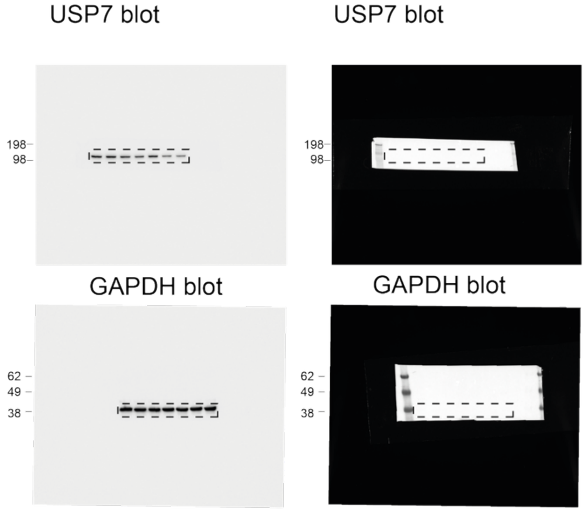

Supplementary Fig. 2g

Panc89

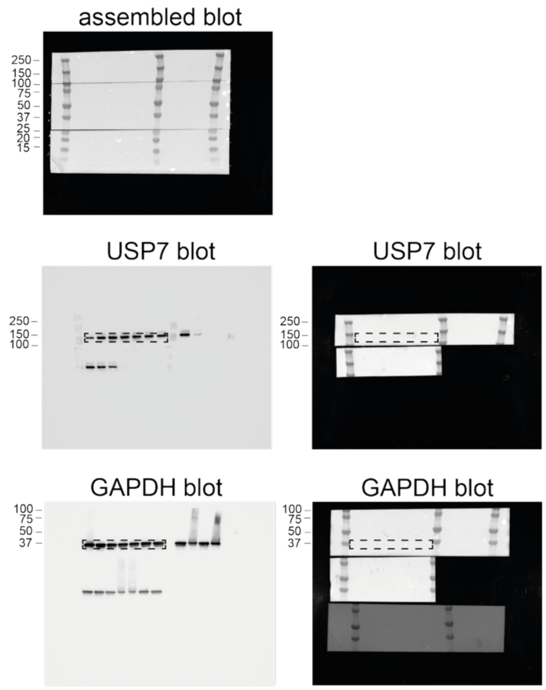

Supplementary Fig. 2h

Ma-Mel-47

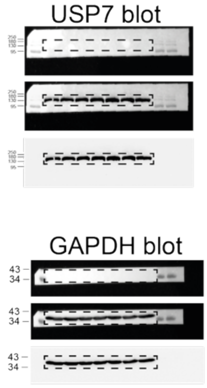

Supplementary Fig. 7e

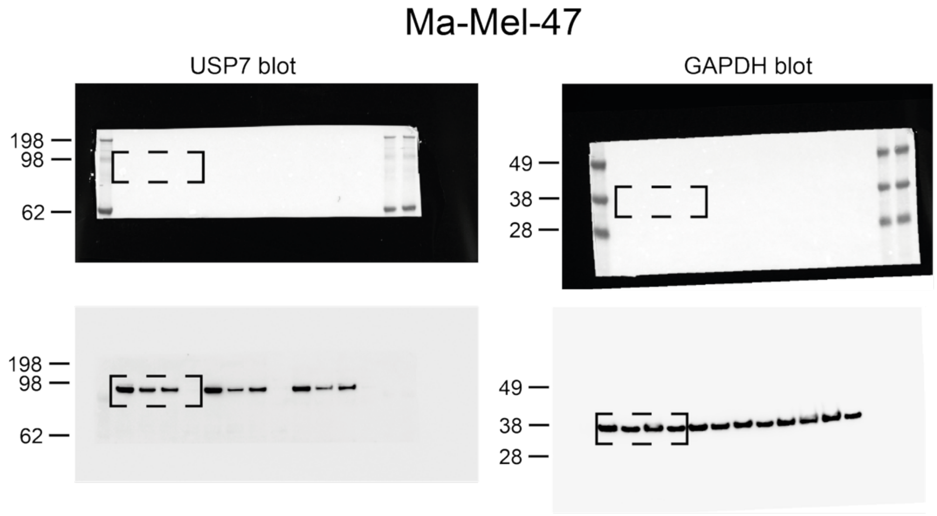

Supplement: Supplementary file 10 — Source Data [file 41467_2026_72295_MOESM10_ESM.zip › Source_Data_Blots.pdf]
